# Climate change will redefine taxonomic, functional, and phylogenetic diversity of Odonata in space and time

**DOI:** 10.1038/s44185-022-00001-3

**Published:** 2022-11-17

**Authors:** Tommaso Cancellario, Rafael Miranda, Enrique Baquero, Diego Fontaneto, Alejandro Martínez, Stefano Mammola

**Affiliations:** 1 University of Navarra, Biodiversity and Environment Institute BIOMA, Irunlarrea 1, 31080 Pamplona, Spain; 2 Molecular Ecology Group (MEG), Water Research Institute (IRSA), National Research Council of Italy (CNR), Verbania, Italy; 3 Laboratory for Integrative Biodiversity Research (LIBRe), Finnish Museum of Natural History (Luomus), University of Helsinki, Helsinki, Finland

**Keywords:** Biodiversity, Biogeography, Climate-change ecology, Community ecology, Ecological modelling, Freshwater ecology

## Abstract

Climate change is rearranging the mosaic of biodiversity worldwide. These broad-scale species re-distributions affect the structure and composition of communities with a ripple effect on multiple biodiversity facets. Using European Odonata, we asked: i) how climate change will redefine taxonomic, phylogenetic, and functional diversity at European scales; ii) which traits will mediate species’ response to global change; iii) whether this response will be phylogenetically conserved. Using stacked species distribution models, we forecast widespread latitudinal and altitudinal rearrangements in Odonata community composition determining broad turnovers in traits and evolutionary lineages. According to our phylogenetic regression models, only body size and flight period can be partly correlated with observed range shifts. In considering all primary facets of biodiversity, our results support the design of inclusive conservation strategies able to account for the diversity of species, the ecosystem services they provide, and the phylogenetic heritage they carry in a target ecosystem.

## Introduction

Recent climate change is driving the reshuffling of the biodiversity patchwork on the Earth^1^. Upon those abrupt global changes, few species can survive in situ by adapting to the novel environmental conditions, whereas many more are forced to shift their ranges tracking their eco-physiological optima for growth and survival^2,3^. Never before a single human generation witnessed such a rapid and massive biological migration induced by the increase of temperature, with terrestrial species rising towards higher latitudes and elevations and marine life sinking at greater depths^4–6^. Inevitably, these rapid readjustments in species ranges are leaving a considerable imprint on the structure of local communities, which has cascading effects on ecosystem functioning and the provisioning of nature’s contribution to human societies^7–9^. The ecological and economic impacts of these changes are expected to be unprecedented^10^.

Climate changes will lead to cumulative non-linear responses in the biological assemblages, permeating through all biodiversity facets. This is because as climate changes, so does the distribution of certain species, with a ripple effect on species richness, trait composition, and evolutionary heritage of local communities^11–13^. Therefore, the impact of climate change can be quantified by predicting changes in the number of species (hereinafter “taxonomic diversity”), traits (“functional diversity”) and evolutionary lineages (“phylogenetic diversity”) that are present in an ecosystem. As approximative as the approach might be, a quantification of the spatial and temporal rearrangement of these metrics is paramount to understand causally the mechanisms that drive the evolution of biodiversity across its multiple facets. Given that taxonomic, functional, and phylogenetic biodiversity are linked with ecosystem functioning and stability, ecologists and conservation biologists are increasingly considering these three facets when designing conservation plans^13^.

The potential effects of environmental constraints on biological aggregations can be reflected in the variation of *α* diversity, which summarises community structure as the total richness of taxa, traits, and evolutionary history^14–17^. Additional features that are affected by environmental perturbations include community composition, potentially allowing causal understanding of the mechanisms that may regulate the effects^18^. The calculation of *β* diversity, which traces the individual elements that change across biological communities, can further be decomposed into its replacement and richness components^19^.

**Fig. 1:**
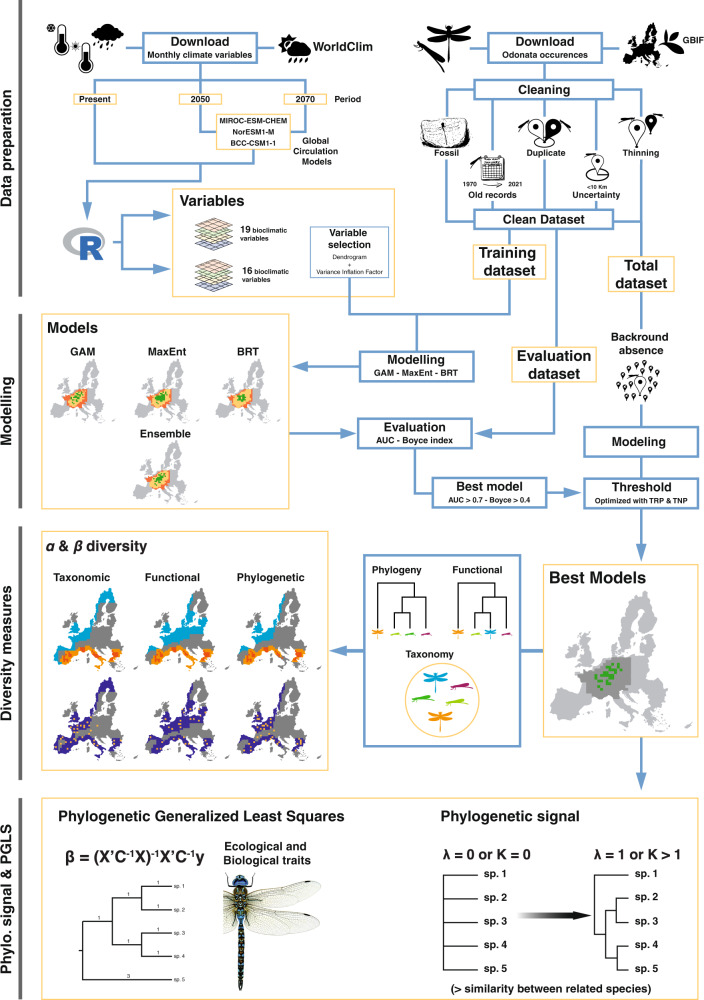
Infographic summarising the study workflow. In this work, we first constructed a species distribution model for each species of European Odonata to predict their current and future habitat suitability. Then, we stacked the model projections and used community-level data (*α* and *β* diversity) to quantify the temporal variation of taxonomic, functional, and phylogenetic diversity. Finally, we used the predicted range shift to assess whether the response of Odonata to climate change is driven mainly by their evolutionary history or by distinctive biological and ecological traits.

In this context, we described the spatio-temporal effects produced by the shift of habitat suitability induced by climate changes on three biodiversity facets, incorporating both *α* and *β* diversity metrics. First, we modelled how global warming will affect the habitat suitability of each European species of Odonata during their imaginal stage. Second, we evaluated how the predicted changes in species habitat suitability will influence the taxonomic, phylogenetic, and functional diversity of Odonata communities in space and time (Fig. 1). Third, we used the predicted range shift to assess whether the response to climate change of Odonata (including both the sub orders Anisoptera and Zygoptera) is driven mainly by the evolutionary history or by distinctive biological and ecological traits. We chose dragonflies and damselflies because they are well-established model organisms to address general macroecological questions in global change biology^20,21^ and thermal physiology^22,23^, being even regarded as “barometers” for climate change^20^. Furthermore, odonates play a key ecological role in most food webs since they are both predators and prey, delivering important ecological services^24^.

Under the assumption that Odonata species will disperse tracking their ecological optima, we tested three hypotheses. (1) Species will redistribute poleward along the latitudinal gradient and upward along the altitudinal gradient. (2) Those changes will affect community composition and will permeate phylogenetic and functional components. Specifically, we expect that *α* diversity will increase in areas with more conservative climate, whereas *β* diversity will change more in areas experiencing faster climate change rates, especially so in the *β* richness component given the high dispersal ability of Odonata^23^. (3) The response of Odonata to climate change will also be explained by a shared evolutionary history, since phylogenetically related species may have similar patterns of distribution change and similar biological and ecological traits related to their dispersal ability.

## Results

### Species distribution models

#### Predictor variables and model performance

Using species distribution models (SDM), we successfully calculated the habitat suitability for 107 species of European Odonata (63% of the 169 species contained in our checklist). We omitted 62 species due to the low number of occurrences available in Global Biodiversity Information Facility (GBIF). Our models incorporated seven non-collinear predictors (results of multicollinearity analysis are in Supplementary Material 1): water bodies, elevation, Emberger’s pluviometric quotient (embergerQ), temperature annual range (bio 7), mean temperature of the wettest quarter (bio 8), mean temperature of the warmest quarter (bio 10), and precipitation seasonality (bio 15). Boosted Regression Trees attained the greatest Area Under the Receiver Operator Curve (AUC) values in 96 species, the ensemble of models in six species, the Maximum Entropy in four, and the Generalised Additive Model only in one (Supplementary Materials 2).

#### Species distribution model future predictions

**Fig. 2:**
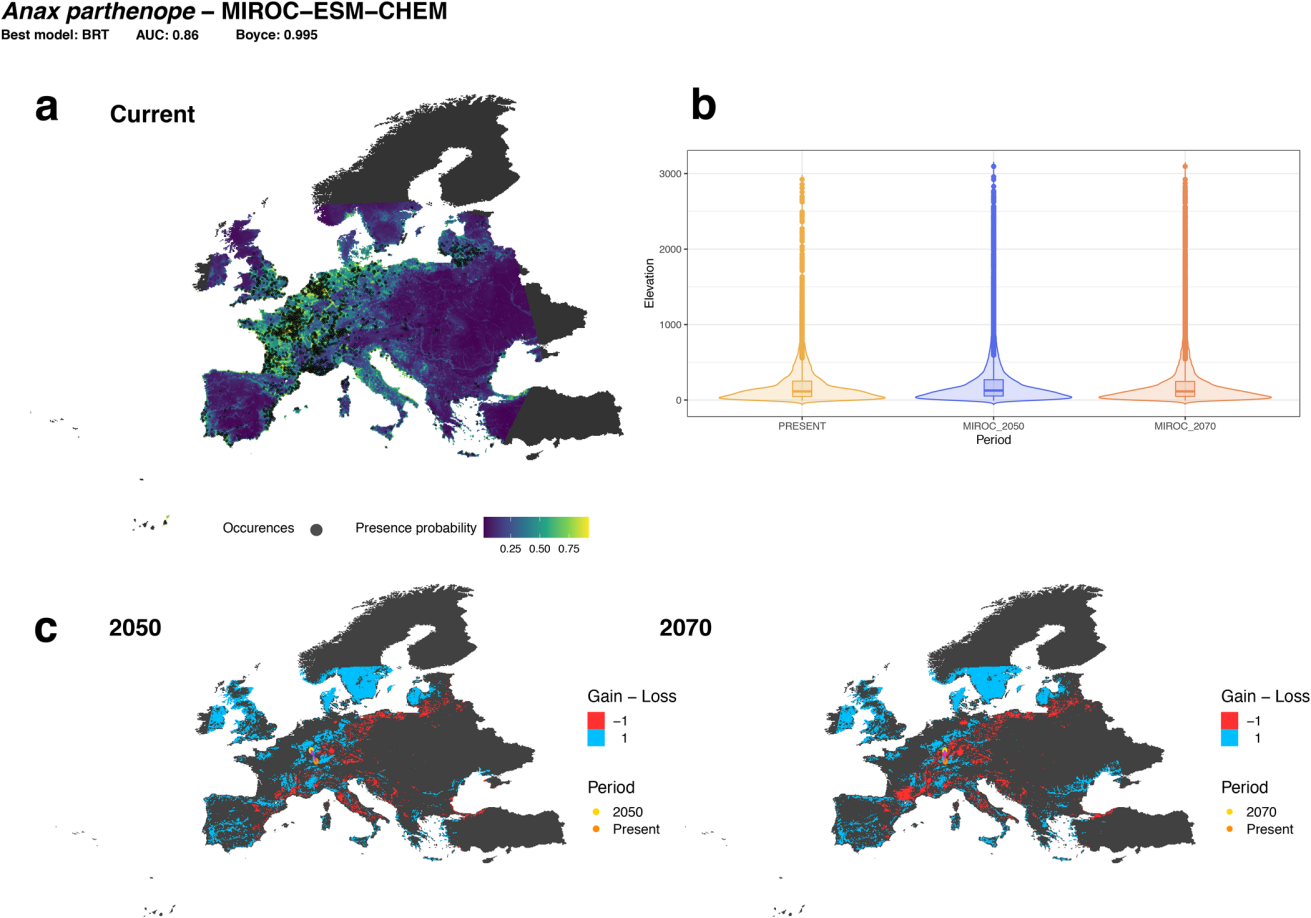
Example of summarised species distribution model (SDM) projections for an individual odonate species. **a** Best model prediction map for the current time period. **b** Extent of elevation shift across time periods. **c** Variation of habitat availability between future and current time periods. Habitat gain and loss are depicted with blue and red colours respectively. Centroid shift is represented by the variation among the orange (present) and yellow point (future). In the box plots, the box indicates the inter-quartile range (25–75th percentile); the bold line is the median; the upper and lower whiskers extend from the hinge to the largest and smallest value no further than 1.5 * inter-quartile range; data beyond the end of the whiskers are outliers. Summarised SDM outcomes for all species are available in Supplementary Material 3.

In accordance with our first hypothesis, we consistently predicted an increase in habitat availability towards northern European regions and upper elevations for most species (Table 1). These shifts were coupled with a contraction of suitable habitats in the Mediterranean region. Most species of Odonata experienced a shift in the centroid of their distribution towards northern latitudes (Table 1), as well as a rise of their preferred mean elevation in the future climate scenarios (Table 1). These predictions varied minimally under different Global Circulation Models. Example model projections for one of the species is available in Fig. 2 (Supplementary Material 3 for the entire set of species).

**Table 1:** Average (± Standard Error) shift in species distribution toward northward latitudes and upper altitudes.

Average northward shift in centroid latitude		
	2050	2070
BCC-CSM1-1	0.93 ± 0.11 (91/107)	1.12 ± 0.12 (86/107)
MIROC-ESM-CHEM	0.49 ± 0.11 (73/107)	0.70 ± 0.13 (81/107)
NorESM1-M	0.81 ± 0.11 (80/107)	0.37 ± 0.11 (70/107)

Average altitudinal shift in metres		
BCC-CSM1-1	37.14 ± 5.10 (81/107)	33.43 ± 6.08 (73/107)
MIROC-ESM-CHEM	61.56 ± 6.28 (88/107)	67.41 ± 6.78 (91/107)
NorESM1-M	44.44 ± 5.09 (90/107)	56.43 ± 5.47 (93/107)

In parenthesis, the number of species (out of the total modelled species) shifting northward latitude and upper altitudes is reported.

### Quantification of change of biodiversity measures

We calculated taxonomic, functional, and phylogenetic diversity for 105 of the 107 species, since we lack genetic data for *Orthetrum taeniolatum* (Schneider, 1845) and *Sympetrum sinaiticum* Dumont, 1977.

#### *α* diversity patterns

**Fig. 3:**
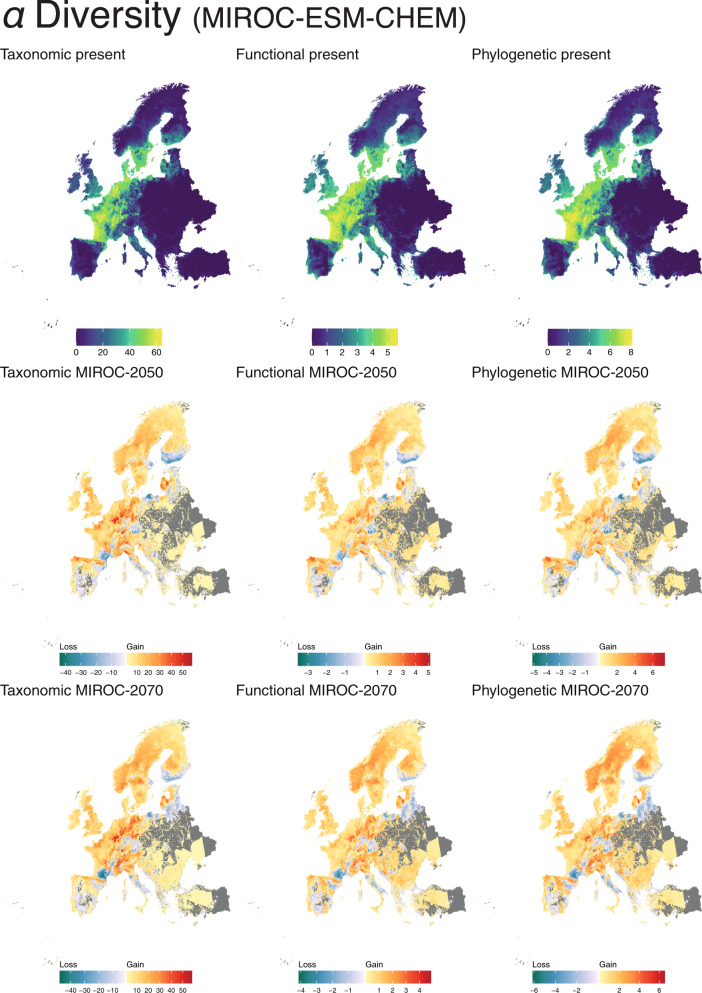
Quantification of *α* diversity per different time period (current; 2050; 2070) and biodiversity facets (taxonomic, functional and phylogenetic) under the climate scenario MIROC-ESM-CHEM. For future scenarios, the cold-colour gradient indicates the extent of species loss, whereas the warm-colour gradient indicates the species gain. See Supplementary Material 4 for BCC-CSM1-1 and NorESM1-M climate scenarios.

Current *α* diversity patterns were overall congruent across the three biodiversity facets. The highest taxonomic diversity concentrated around the Central-Atlantic European region, whereas the highest values for functional and phylogenetic diversity were attained in Italy, Ireland, and the North of the United Kingdom (Fig. 3).

Future *α* diversity projections revealed an increase in taxonomic, functional, and phylogenetic diversity in northern and eastern Europe, particularly in the Fennoscandian peninsula, the British Isles, and around the Black Sea. In contrast, these three metrics are predicted to decrease in Central Europe and the Mediterranean, particularly in France, Germany, and the Baltic countries, as well as the Hellenic, Italian, and Iberian peninsulas. Furthermore, all three *α* diversity metrics are predicted to increase towards higher altitude, particularly in the Alps, Cantabrian mountains, and the Pyrenees (Fig. 3; Supplementary Material 4).

#### *β* diversity patterns

**Fig. 4:**
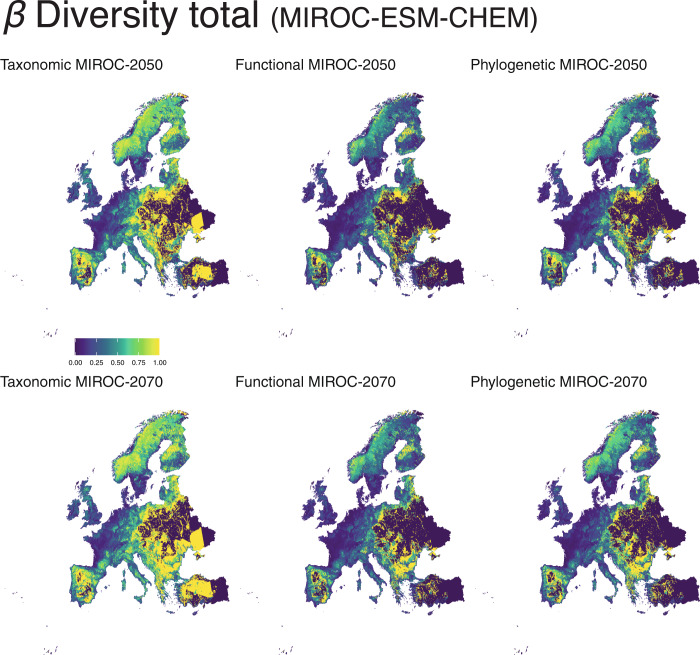
Quantification of total β diversity (β replacement + β -richness^19^) per different time period (current; 2050; 2070) and biodiversity facets (taxonomic, functional and phylogenetic) under the climate scenario MIROC-ESM-CHEM. See Supplementary Material 4 for BCC-CSM1-1 and NorESM1-M climate scenarios.

We observed greater values of taxonomic, functional, and phylogenetic metrics of *β* diversity in the Iberian Peninsula, Scandinavia, and in scattered areas across western Europe. We obtained congruent patterns across future Global Circulation Models, and between 2050 and 2070 predictions (Fig. 4). These changes were primarily due to variations in the richness component of *β* diversity, rather than in the replacement component. The highest *β* diversity richness values were predicted for the Iberian Peninsula, Turkey, Scandinavia, and Eastern Europe; those for *β* diversity replacements were predicted in Iberia, as well as the Balkans, and the Baltic countries (Fig. 4; Supplementary Material 4).

### Phylogenetic signal and phylogenetic generalised least squares

**Fig. 5:**
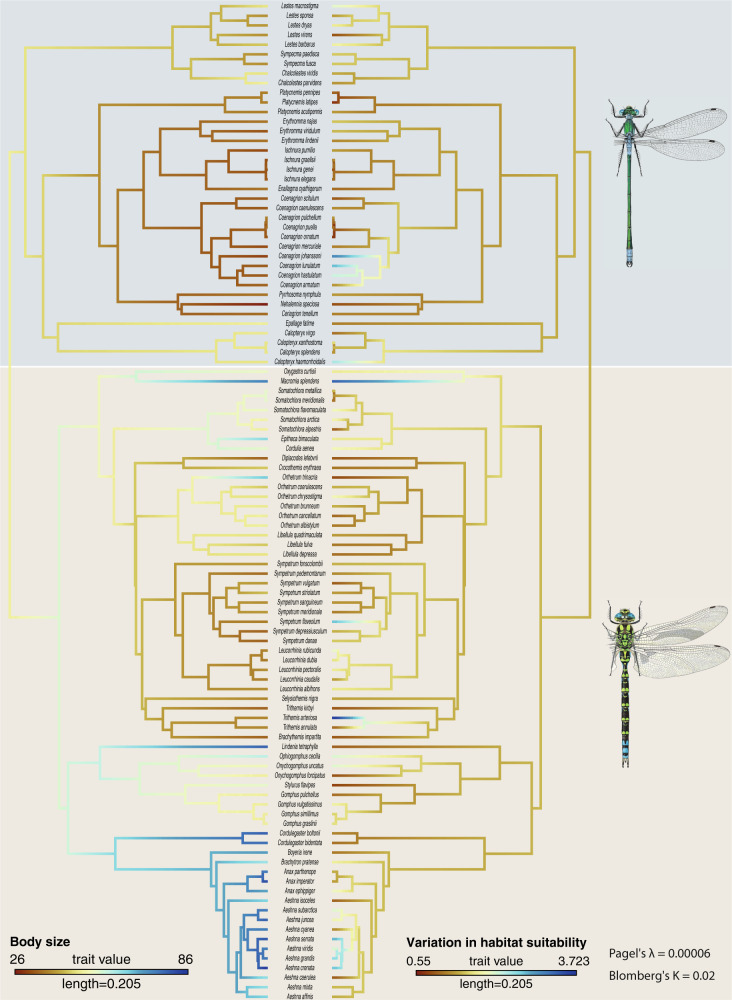
Reconstruction of ancestral character states for the variables body size (left) and variation in habitat suitability (right). Pagel’s λ and Blomberg’s K indicate the estimated values for the response variables “Variation of habitat suitability” (see Supplementary Material 6 for the other tree of ancestral character reconstructions). “Length” in the legend provides the scale for the branch lengths of the phylogenetic tree. The grey box delimits the Zygoptera clade whereas the brown one the Anisoptera clades.

Pagel’s λ and Blomberg’s K were non-significant and low for all predictors (relative area change; altitude difference; centroid difference), scenarios (BCC-CSM1; MIROC-ESM-CHEM; NorESM1-M) and time periods (2050; 2070) (*p* > 0.01, *K* < 0.1, λ < 0.5) (Supplementary Material 5), indicating no phylogenetic signal in the response of Odonata to climate change. These results were consistent whether they were calculated for the phylogeny of the Odonata or its suborders Anisoptera and Zygoptera separately. Exceptionally, Pagel’s λ was relatively high and significant (λ = 0.73; *p* = 0.003) for the centroid shift in the MIROC-ESM-CHEM 2050 climate change scenario, when calculated over the entire phylogeny of Odonata. In congruence, ancestral character reconstruction revealed no clear patterns of change in of our trees (an example in Fig. 5, Supplementary Material 6).

Phylogenetically explicit least-square models (PGLS) revealed that only body size and duration of flight period significantly affected the proportional variation in habitat suitability and the centroid shift, but only for some of our global circulation models and time periods (Table 2; Supplementary Material 7). The influence of traits on range shift becomes more pronounced, although more complex to interpret, when we considered the orders Anisoptera and Zygoptera separately. The response of Anisoptera to climate changes was significantly affected by their body size, flight period, and preference for lentic habitat; the response of Zygoptera was affected by their flight period and preference for lotic habitat (Supplementary Material 7).

**Table 2:** Results of phylogenetic generalised least square (PGLS) models. Significant effects are highlighted in bold.

	Estimate	Std. Error	*t* value	Pr(>|t|)	Res. variable	Scenario	Time period
(Intercept)	207228.6	52990.2	3.9	0.0001	Centroid difference	MIROC	2050
Body length	−1828.1	606.9	−3.0	**0.0032**	Centroid difference	MIROC	2050
Flight Season	2777.5	5808.9	0.5	0.6336	Centroid difference	MIROC	2050
Habitat Lentic	12534.6	31484.4	0.4	0.6913	Centroid difference	MIROC	2050
Habitat Lotic	17994.4	34691.4	0.5	0.6051	Centroid difference	MIROC	2050
(Intercept)	253527.6	63388.1	4.0	0.0001	Centroid difference	MIROC	2070
Body length	−1767.6	709.5	−2.5	**0.0143**	Centroid difference	MIROC	2070
Flight Season	1037.7	6738.3	0.1	0.8779	Centroid difference	MIROC	2070
Habitat Lentic	7731.6	39206.2	0.2	0.8440	Centroid difference	MIROC	2070
Habitat Lotic	8406.5	42340.7	0.2	0.8430	Centroid difference	MIROC	2070
(Intercept)	1.9	0.3	5.8	7.23E-08	Relative area change	BCC	2070
Body length	0.0	0.0	0.4	0.6580	Relative area change	BCC	2070
Flight Season	−0.1	0.0	−3.6	**0.0005**	Relative area change	BCC	2070
Habitat Lentic	0.3	0.2	1.5	0.1337	Relative area change	BCC	2070
Habitat Lotic	0.2	0.2	1.1	0.2877	Relative area change	BCC	2070
(Intercept)	1.2	0.3	3.7	0.0003	Relative area change	MIROC	2050
Body length	0.0	0.0	2.1	**0.0344**	Relative area change	MIROC	2050
Flight Season	−0.1	0.0	−1.7	0.0844	Relative area change	MIROC	2050
Habitat Lentic	0.3	0.2	1.3	0.2064	Relative area change	MIROC	2050
Habitat Lotic	0.0	0.2	0.1	0.9009	Relative area change	MIROC	2050
(Intercept)	1.7	0.3	6.6	1.63E-09	Relative area change	NOR	2050
Body length	0.0	0.0	0.8	0.4473	Relative area change	NOR	2050
Flight Season	−0.1	0.0	−3.5	**0.0007**	Relative area change	NOR	2050
Habitat Lentic	0.2	0.1	1.4	0.1539	Relative area change	NOR	2050
Habitat Lotic	0.2	0.2	1.4	0.1510	Relative area change	NOR	2050

Total table containing PGLS results is in Supplementary Material 7.

## Discussion

We forecasted variations in habitat availability for 107 species of European Odonata, predicting conspicuous future readjustments of their *α* and *β* diversity at European scale across taxonomic, functional, and phylogenetic diversity facets. The relation of those readjustment to species phylogenetic position and traits were complex but, overall, the magnitude of range shifts across the evolutionary tree of European Odonata exhibited low phylogenetic signal and poor relationship to the considered functional traits.

### Biodiversity change: a multifaceted problem with non-linear responses at European scales

Odonate communities will not reshuffle randomly: according to our predictions, habitat suitability will increase towards northern latitudes and upper elevations. These changes will be coupled with a contraction of suitable areas in the Mediterranean for most species. Similar shifts have been observed for many freshwater invertebrates^25–28^ although their effects on the structure and composition of natural communities remains poorly documented. Consequently, odonate communities will face a future taxonomic rearrangement, paralleled by alteration of ecosystem dynamics and functioning^9^. The congruence across biodiversity metrics suggests that taxonomic diversity could be used as a proxy to predict change in phylogenetic and functional diversity metrics—which are generally more difficult to estimate. Importantly, this congruence in biodiversity patterns simplifies designing conservation strategies^29^. A similar congruent relation across biodiversity metrics has been found in other organisms and ecosystems, such as corals^30^, ants^31^, and freshwater fish^32^, but never in freshwater invertebrates. These results strengthen our increasing awareness of the indissoluble relation that links ecosystem functioning and human societies. Biodiversity loss will indeed permeate all its facets leading to tilting consequences on humankind. Being irreplaceable nodes in ecological networks as much as providers for uncountable ecosystem services, freshwater invertebrates must not be left out from future climate actions^33,34^.

Biodiversity changes depend on non-linear species interactions branching throughout the ecosystem and sometimes involving several temporal and spatial scales. This complexity is not easily predictable using correlative methods^35^. For instance, increasing taxonomic, functional, and phylogenetic diversity might import new evolutionary lineages to a given ecosystem, thereby improving its resilience with novel functions^36^. Concomitantly, arriving new species might bring new possibilities for biotic interactions, which are often difficult to anticipate^37^. In damselflies, this has been illustrated by the climate-driven expansion range in the Iberian Peninsula of *Ischnura elegans* (Vander Linden, 1820), which has hybridised with the previously reproductively isolated species *Ischnura graellsii* (Rambur, 1842)^38^. The hybrids represent new forms originated in situ, which bring original functions to the ecosystems and unpredictable consequences in the native communities^39^.

In contrast, decreasing taxonomic, functional, and phylogenetic diversity metrics could reduce ecosystems’ stability and resilience by narrowing possible species-specific responses to environmental fluctuations. This often leads to a functional homogenisation^40^ and a reduction of genetic diversity^41^. For example, the climate-change driven arrival of highly mobile generalist species paralleled by the disappearance of habitat specialised species has caused the homogenisation of the odonate communities in North America^42^. Similar processes of homogenisation driven by habitat alteration and anthropogenic pressures has been registered for European freshwater invertebrates^43^, including in Odonata communities^44^. These examples collectively illustrate the complex interplay of the three diversity metrics in natural ecosystems. Therefore, predicting how changes in those metrics might affect long term ecosystem function and stability is challenging and not only requires high-quality data, but also mechanistic modelling^45^.

### Species distribution models: a useful tool for climatic predictions, but not without caveats

Species distribution models are robust and reliable approaches to map species distributions in space and time, although bearing in mind key limitations. The outcome of these models is unavoidably coupled with the goodness of the ecological variables selected for their calculation^46^. In this study, our models lack variables related to specific habitats, such as presence of intermittent freshwater habitats or changes in the future extension of water bodies. Instead, they largely rely on the use of climatic variables, mostly because they are more readily available in public databases. As such, the projected outcomes from species distribution models must be interpreted as general indications of future trends, rather than as precise descriptions of species range boundaries.

Another caveat comes from the availability of reliable occurrences for the target species. A valid criticism to our approach is that we entirely rely on species data retrieved from GBIF. In our case, some of the limitations associated with GBIF data (e.g., samples collected opportunistically and spatially distorted) are alleviated by the relatively large size and the popularity of dragonflies amongst entomologists, such that information available in GBIF is arguably less biased compared to the actual knowledge available for other freshwater groups. Taxonomic identification errors are another pervasive problem found in GBIF datasets, but, again, this issue is limited for odonates due to the availability of high-quality field guides and the facility to recognise the adult stages. Despite of entirely relying on GBIF, our results of the current *α* taxonomy agree with the distribution map proposed by Kalkman et al.^47^.

We acknowledge that other sources of records are available in the literature, but they are generally non-digitalised, and the information provided is not standardised geographically and taxonomically. Since those are important limitations for the use of many sources in large scale macroecological projects, we urge authors with available occurrence and functional data to make it available in public databases.

Finally, we only accounted for dispersal dynamics indirectly by restricting the model calibration area based on a proxy measure of dispersal potential for each species^48^. Alternatives to incorporate dispersal in SDM are available^49^ and are increasingly used in invertebrates^45^, but we were forced down this modelling road due to broad limitations in terms of available data. Concerning dispersal, we also consider Europe as a close area, not susceptible to the arrival of immigrant species from Africa or Asia, which leads to an underestimation of the future biodiversity facets, particularly in the Mediterranean basin. Whether this is a realistic assumption or not, all species distribution models need to depart for a limited geographical area. Therefore, rather than a problem linked to our specific approach, this is an axiom of our analyses that needs to be accounted for during the interpretation of the results. Enlarging our study area towards Africa and Asia would be certainly interesting but would also introduce new limitations to our predictions given the strong biases affecting occurrence data available for those areas when compared to Europe.

### The challenge of predicting collective responses from individual species traits

Our analyses indicate a collective shift of odonates poleward and towards upper altitudes in response to climate change^5,50,51^. However, this is not true for all the species included in our analyses, insofar as a few of them exhibited responses that deviated from these average predictions^3^. Hence, we explored whether these individual responses could be explained by the phylogenetic position and functional traits of each individual species, to connect the properties of the individual elements of our system and its collective response. Connecting the role of phylogenetic and functional traits in the responses of individual species and how such changes may affect biological communities is also critical to designing effective management and conservation plans^44^.

Phylogenetic relatedness did not explain the future shift of the European Odonates, given that we found no strong phylogenetic signal in any of the metrics that we calculated to characterise their change in range shift. These results contrast with those proposed by Pinkert et al.^52^, who found a robust phylogenetic conservatism related to thermal preference by studying the changes in European odonate communities since the Last Glacial Maximum. However, these results are hardly comparable to ours due to the different time periods (thousands of years versus decades) and climatic events analysed (ice retraction versus global warming). In our case, the current climate change imposes a very quick migration for most species, which seems to respond with independence to the phylogeny. This seems to be congruent with Castillo-Pérez et al.^23^, who documented a differential response of odonates to temperature with strong variations at species and population levels in changing environments.

In contrast to the phylogeny, the relationships between species traits and ranges shifts were complex, largely depending on the traits, response variables, and the global circulation models under consideration. This variability somehow agrees with the contrasting responses predicted across species and populations in previous studies^23^. Body size and flight period explained the changes in the range shift across the entire Odonata but not consistently across all climatic scenarios, neither when only the species in the suborder Zygoptera or Anisoptera were analysed. These results contrasts with those proposed by Grewe et al.^21^, where neither biological (e.g., abdomen length and wing size) nor ecological (e.g., flight period) traits have returned significant relation with observed range shift. Differences in modelling methods and trait sources may account for this mismatch.

Due to the complexity of these outcomes, we prefer not to provide strong biological conclusions from our results. We only cautiously reaffirm that biological and ecological traits seem to explain some degree of variability in species-specific responses to climate change^53^, opening a door for further studies aiming at a more mechanistic trait-based understanding of these phenomena. Future investigations based on high-resolution physiological adaptations and dispersal abilities (e.g., GPS-tracking, flight muscle mass, wing loading and shape, temperature tolerance)^45,54^, and possibly incorporating traits and phylogenies explicitly into the modelling pipeline (e.g., ref. ^55^), might reveal key traits and mechanisms associated with species’ climate-induced responses. Traits obtained from larval stages might also be informative, since most of the life of these insects is spent underwater [e.g., in *Anax imperator* (Leach, 1815) the life span is two years in larvae and eight to nine weeks in adults^56^]. Unfortunately, these additional sources of information remain scarcely available for most odonate species, forcing us to exclude them from the analysis. This scarcity reminds us of the importance of basic research into the natural history of most Odonata species as a tool to design more effective conservation strategies.

## Methods

### Rationale

To model species distribution, we used SDMs, mainstream analytical tools in ecological and biogeographical research^57–59^. Due to the easy implementation and the often accessible interpretation of results (but see ref. ^60^), SDMs are routinely used in disciplines as diverse as conservation planning^61^, habitat restoration^62^, invasion biology^63,64^, and climate change biology^65,66^. In short, distribution modelling refers to the practice of using an algorithm to infer a relationship between the occurrences for a given species (e.g., georeferenced points) and environmental predictors (e.g., climatic variables, topographic parameters, habitat type), forecasting its potential distribution in space and/or time.

As a model organism, we selected Odonata, an order of insects with tropical evolutionary origin^67^ and including species with contrasting thermal preferences. Odonata are well-established model organisms in ecology and behaviour^68–70^, and have been successfully used for tracking climate change using species distribution models^20^. These insects have an amphibiotic life with benthic vagile larvae living in freshwater habitats; the adults are excellent fliers with high dispersibility compared to other freshwater invertebrates^71^. Broadly within the Odonata, generalist and lentic species have greater dispersal abilities than specialist and lotic species. Furthermore, comparing the odonates suborders, usually, Anisoptera disperse more than Zygoptera^72^.

### Taxonomic checklist and assembly of distribution data

We produced a complete checklist of all 169 European Odonata by merging the information of the “Atlas of the European dragonflies and damselflies”^73^ and the field guide “Dragonflies of Britain and Europe”^74^ (Supplementary Material 8). These are the most comprehensive references for European Odonata available today. We focused on the European continent because it has been intensively studied compared to other areas of the world^75^. We excluded European Russia (including Kaliningrad) due to the scarcity of Odonata occurrences therein. We included Turkey to account for the entire arch of northern Mediterranean countries.

We downloaded all georeferenced occurrences of Odonata available at the GBIF (09 January 2021; DOI: 10.15468/dl.kvrqug). Despite its biases^76^, GBIF remains one of the most extensive global biodiversity databases^77^. The coverage provided by GBIF (highest coverage in UK, France, the Netherlands, Austria, and Germany; lowest in southern and eastern Europe) for Odonata is congruent with the current expert-based knowledge about European odonates^21,47^. If not specified otherwise, we assumed that occurrences were adult stages.

We discarded data for fossil, non-European species, records before 1970, and occurrences falling outside the study area. We also removed duplicates and records with spatial uncertainty greater than the resolution of our predictor variables (~10 km; see section “Selection of environmental predictors”). We minimised the effects of uneven sampling effort *via* spatial thinning with the function *reduceSpatialCorrelation* from the pack SDMworkshop (https://github.com/BlasBenito/sdmflow), setting the *minimum.distance* parameter to 1 (~10 km) to match the resolution of our predictors.

### Accessible area delimitation

For each species, we calibrated models within an accessible area^48^. The accessible area is the geographic extent hypothesised to fall within the long-term dispersal potential for a particular species over its evolutionary history. It is often a broader area than the one reachable through dispersal within a single generation. In multi-species analyses, when lacking detailed information on species biogeographic history and dispersal ability, the simplest way to limit the boundary of the accessible area is by constructing a continuous border where most of the occurrences of a taxon are contained. For this, we used a Minimum Convex Polygon, the smallest area surrounding the points in which every internal angle does not exceed 180°^78^. We estimated a conservative Minimum Convex Polygon for each species using the R function *mcp* from the package *adehabitatHR* v0.4.19^79^, setting the percentage of outliers to be omitted at 1%. Finally, to account for the fact that different species vary in their dispersal ability, we created an external buffer around each accessible area, weighting the distance with the flight period of each species [100,000_distance in metres_ * (Flight period in months/10)].

### Selection of environmental predictors

We downloaded four variables from WorldClim 2^80^: monthly minimum and maximum temperature (°C), monthly precipitation (mm), and Digital Elevation Model (m a.s.l.). Current climatic data are the average for the period 1970–2000. We retrieved the water bodies’ map from the FAO’s GeoNetwork data portal. This raster map includes lentic and lotic permanent water body at world scale. We adjusted the resolution of the water bodies’ map to 5 min (~10 km) using the function *resample* from the R package *raster* v3.5-2^81^ setting ‘bilinear’ method. Starting from the three climate variables (min/max temperature and precipitation), we calculated 19 bioclimatic variables using the function *biovars* from the R package *dismo* v1.3-3^82^ and 16 environmental variables using the function *layerCreation* from the package *envirem* v2.3^83^. More information about the latter variables can be retrieved at https://www.worldclim.org/data/bioclim.html and https://envirem.github.io.

We visualise the multicollinearity effect amongst our 37 predictors variables (19 bioclimatic, 16 environmental, elevation, water bodies) via pairwise Pearson’s *r* correlation and a dendrogram based on variables’ distance matrix^84^. We extracted the final set of predictor variables at |*r*| < 0.5^85^ and then we removed variables with a Variance Inflation Factor (VIF) > 3^86^.

We downloaded the same predictors for three future climate scenarios (Global Circulation Models: BCC-CSM1; MIROC-ESM-CHEM; NorESM1-M) and two time periods, 2050 (average for 2041–2060) and 2070 (average for 2061–2080). We chose a moderate Representative Concentration Pathway (RCP 4.5), namely a scenario that accounts for the greenhouse emission according to the current green policies^87^. We assumed elevation and water bodies to remain constant in the future.

### Modelling procedure

To model the distribution, we selected one algorithm for each main family of modelling algorithms (regression, maximum entropy, and decision trees)^88,89^. We opted for Generalised Additive Model^90^, MaxEnt^91,92^, and Boosted Regression Trees^93^, respectively, given their high performance^94^. Furthermore, we compared the performance of each individual algorithm with an ensemble model, computed with the function *calc* in the package *raster*, since the aggregation of forecasts of different models (ensemble model) may improve the prediction habitat suitability of a given species^95,96^. Specific settings and parameters for each algorithm are available in Supplementary Material 9. To discriminate the areas where each species was more likely to be absent, we contrasted the presence data against a set of background points generated within their buffered accessible area. The number of background points doubled the number of presences^97^.

We evaluated the model performance using a holdout approach, whereby we used 75% of the occurrences of each species as a “train” dataset and the remaining 25% as “test” dataset to evaluate their predictive power. We calculated two performance metrics: AUC and Boyce index^98^. The AUC values range from 0 to 1, with higher values indicating better model discrimination. Whereas this metric is problematic for determining the absolute performance ability of SDMs, it is acceptable to use it for relative comparisons across models fitted with the same data^99^. The Boyce index is considered one of the most appropriate model evaluation metrics when absence data are lacking^98^, and thus we chose it as a *proxy* measure of absolute model performance. The continuous Boyce index varies from –1 to 1: values above zero indicate model predictions consistent with distribution data, values around zero indicate performance no better than random, and values below zero refer to incorrect model predictions^98^. We considered predictions with AUC < 0.7 and/or Boyce < 0.4 as low-quality performance.

After their evaluation, we fitted a final model for each species with the complete set of occurrences and used it to project potential distribution ranges under current and future climates. We converted the continuous habitat suitability projections into binary maps by using a threshold maximising the sensitivity (True Positive Rate) and specificity (True Negative Rate)^100,101^. We calculated both spatial (e.g., suitable range size, mean elevation, and centroid) and biodiversity measures (see the next paragraph for biodiversity measures) only on the binary maps obtained from the best-performing modelling method^102^.

In constructing and reporting SDMs, we followed the ODMAP (Overview, Data, Model, Assessment and Prediction) protocol^103^, designed to maximise reproducibility and transparency of distribution modelling exercises. The ODMAP for this study is available as Supplementary Material 9.

### Estimation of taxonomic, functional, and phylogenetic diversity metrics

We calculated three diversity metrics for the predicted community of Odonata occurring within a cell of each raster map. We first stacked SDM projection for all the analysed species. We estimated taxonomic diversity as the number of species predicted to occur in each cell. We calculated functional and phylogenetic diversity as the total branch length entailed by the species predicted to occupy each cell, based on a functional and phylogenetic tree^16,104–106^ (see next sections). We chose tree-based descriptors of relationships to make the formulation of functional and phylogenetic diversity more comparable^107^.

#### Estimation of the functional dendrogram

We calculated the functional tree for European Odonata using six traits broadly related to dispersal and species response to climate change, namely: total body size (mm), abdomen length (mm), wings length (mm), abdomen pigmentation (in RGB), habitat (lentic or lotic), and flight season time (in months) (Table 3). We focused on the adult stage because they disperse at large spatial scales^108^ via morphological (e.g., wings) and behavioural (e.g., reversible polarotaxis, repulsion/attraction of polarised light^109^). In contrast, larva might disperse as well, but its ability is limited to the aquatic environment. Therefore, we expect that immigration promoted by climate change will involve mainly adults.

We determined the male abdomen pigmentation from three pictures of each species, preferably downloaded from Dragonflypix (http://www.dragonflypix.com/index.html). We clipped the image around the abdomen using the software Gimp^110^ and extracted the RGB colour-space using the function *getImageHist* (*colordistance* v1.1.2^111^). We obtained the mean value of the abdomen colour for each species as the average of the two predominant colours on the three photos (data available at https://osf.io/swnu4/download).

We calculated functional dendrograms^16^ with the *hclust* function in the R package *stats* v4.1.0^112^ and a Gower’s dissimilarity matrix constructed with the package *gawdis* v0.1.3^113^. This function is an extension of the classical Gower’s distance that provides a solution to limit unequal traits contribution when different traits are combined in a multi-trait dissimilarity matrix^113^ (functional dendrogram: Supplementary Material 10). The Gower’s distance groups are reported in Table 3.

**Table 3:** The total traits considered in the analyses with an indication of their expected functional meaning and the number of Gower distance groups^113^.

Trait	Trait type	Expected functional meaning	Bibliography	Gower group
Body size	Biological [Continuous]	Body size is tightly linked to temperature. Body size of assemblages of odonates is mainly driven by temperature.	^114,115^	Group 1
Abdomen length	Biological [Continuous]	As for body size.		Group 1
Wings length	Biological [Continuous]	Proxy for dispersal.	^116,117^	Group 1
Habitat	Ecological [Categorical]	Freshwater habitats (lentic/lotic) are among the most threatened ecosystems by climate change.	^118^	Group 2
Flight season time	Ecological [Continuous]	Indirect measure of dispersal potential.	^21^	Group 3
Abdomen pigmentation	Biological [Continuous]	Pigmentation and colour patterns are directly related with thermoregulatory mechanisms. For example, melanism is linked to greater absorption of solar radiation heat in cooler regions.	^114,115,119–121^	Group 4

#### Estimation of the phylogenetic tree

We calculated phylogenetic diversity from a tree calculated with sequences available in GenBank for the analysed species. We retained the five molecular markers (16S rRNA gene; 18S rRNA gene; Cytochrome c oxidase subunit I, COI; Histone H3; NADH dehydrogenase subunit 1, NADH) with the highest taxonomic coverage. We aligned each marker separately using the E-INS-i algorithm implemented in *MAFFT* v7^122^. We translated alignments of protein-coding genes into amino acids and checked them for indels and stop codons. When multiple sequences were available for the same species, we chose the one with the greatest quality and length. Our final alignment included a 1996 base pair for the 16S rRNA gene (number of aligned sequences 87), 1772 base pairs for the 18S rRNA gene (37), 658 base pairs for COI (101), 329 base pairs for H3 (17), and 340 base pairs for NADH (31). We concatenated gene fragments with *SequenceMatrix*^123^ and selected the optimal partition scheme using the Akaike Information Criterion calculated in *PartitionFinder*^124^. We calculated ultrametric phylogenetic trees using *BEAST 2*^125^, setting a relaxed molecular clock model for each partition and a Yule model for the estimation of the topology. Our four Markov Chain Monte Carlo were allowed to run for 100,000,000 generations and sampled every 10,000 generations. The 10% of initial trees were discarded. We used *Tracer* v1.7.1^126^ to confirm the correct mixing of all the parameters and *TreeAnnotator* v2.6.0^125^ to calculate the consensus tree (Supplementary Material 11).

#### Elaboration of the taxonomic, functional, and phylogenetic diversity maps

We assembled taxonomic, functional, and phylogenetic diversity maps using modified versions of the functions *alpha* (temporalAlpha) and *beta* (temporalBeta) from the package *BAT* v2.7.1^127^. First, we stacked the binary maps obtained from the best-performing SDMs models of each species. Then, we calculate *α* diversity across the three biodiversity facets for present and future stacked maps. We quantified variations in *α* diversity between present and future scenarios by subtracting the *α* diversity values in the future and the present. We calculated *β* diversity in the same way, estimating replacement and richness components of *β* diversity^18^ for each cell comparing future and present communities. To calculate the *α*/*β* functional and phylogenetic diversity, we used the functional or phylogenetic tree as an additional parameter into the functions.

### Testing for phylogenetic signal and trait influence on species response to climate change

We used phylogenetic comparative methods to examine the influence of phylogeny and traits on the responses of Odonata to climate change. We characterised the response to climate change of odonates using three response variables: i) the proportional variation in habitat suitability, calculated as the ratio between future and current predicted area (Relative area change); ii) the altitudinal shift in the distribution, estimated as the difference between future and current mean altitude (Altitude difference); and iii) the centroid shift in the distribution, measured as the linear distance between the position of future and current centroid (Centroid difference). We used the function *distGeo* from the R package *geosphere* v1.5-14^128^ to estimate the centroid position.

We investigated whether closely related species experience similar responses to climate change using Pagel’s λ and Blomberg’s K, as implemented in the function *phylosig* from the R package *phytools* v0.7-80^129^. Values close to 0 indicate a weak phylogenetic signal, whereas values close to 1 or higher suggest the presence of phylogenetic signal. We then visualised the phylogenetic signal of each trait using maximum likelihood ancestral character reconstruction. This technique is widely used in phylogenetics, and it is useful to reconstruct the transformation series of a characters given a tree topology and branch length^130^. To create the ancestral character reconstruction trees, we used the function *contMap*of the R package *phytools*.

Finally, we explored the relationship between traits and the species’ response to climate change using PGLS. This approach is suitable to investigate the effect of several explanatory variables on a single response variable while controlling for the non-independence of residuals due to the phylogenetic history shared across species. To run this analysis, we used the function *pgls* from the package *caper* v1.0.1^131^. We constructed three separate models, one for each of the response variables (Altitude difference, Centroid difference, Relative area change) and three traits (Body length, Flight Season, Habitat). We excluded other traits from the models due to multicollinearity. We used three functions of branch transformation (lambda, kappa, and delta) to adjust the covariance matrix to the data selecting the best transformation through a maximum likelihood procedure. Prior to model fitting, we performed data exploration, visually inspecting for the presence of outliers in the predictor and response variables with dotcharts and verifying multicollinearity among predictor variables^86^.

We calculated phylogenetic signal, ancestral character reconstruction, and PGLS for the entire order of Odonata first, and afterward we repeated all analyses for the suborders Anisoptera and Zygoptera separately, since it has been demonstrated that they can respond differently to environmental change^132^.

### Supplementary information

Supplementary material 1

Supplementary material 2

Supplementary material 3

Supplementary material 4

Supplementary material 5

Supplementary material 6

Supplementary material 7

Supplementary material 8

Supplementary material 9

Supplementary material 10

Supplementary material 11

## Data Availability

We stored all data, raw predictor variables and detailed model outputs in the OSF repository (https://osf.io/4rjuc/).

## Code availability

All code used to perform analyses and produce plots is available in GitHub (https://github.com/TommasoCanc/Odonata_SDM_2022).
