# Supplementary material for: Climate change will redefine taxonomic, functional, and phylogenetic diversity of Odonata in space and time
Source: NPJ Biodivers. 2022 Nov 17;1:1. doi: 10.1038/s44185-022-00001-3 (PMC11290607; doi:10.1038/s44185-022-00001-3)
Supplement: Supplementary file 1 — Supplementary material 1 [file 44185_2022_1_MOESM1_ESM.pdf]

Supplementary Material 1

Multicollinearity dendrogram

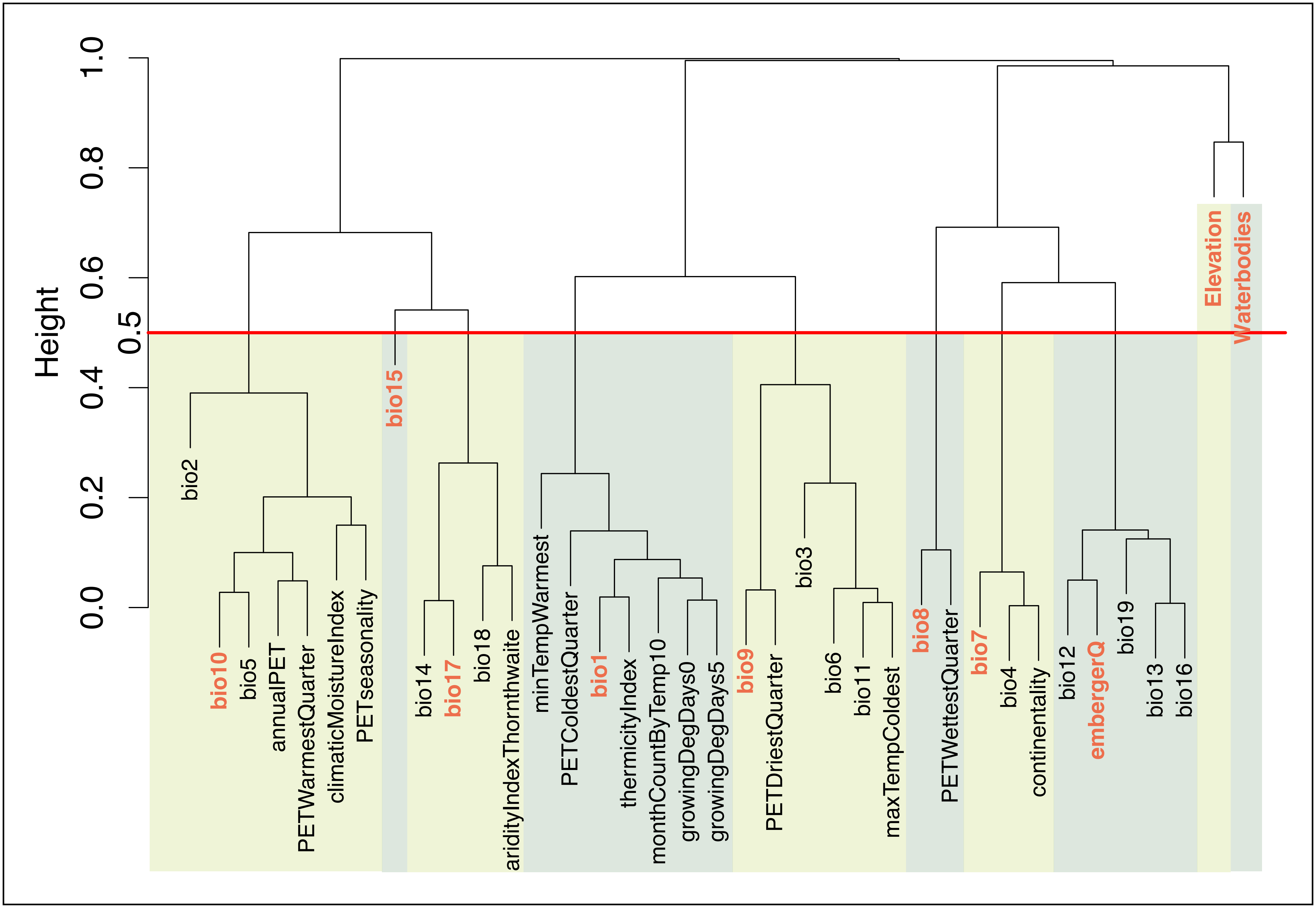

Variance Inflation Factor

Elevation	1.59
embergerQ	2.48
Waterbodies	1.07

bio7	2.52
bio8	1.80
bio10	1.41
bio15	1.46
