# Supplementary material for: Climate change will redefine taxonomic, functional, and phylogenetic diversity of Odonata in space and time
Source: NPJ Biodivers. 2022 Nov 17;1:1. doi: 10.1038/s44185-022-00001-3 (PMC11290607; doi:10.1038/s44185-022-00001-3)
Supplement: Supplementary file 3 — Supplementary material 3 [file 44185_2022_1_MOESM3_ESM.docx]

**Supplementary Material 3.**

**Table of Content**

- Brief description of Representative Concentration Pathway and Climate system models
- Hexabin map depicting the number of GBIF occurrences and their spatial arrangement.
- Species distribution model projections species by species

**Representative Concentration Pathway (RCP) and Climate system model description.**

RCP 4.5 is an intermediate greenhouse gas concentration projection scenario proposed by the Intergovernmental Panel on Climate Change. This scenario expects that greenhouse gas will increase until 2040, whit a subsequent decline. According to the model, the emissions of carbon dioxide (CO_2_), methane (CH_4_), and sulphur dioxide (SO_2_) will start to decline approximately after 2040. Based on RCP 4.5, it is forecasted that the global air temperature will increase by 2–3 ˚C, and the mean sea level will rise 35% higher than that of RCP 2.6.

BCC_CSM1 is a Climate system model developed at the Beijing Climate Center (BCC). BCC_CSM1 comprises four separate components (atmospheric: BCC_AGCM2.0.1, land-surface: CLM3, oceanic: POP, and sea-ice: CSIM4) joined by the central coupler CPL5.

We refer the reader to the official web site for further details (http://forecast.bcccsm.ncc-cma.net/web/channel-11.htm)

MIROC-ESM-CHEM is a Climate system model developed cooperatively by the University of Tokyo and JAMSTEC (Watanabe et al., 2011). MIROC-ESM-CHEM include as main components the atmospheric general circulation model (MIROC-AGCM), the oceanic sea-ice component (COCO), the land surface model (MATSIRO), and the atmospheric chemistry.

NorESM1-M is a Climate system model developed by the University Corporation for Atmospheric Research (Bentsen et al., 2013). NorESM1-M include as main components the atmospheric general circulation model (CAM4-Oslo), the oceanic (use at isopycnic surfaces) sea-icecomponent (CICE4), the land surface model (CLM4).

**Supplementary literature**

Watanabe, S. et al., 2011. MIROC-ESM 2010: model description and basic results of CMIP5-20c3m experiments”, Geosci. Model Dev., 4, 845–872.

Bentsen, M. et al., 2013. The Norwegian Earth System Model, NorESM1-M – Part 1: Description and basic evaluation of the physical climate, Geosci. Model Dev., 6, 687–720.

**Hexabin map depicting the number of GBIF occurrences and their spatial arrangement.**


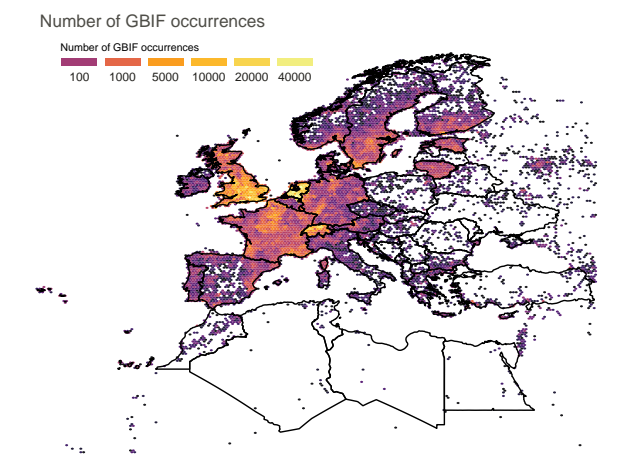


**Main scheme of the following pictures:**

- **Top-left map**: Best model prediction map for the current condition.

- **Top-right maps**: Habitat availability gain (light blue), loss (red) maps and centroid shift future (yellow point) vs present (orange point); time period 2050 (up) and 2070 (down).

- **Bottom-left table**: Lon: Longitude centroid; Lat: Latitude centroid; period: Time period; avgAltitude: average altitude of the predicted binary map; cellNumber: Number of cells of the predicted binary map; cellArea: total cell area of the predicted binary map; cellDifArea: Difference of area between future and present prediction; direction: direction of centroid shift; dist_km: Shift of centroid comparing future and present prediction.

- **Bottom-right plot**: Violin plot shows the elevation shift across time periods.

[**Anisoptera**](#_Toc82005084)

[**Family: Aeshnidae**](#_Toc82005085)

[*Aeshna affinis* Vander Linden, 1820](#_Toc82005086)

[*Aeshna caerulea* (Ström, 1783)](#_Toc82005087)

[*Aeshna crenata* Hagen, 1856](#_Toc82005088)

[*Aeshna cyanea* (Müller, 1764)](#_Toc82005089)

[*Aeshna grandis* (Linnaeus, 1758)](#_Toc82005090)

[*Aeshna isosceles* (Müller, 1767)](#_Toc82005091)

[*Aeshna juncea* (Linnaeus, 1758)](#_Toc82005092)

[*Aeshna mixta* Latreille, 1805](#_Toc82005093)

[*Aeshna serrata* Hagen, 1856](#_Toc82005094)

[*Aeshna subarctica* Walker, 1908](#_Toc82005095)

[*Aeshna viridis* Eversmann, 1836](#_Toc82005096)

[*Anax ephippiger* (Burmeister, 1839)](#_Toc82005097)

[*Anax imperator* Leach, 1815](#_Toc82005098)

[*Anax parthenope* (Selys, 1839)](#_Toc82005099)

[*Boyeria irene* (Fonscolombe, 1838)](#_Toc82005100)

[*Brachytron pratense* (Müller, 1764)](#_Toc82005101)

[*Caliaeschna microstigma* (Schneider, 1845)](#_Toc82005102)

[**Family: Gomphidae**](#_Toc82005103)

[*Gomphus graslinii* Rambur, 1842](#_Toc82005104)

[*Gomphus pulchellus* Selys, 1840](#_Toc82005105)

[*Gomphus simillimus* Selys, 1840](#_Toc82005106)

[*Gomphus vulgatissimus* (Linnaeus, 1758)](#_Toc82005107)

[*Lindenia tetraphylla* (Vander Linden, 1825)](#_Toc82005108)

[*Onychogomphus forcipatus* (Linnaeus, 1758)](#_Toc82005109)

[*Onychogomphus uncatus* (Charpentier, 1840)](#_Toc82005110)

[*Ophiogomphus cecilia* (Geoffroy in Fourcroy, 1785)](#_Toc82005111)

[*Stylurus flavipes* (Charpentier, 1825)](#_Toc82005112)

[**Family: Cordulegastridae**](#_Toc82005113)

[*Cordulegaster bidentata* Selys, 1843](#_Toc82005114)

[*Cordulegaster boltonii* (Donovan, 1807)](#_Toc82005115)

[**Family: Macromiidae**](#_Toc82005116)

[*Macromia splendens* (Pictet, 1843)](#_Toc82005117)

[**Family: Corduliidae**](#_Toc82005118)

[*Cordulia aenea* (Linnaeus, 1758)](#_Toc82005119)

[*Epitheca bimaculate* (Charpentier, 1825)](#_Toc82005120)

[*Somatochlora alpestris* (Selys, 1840)](#_Toc82005121)

[*Somatochlora arctica* (Zetterstedt, 1840)](#_Toc82005122)

[*Somatochlora flavomaculata* (Vander Linden, 1825)](#_Toc82005123)

[*Somatochlora meridionalis* Nielsen, 1935](#_Toc82005124)

[*Somatochlora metallica* (Vander Linden, 1825)](#_Toc82005125)

[**Family: Libellulidae**](#_Toc82005126)

[*Brachythemis impartita* (Karsch, 1890)](#_Toc82005127)

[*Crocothemis erythraea* (Brullé, 1832)](#_Toc82005128)

[*Diplacodes lefebvrii* Rambur, 1842](#_Toc82005129)

[*Leucorrhinia albifrons* (Burmeister, 1839)](#_Toc82005130)

[*Leucorrhinia caudalis* (Charpentier, 1840)](#_Toc82005131)

[*Leucorrhinia dubia* (Vander Linden, 1825)](#_Toc82005132)

[*Leucorrhinia pectoralis* (Charpentier, 1825)](#_Toc82005133)

[*Leucorrhinia rubicunda* (Linnaeus, 1758)](#_Toc82005134)

[*Libellula depressa* Linnaeus, 1758](#_Toc82005135)

[*Libellula fulva* Müller, 1764](#_Toc82005136)

[*Libellula quadrimaculata* Linnaeus, 1758](#_Toc82005137)

[*Orthetrum albistylum* (Selys, 1848)](#_Toc82005138)

[*Orthetrum brunneum* (Fonscolombe, 1837)](#_Toc82005139)

[*Orthetrum cancellatum* (Linnaeus, 1758)](#_Toc82005140)

[*Orthetrum chrysostigma* (Burmeister, 1839)](#_Toc82005141)

[*Orthetrum coerulescens* (Fabricius, 1798)](#_Toc82005142)

[*Orthetrum taeniolatum* (Schneider, 1845)](#_Toc82005143)

[*Orthetrum trinacria* (Selys, 1841)](#_Toc82005144)

[*Selysiothemis nigra* (Vander Linden, 1825)](#_Toc82005145)

[*Sympetrum danae* (Sulzer, 1776)](#_Toc82005146)

[*Sympetrum depressiusculum* (Selys, 1841)](#_Toc82005147)

[*Sympetrum flaveolum* (Linnaeus, 1758)](#_Toc82005148)

[*Sympetrum fonscolombii* (Selys, 1840)](#_Toc82005149)

[*Sympetrum meridionale* (Selys, 1841)](#_Toc82005150)

[*Sympetrum pedemontanum* (Müller in Allioni, 1766)](#_Toc82005151)

[*Sympetrum sanguineum* (Müller, 1764)](#_Toc82005152)

[*Sympetrum sinaiticum* Dumont, 1977](#_Toc82005153)

[*Sympetrum striolatum* (Charpentier, 1840)](#_Toc82005154)

[*Sympetrum vulgatum* (Linnaeus, 1758)](#_Toc82005155)

[*Trithemis annulata* (Palisot de Beauvois, 1807)](#_Toc82005156)

[*Trithemis arteriosa* (Burmeister, 1839)](#_Toc82005157)

[*Trithemis kirbyi* Selys, 1891](#_Toc82005158)

[Zygoptera](#_Toc82005159)

[**Family: Calopterygidae**](#_Toc82005160)

[*Calopteryx haemorrhoidalis* (Vander Linden, 1825)](#_Toc82005161)

[*Calopteryx splendens* (Harris, 1780)](#_Toc82005162)

[*Calopteryx virgo* (Linnaeus, 1758)](#_Toc82005163)

[*Calopteryx xanthostoma* (Charpentier, 1825)](#_Toc82005164)

[**Family: Coenagrionidae**](#_Toc82005165)

[*Ceriagrion tenellum* (De Villers, 1789)](#_Toc82005166)

[*Coenagrion armatum* (Charpentier, 1840)](#_Toc82005167)

[*Coenagrion caerulescens* (Fonscolombe, 1838)](#_Toc82005168)

[*Coenagrion hastulatum* (Charpentier, 1825)](#_Toc82005169)

[*Coenagrion johanssoni* Wallengren, 1894](#_Toc82005170)

[*Coenagrion lunulatum* (Charpentier, 1840)](#_Toc82005171)

[*Coenagrion mercuriale* (Charpentier, 1840)](#_Toc82005172)

[*Coenagrion ornatum* (Selys, 1850)](#_Toc82005173)

[*Coenagrion puella* (Linnaeus, 1758)](#_Toc82005174)

[*Coenagrion pulchellum* (Vander Linden, 1825)](#_Toc82005175)

[*Coenagrion scitulum* (Rambur, 1842)](#_Toc82005176)

[*Enallagma cyathigerum* (Charpentier, 1840)](#_Toc82005177)

[*Erythromma lindenii* (Selys, 1840)](#_Toc82005178)

[*Erythromma najas* (Hansemann, 1823)](#_Toc82005179)

[*Erythromma viridulum* (Charpentier, 1840)](#_Toc82005180)

[*Ischnura elegans* (Vander Linden, 1820)](#_Toc82005181)

[*Ischnura genei* (Rambur, 1842)](#_Toc82005182)

[*Ischnura graellsii* (Rambur, 1842)](#_Toc82005183)

[*Ischnura pumilio* (Charpentier, 1825)](#_Toc82005184)

[*Nehalennia speciosa* (Charpentier, 1840)](#_Toc82005185)

[*Pyrrhosoma nymphula* (Sulzer, 1776)](#_Toc82005186)

[**Family: Euphaeidae**](#_Toc82005187)

[*Epallage fatime* (Charpentier, 1840)](#_Toc82005188)

[**Family: Lestidae**](#_Toc82005189)

[*Chalcolestes parvidens* Artobolevsky, 1929](#_Toc82005190)

[*Chalcolestes viridis* (Vander Linden, 1825)](#_Toc82005191)

[*Lestes barbarous* (Fabricius, 1798)](#_Toc82005192)

[*Lestes dryas* Kirby, 1890](#_Toc82005193)

[*Lestes macrostigma* (Eversmann, 1836)](#_Toc82005194)

[*Lestes sponsa* (Hansemann, 1823)](#_Toc82005195)

[*Lestes virens* (Charpentier, 1825)](#_Toc82005196)

[*Sympecma fusca* (Vander Linden, 1820)](#_Toc82005197)

[*Sympecma paedisca* (Brauer, 1877)](#_Toc82005198)

[**Family: Platycnemididae**](#_Toc82005199)

[*Platycnemis acutipennis* Selys, 1841](#_Toc82005200)

[*Platycnemis latipes* Rambur, 1842](#_Toc82005201)

[*Platycnemis pennipes* (Pallas, 1771)](#_Toc82005202)

[**Family: *Incertae sedis***](#_Toc82005203)

[*Oxygastra curtisii* (Dale, 1834)](#_Toc82005204)

# **Anisoptera**

### **Family: Aeshnidae**

### *Aeshna affinis* Vander Linden, 1820


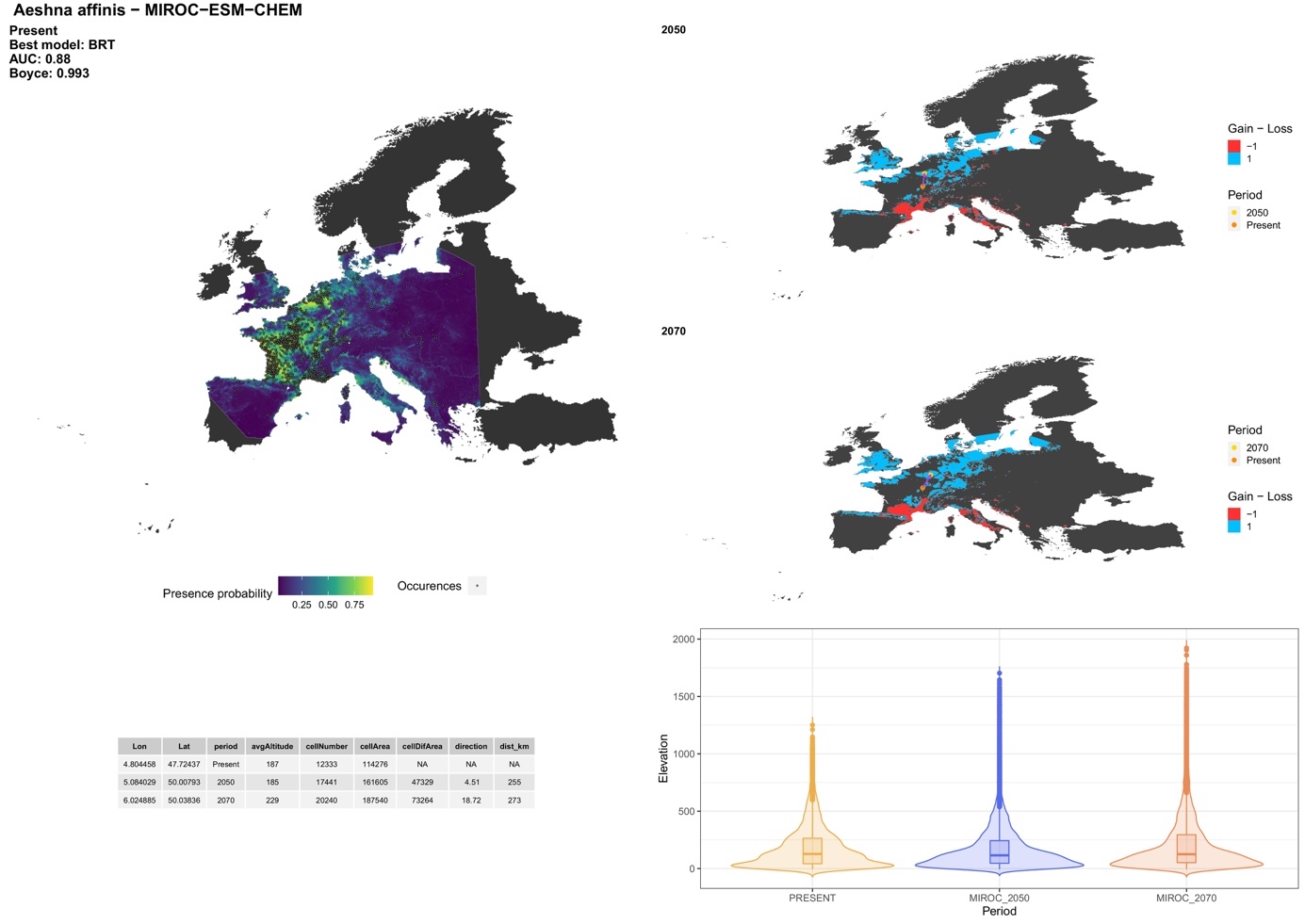


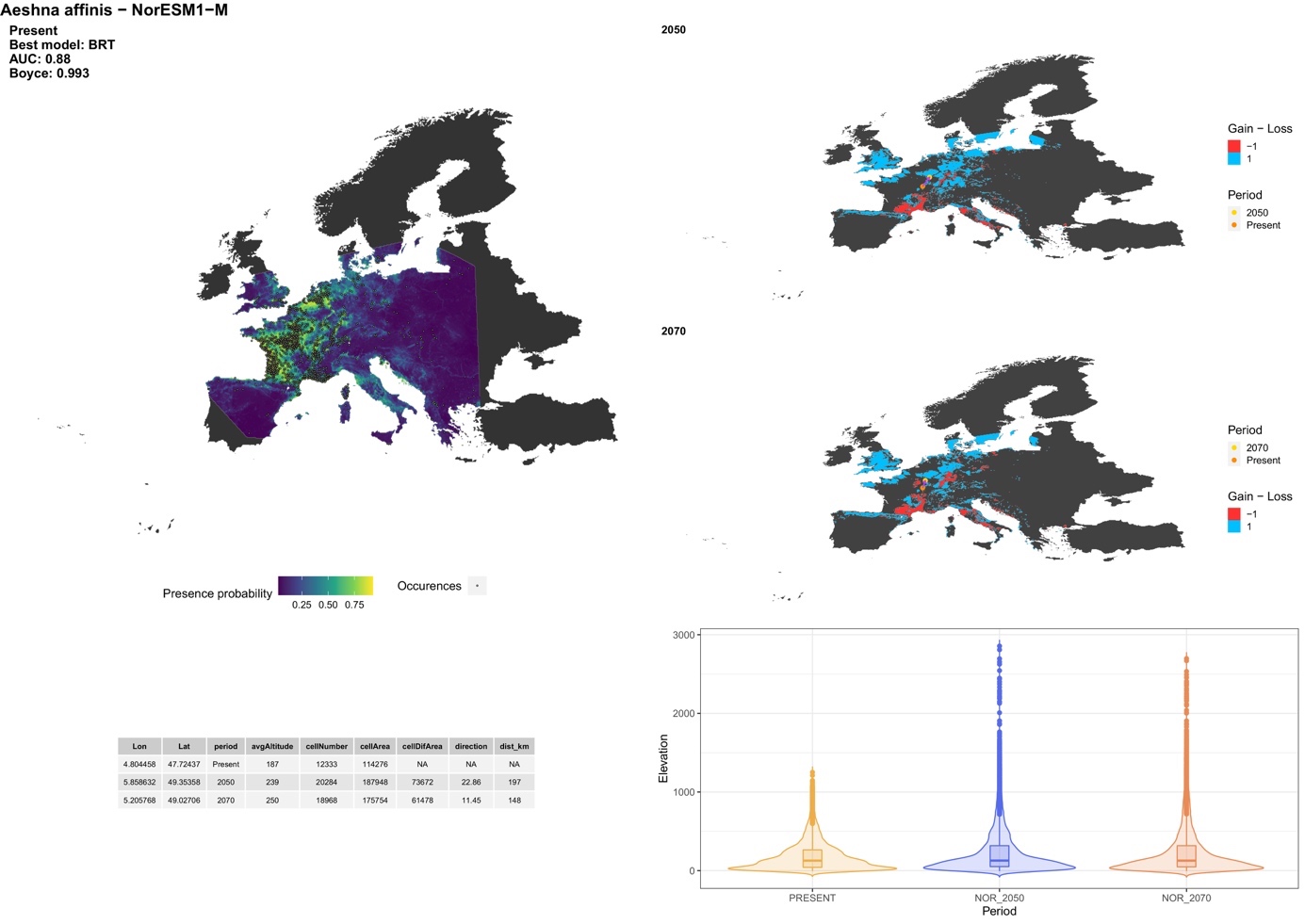

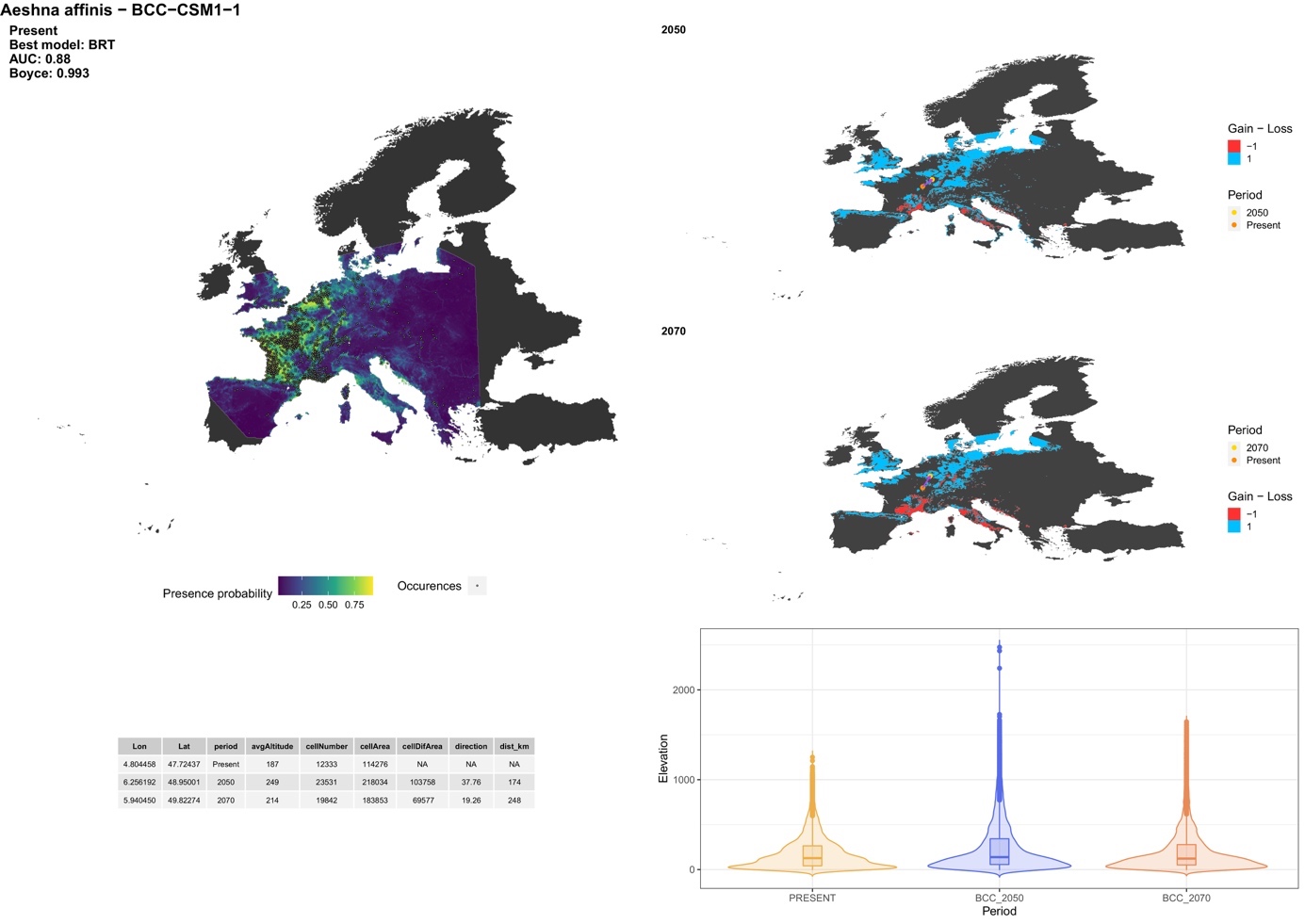


### *Aeshna caerulea* (Ström, 1783)


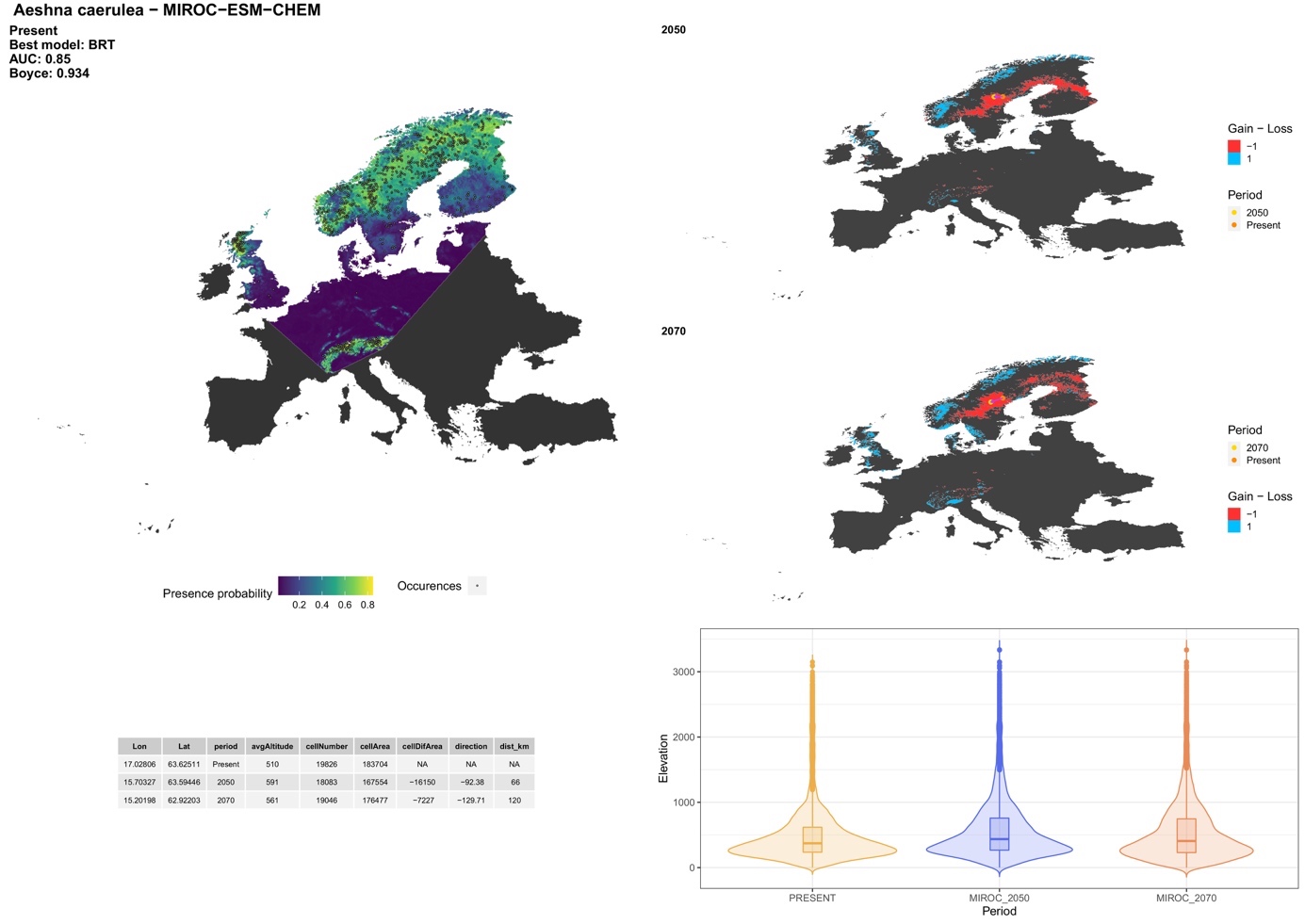


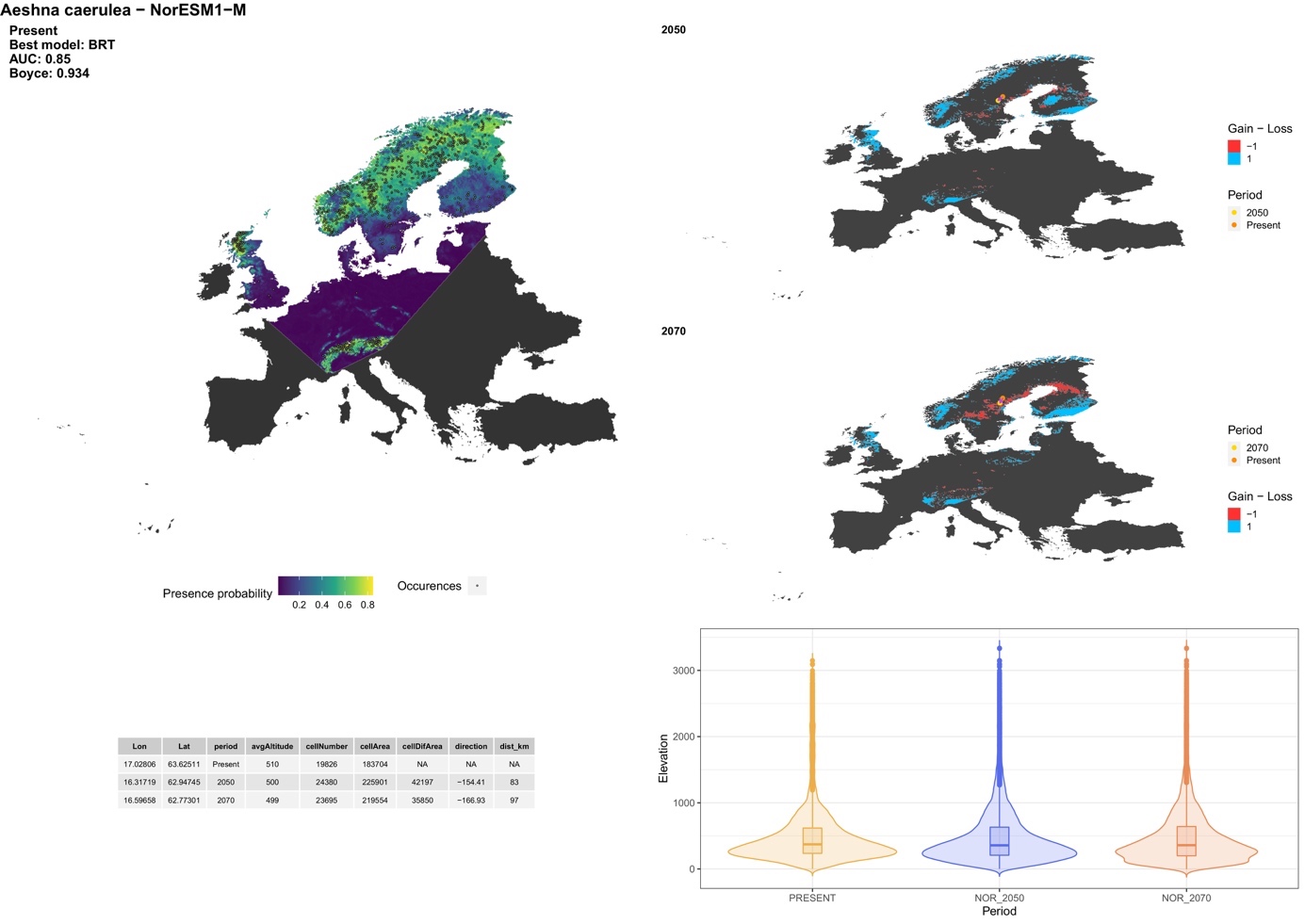


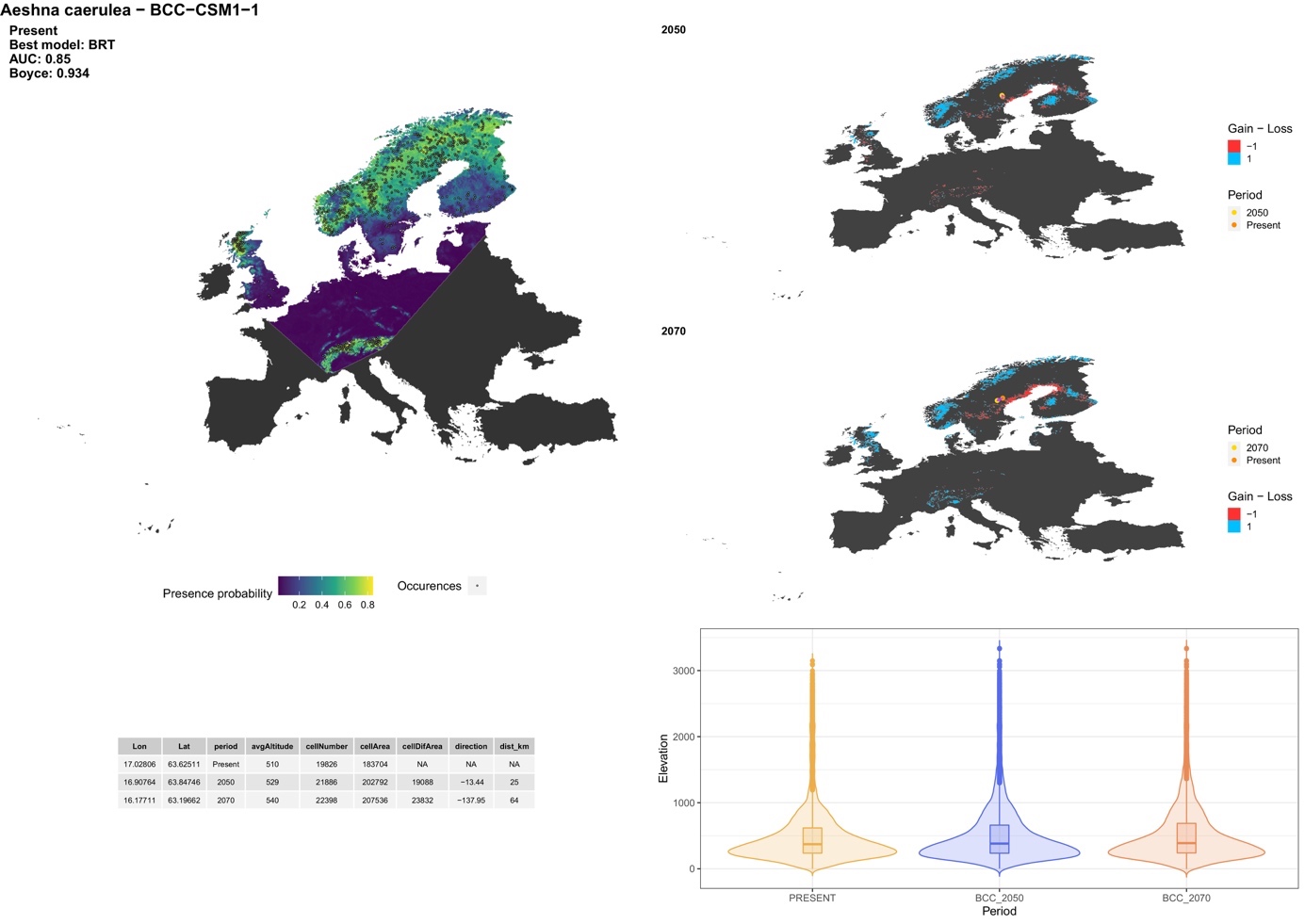


### *Aeshna crenata* Hagen, 1856


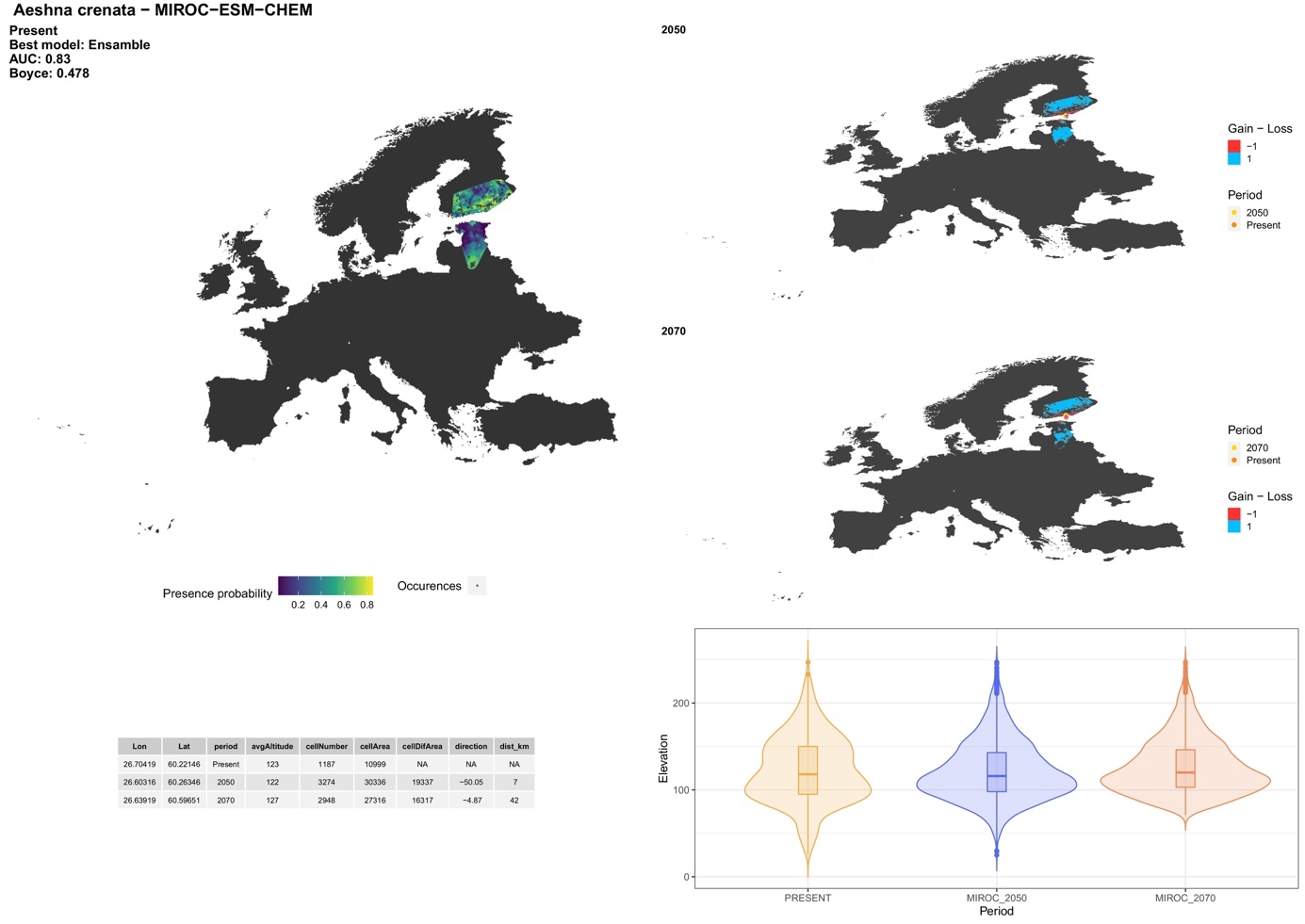


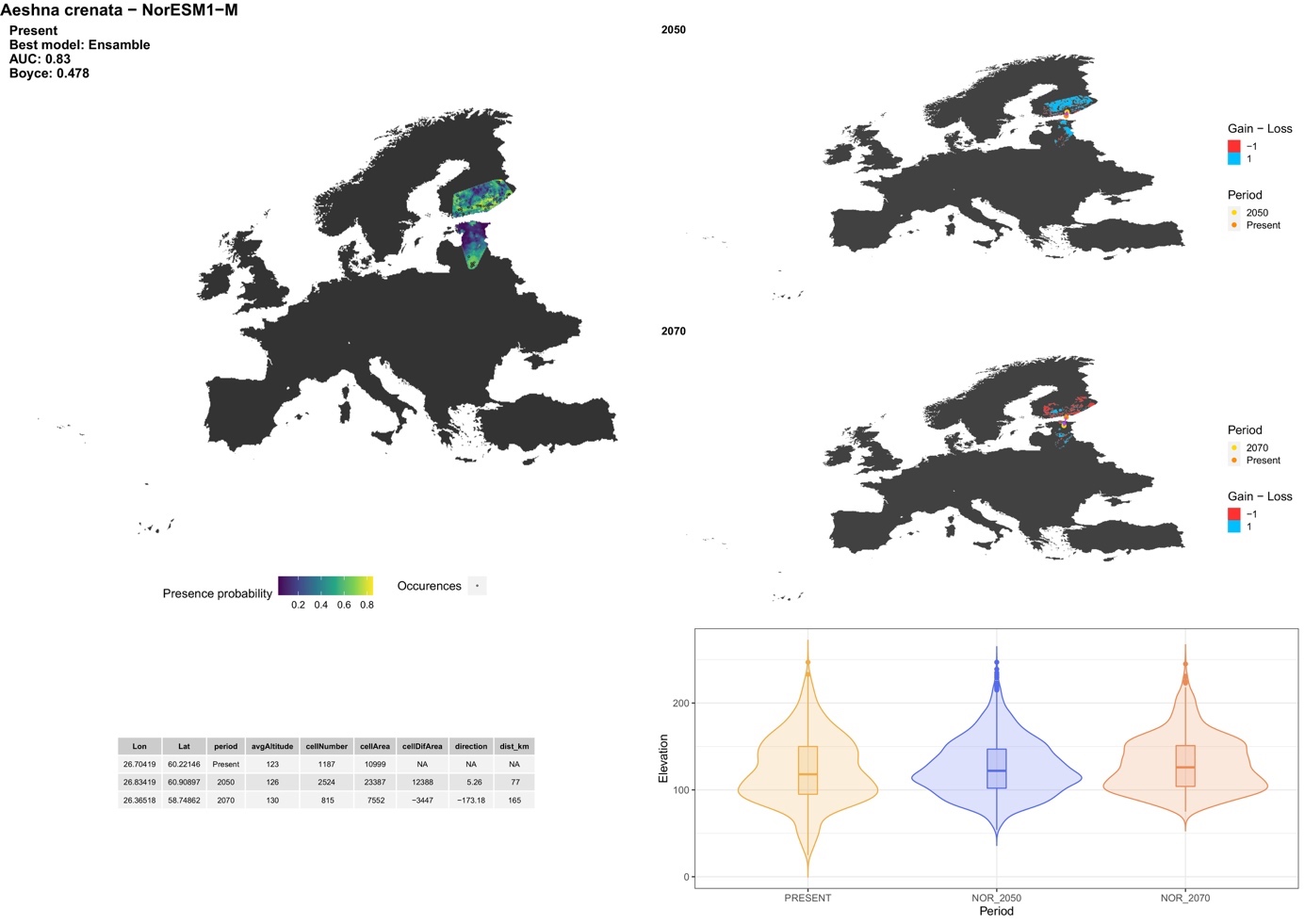


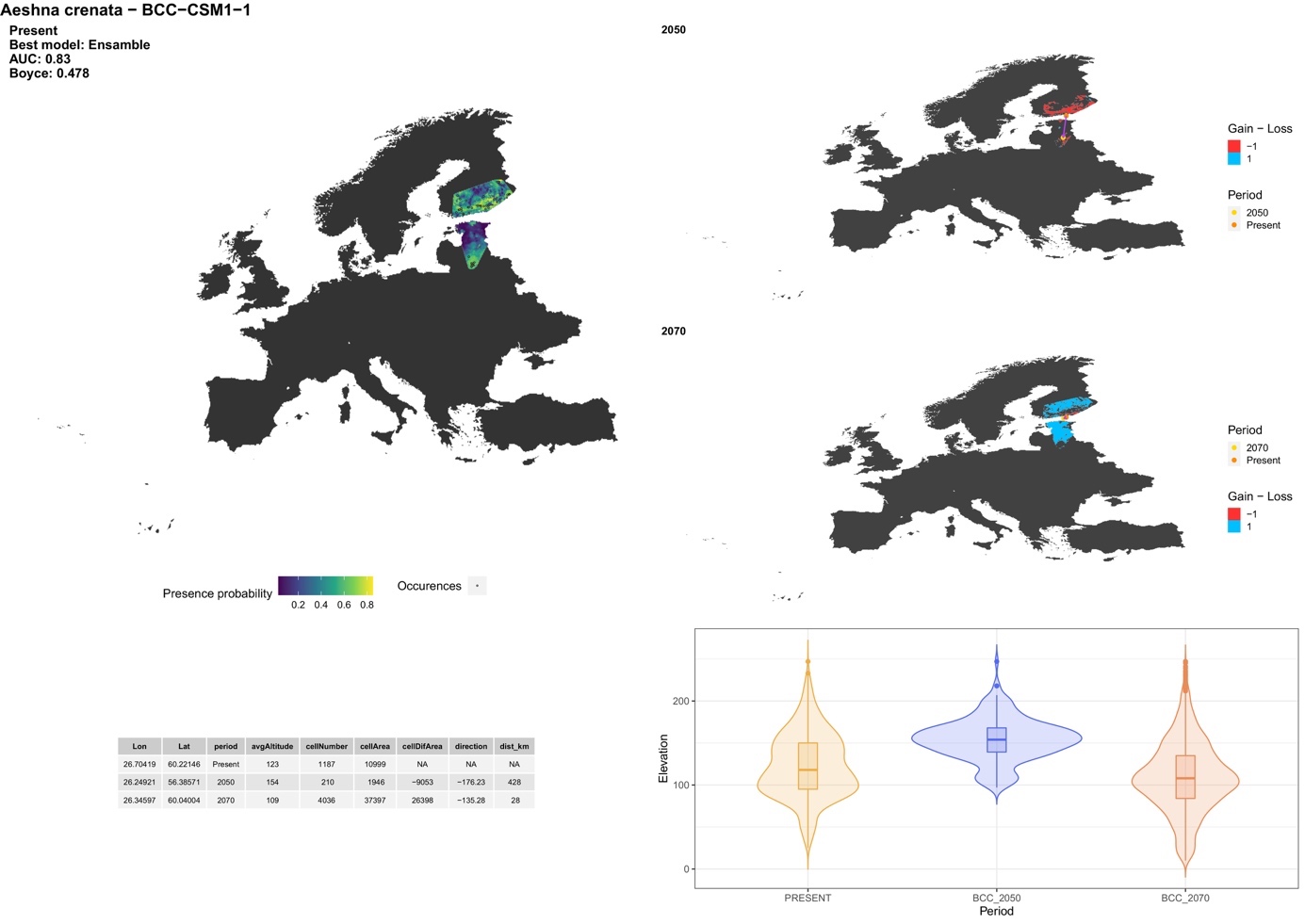


### *Aeshna cyanea* (Müller, 1764)


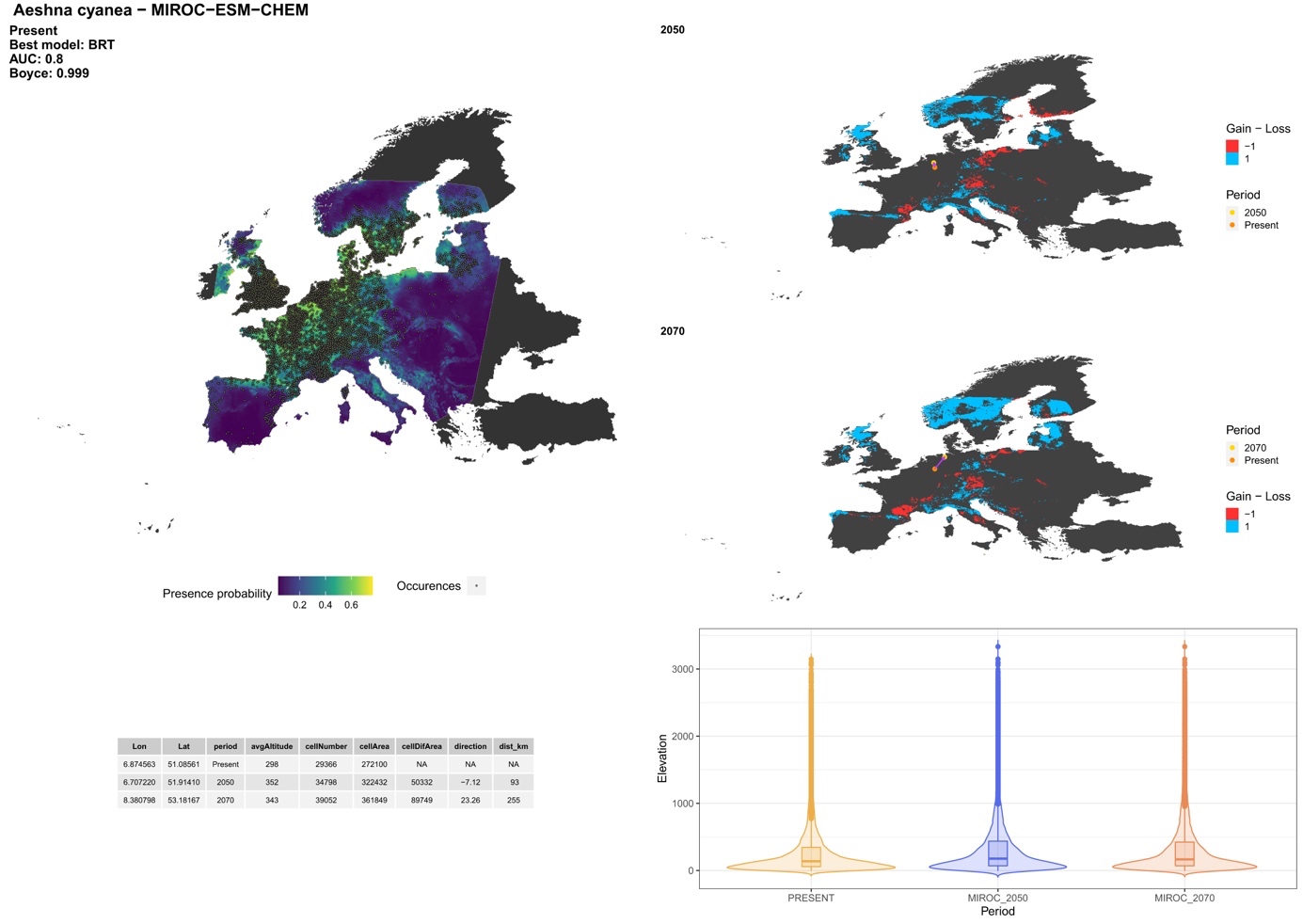


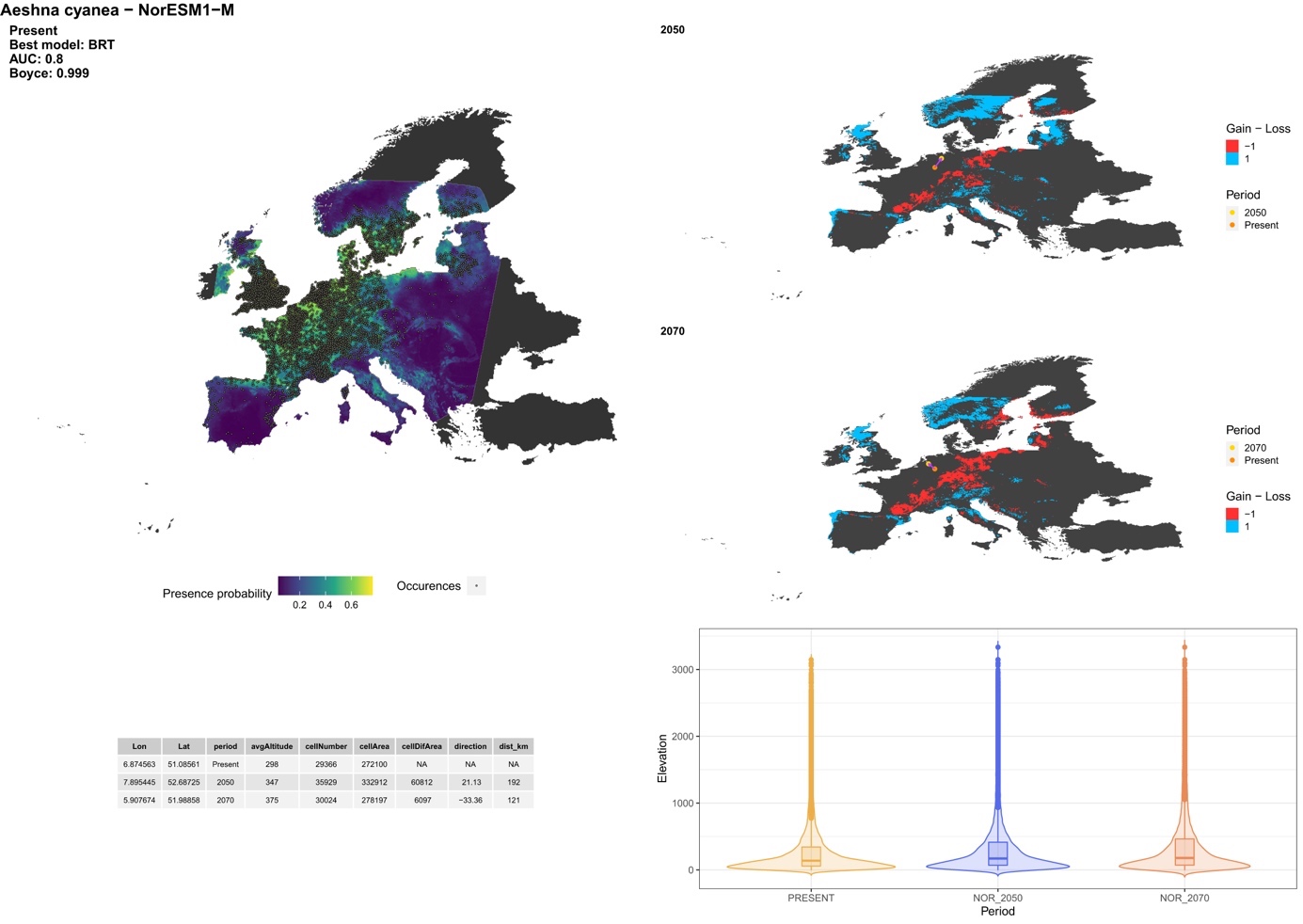


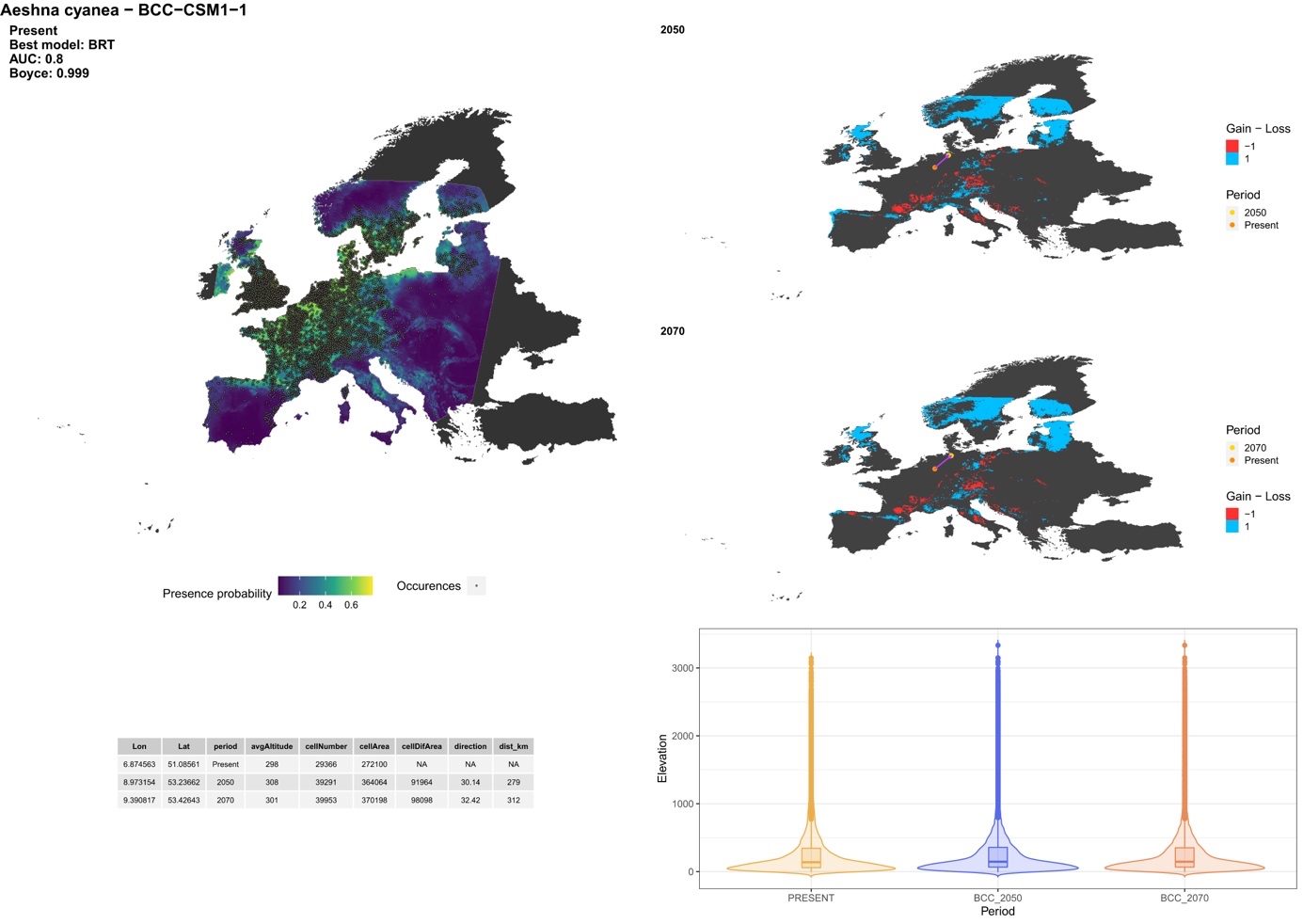


### *Aeshna grandis* (Linnaeus, 1758)


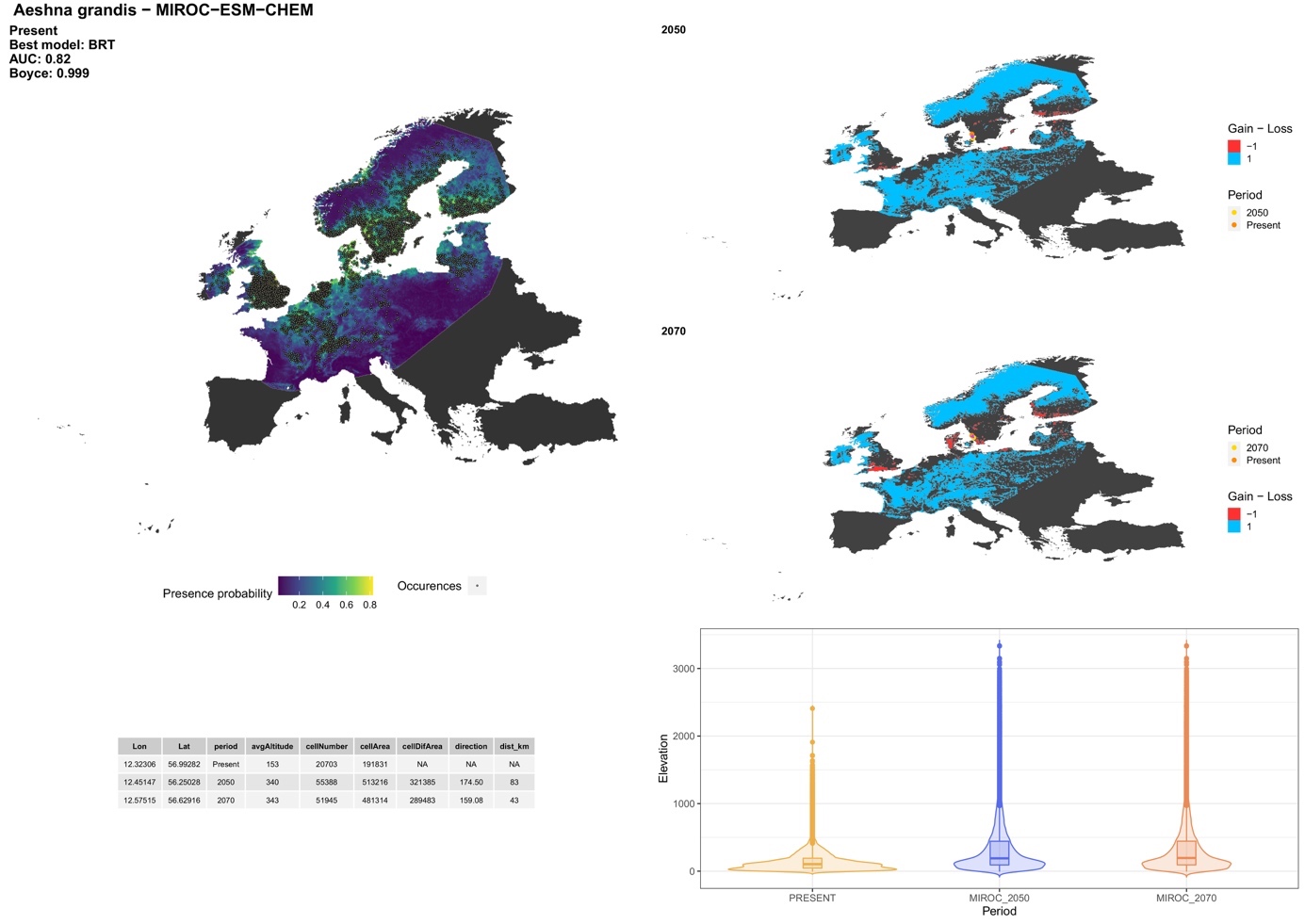


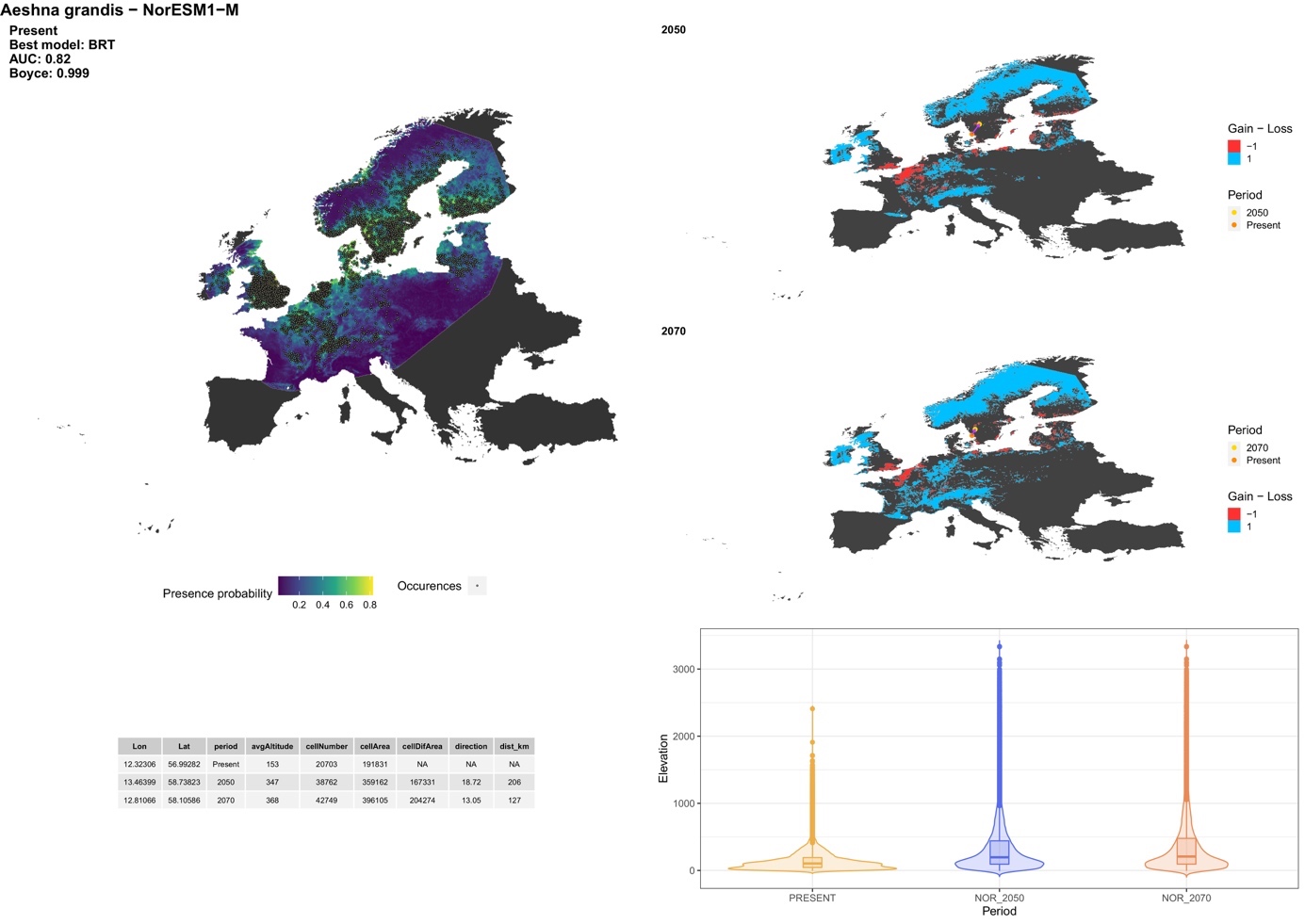


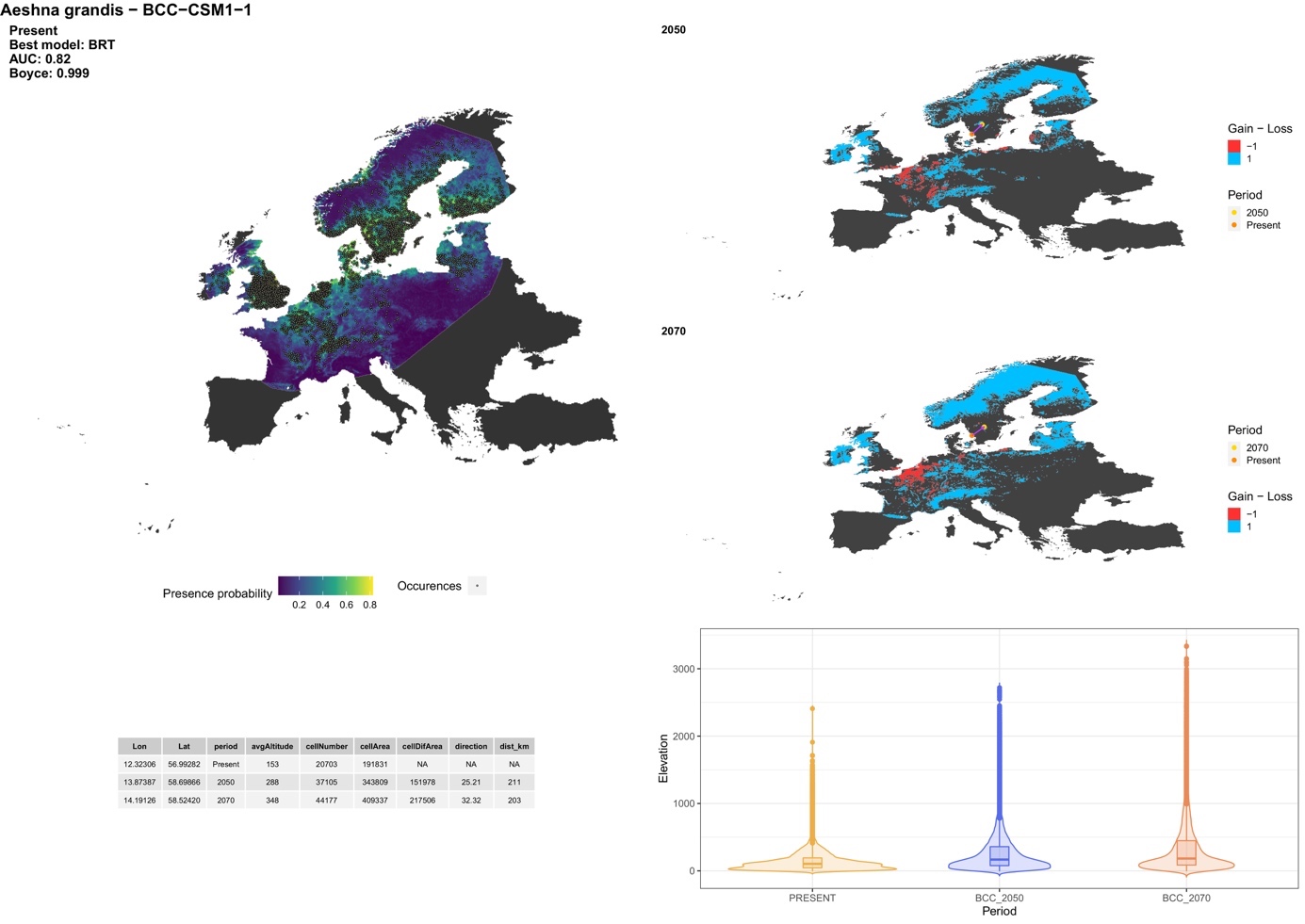


### *Aeshna isosceles* (Müller, 1767)


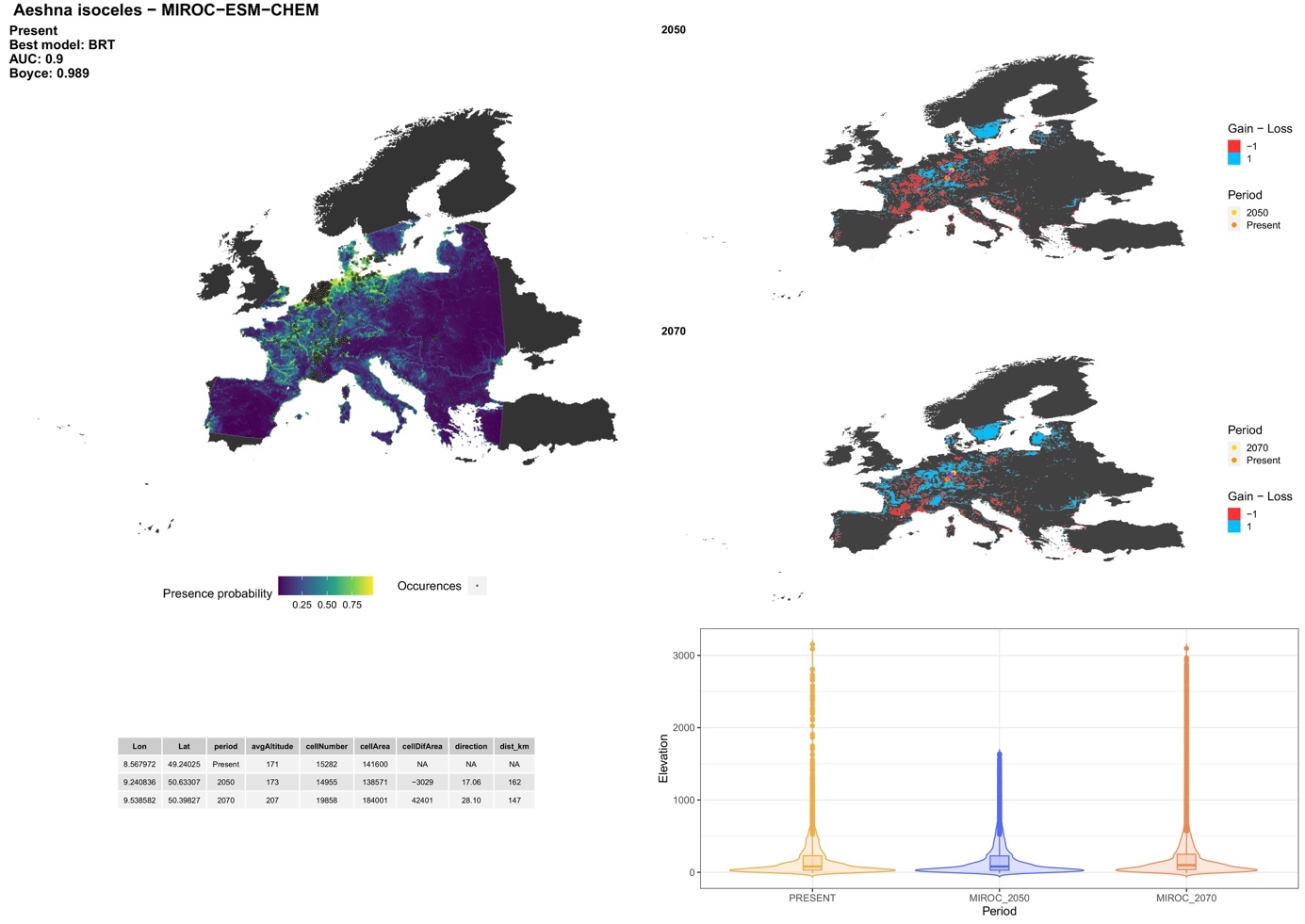


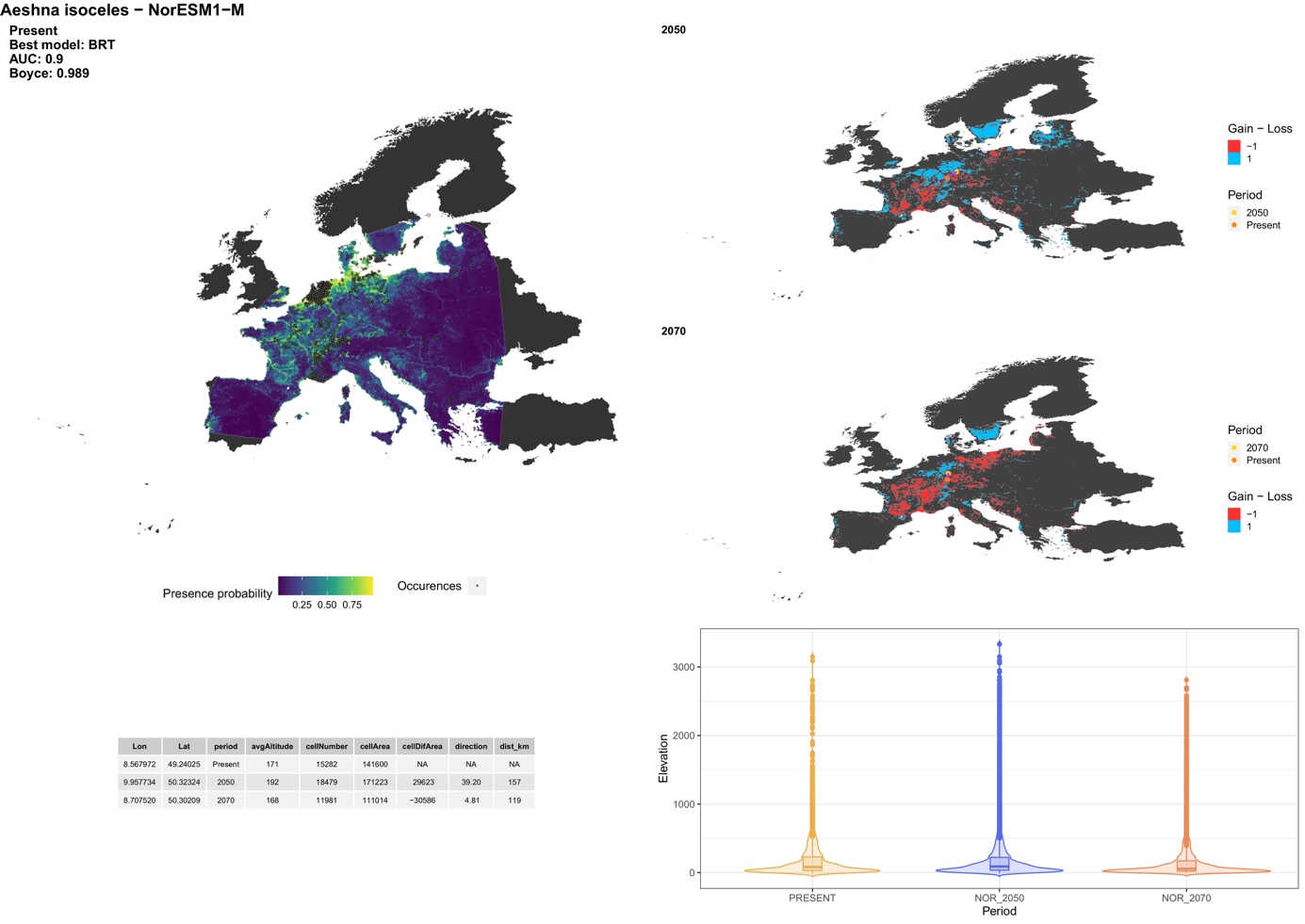


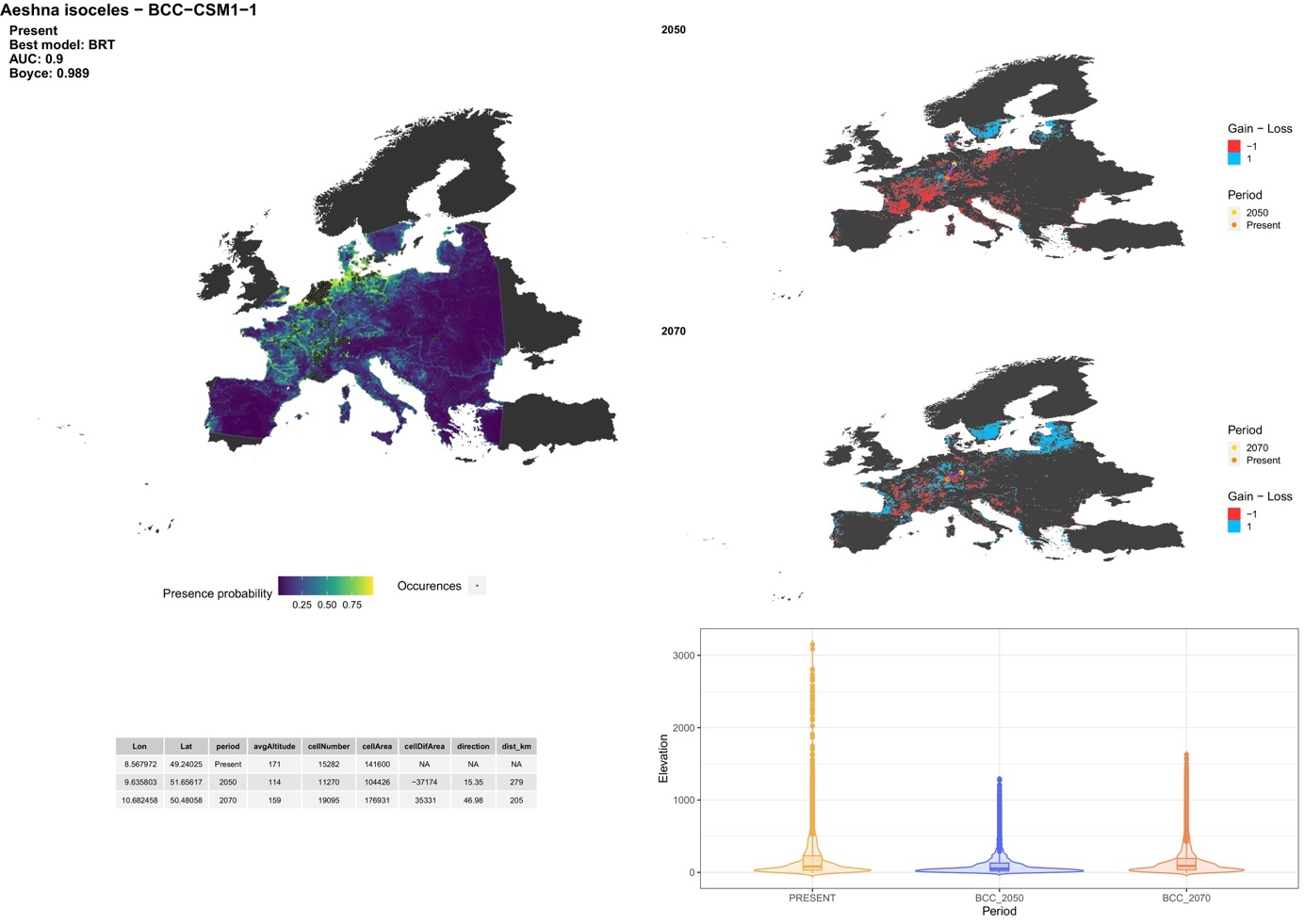


### *Aeshna juncea* (Linnaeus, 1758)


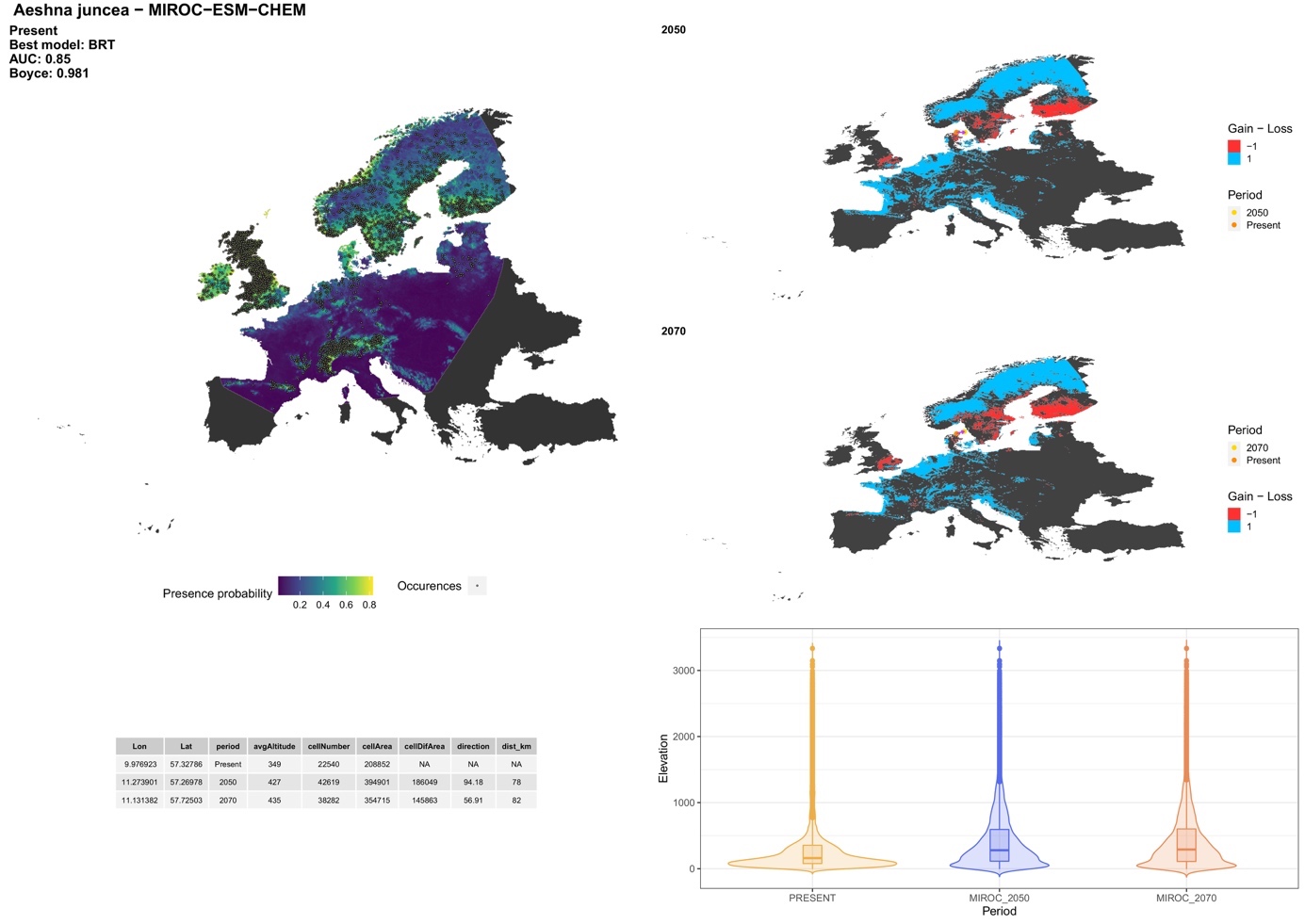


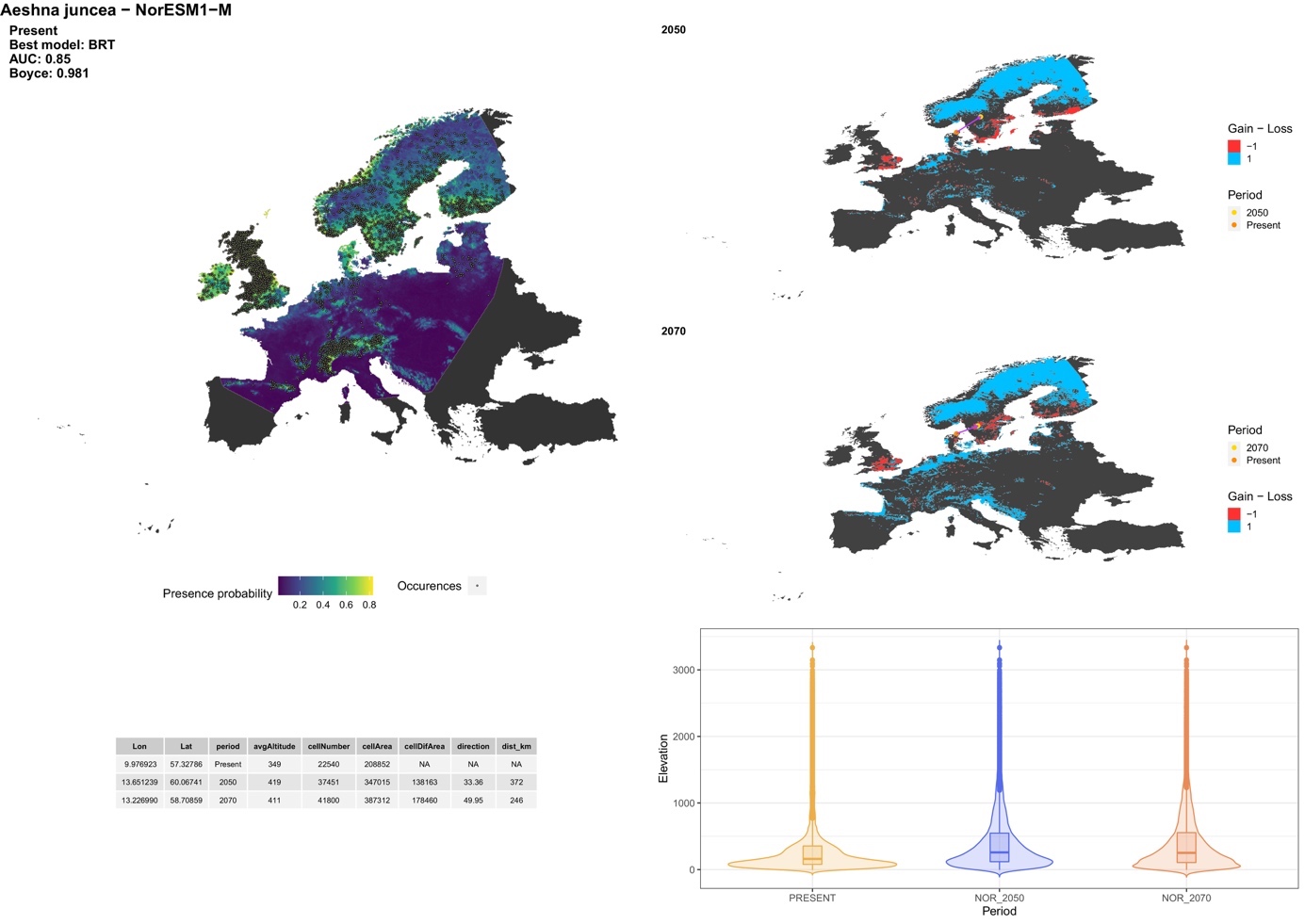


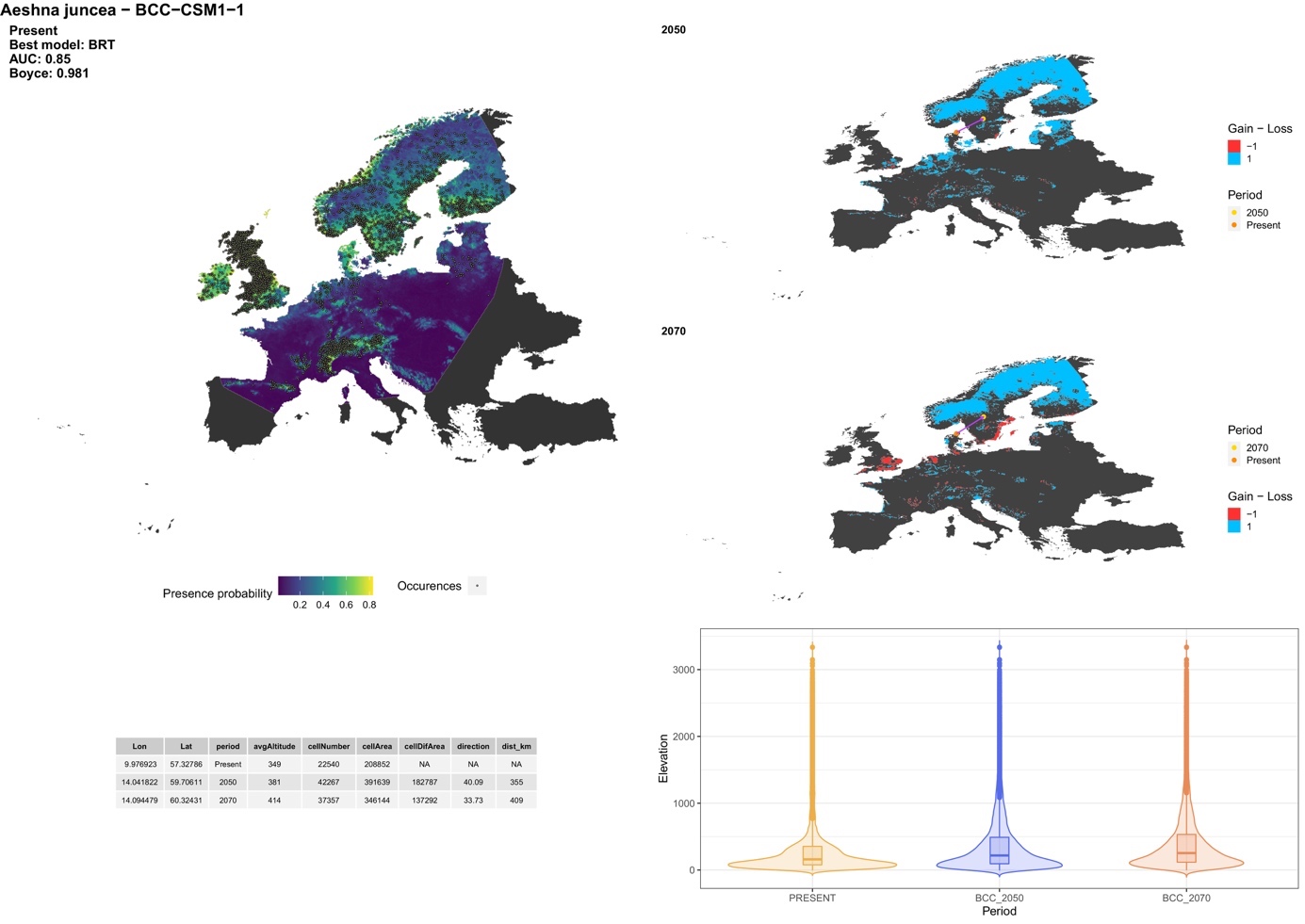


### *Aeshna mixta* Latreille, 1805


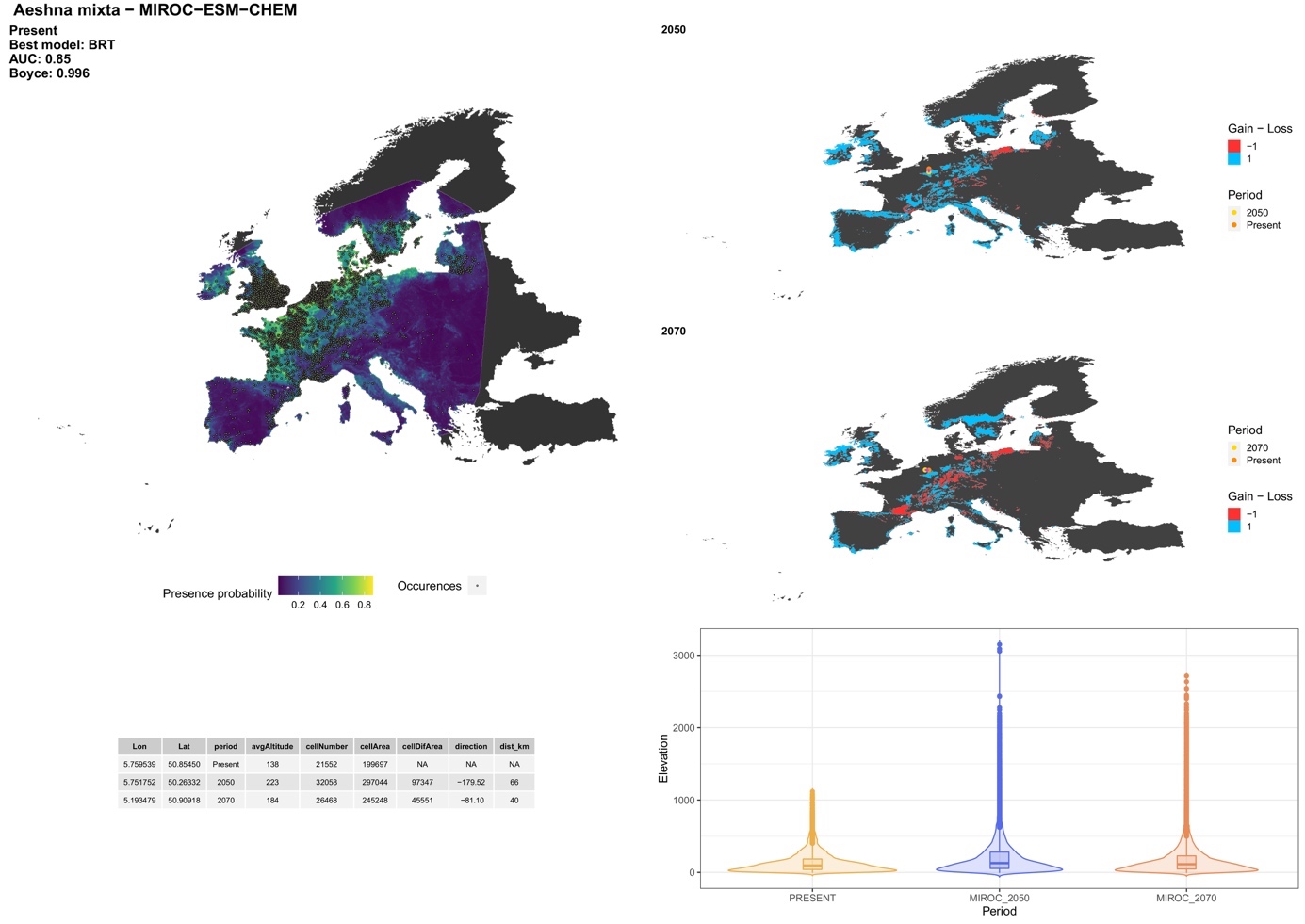


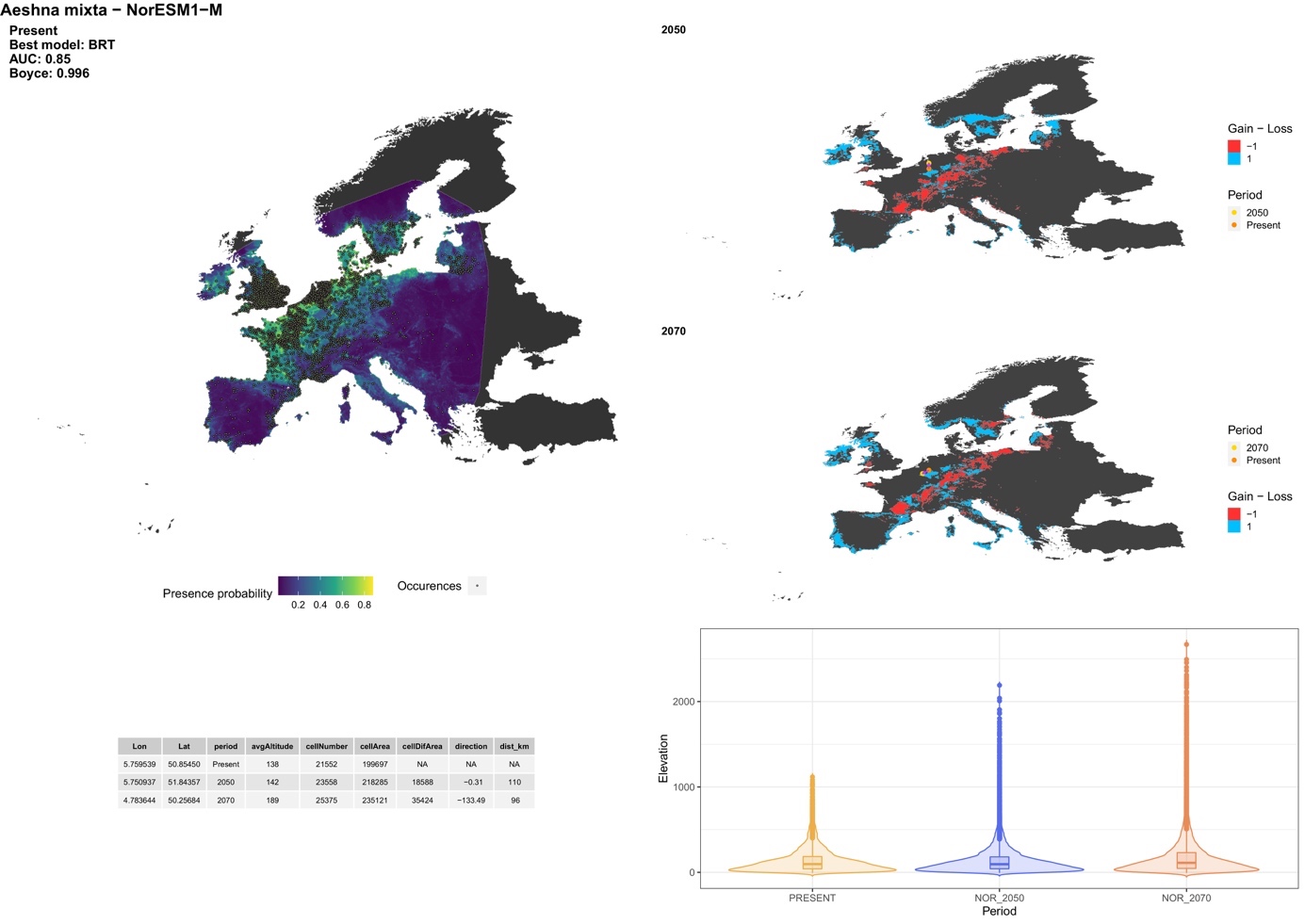


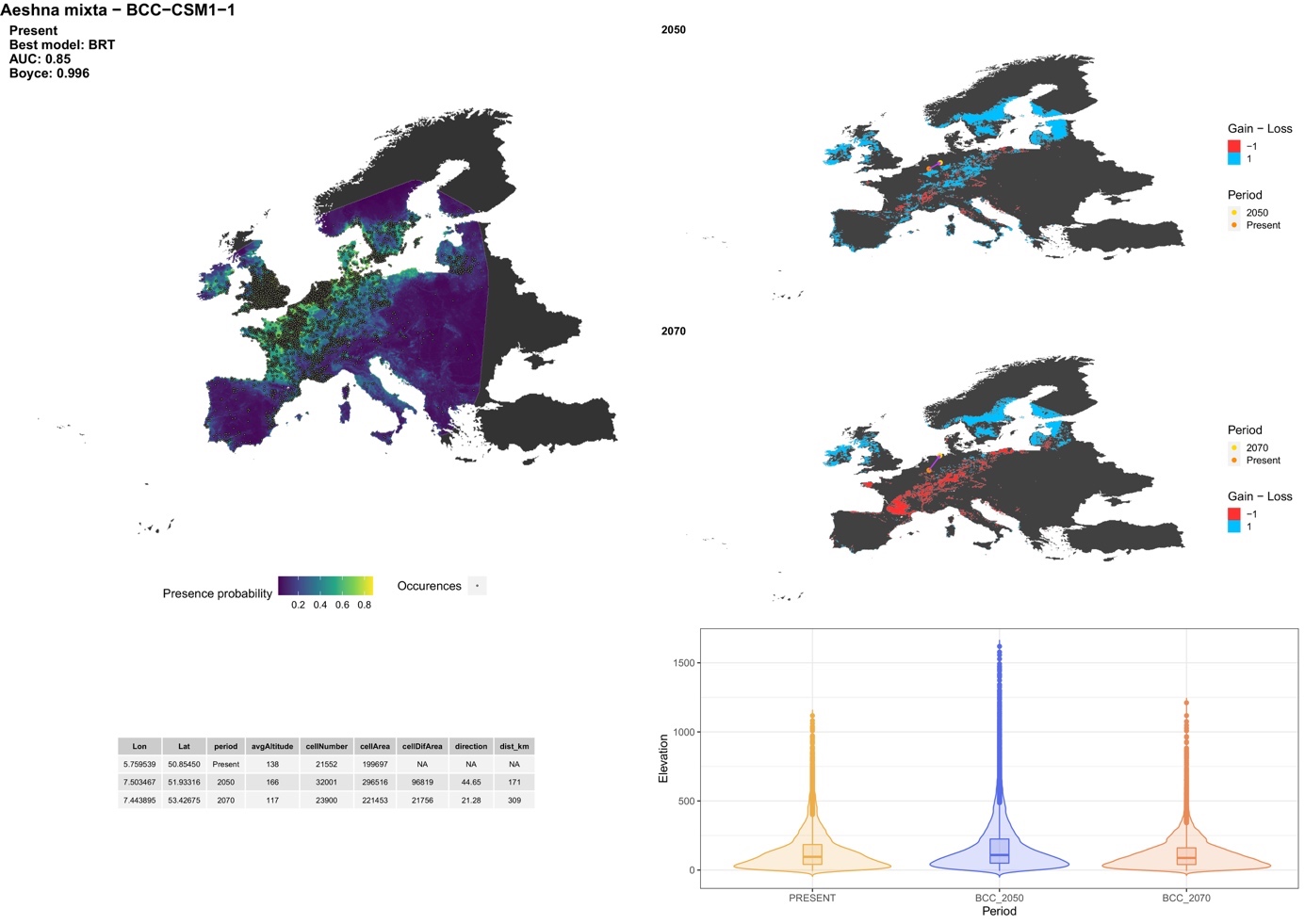


### *Aeshna serrata* Hagen, 1856


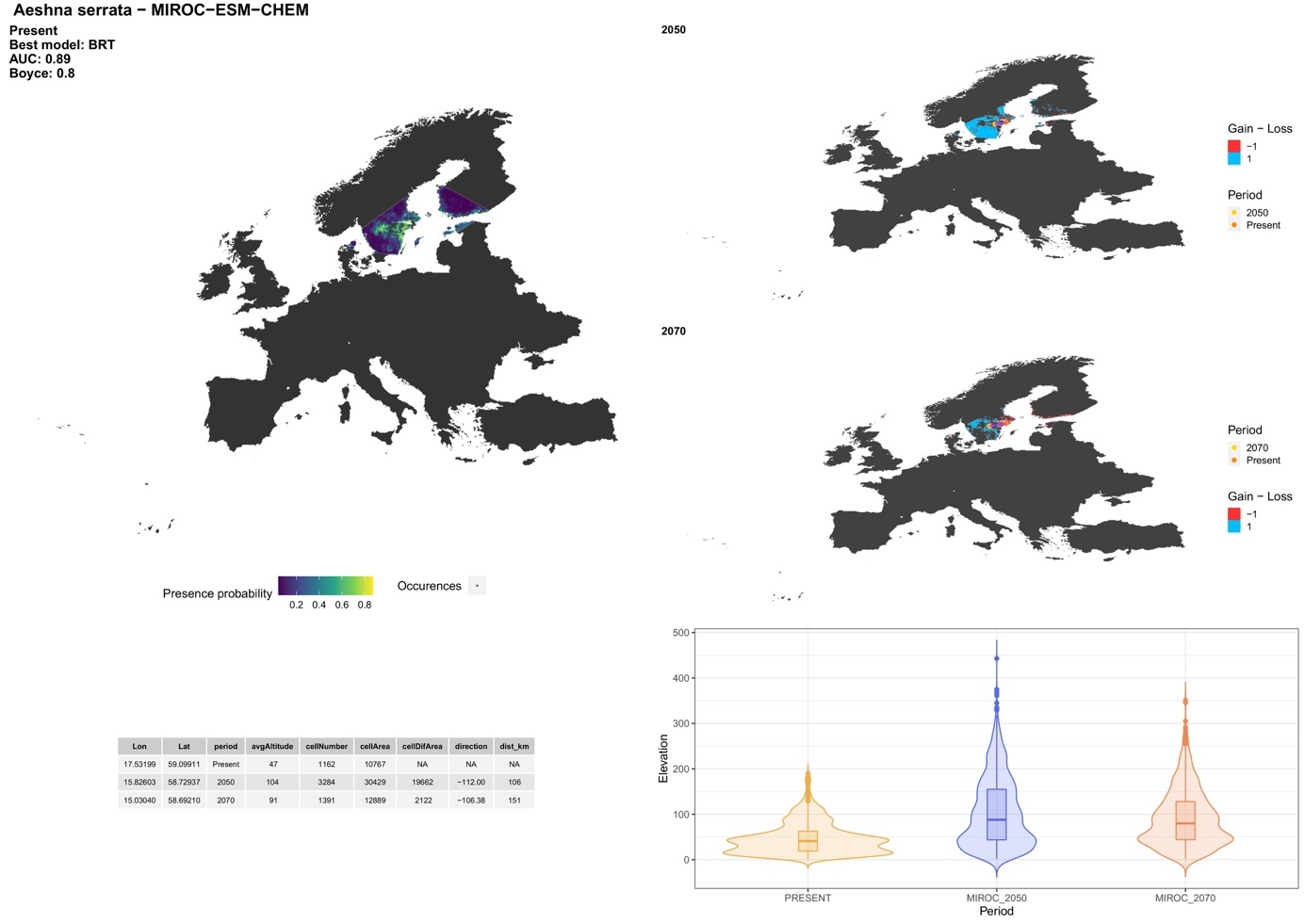


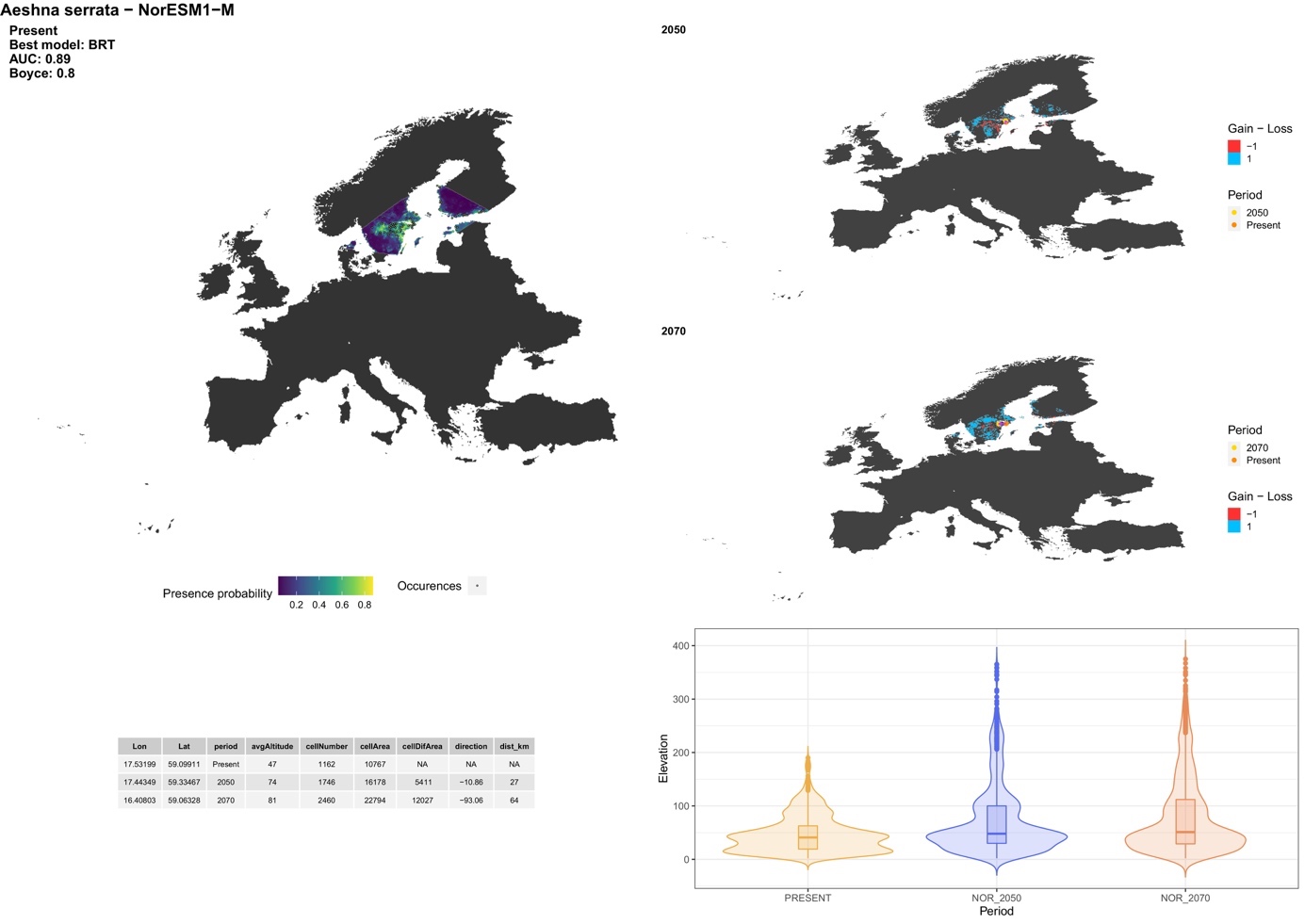


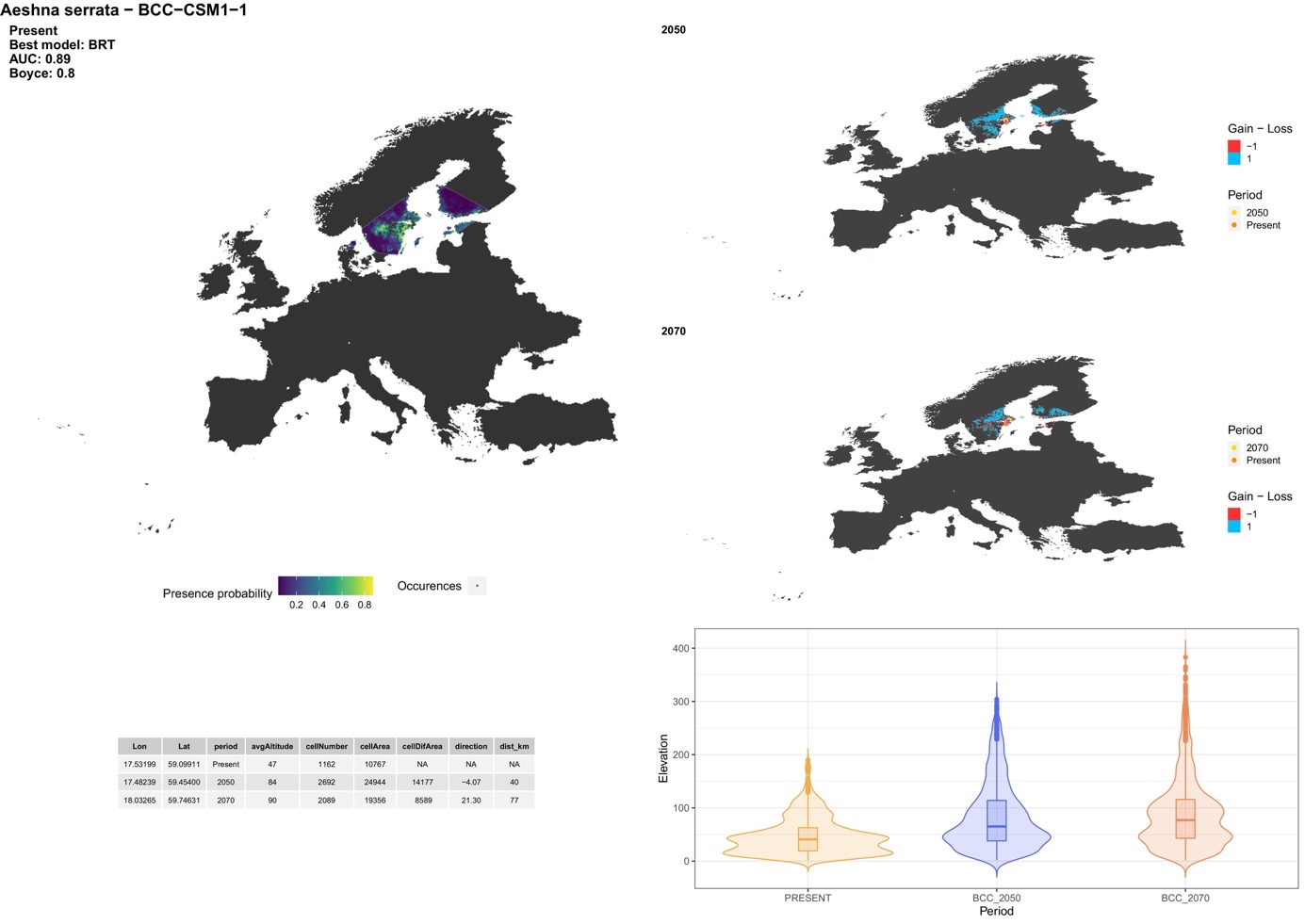


### *Aeshna subarctica* Walker, 1908


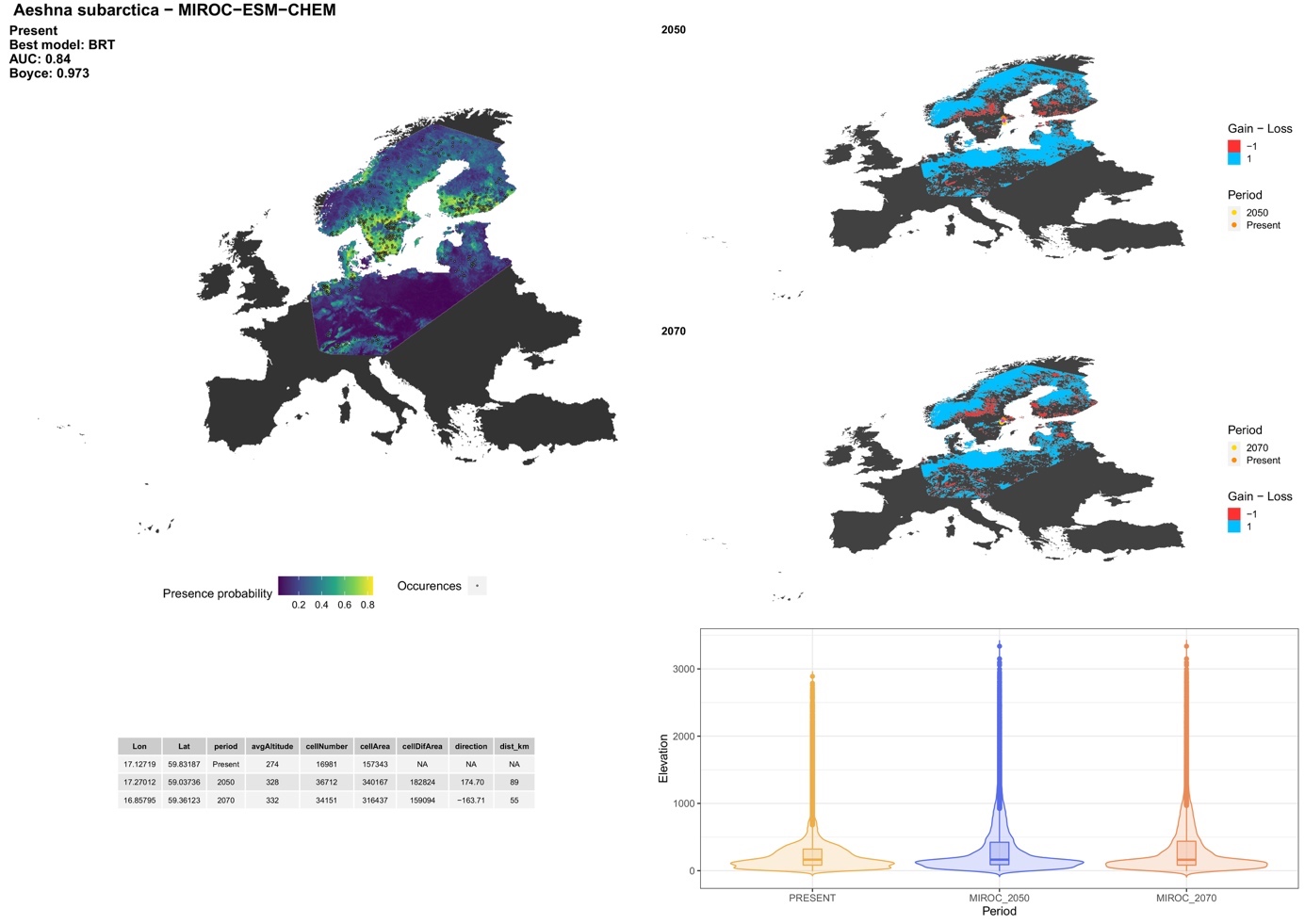


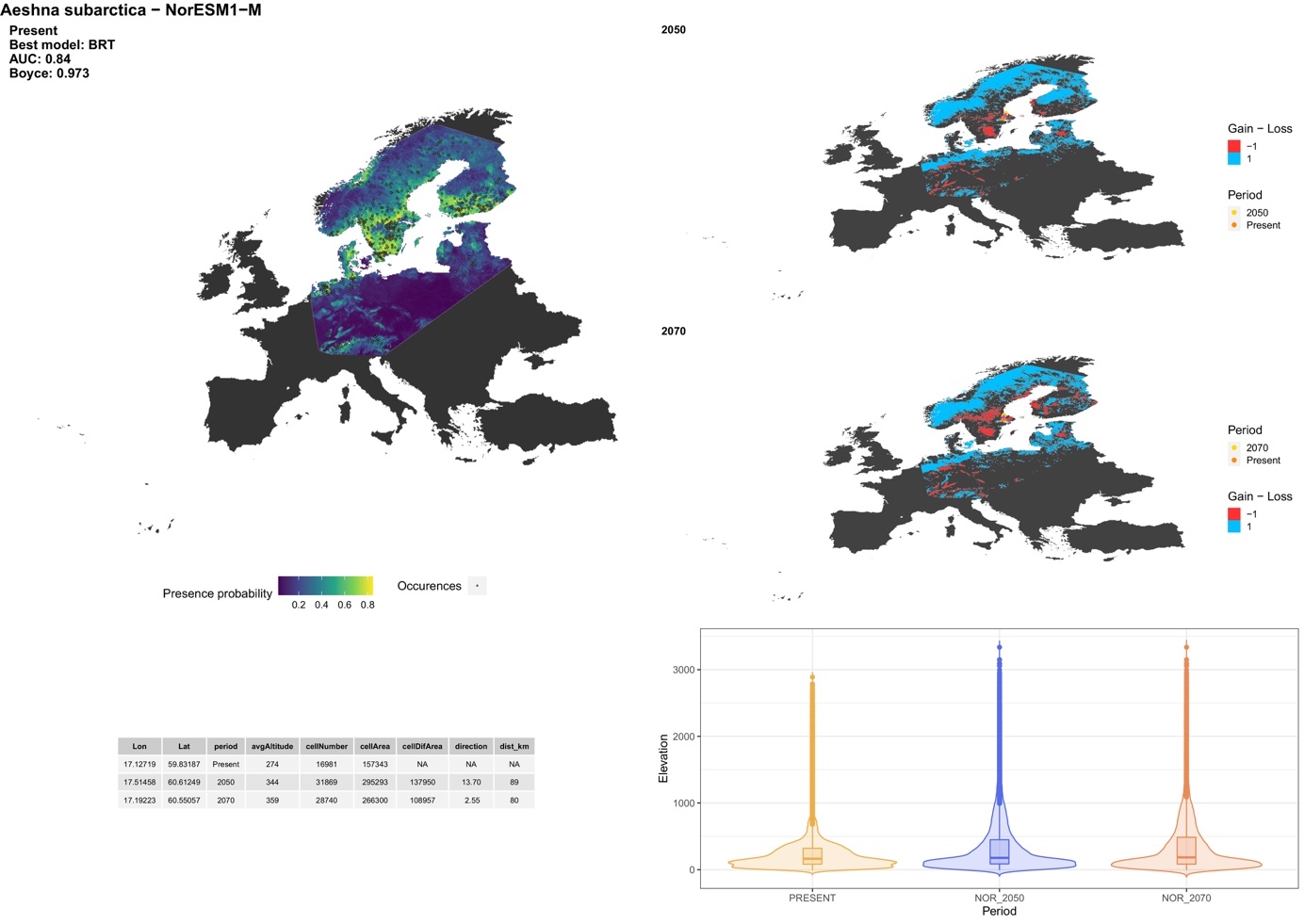


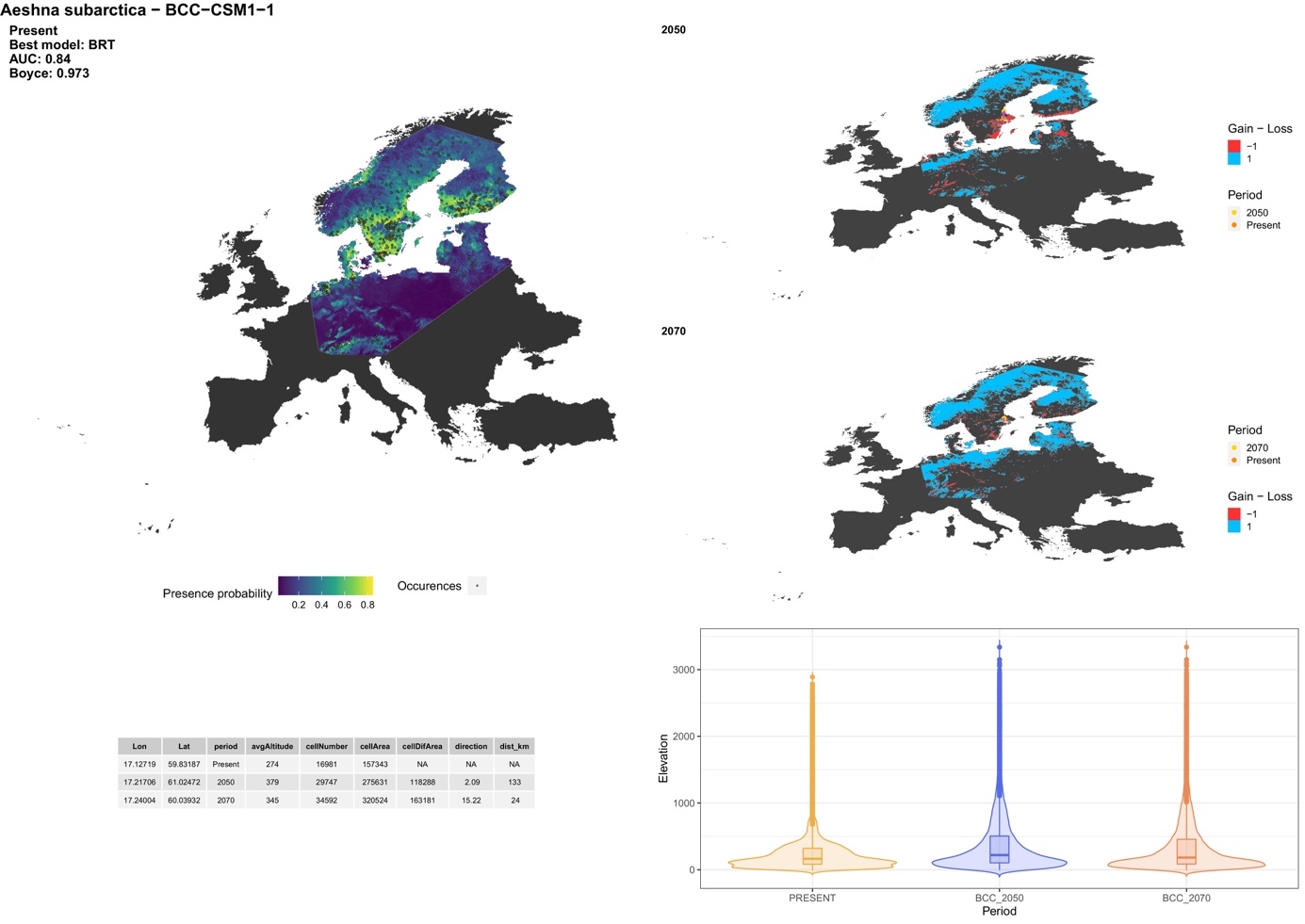


### *Aeshna viridis* Eversmann, 1836


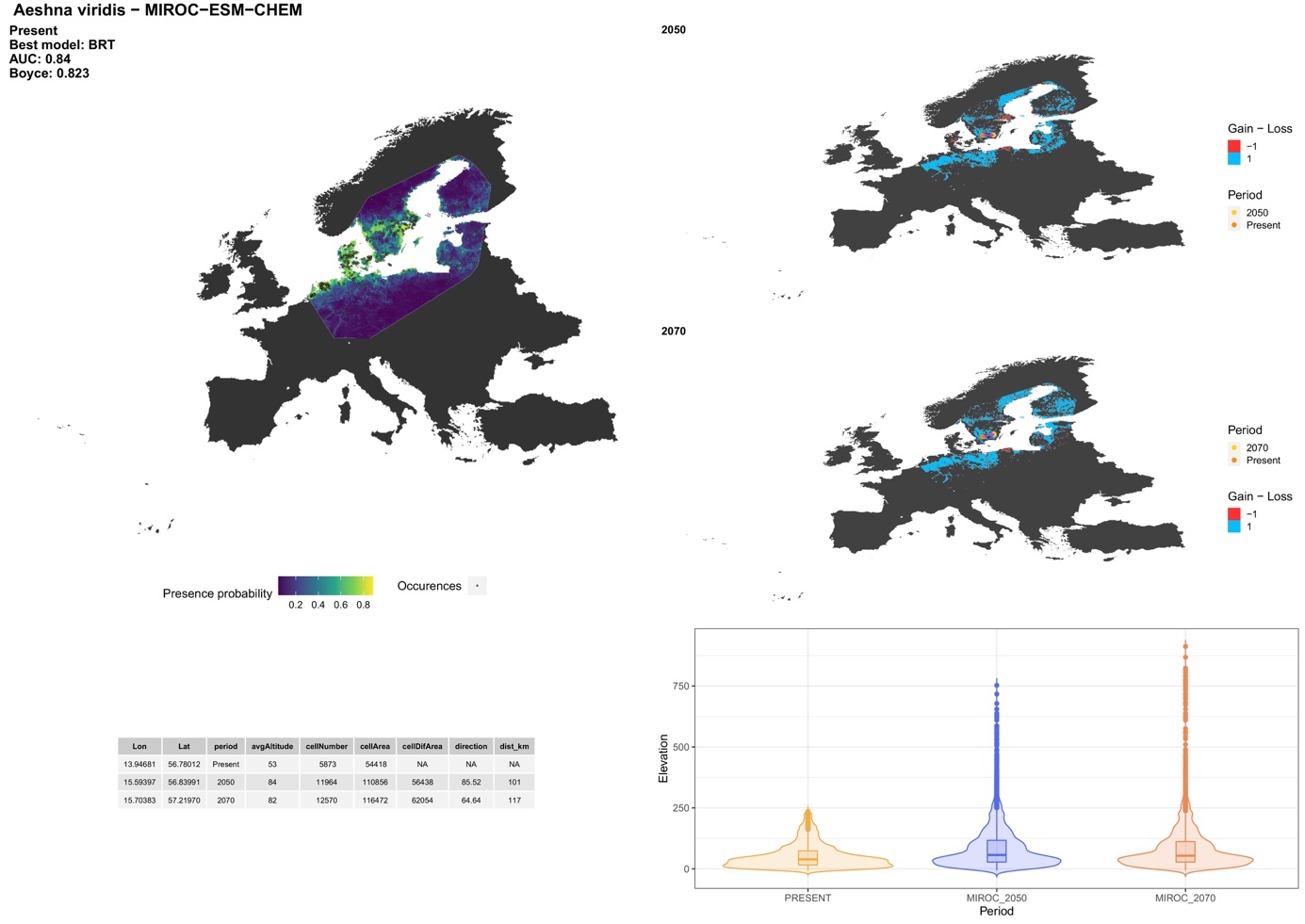


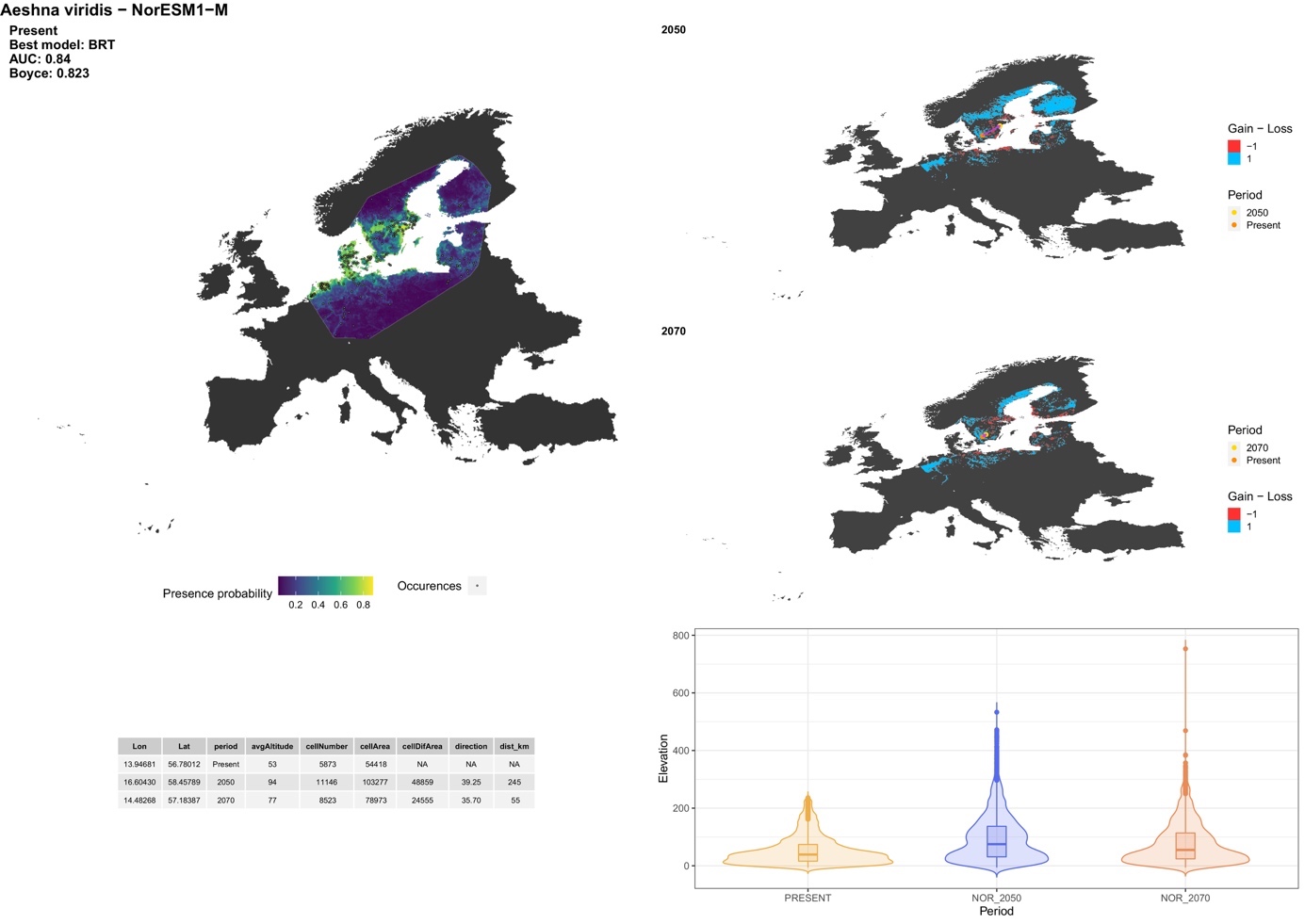


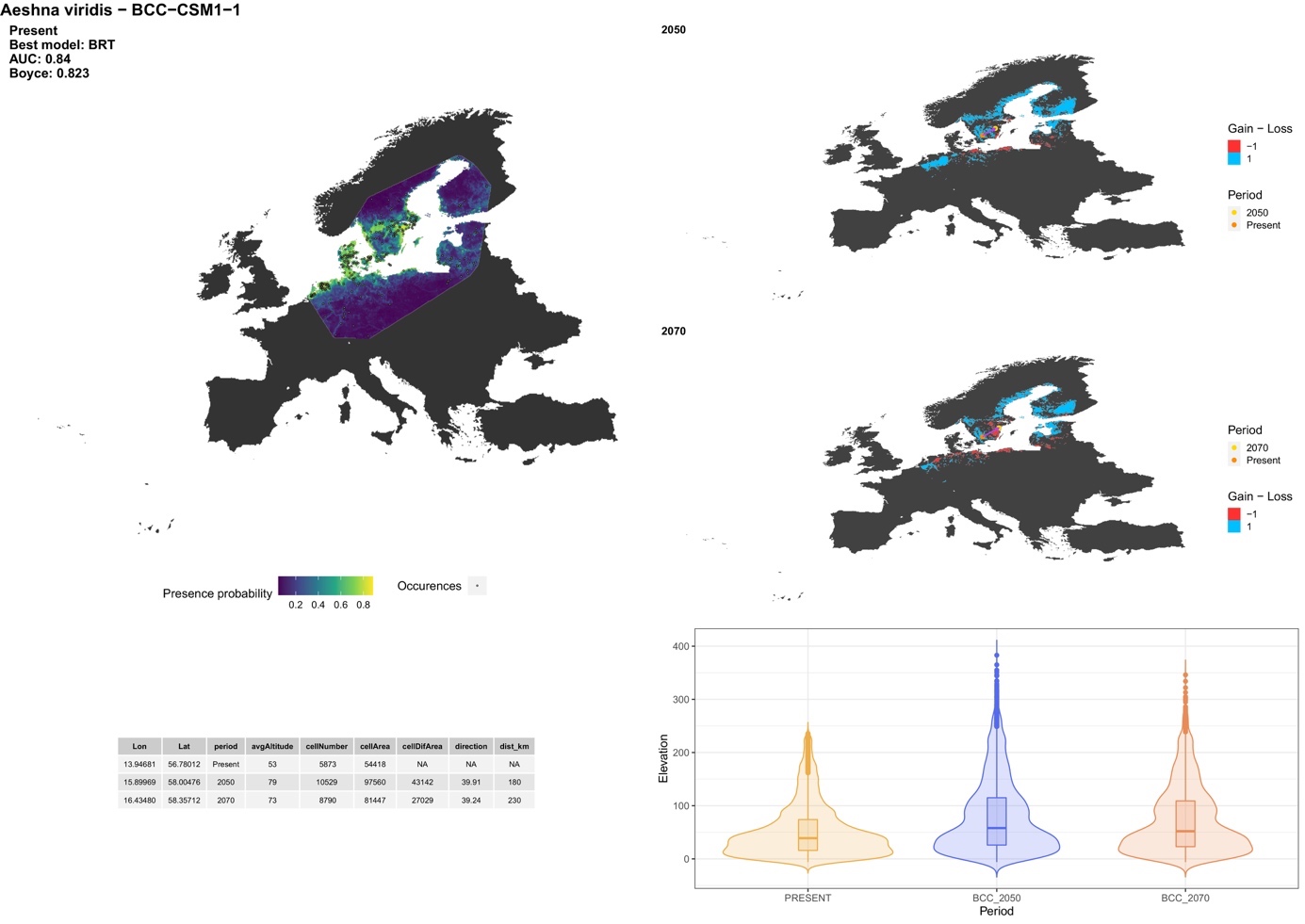


### *Anax ephippiger* (Burmeister, 1839)


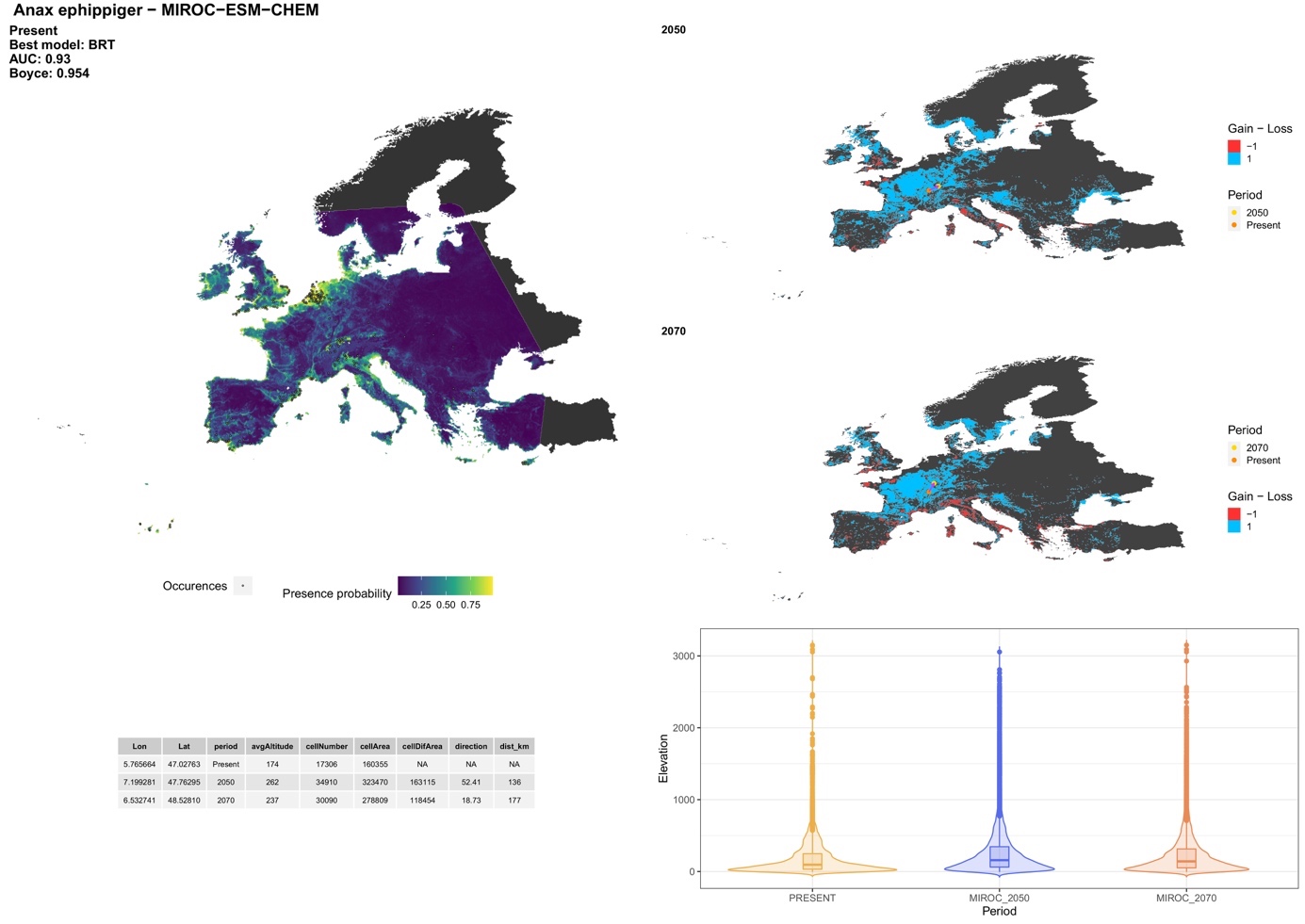


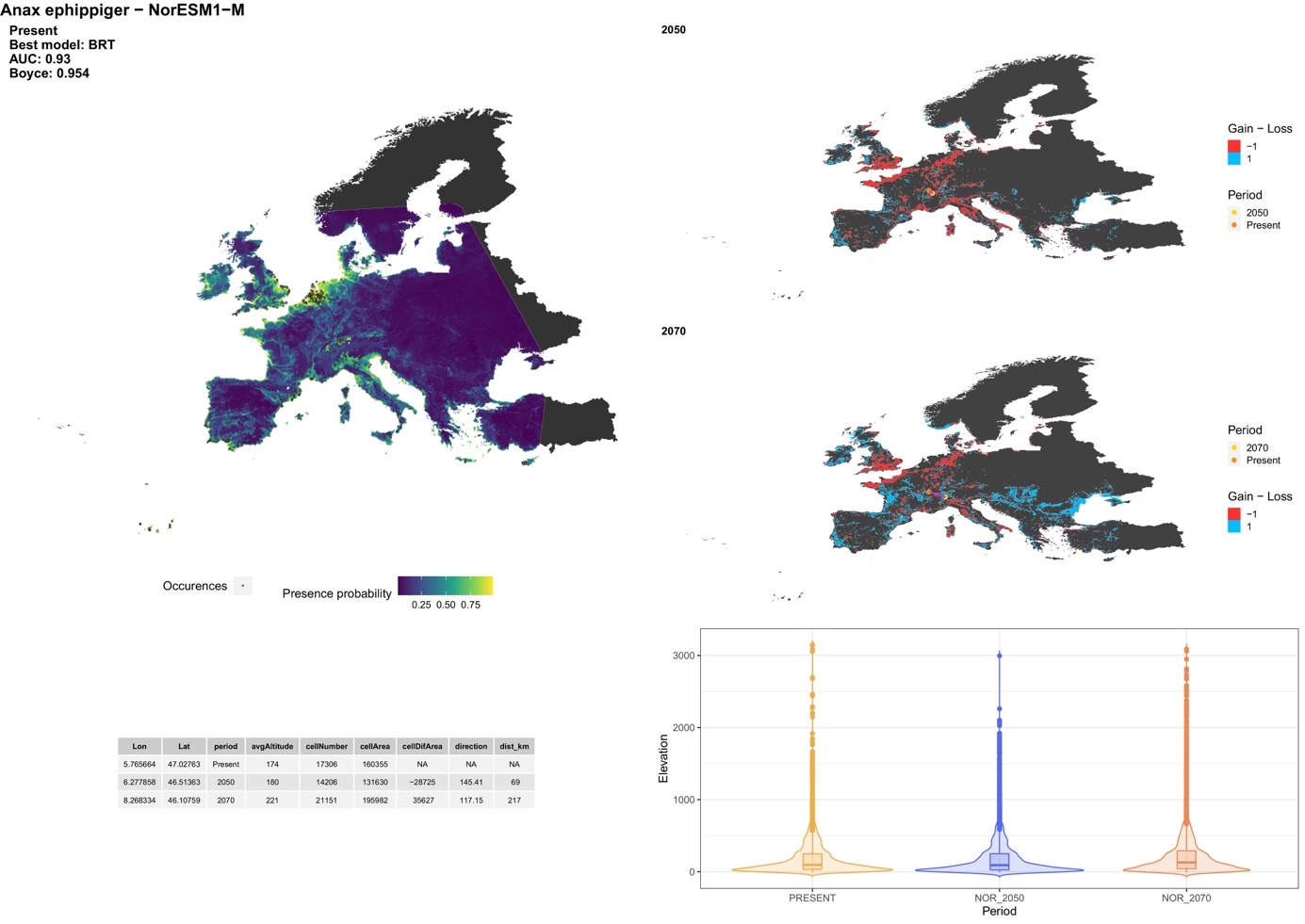


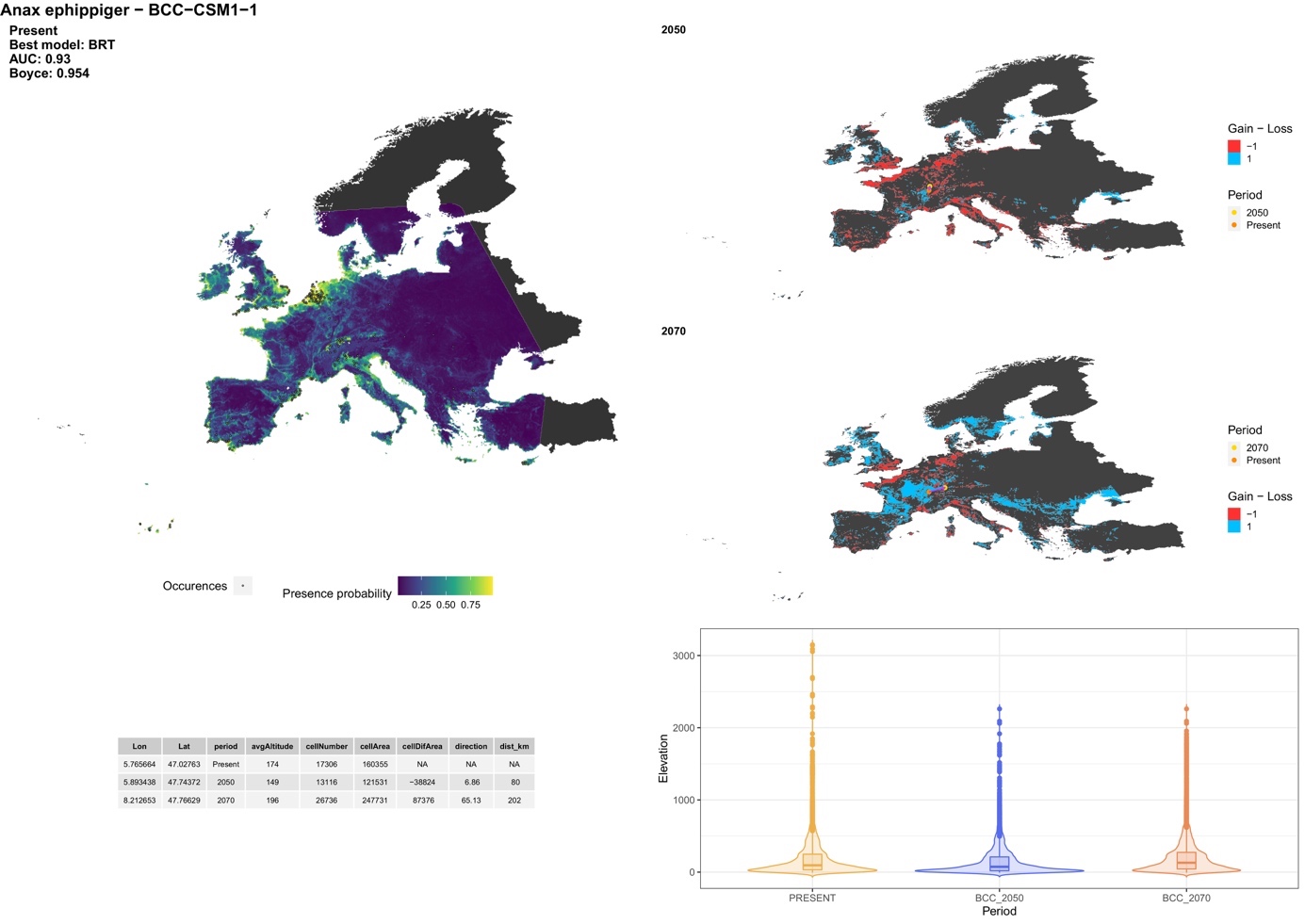


### *Anax imperator* Leach, 1815


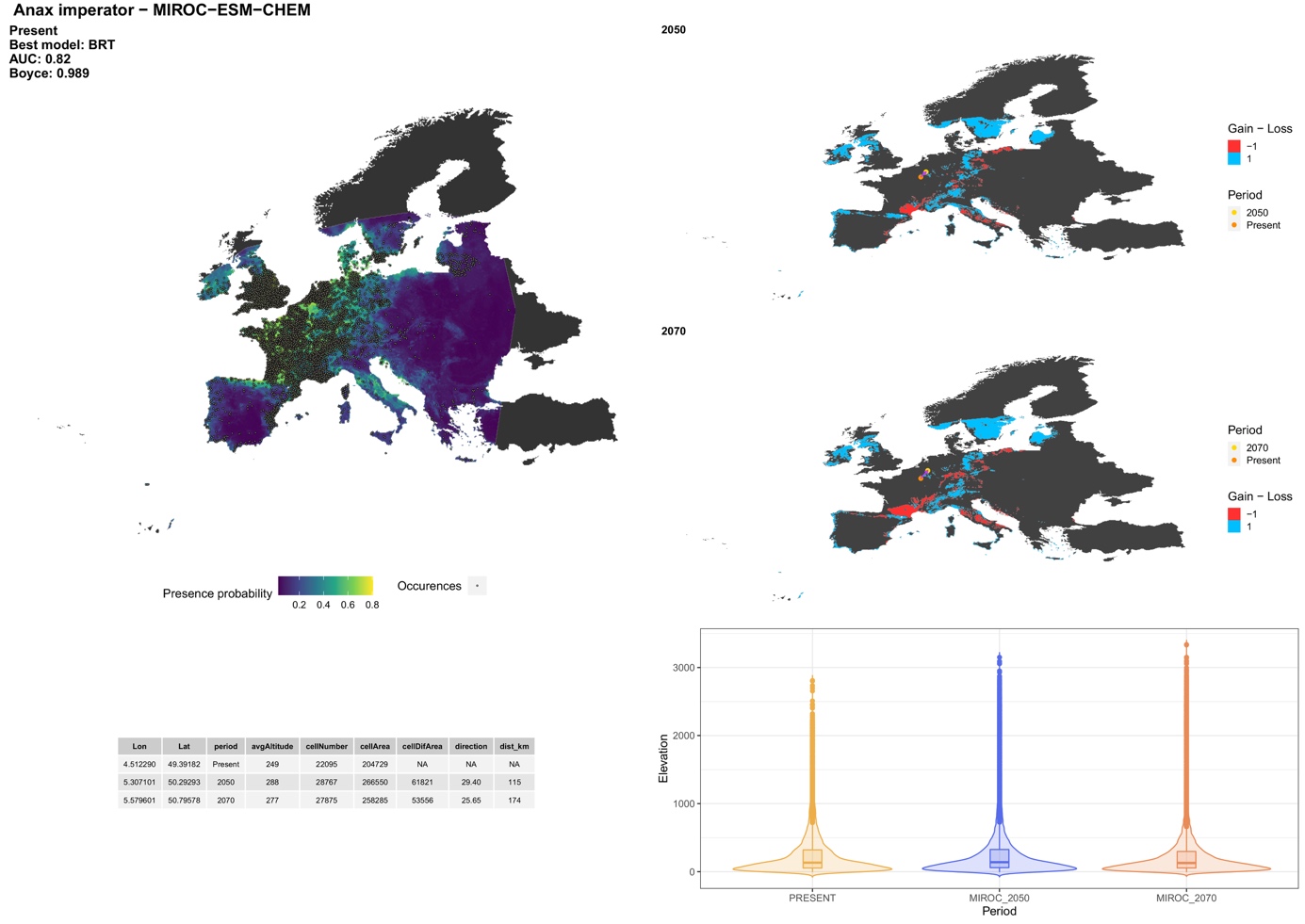

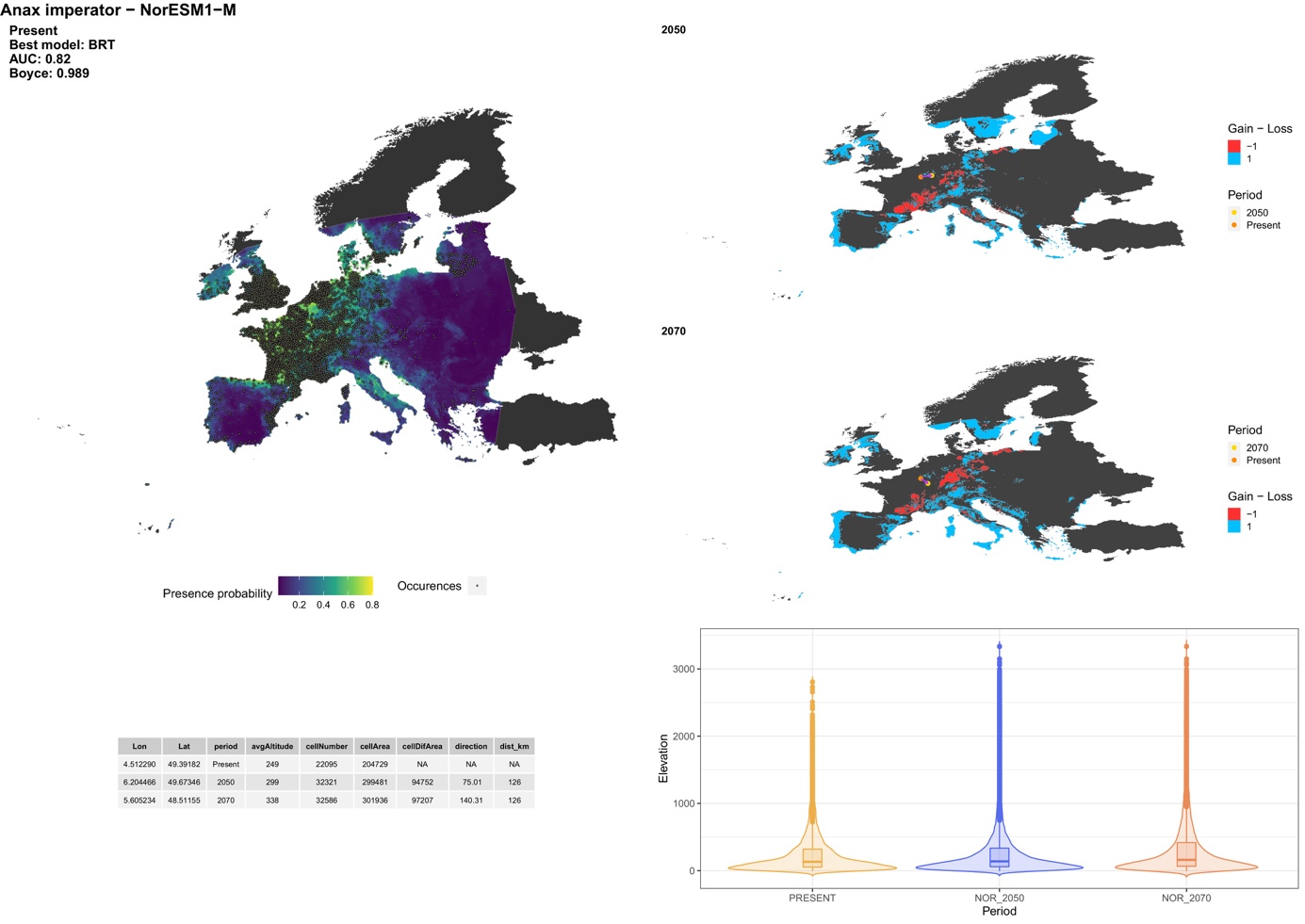


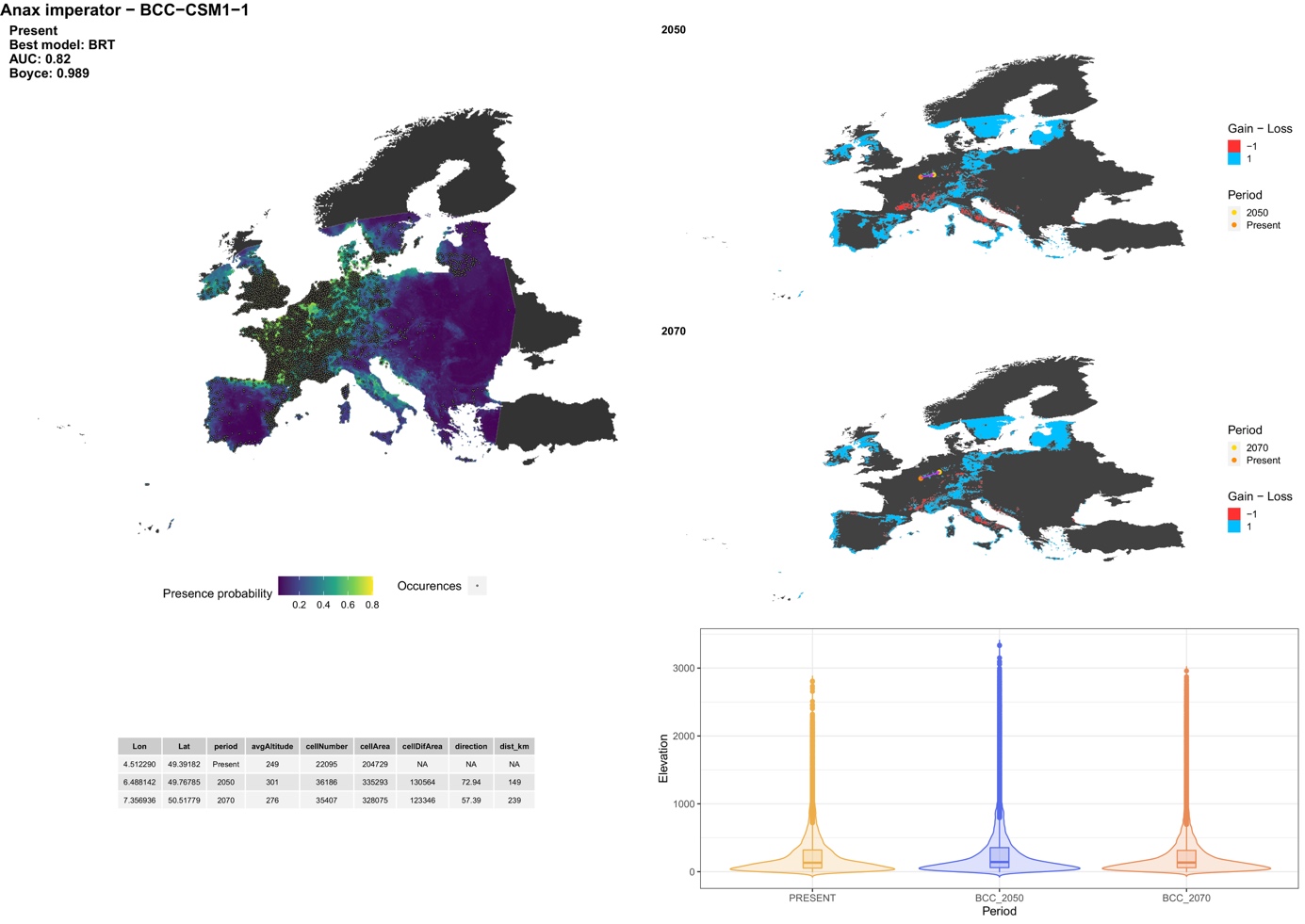


### *Anax parthenope* (Selys, 1839)


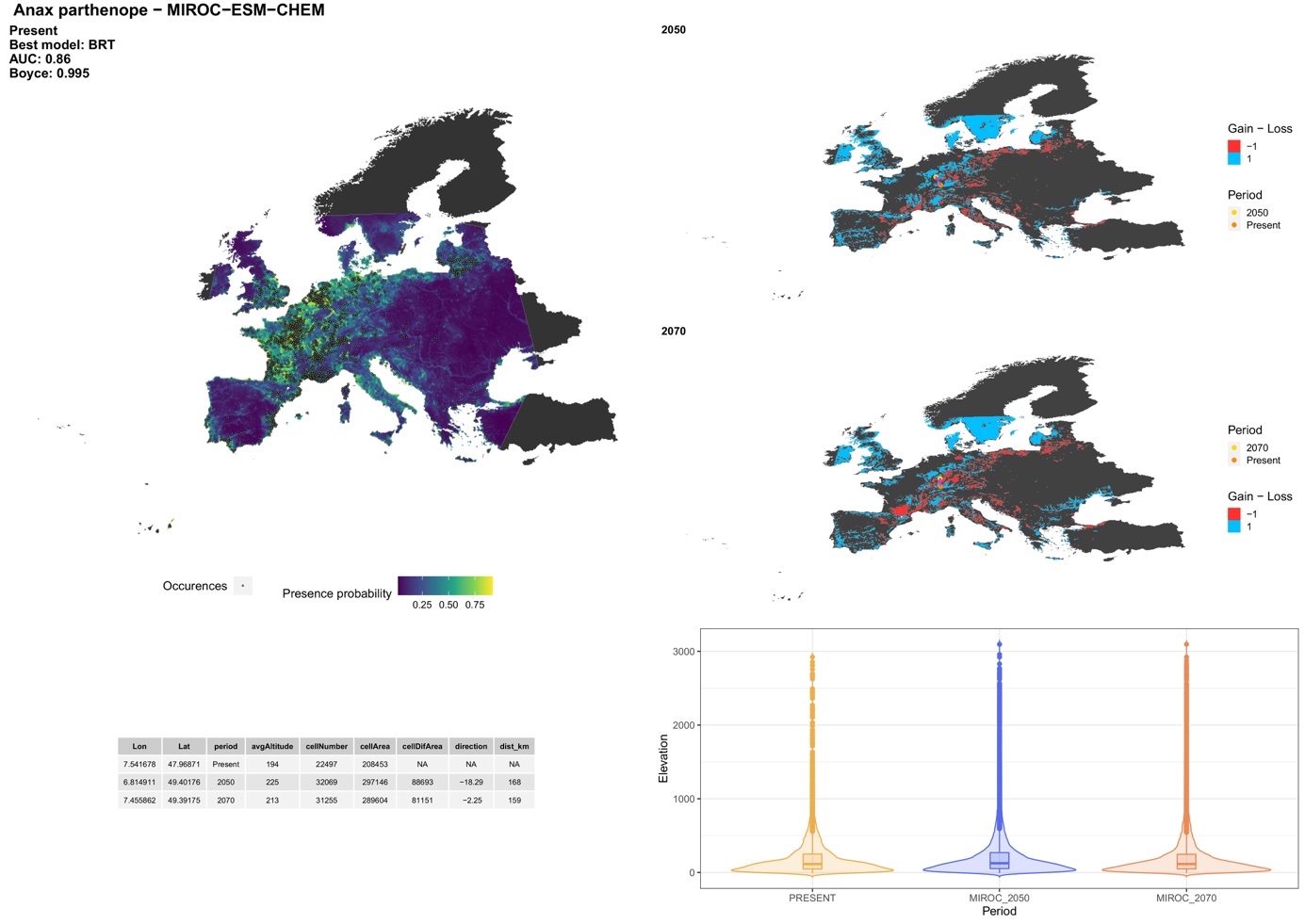


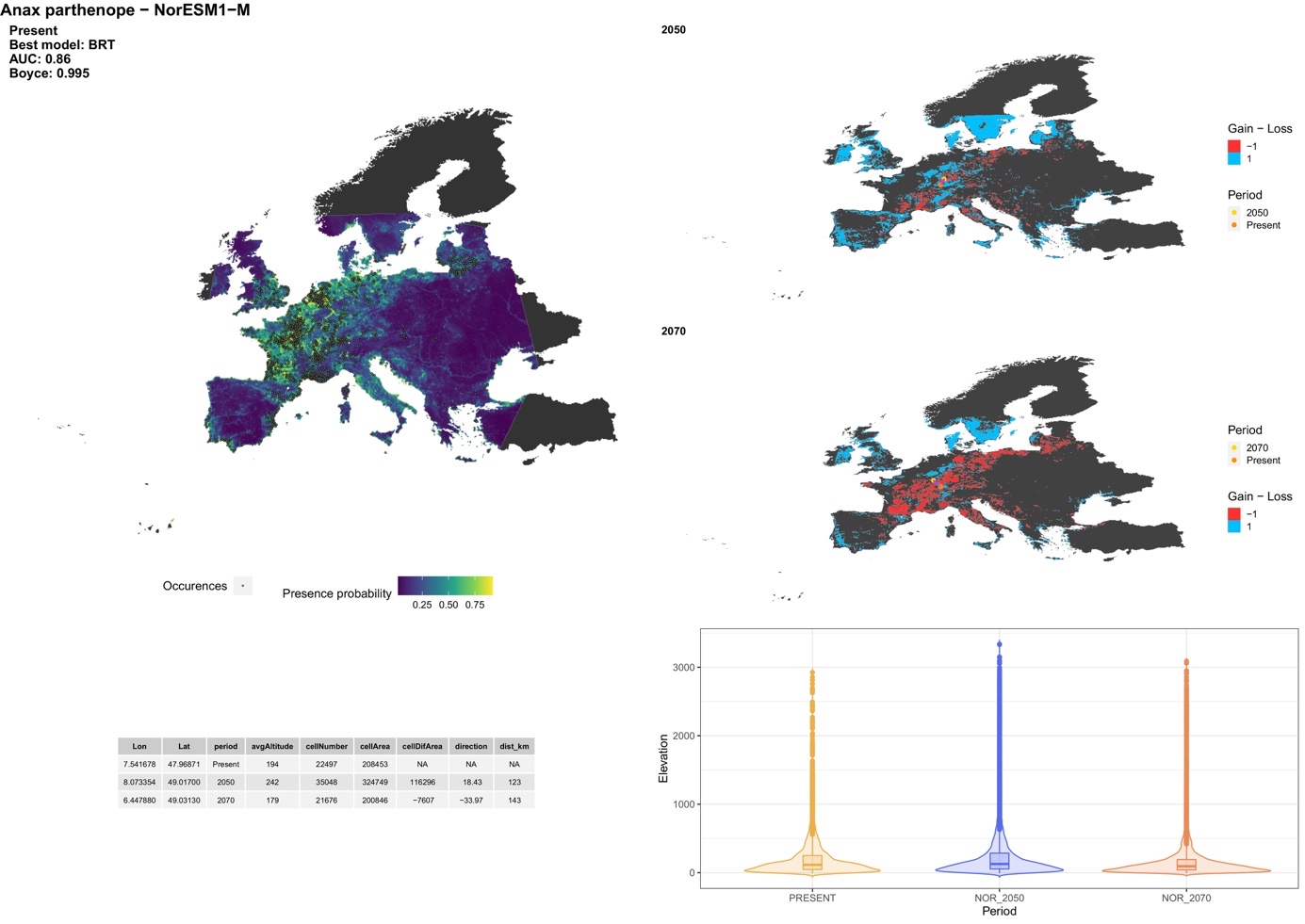


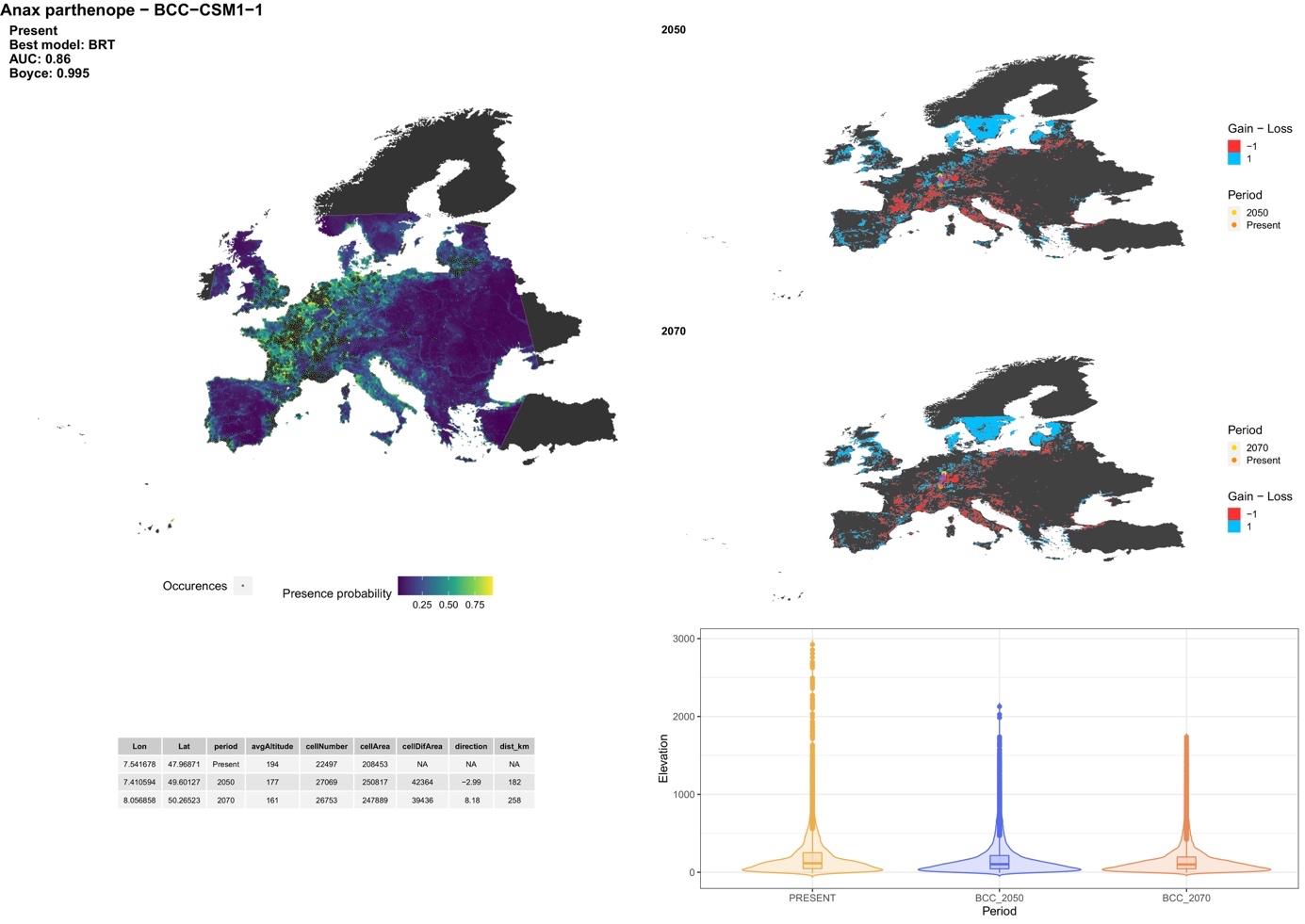


### *Boyeria irene* (Fonscolombe, 1838)


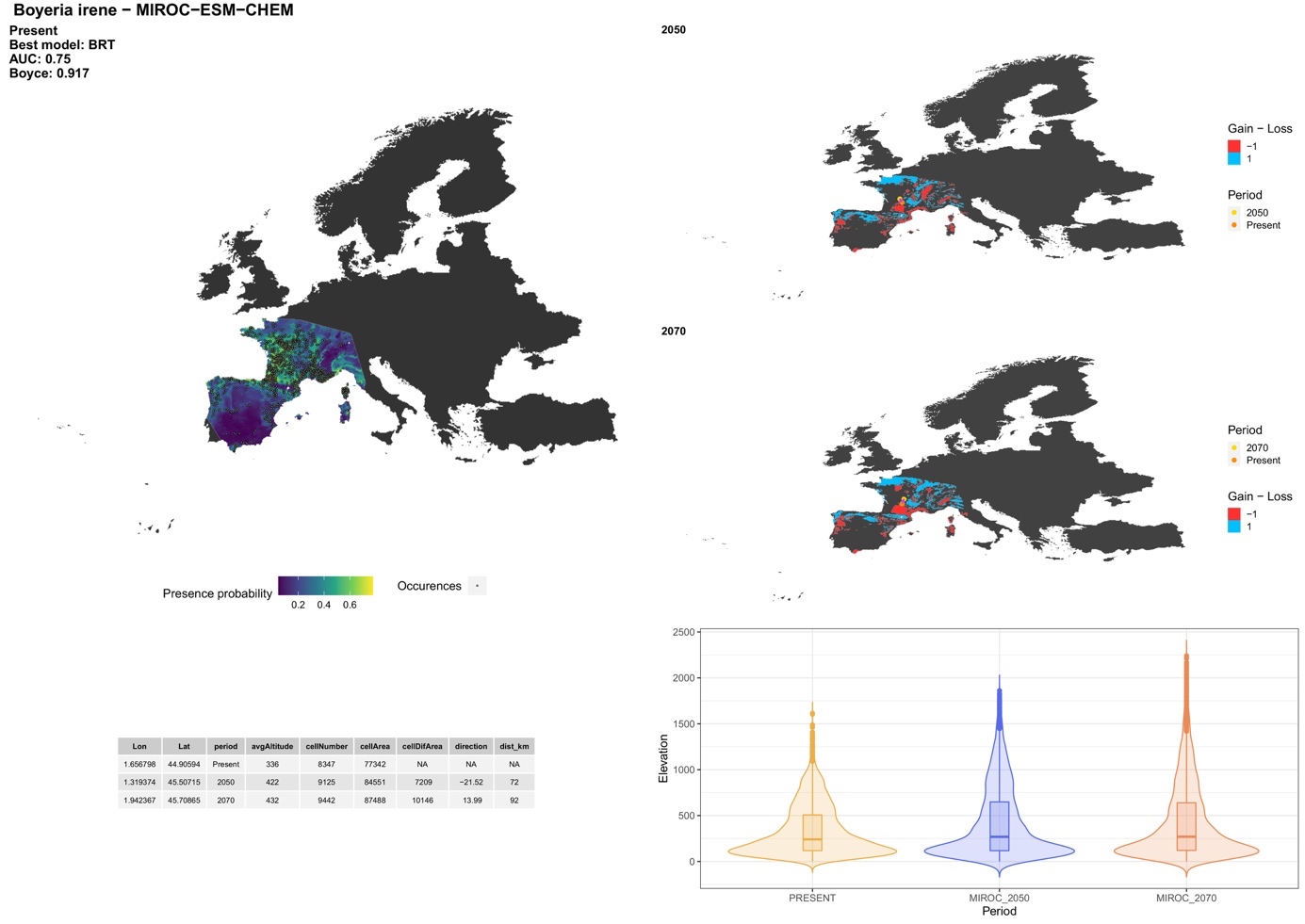


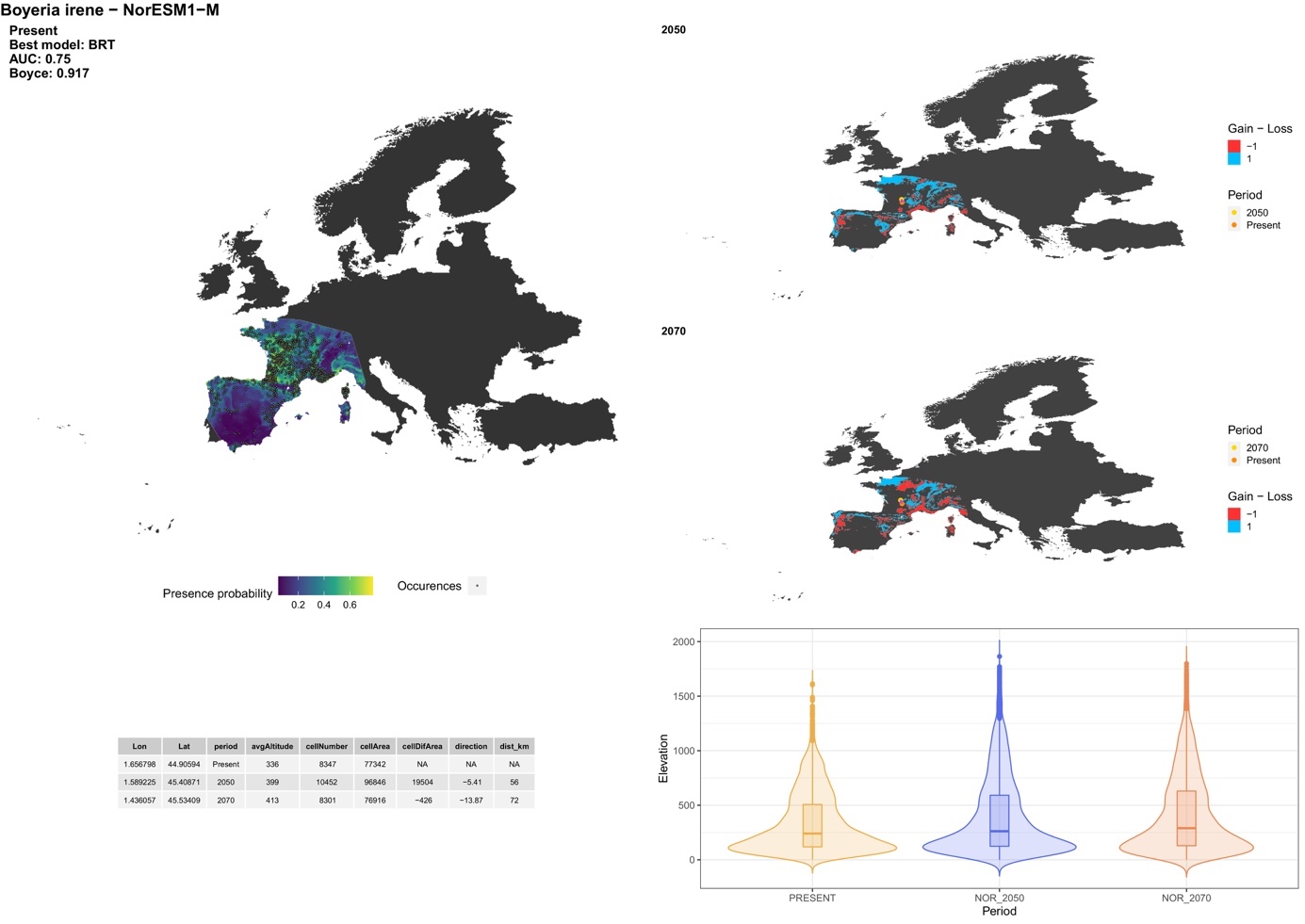


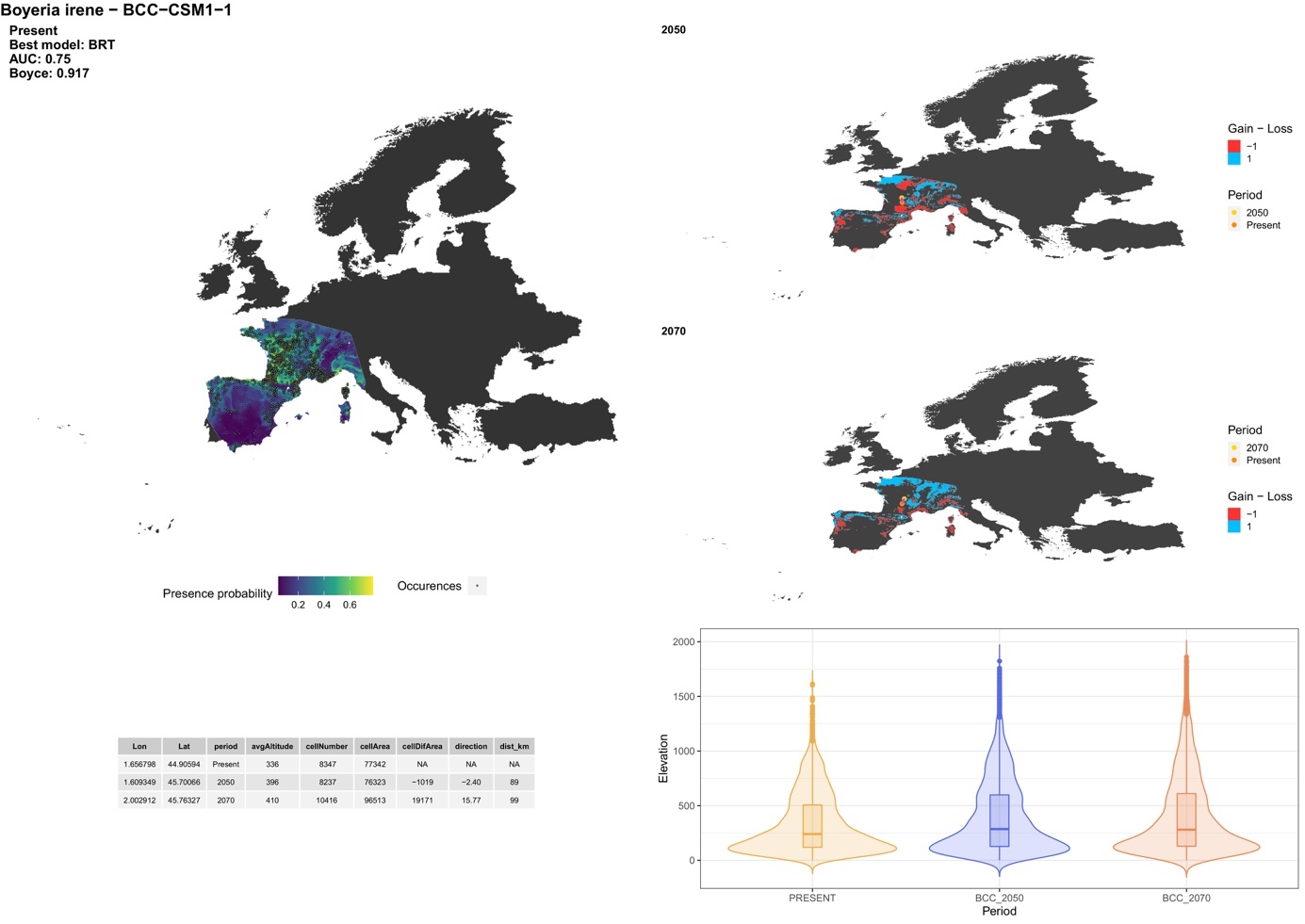


### *Brachytron pratense* (Müller, 1764)


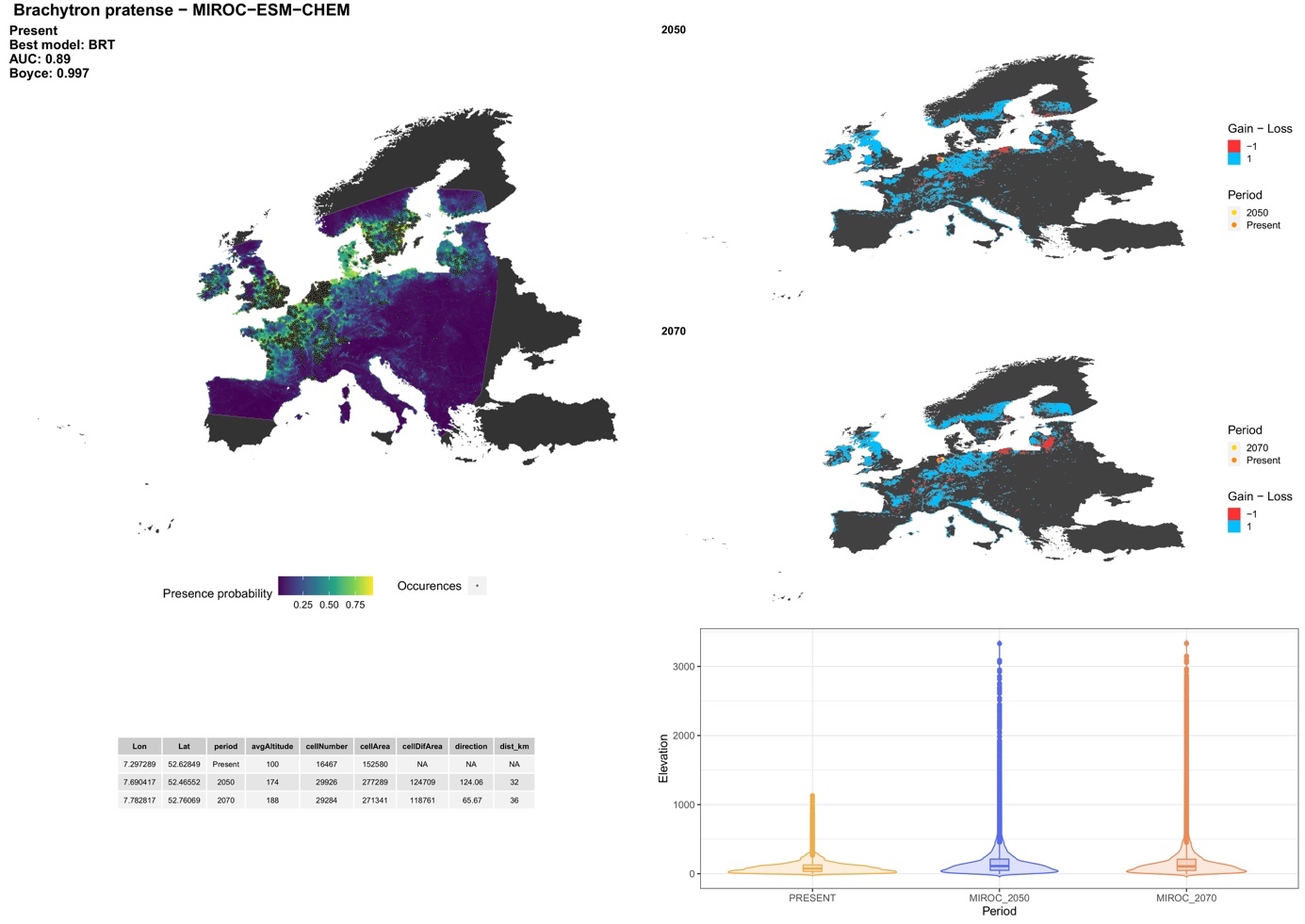


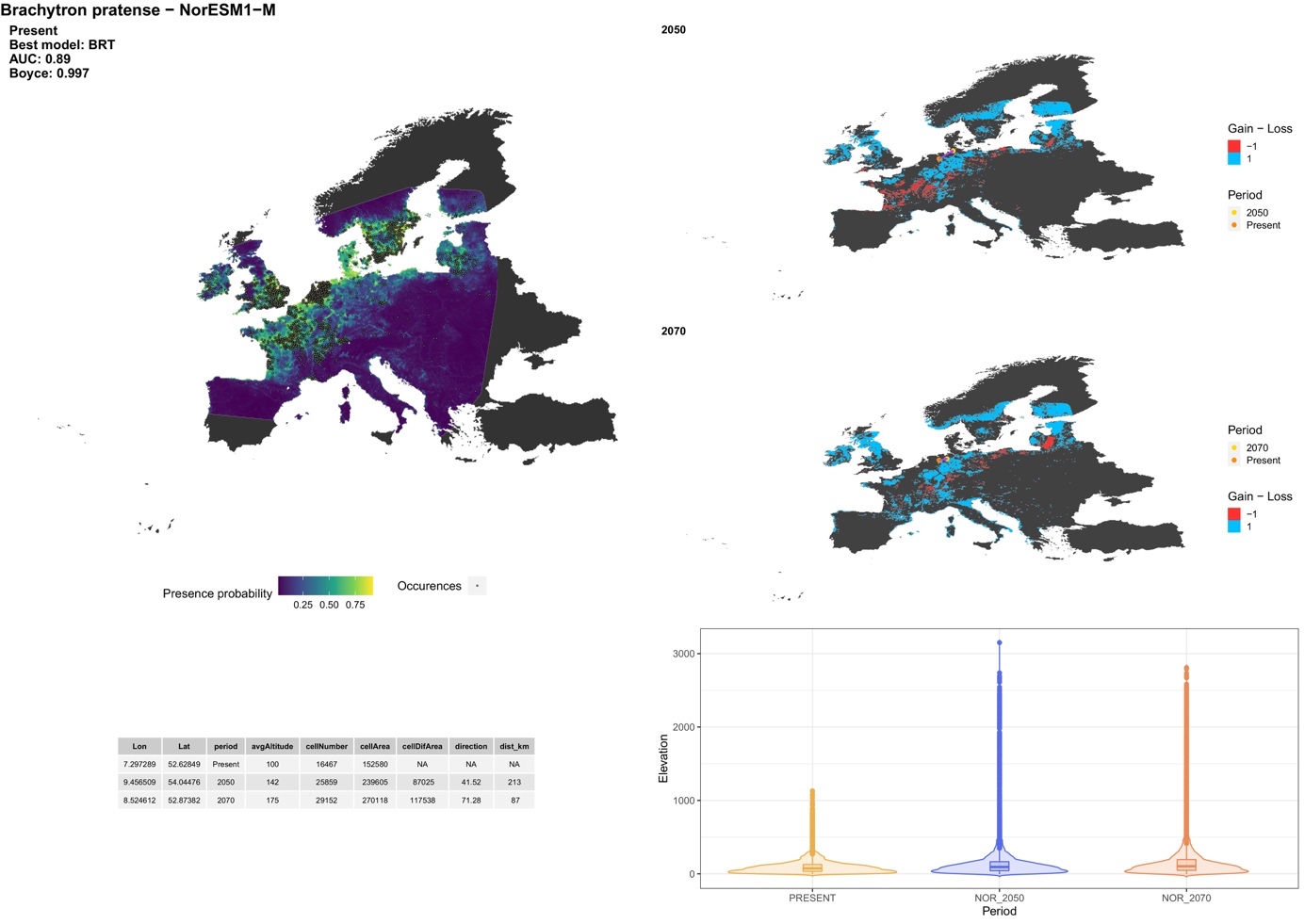


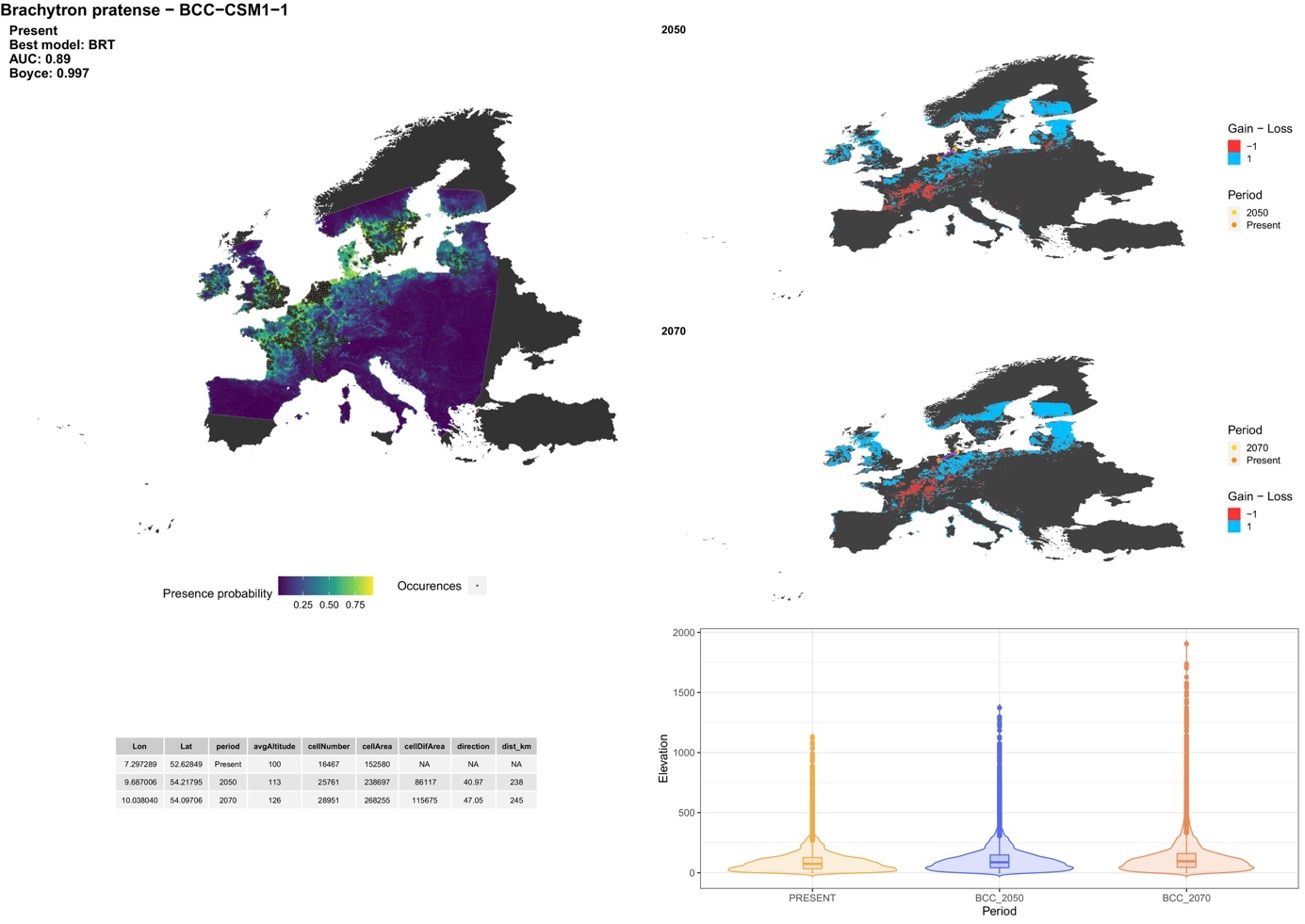


### *Caliaeschna microstigma* (Schneider, 1845)


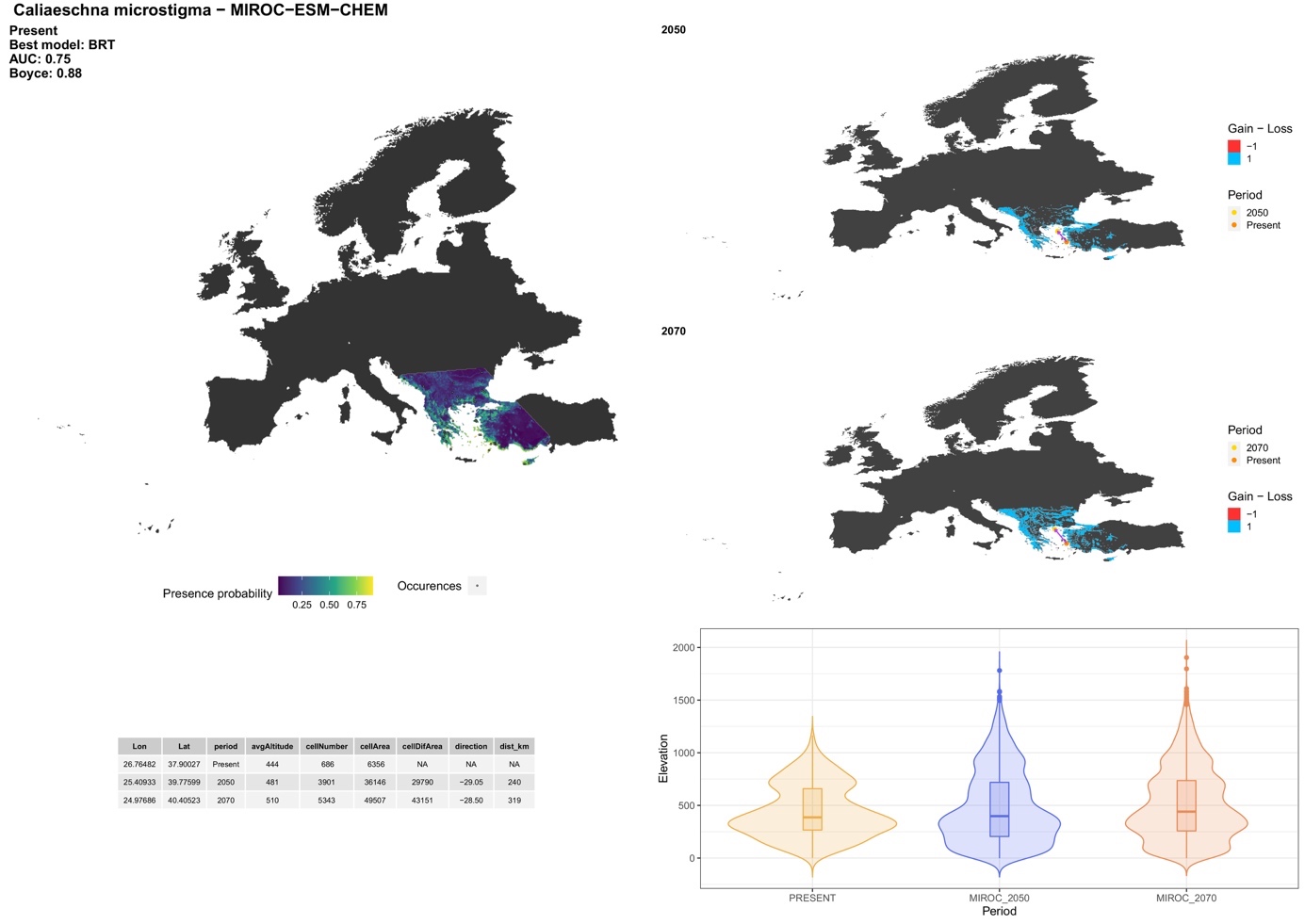


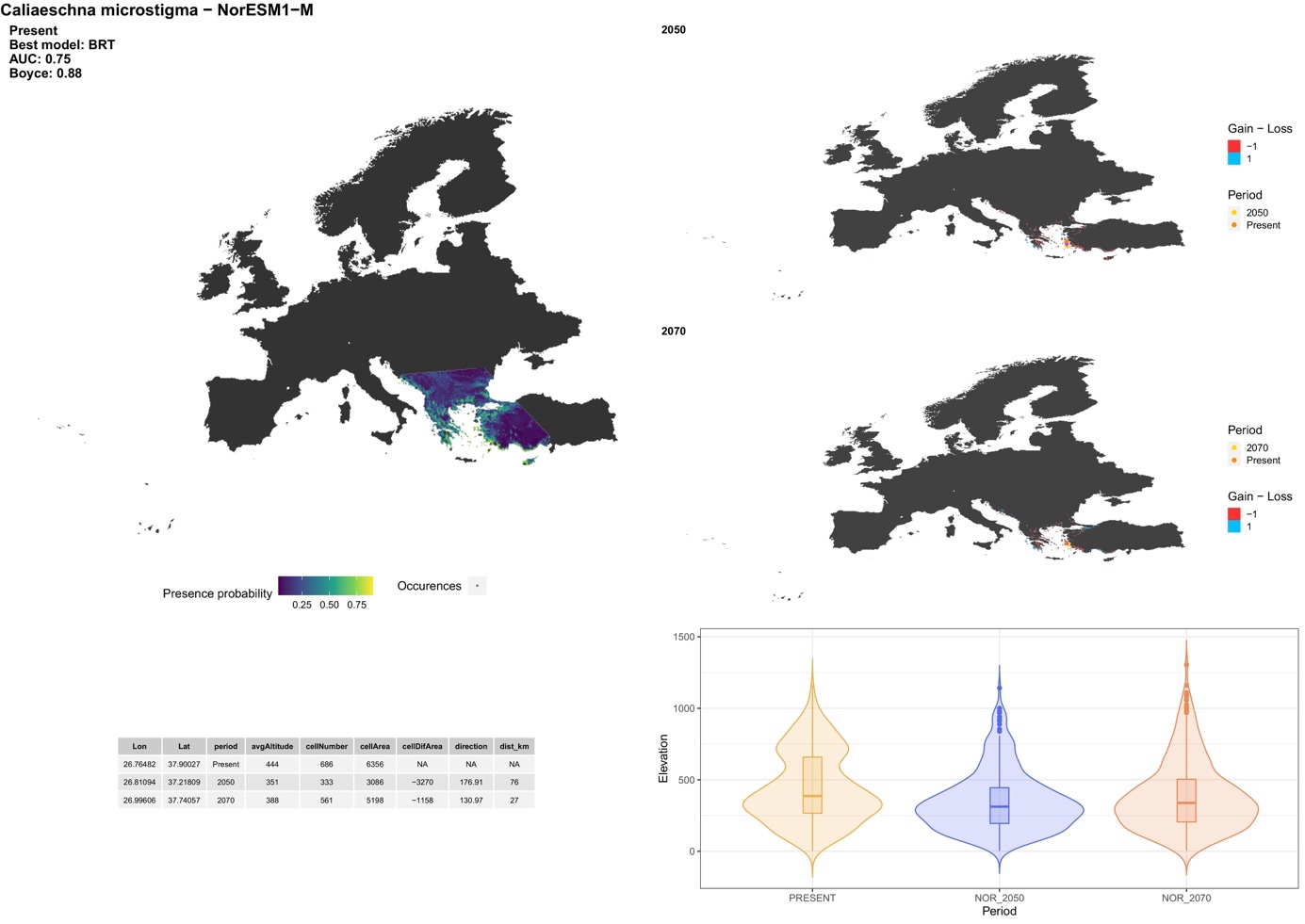


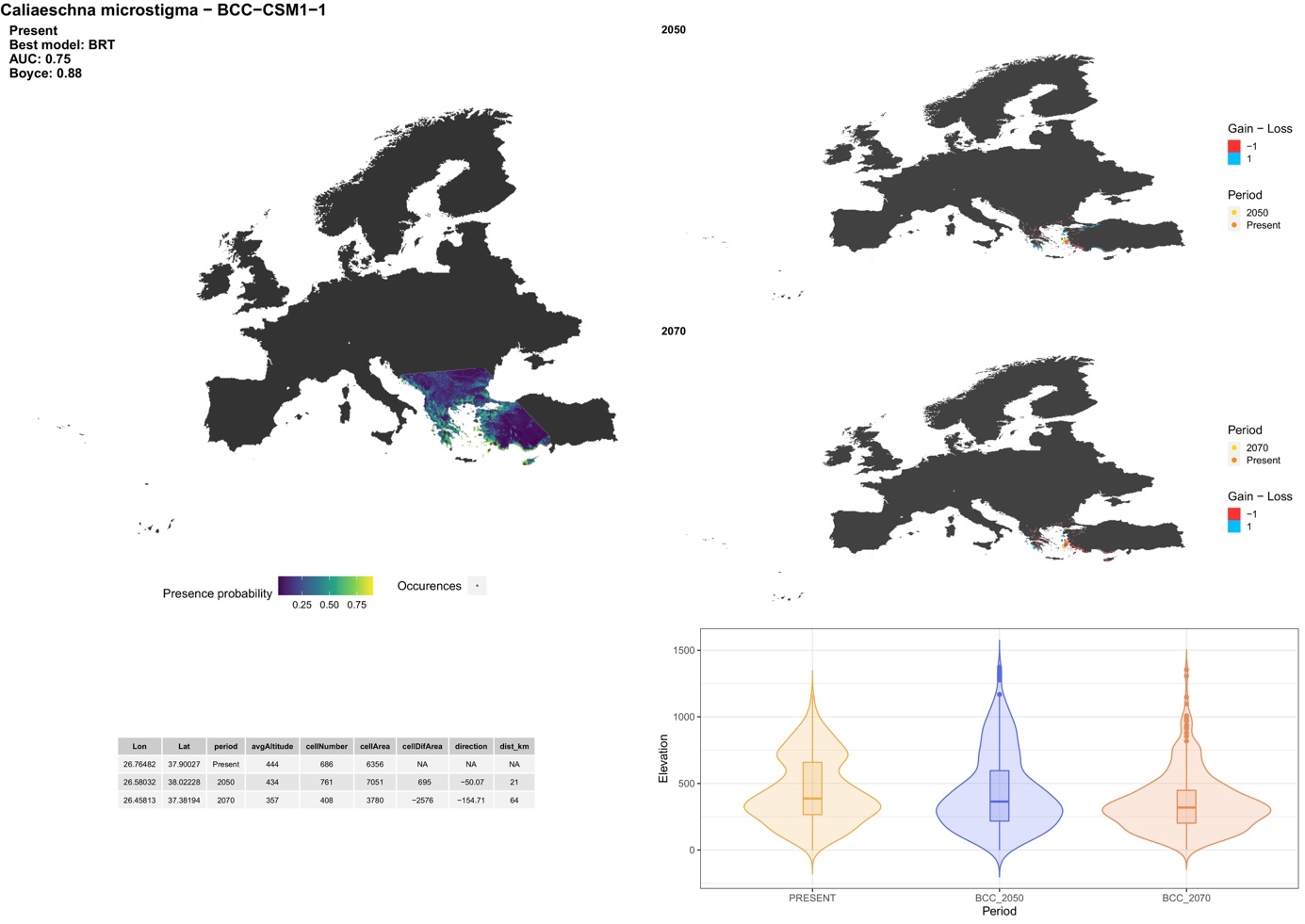


### **Family: Gomphidae**

### *Gomphus graslinii* Rambur, 1842


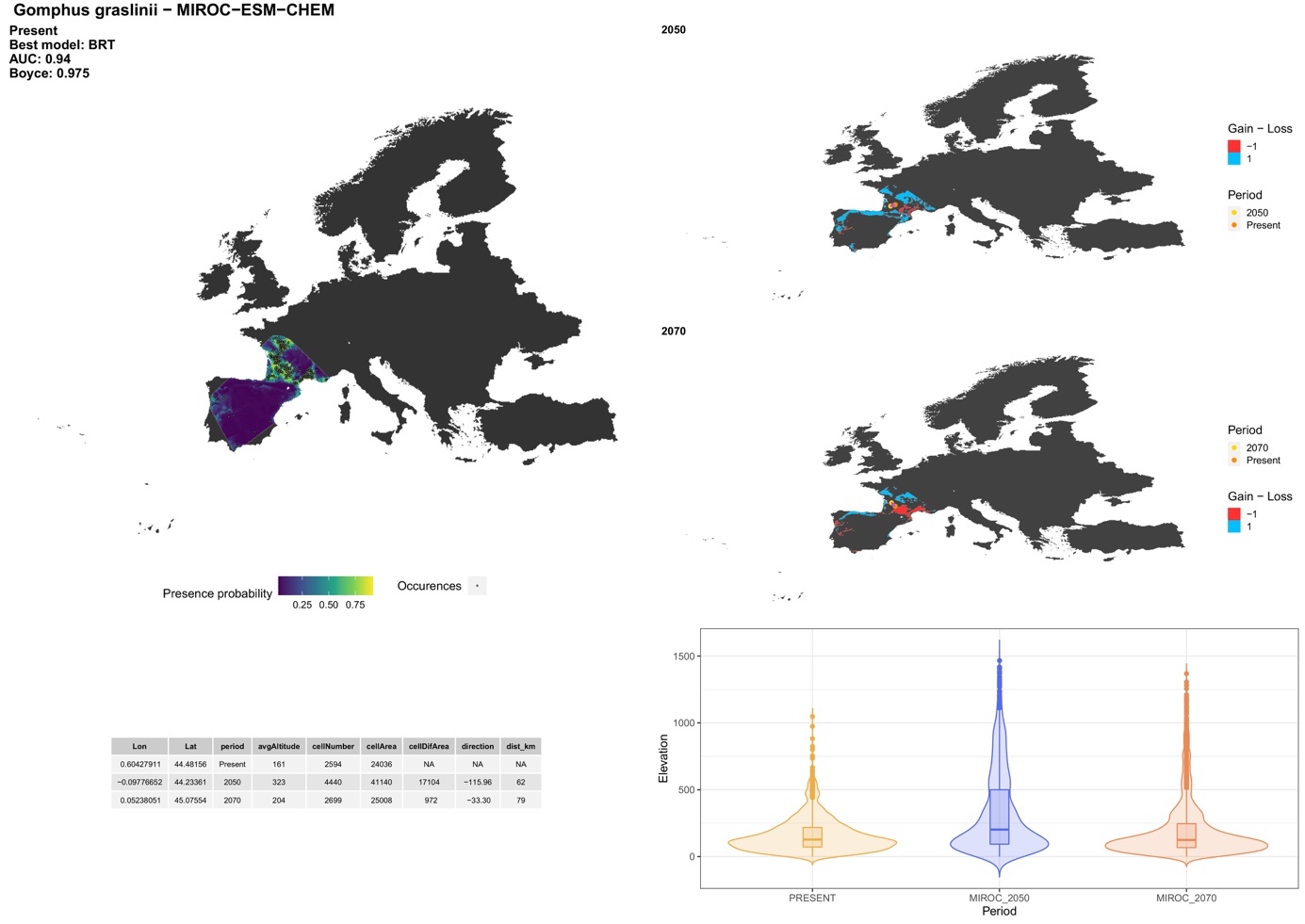


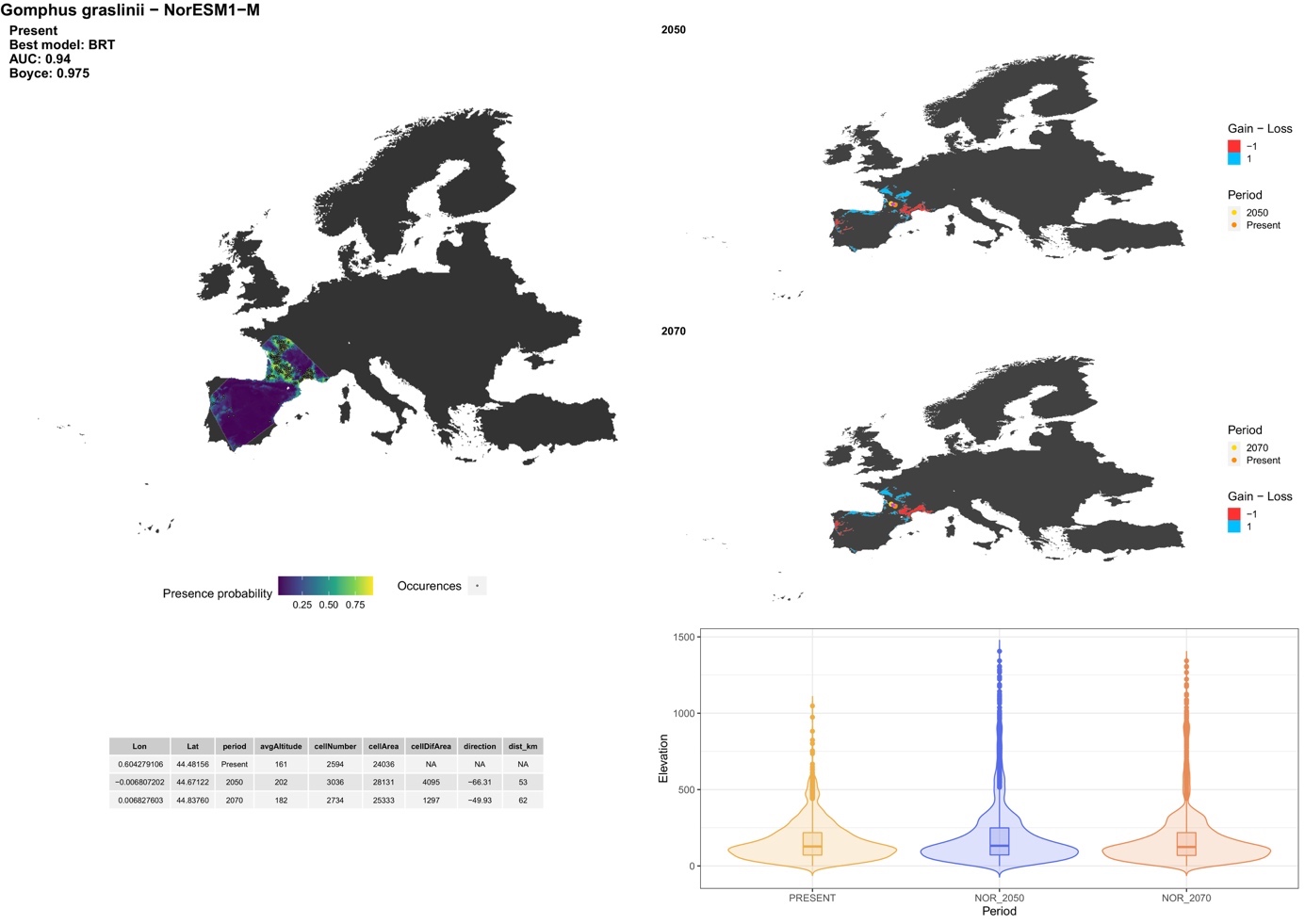


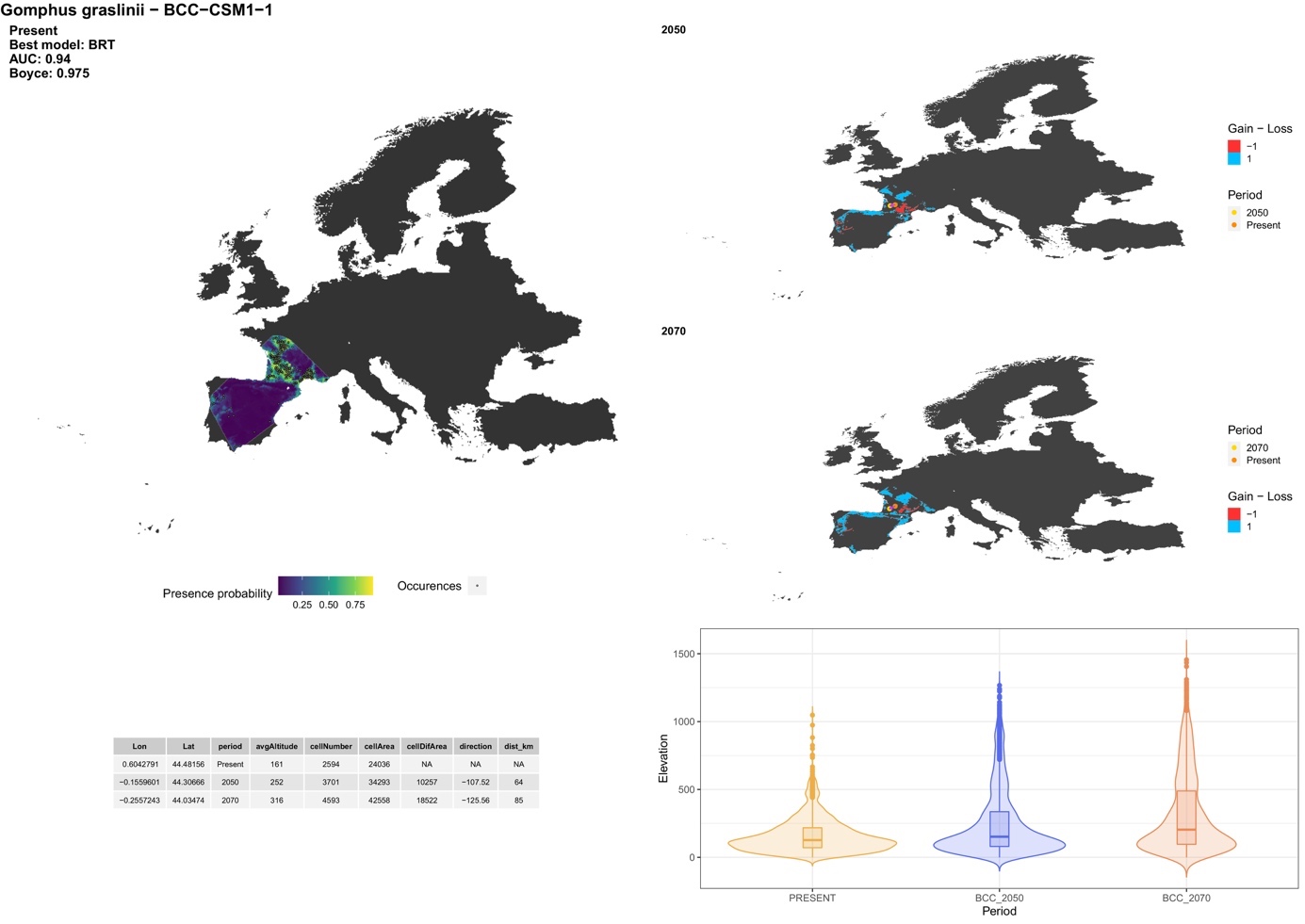


### *Gomphus pulchellus* Selys, 1840


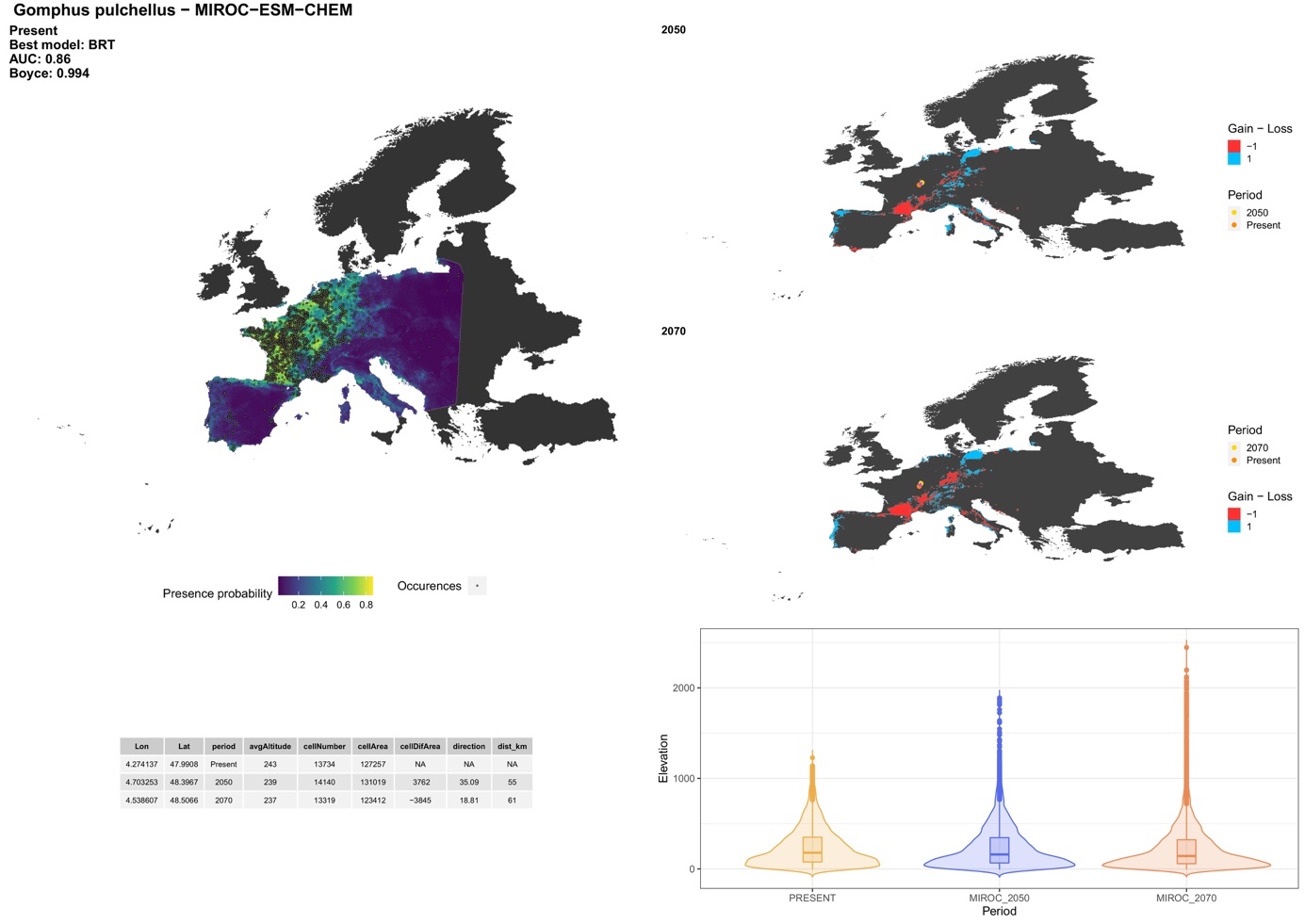


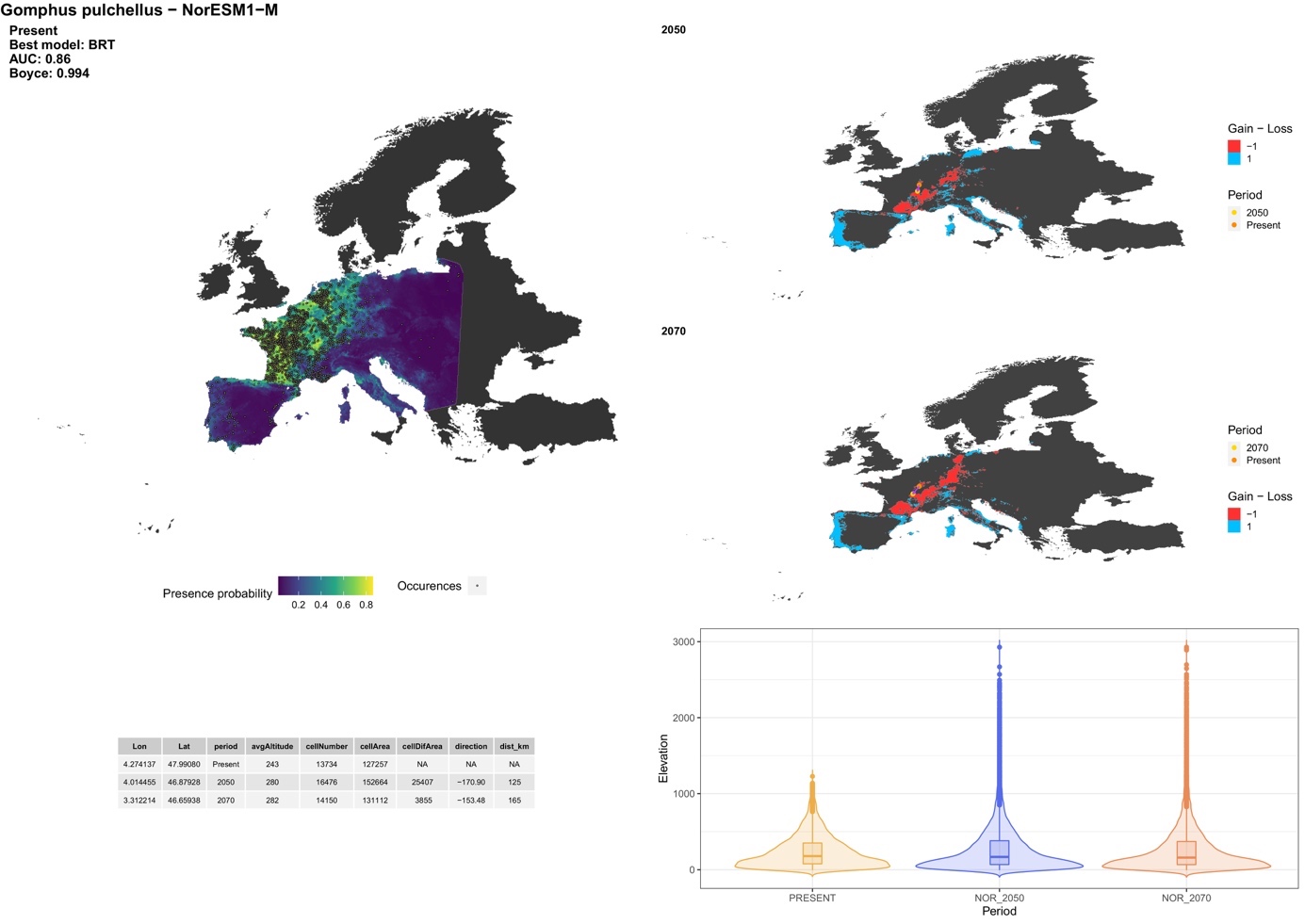


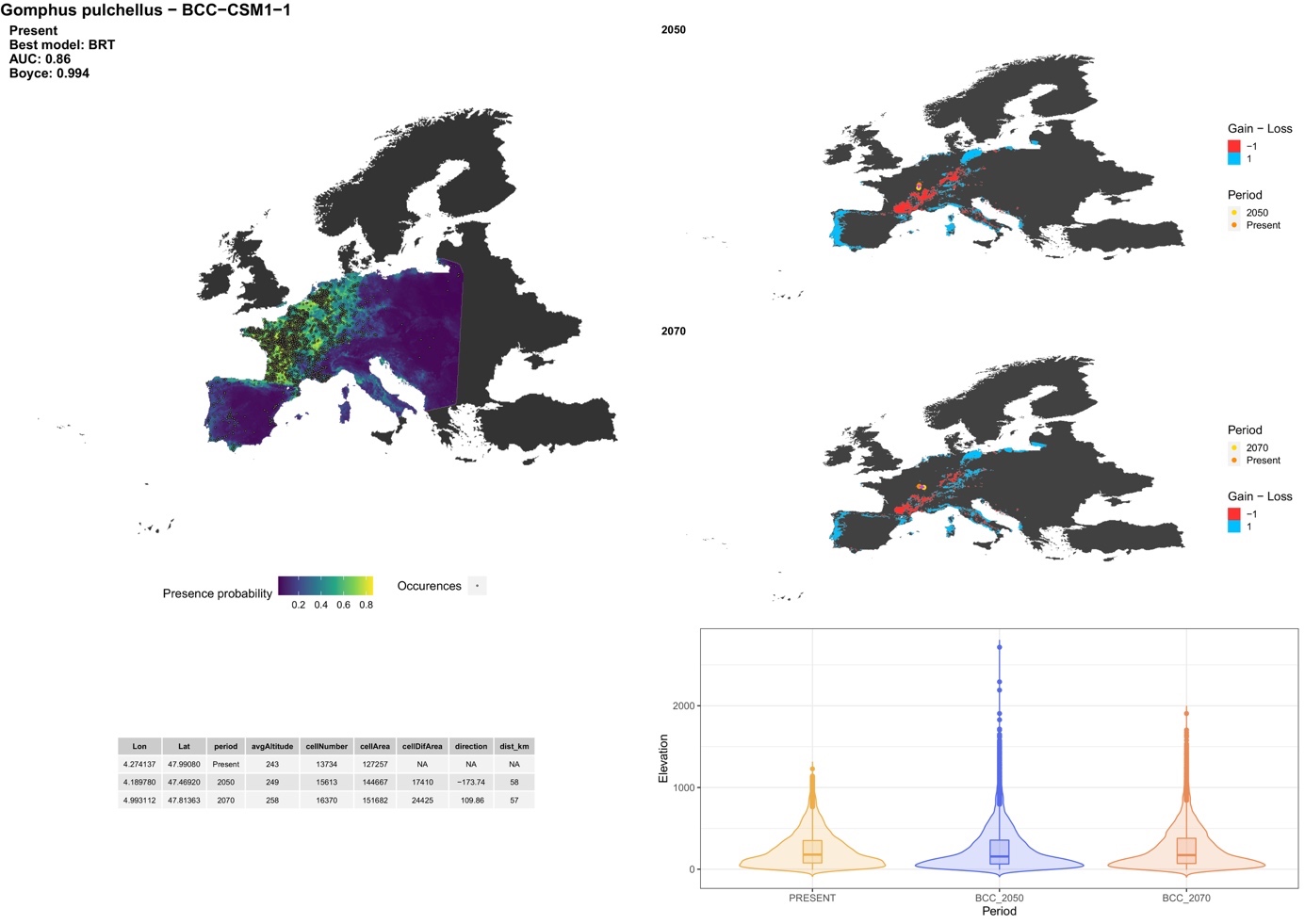


### *Gomphus simillimus* Selys, 1840


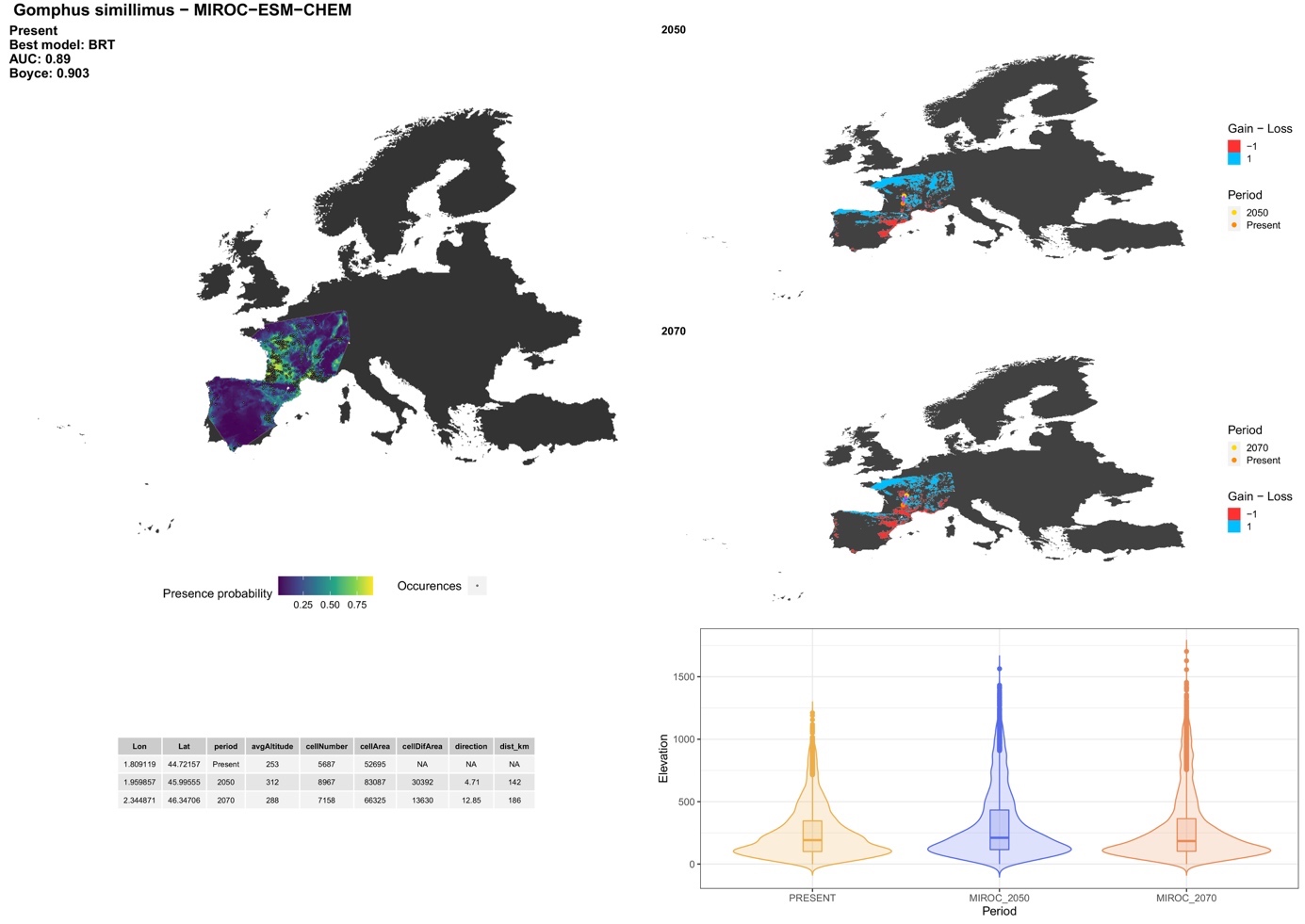


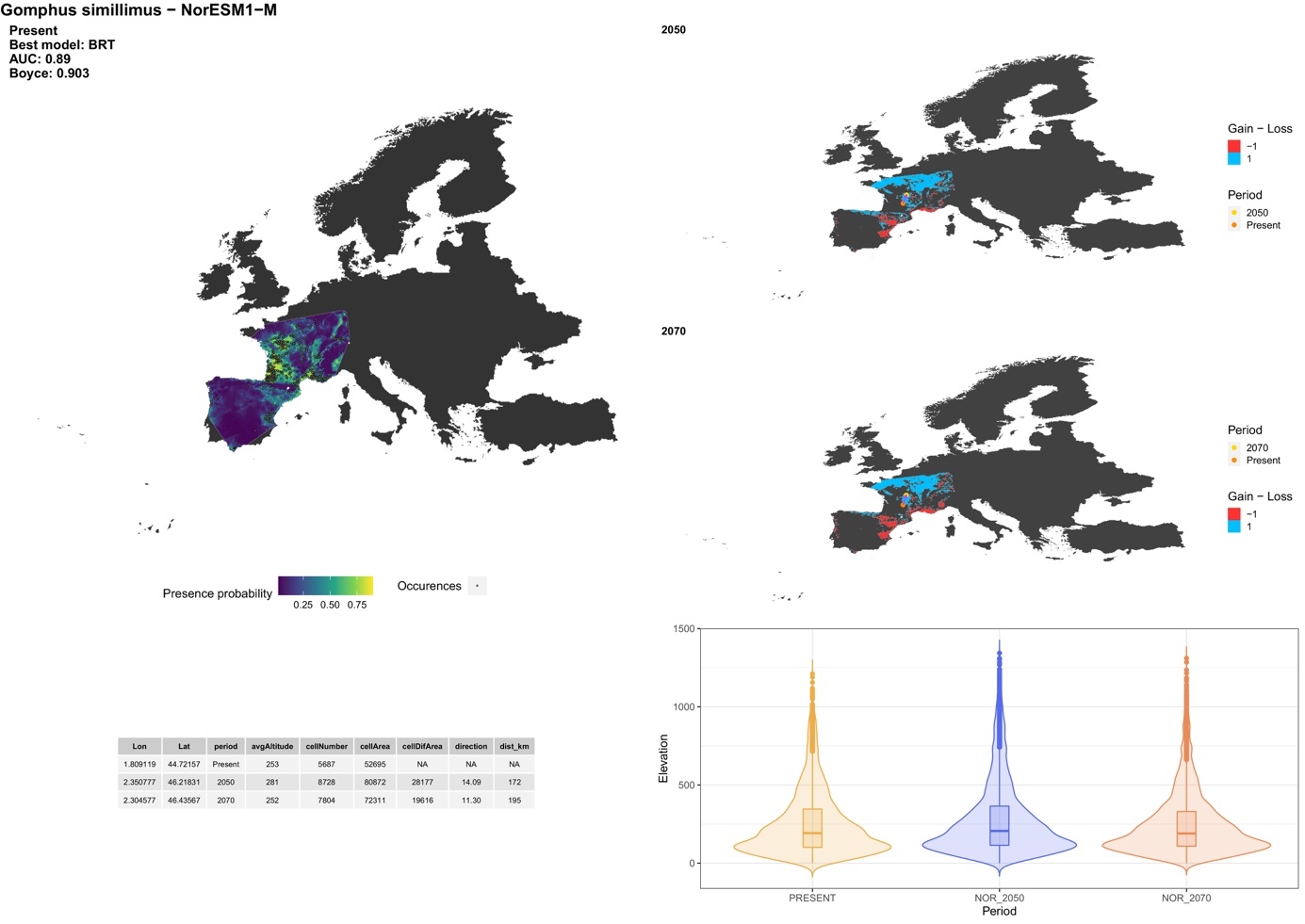


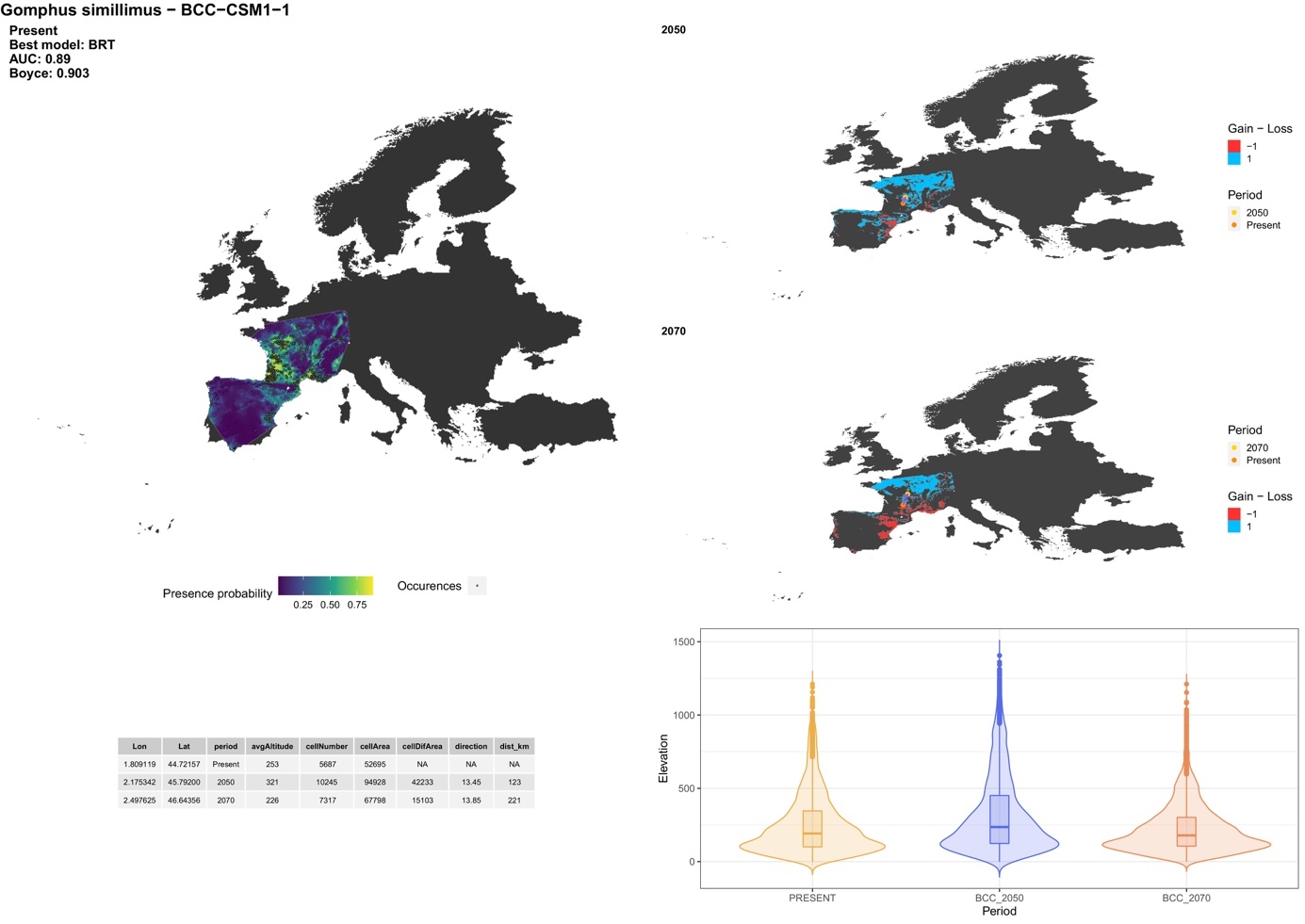


### *Gomphus vulgatissimus* (Linnaeus, 1758)


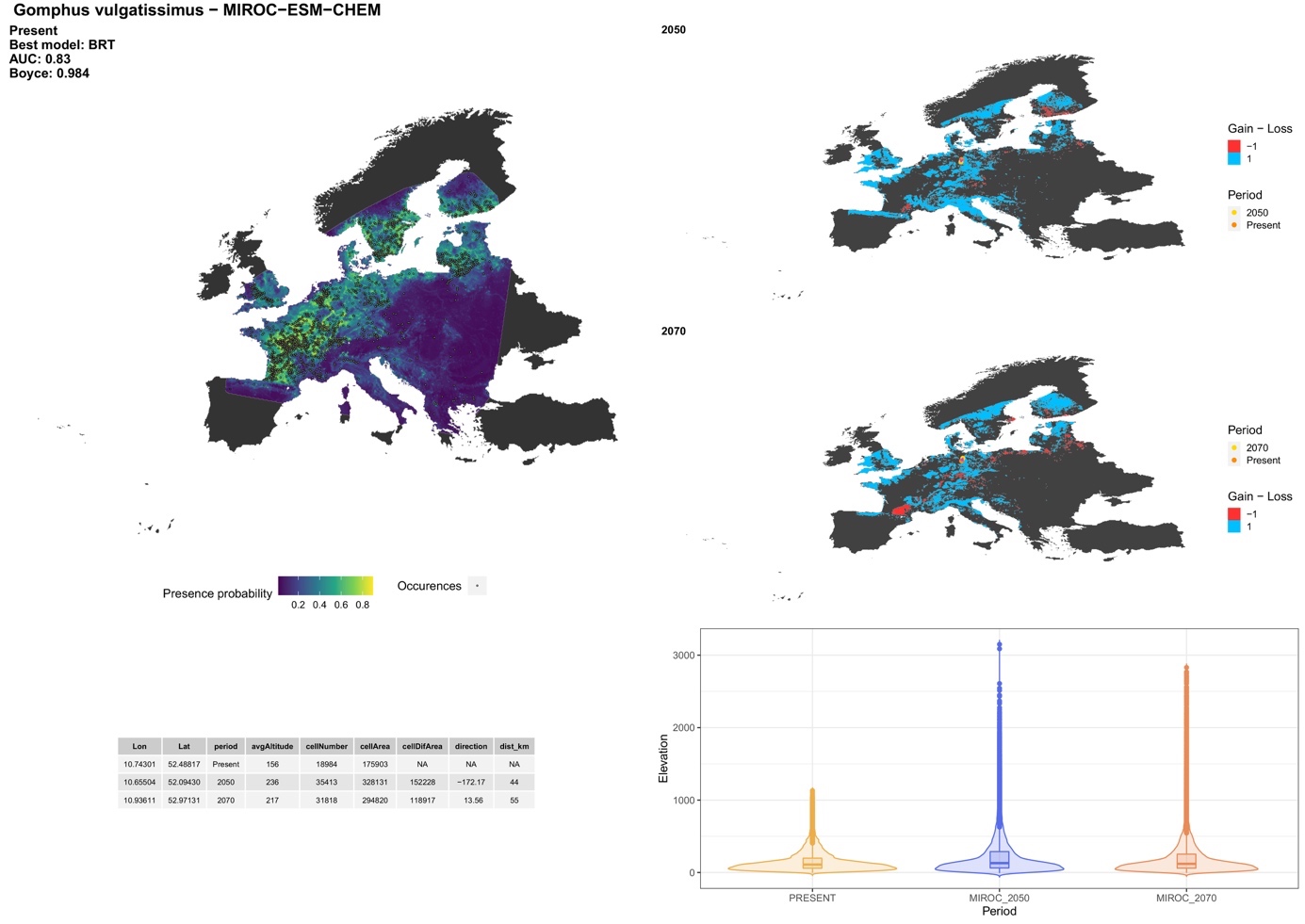


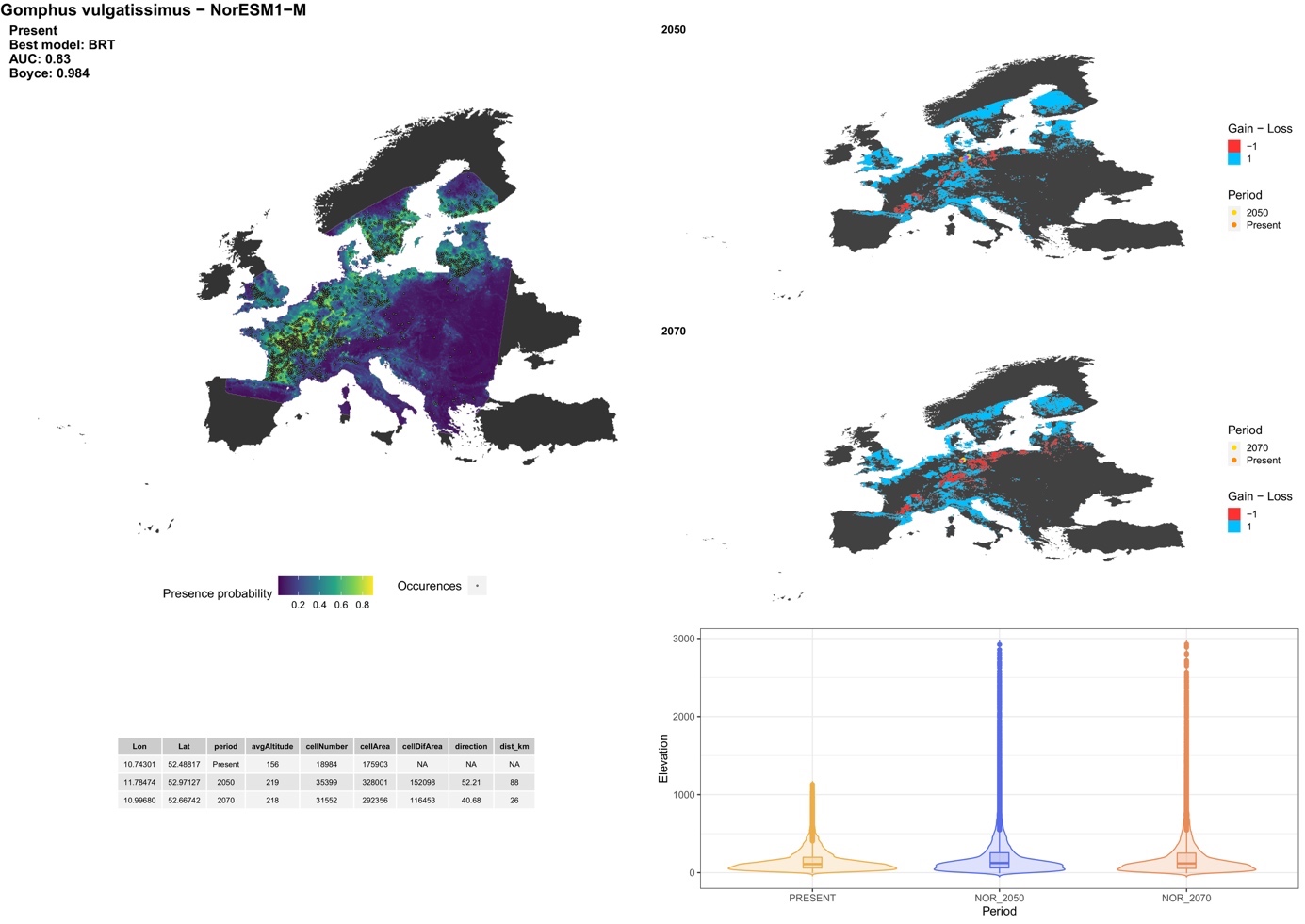


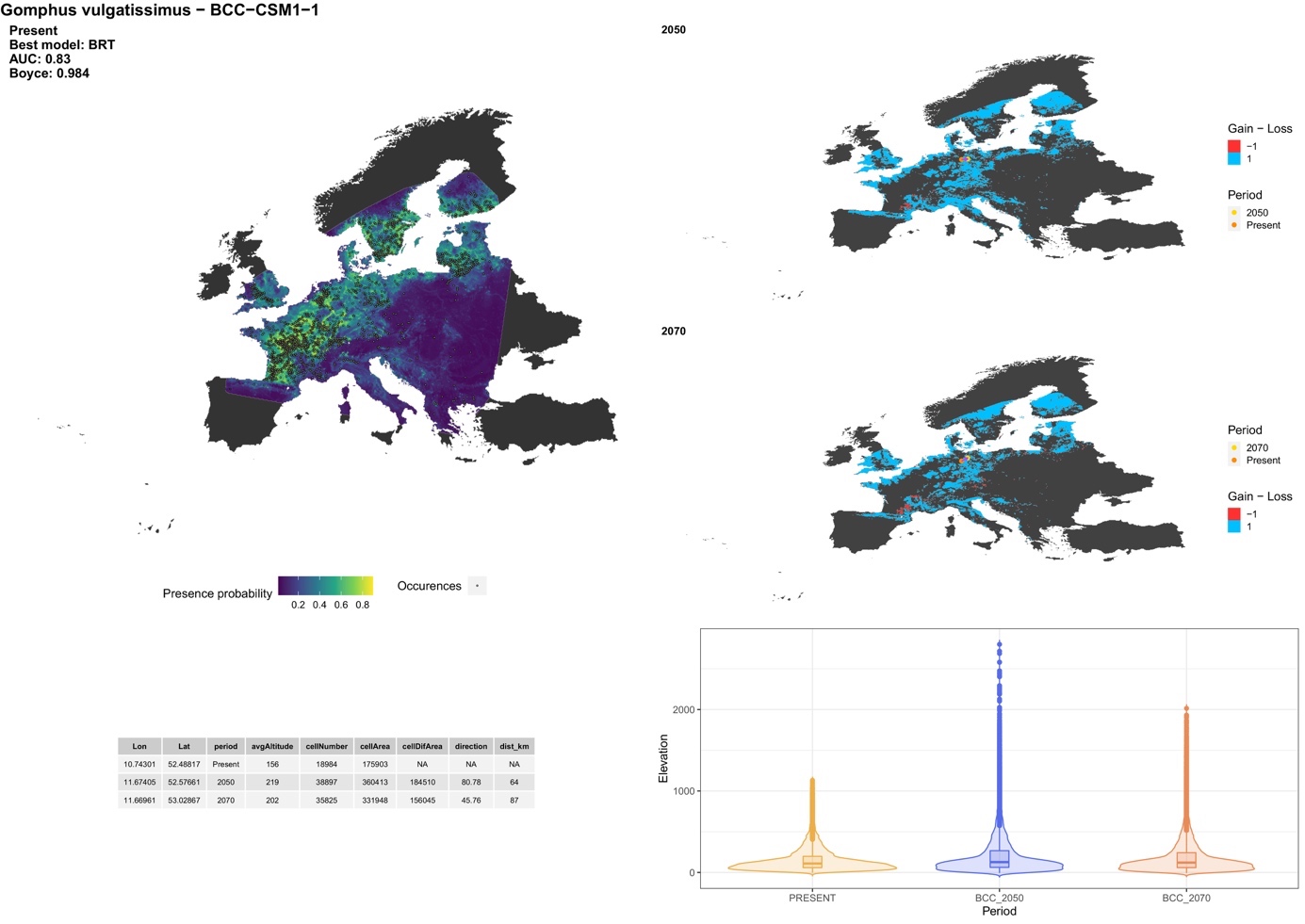


### *Lindenia tetraphylla* (Vander Linden, 1825)


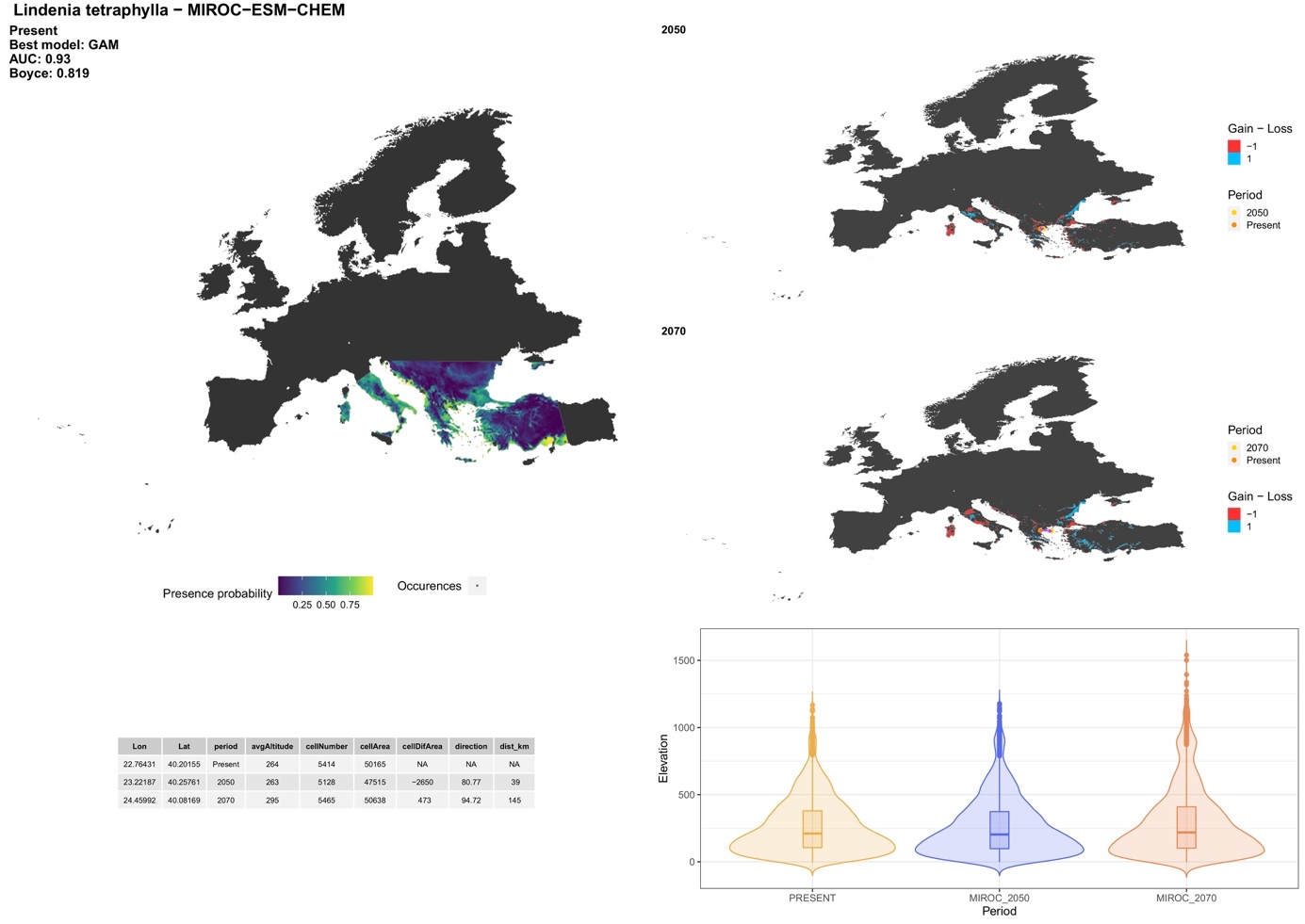


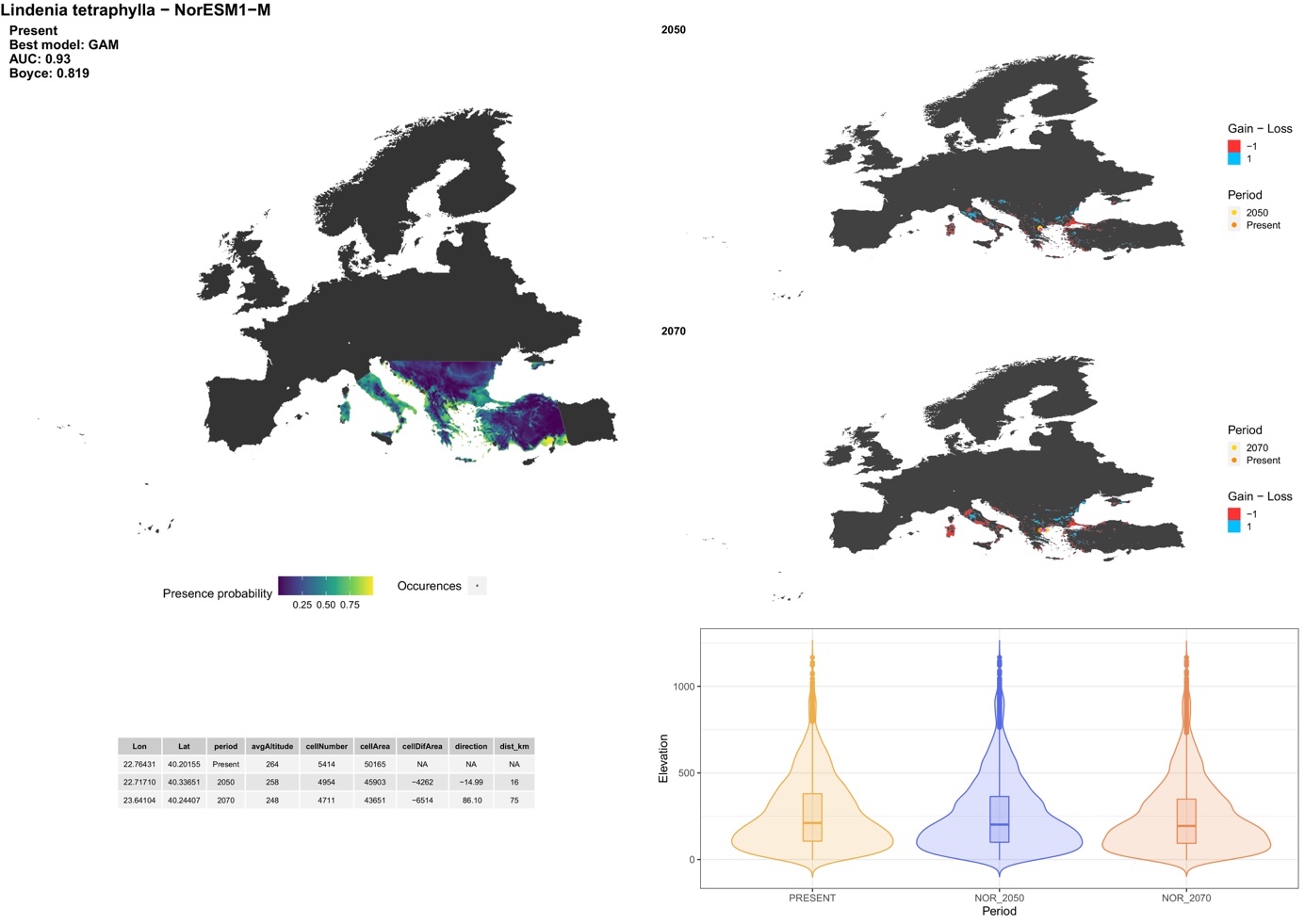


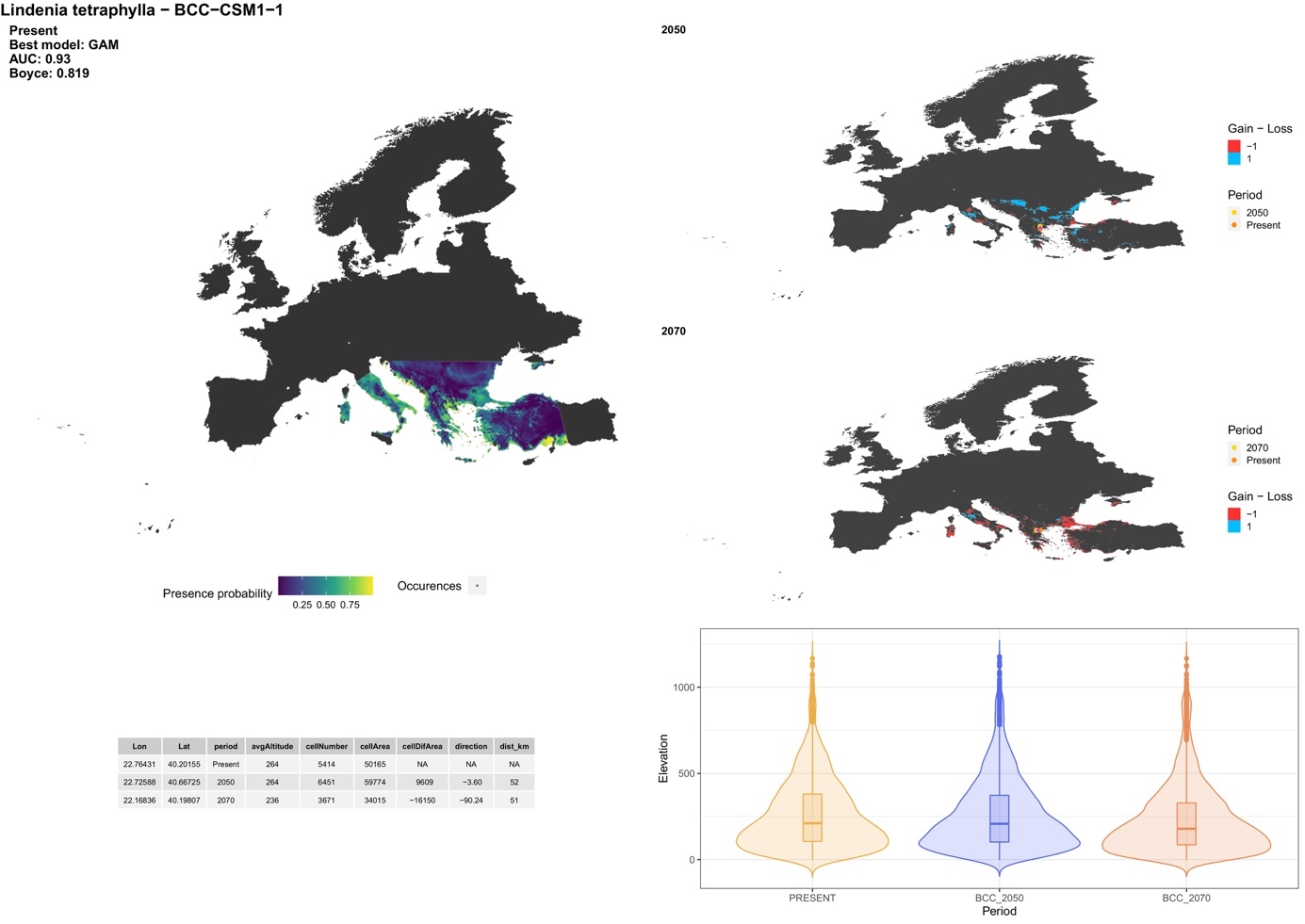


### *Onychogomphus forcipatus* (Linnaeus, 1758)


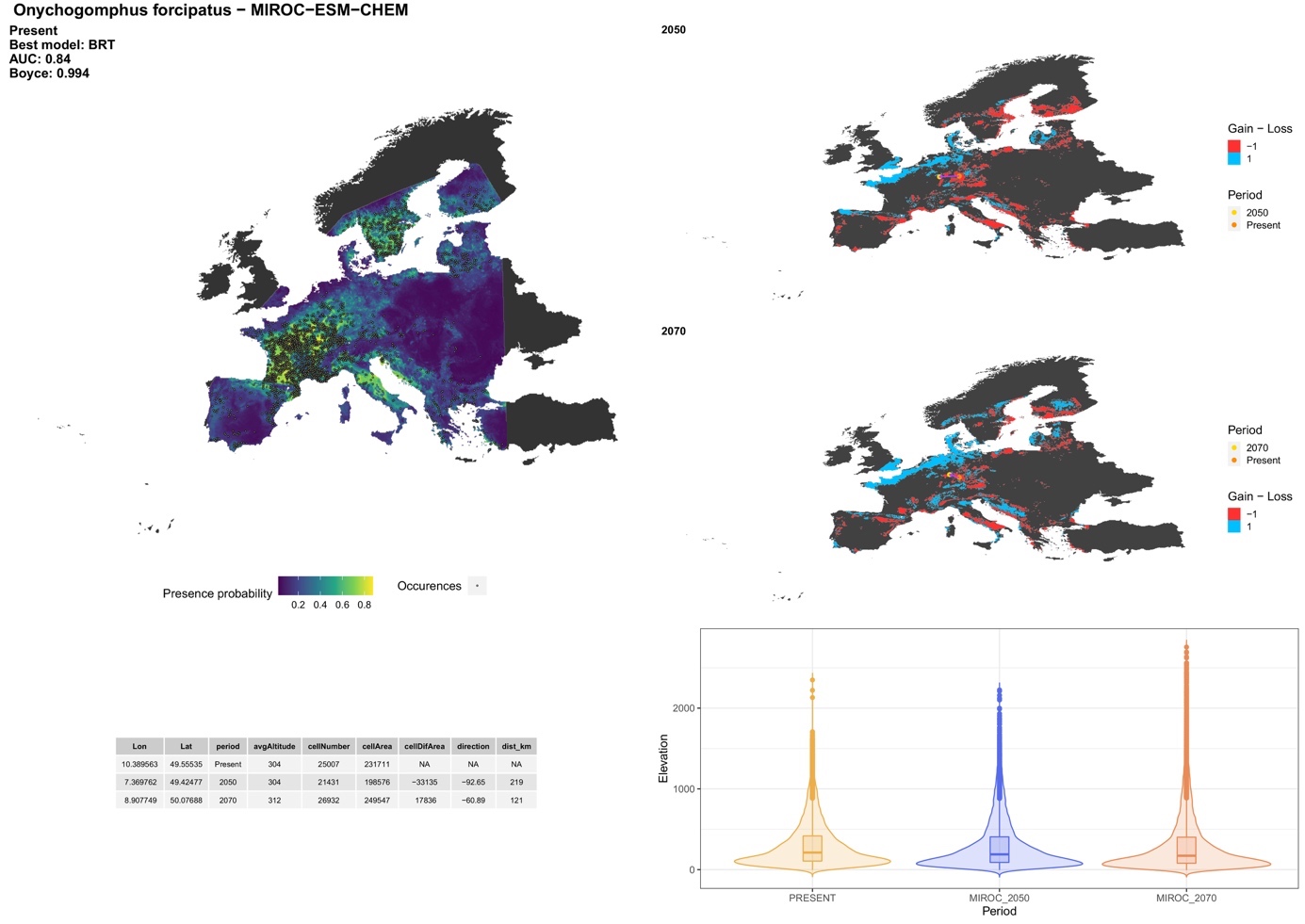


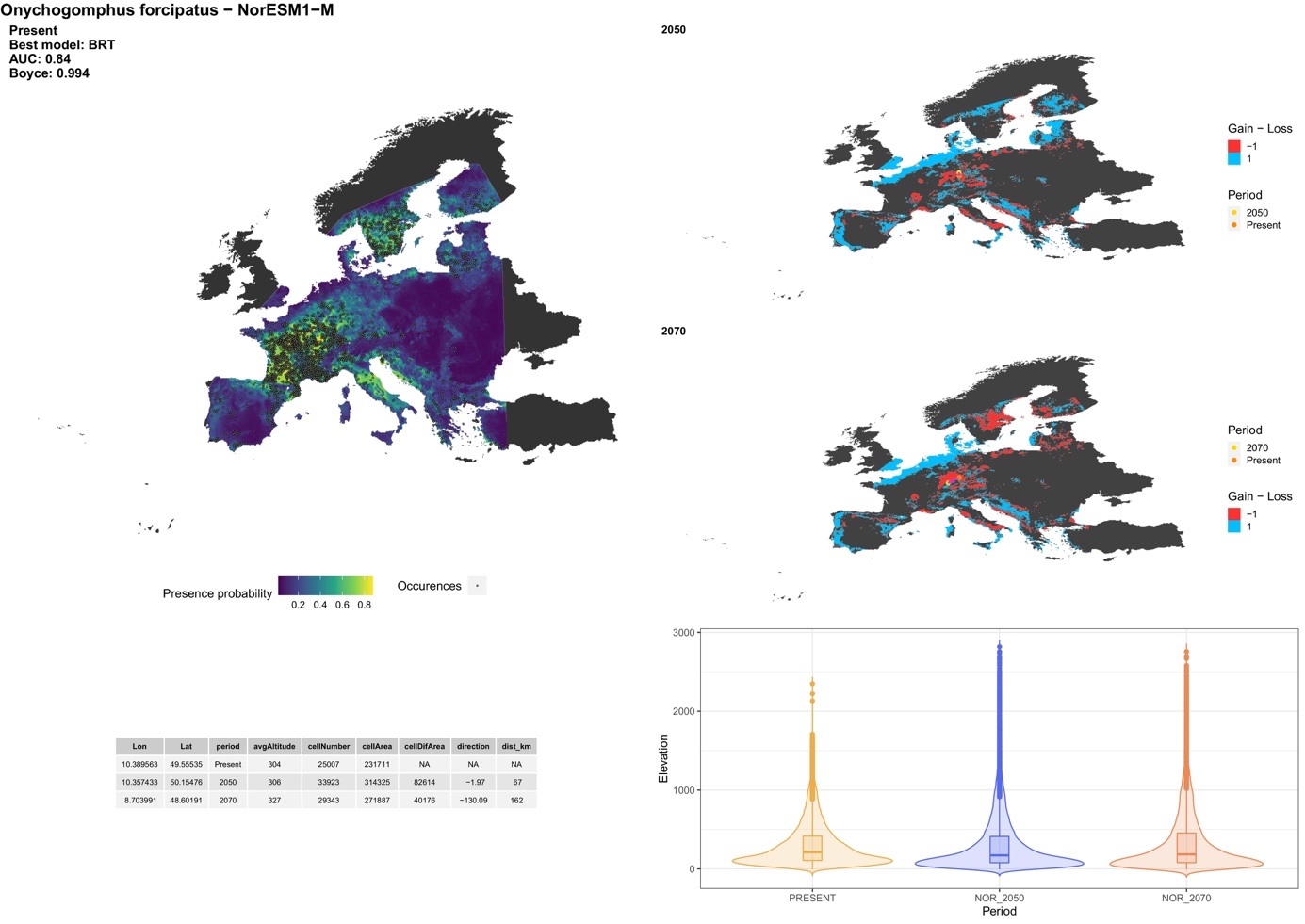


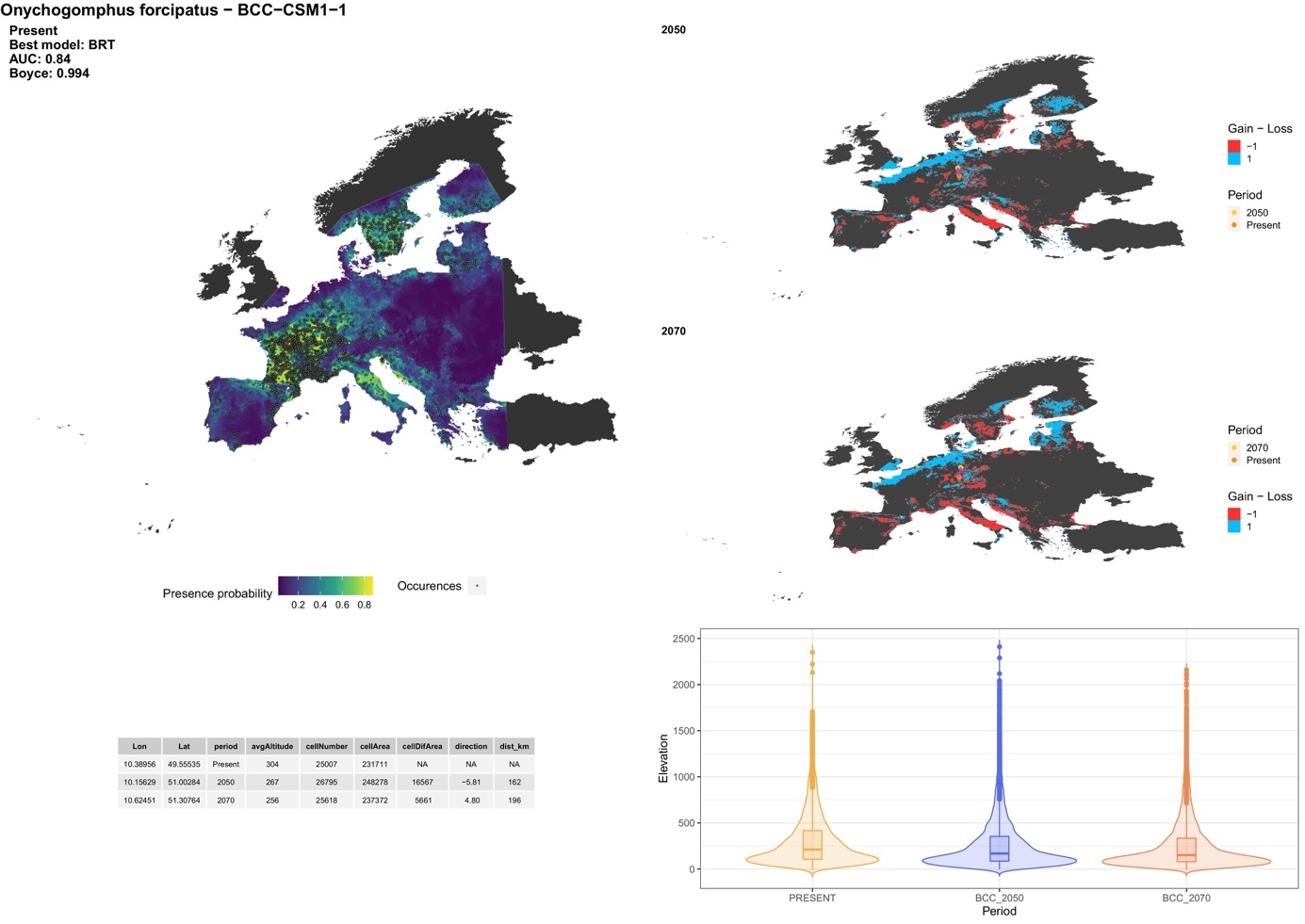


### *Onychogomphus uncatus* (Charpentier, 1840)


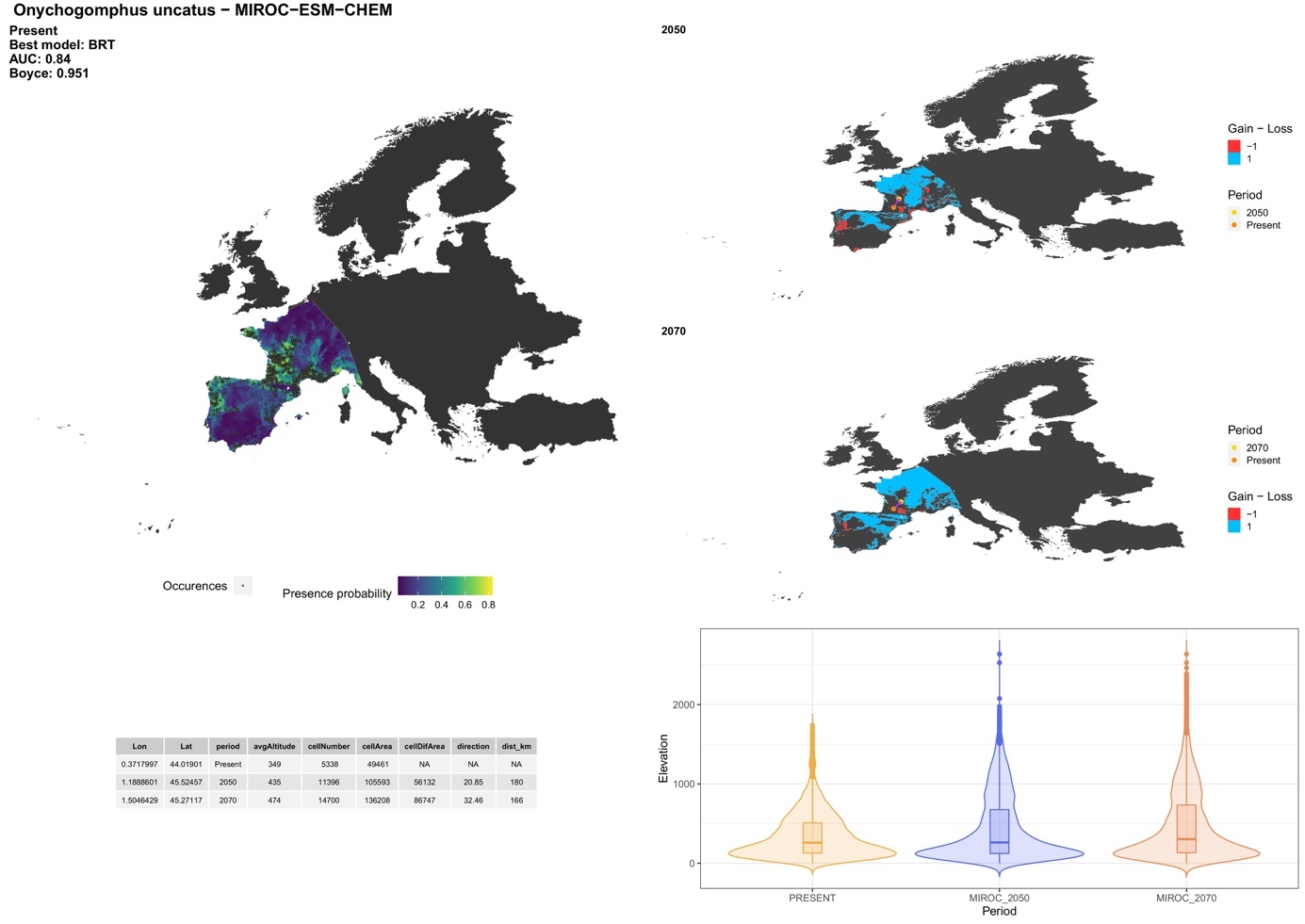


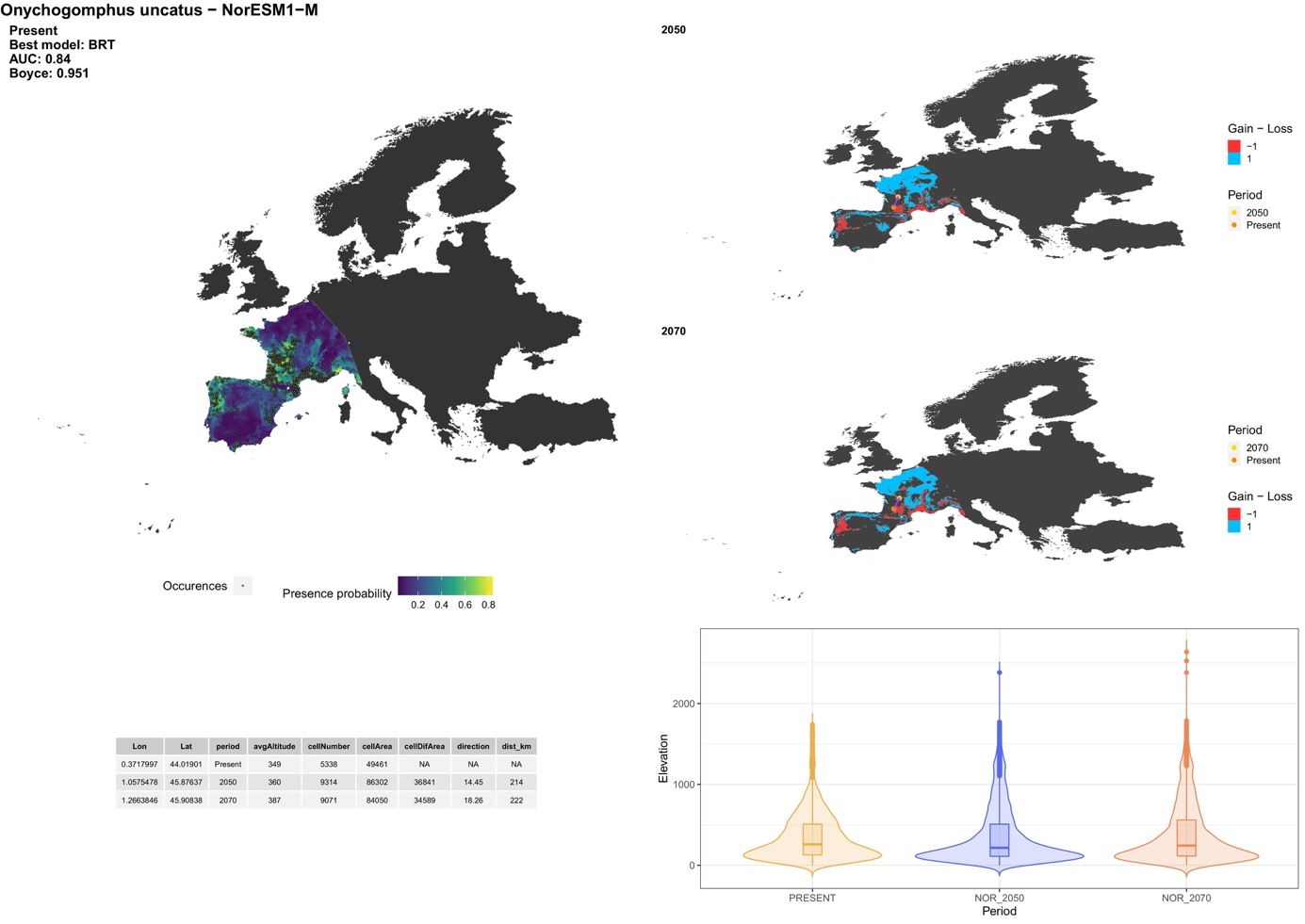


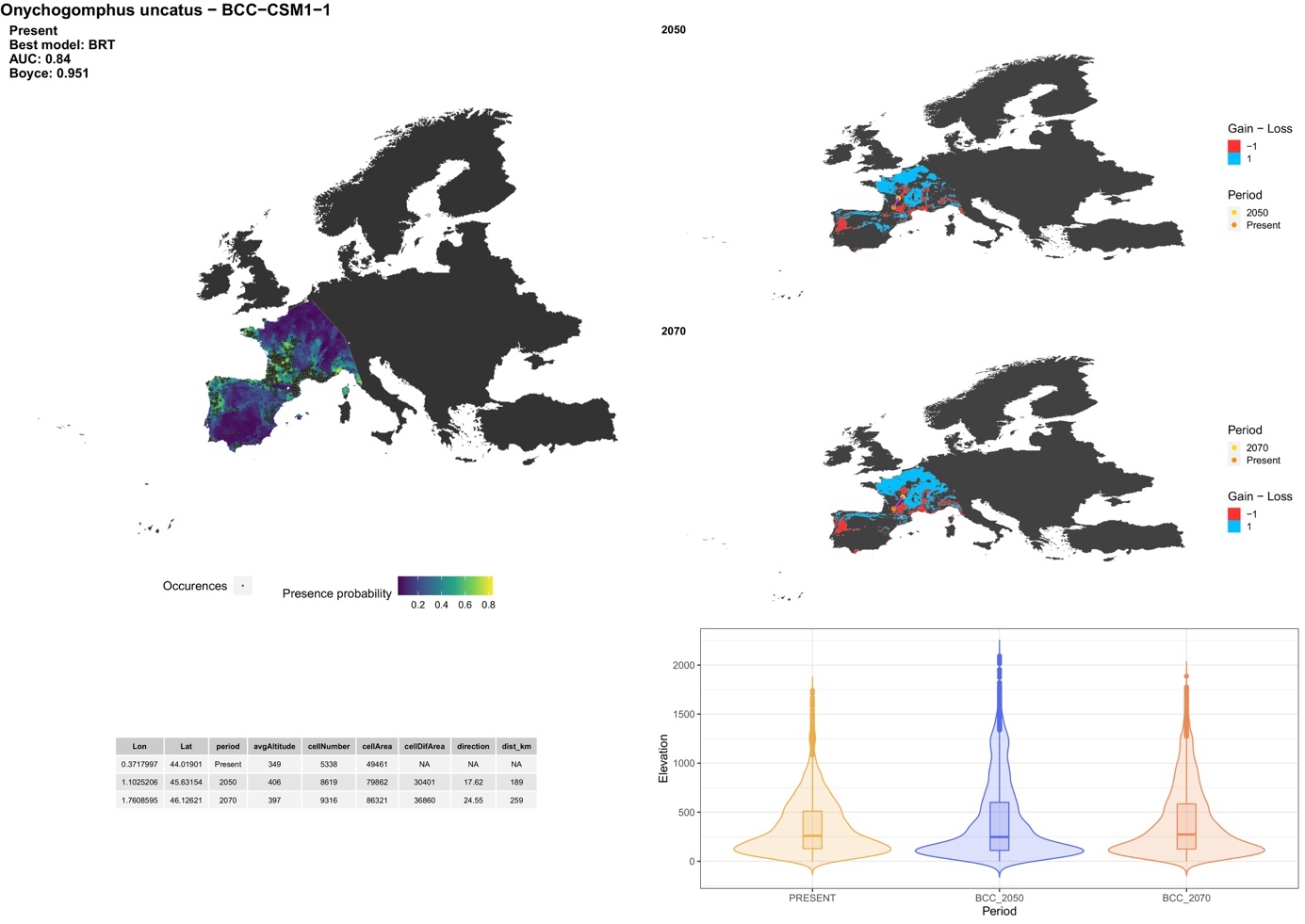


### *Ophiogomphus cecilia* (Geoffroy in Fourcroy, 1785)


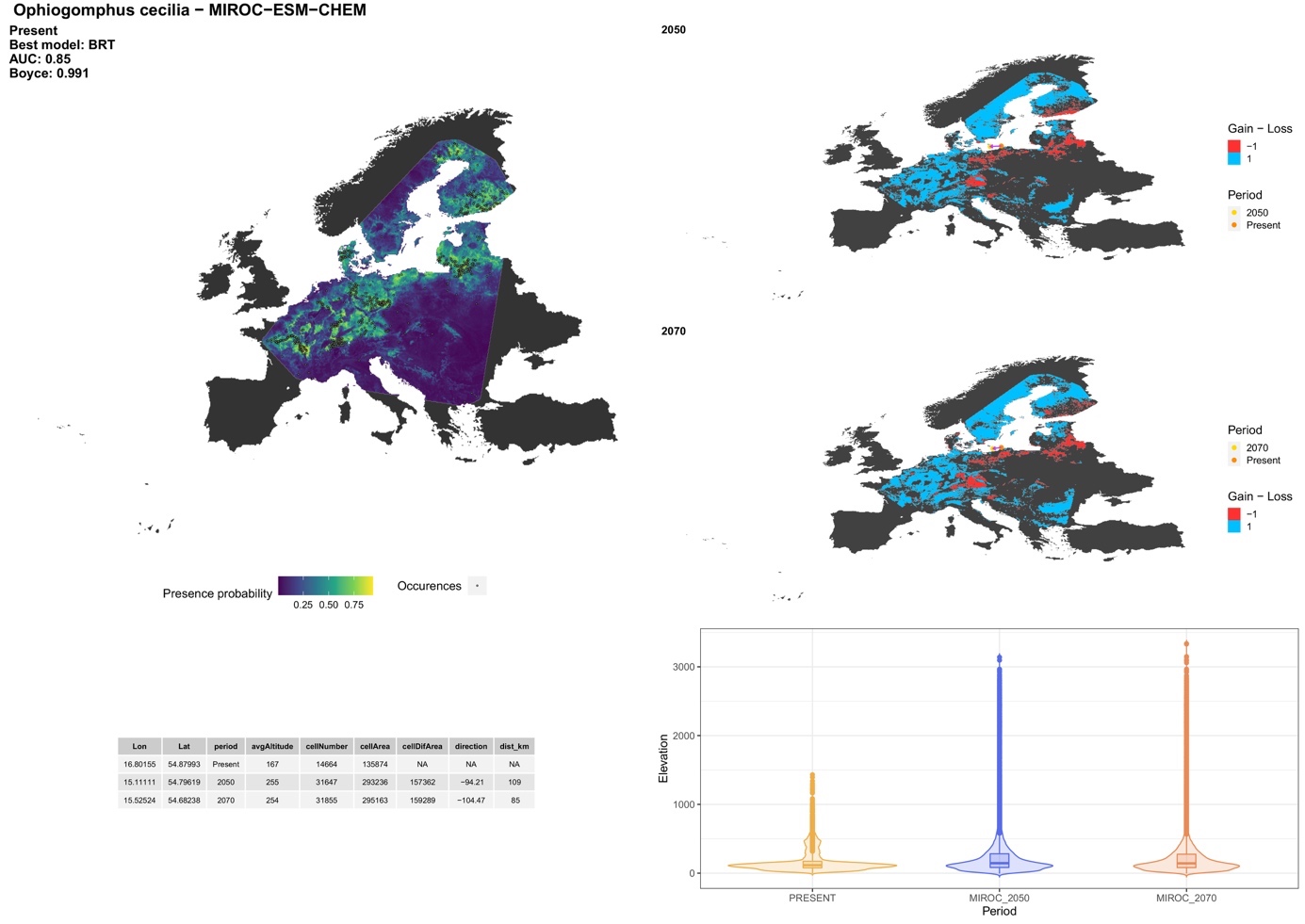


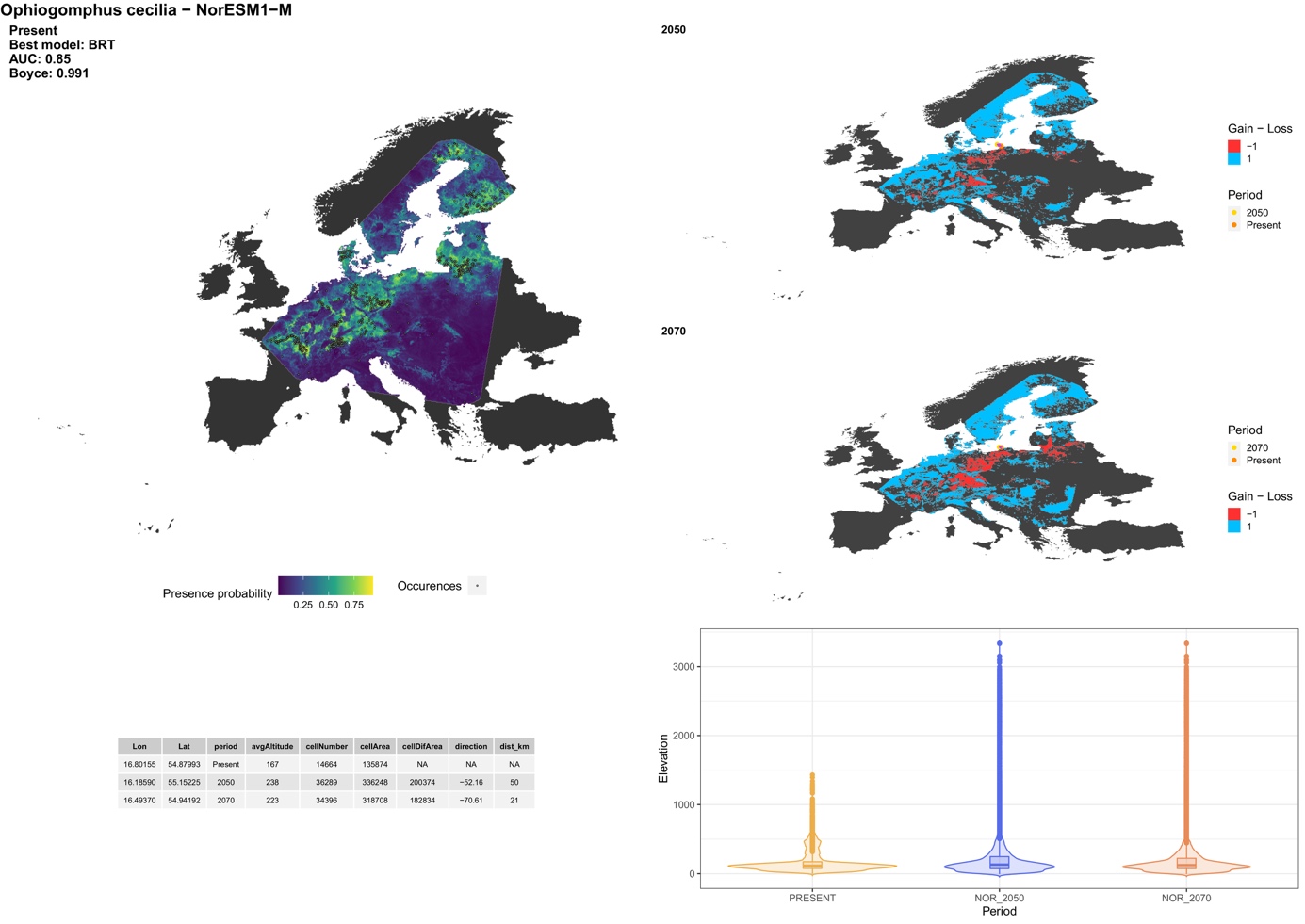


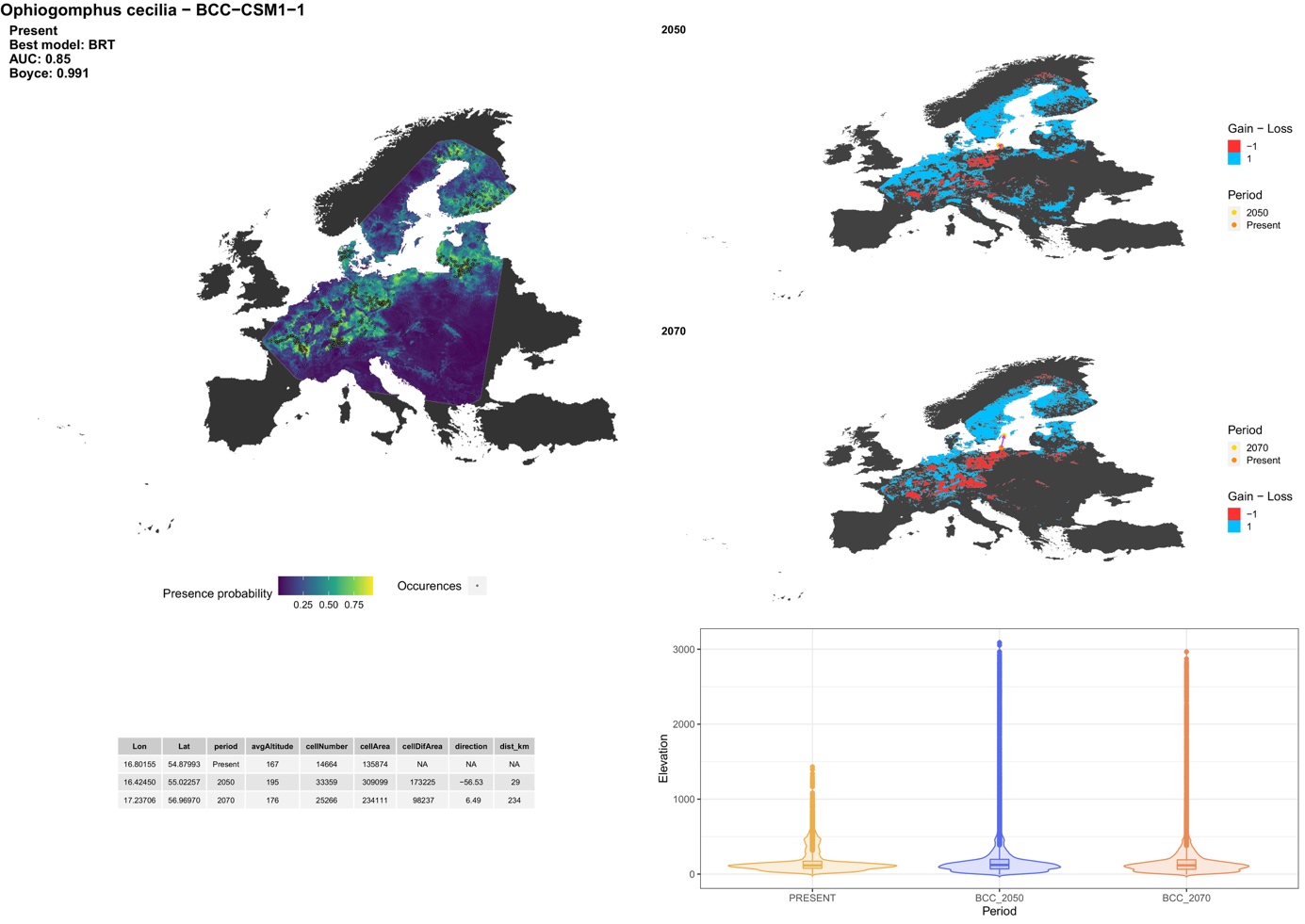


### *Stylurus flavipes* (Charpentier, 1825)


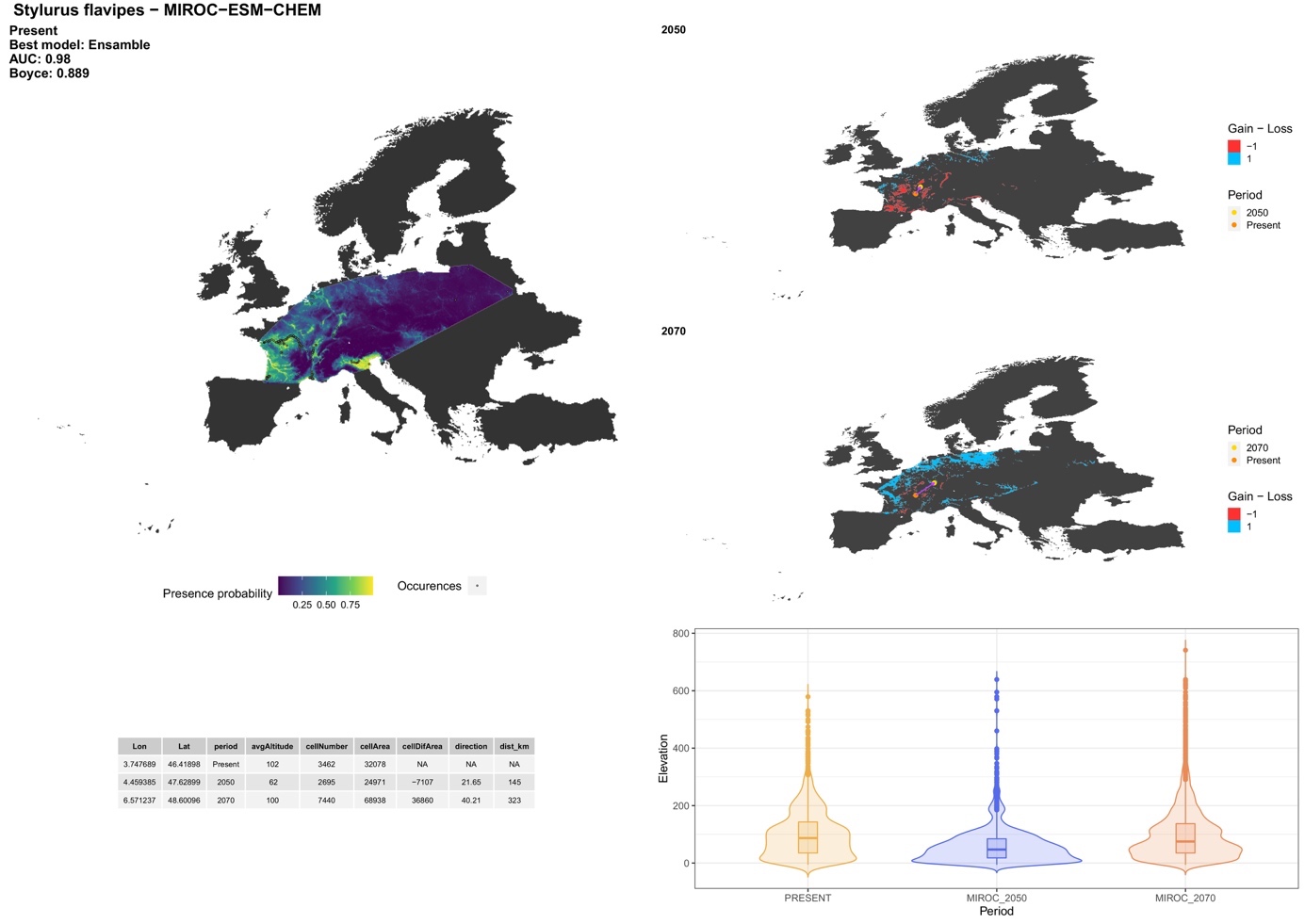


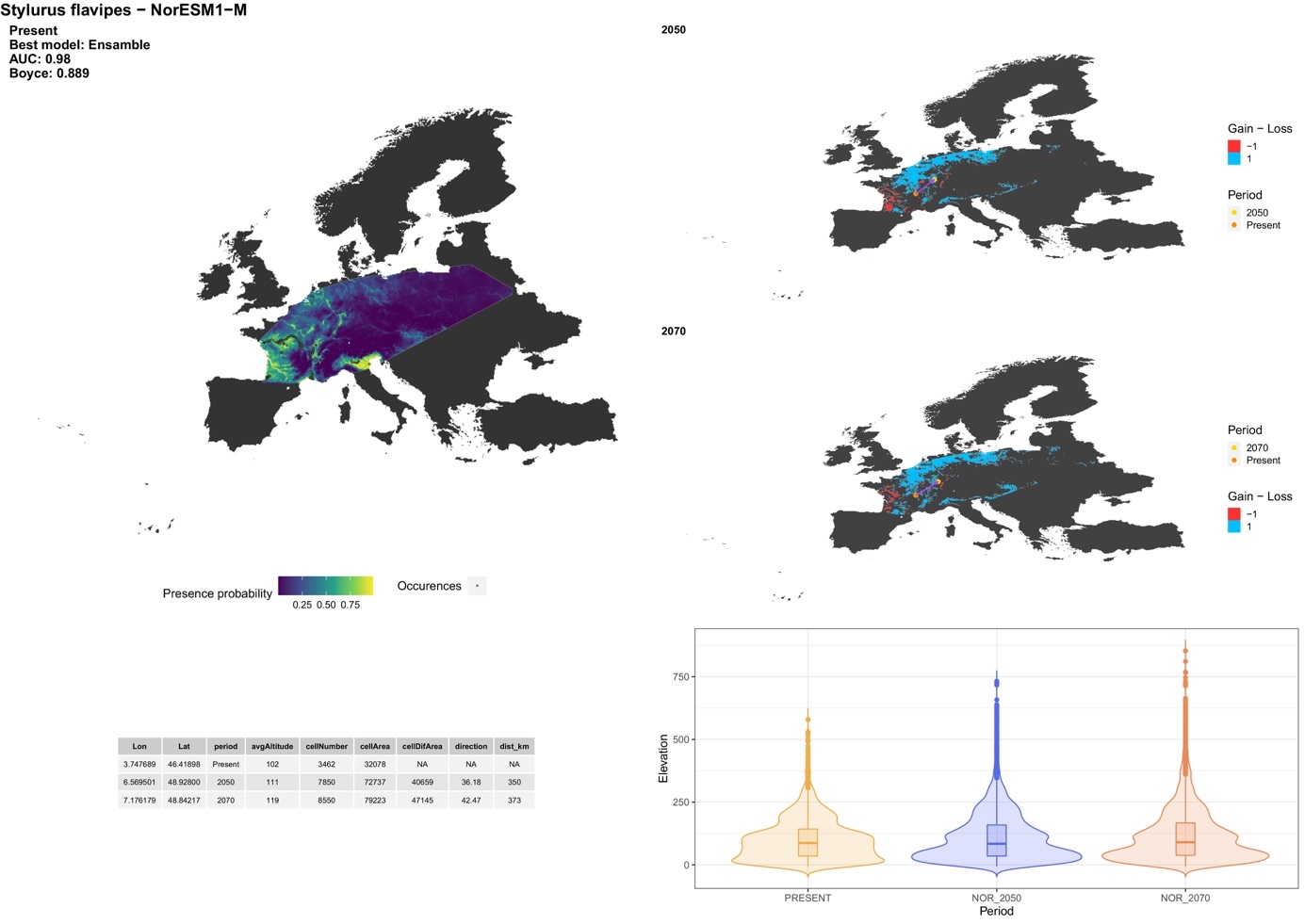


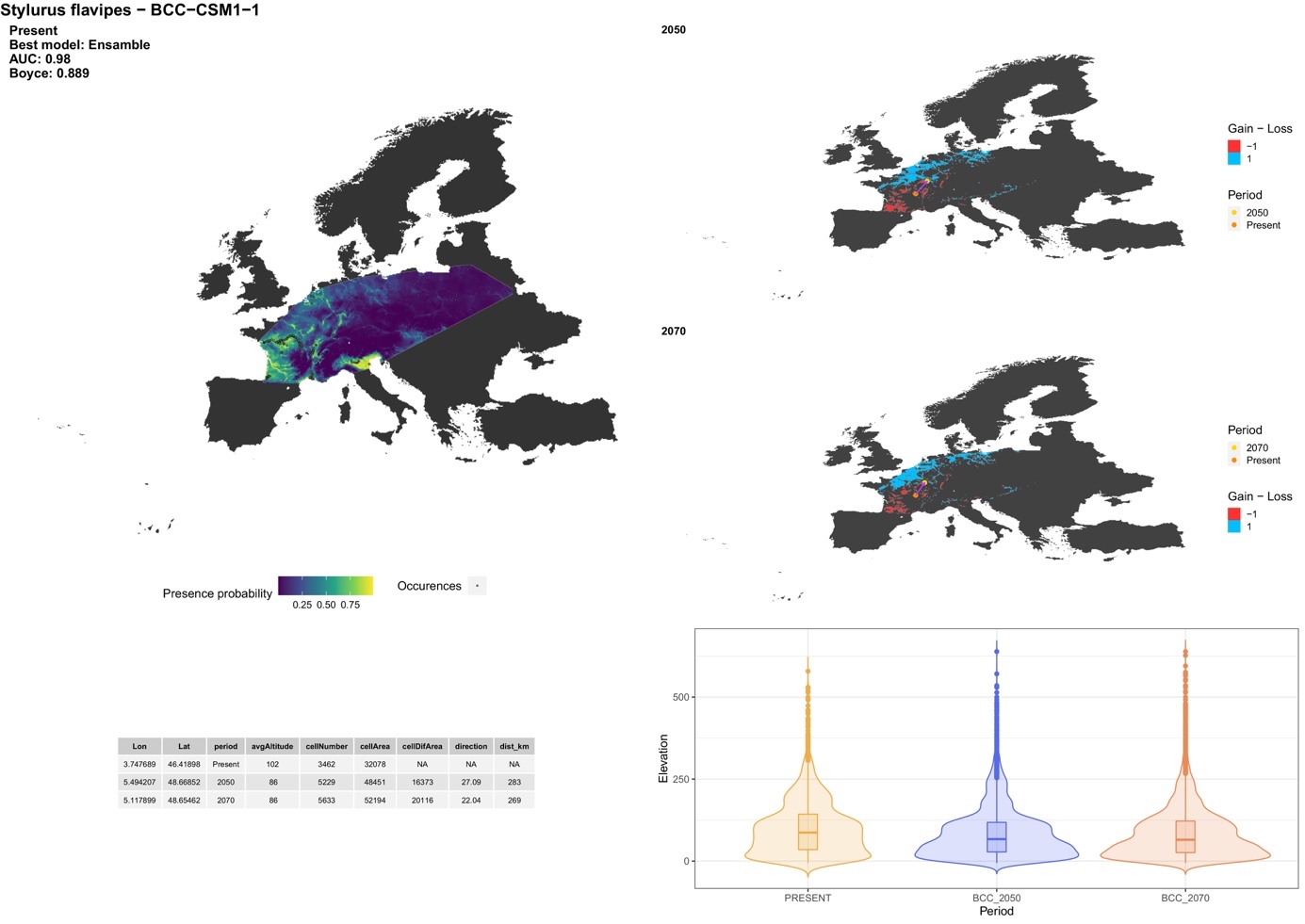


### **Family: Cordulegastridae**

### *Cordulegaster bidentata* Selys, 1843


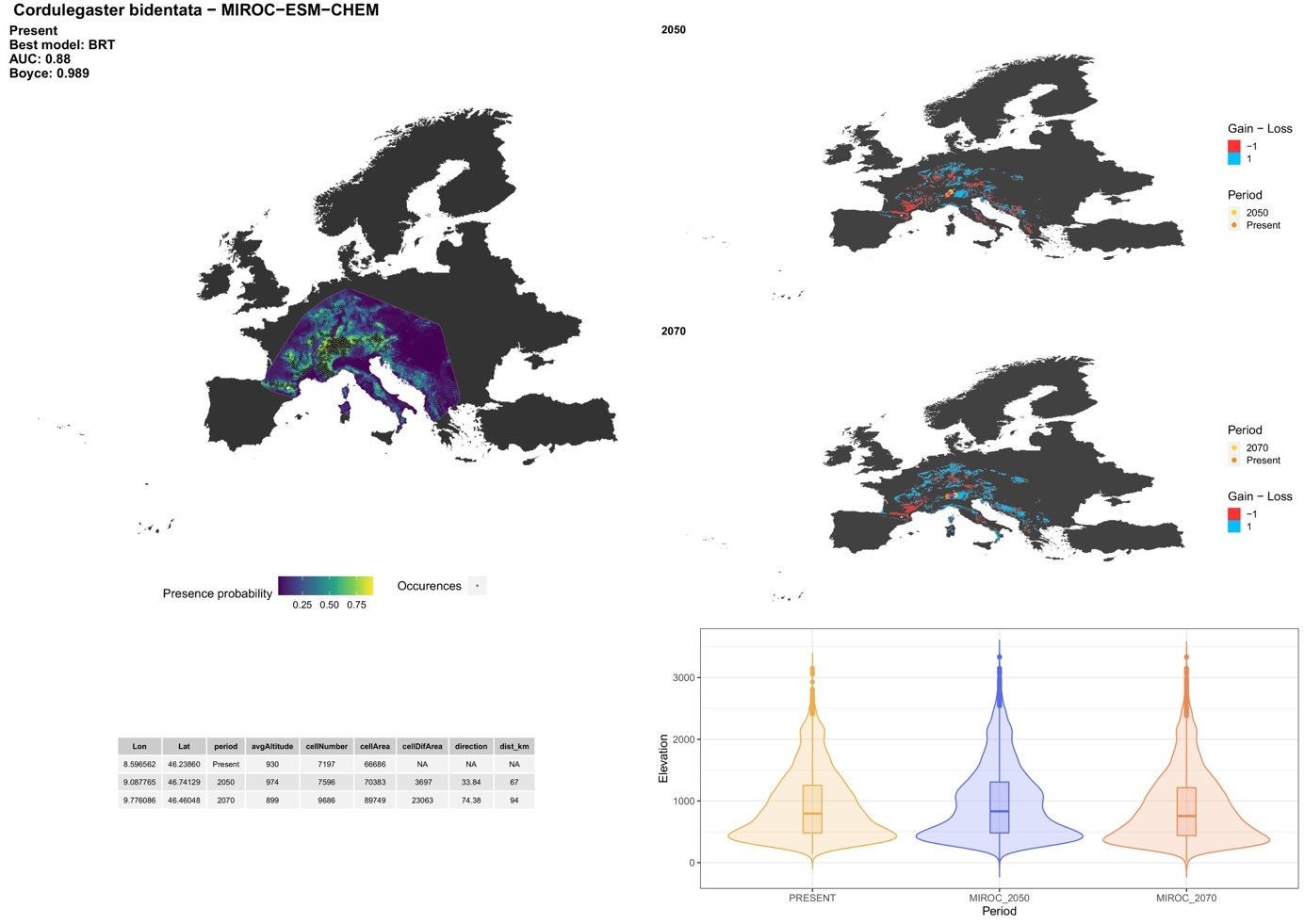


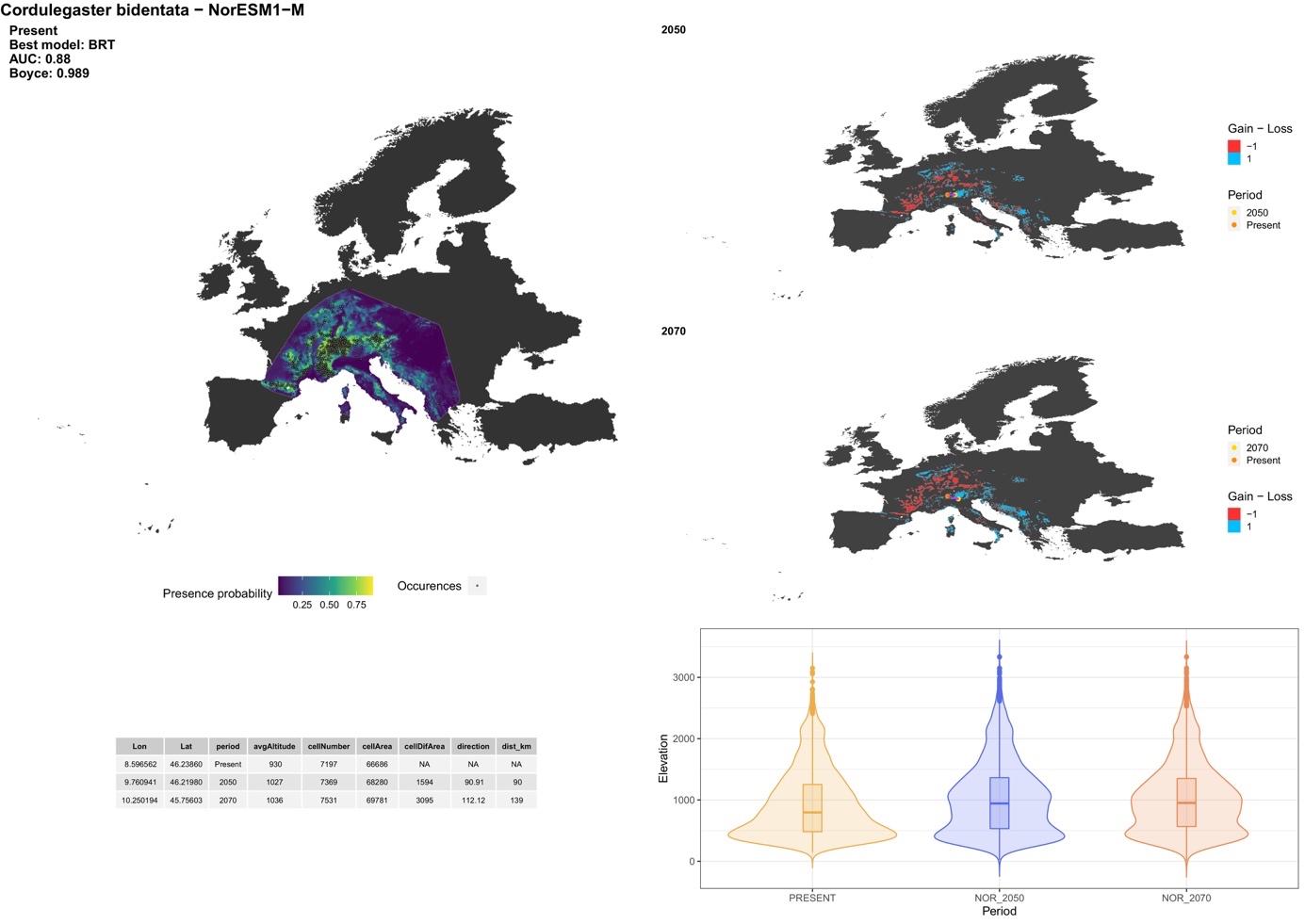


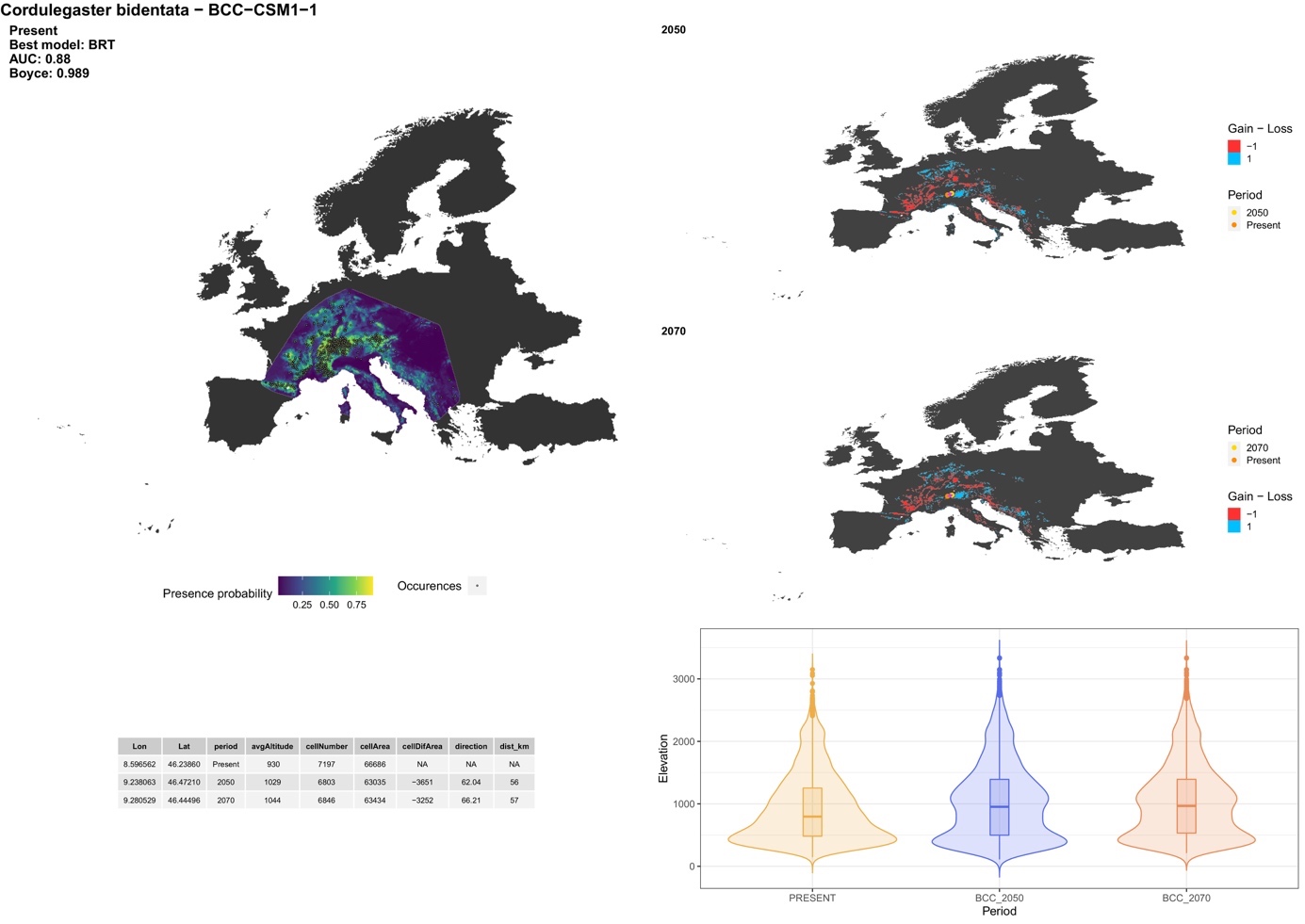


### *Cordulegaster boltonii* (Donovan, 1807)


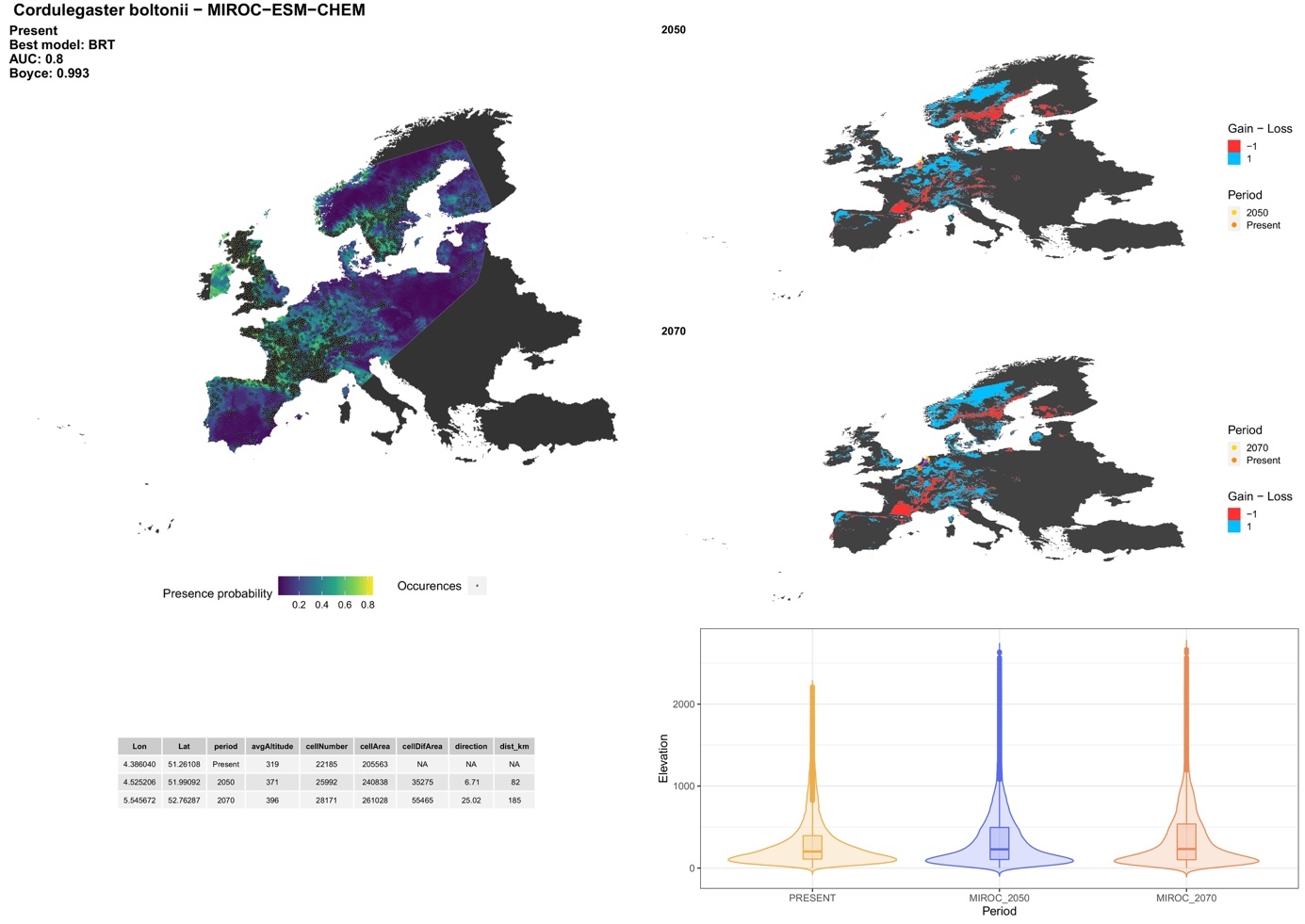


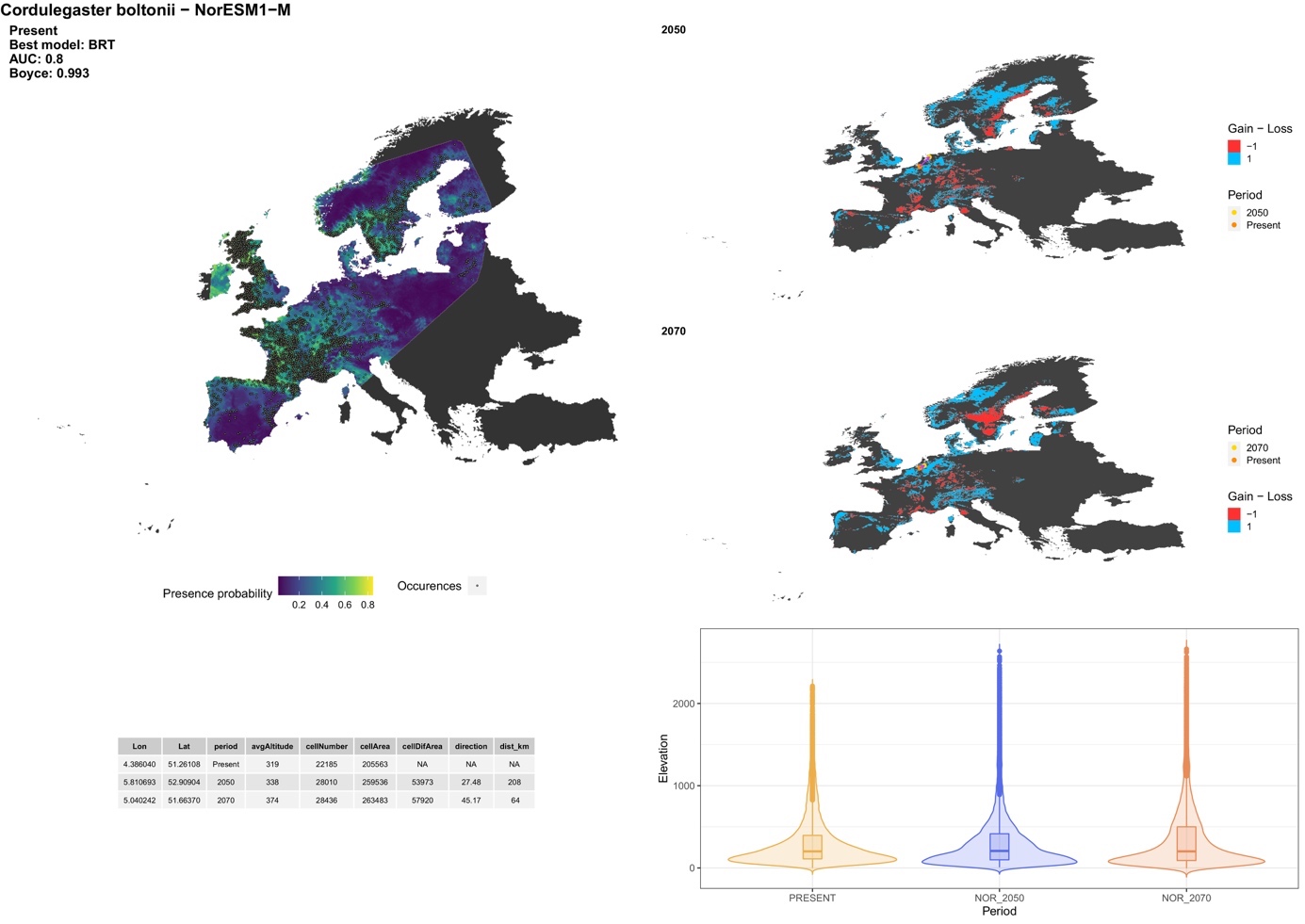


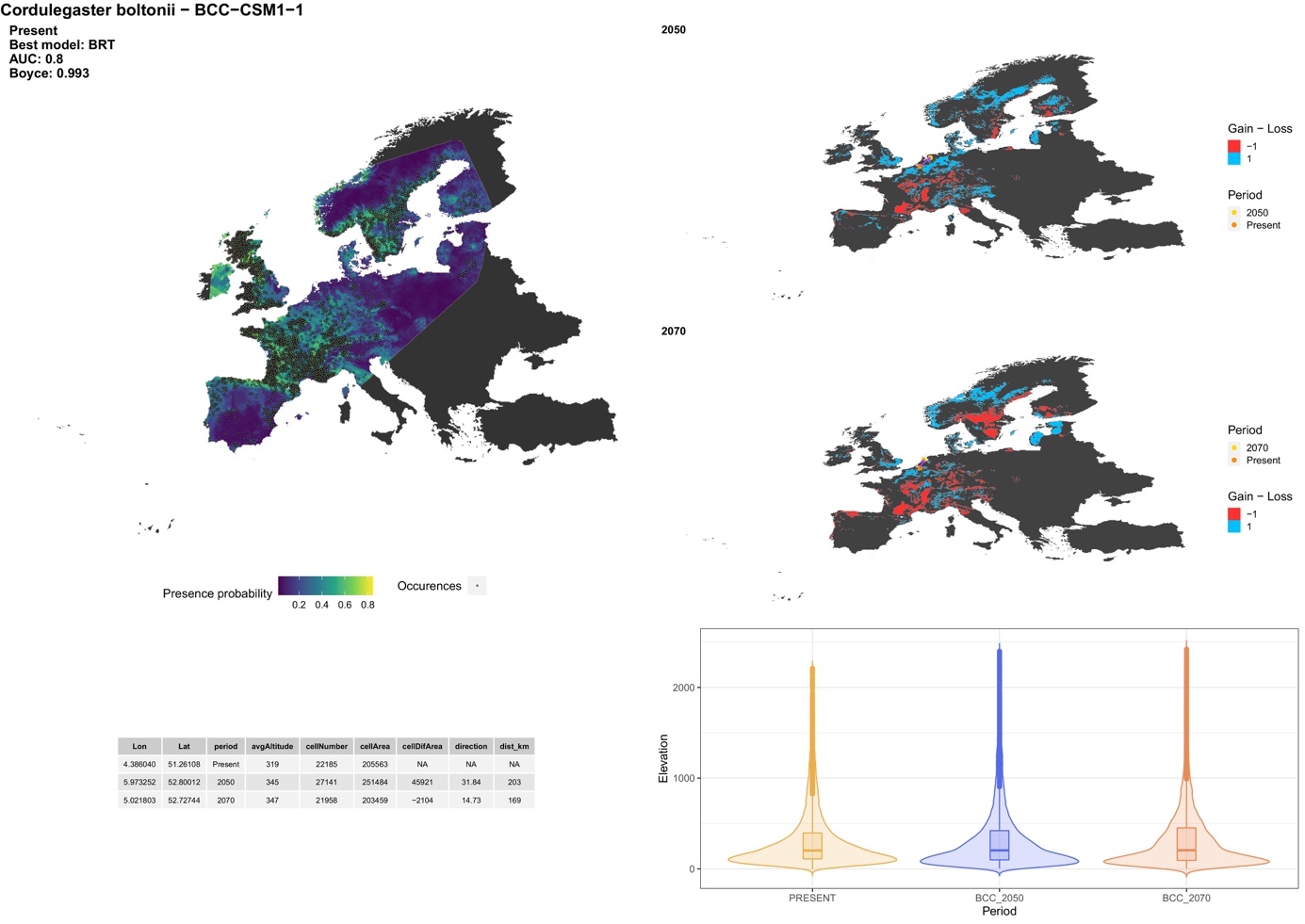


### **Family: Macromiidae**

### *Macromia splendens* (Pictet, 1843)


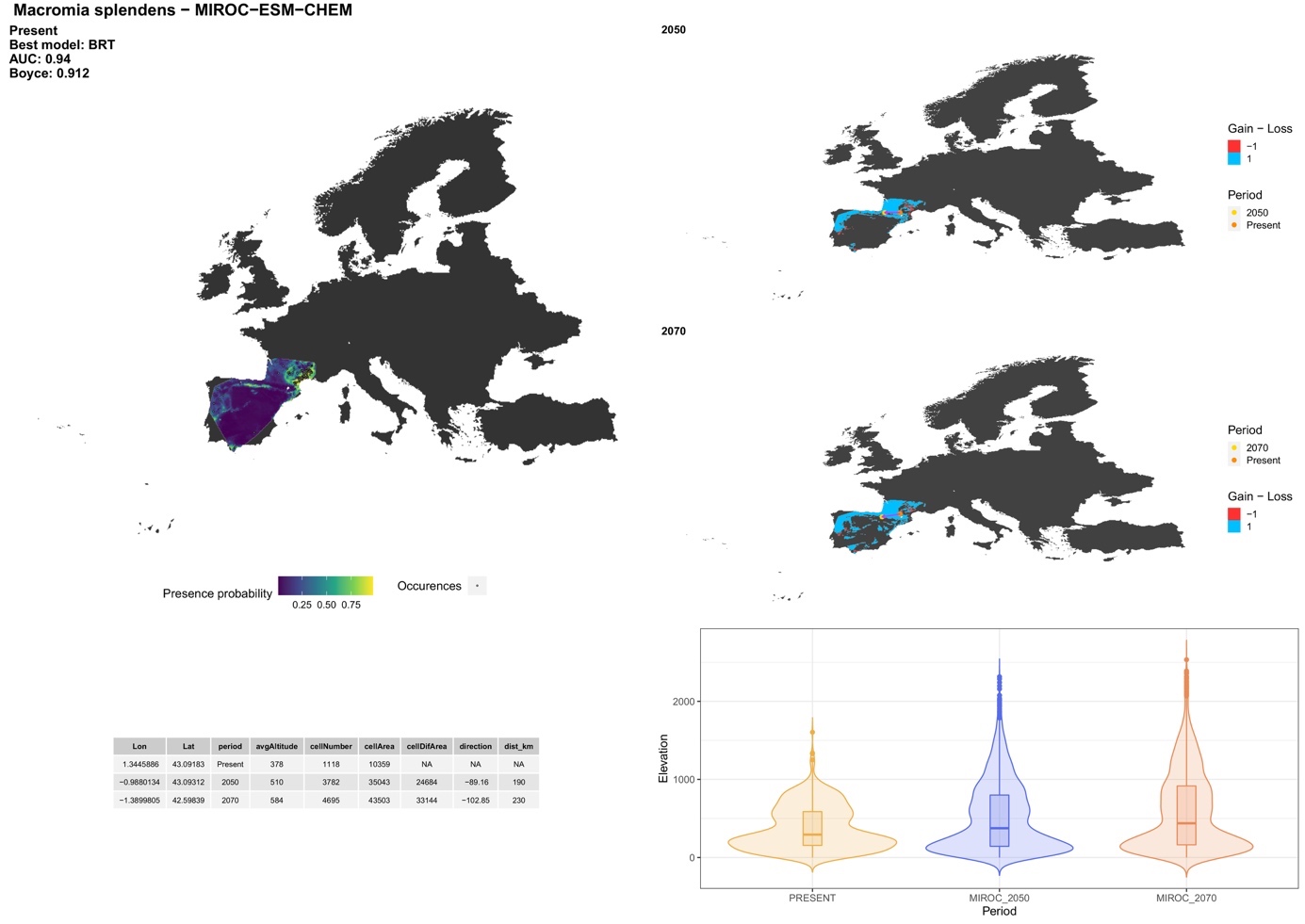


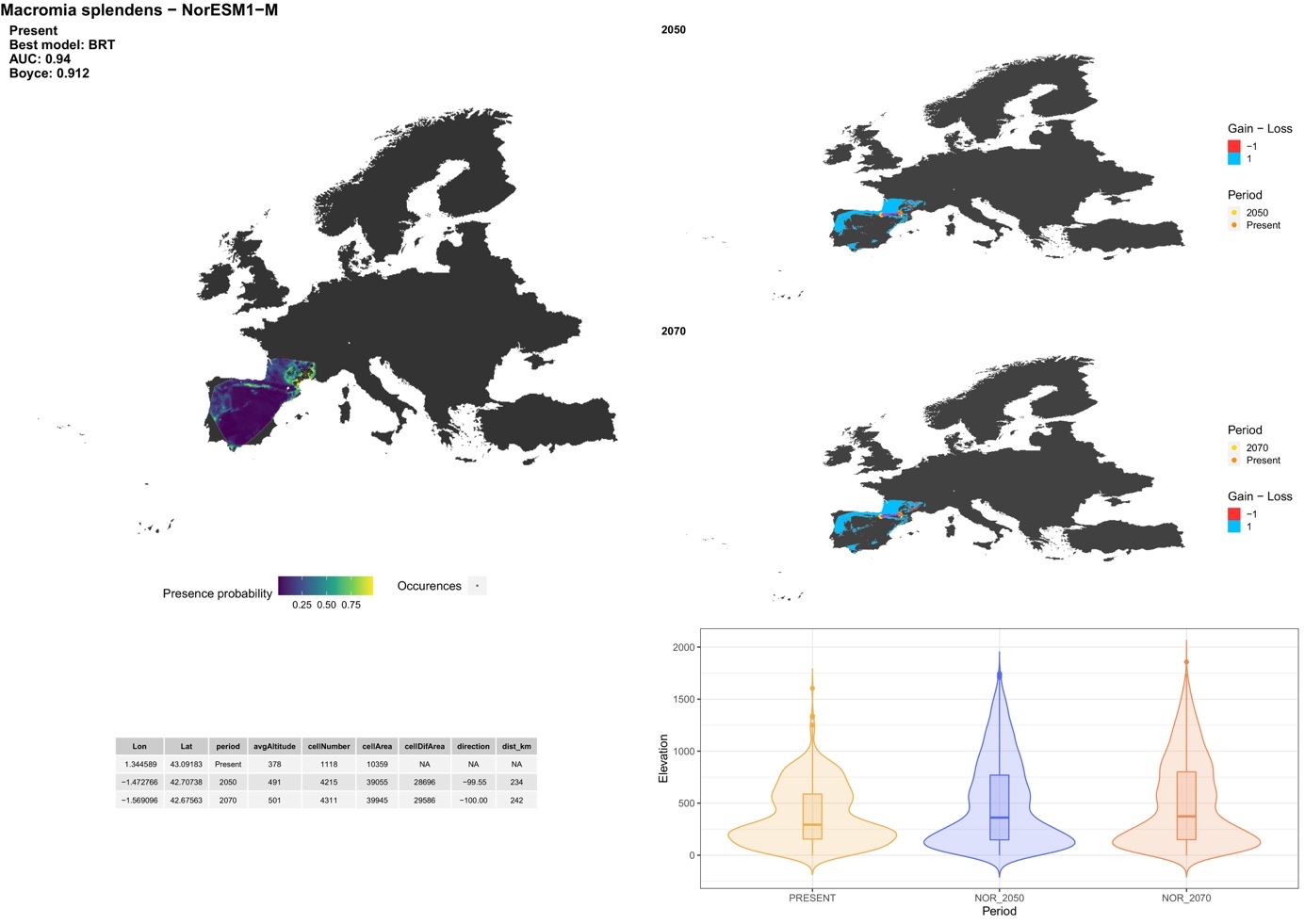


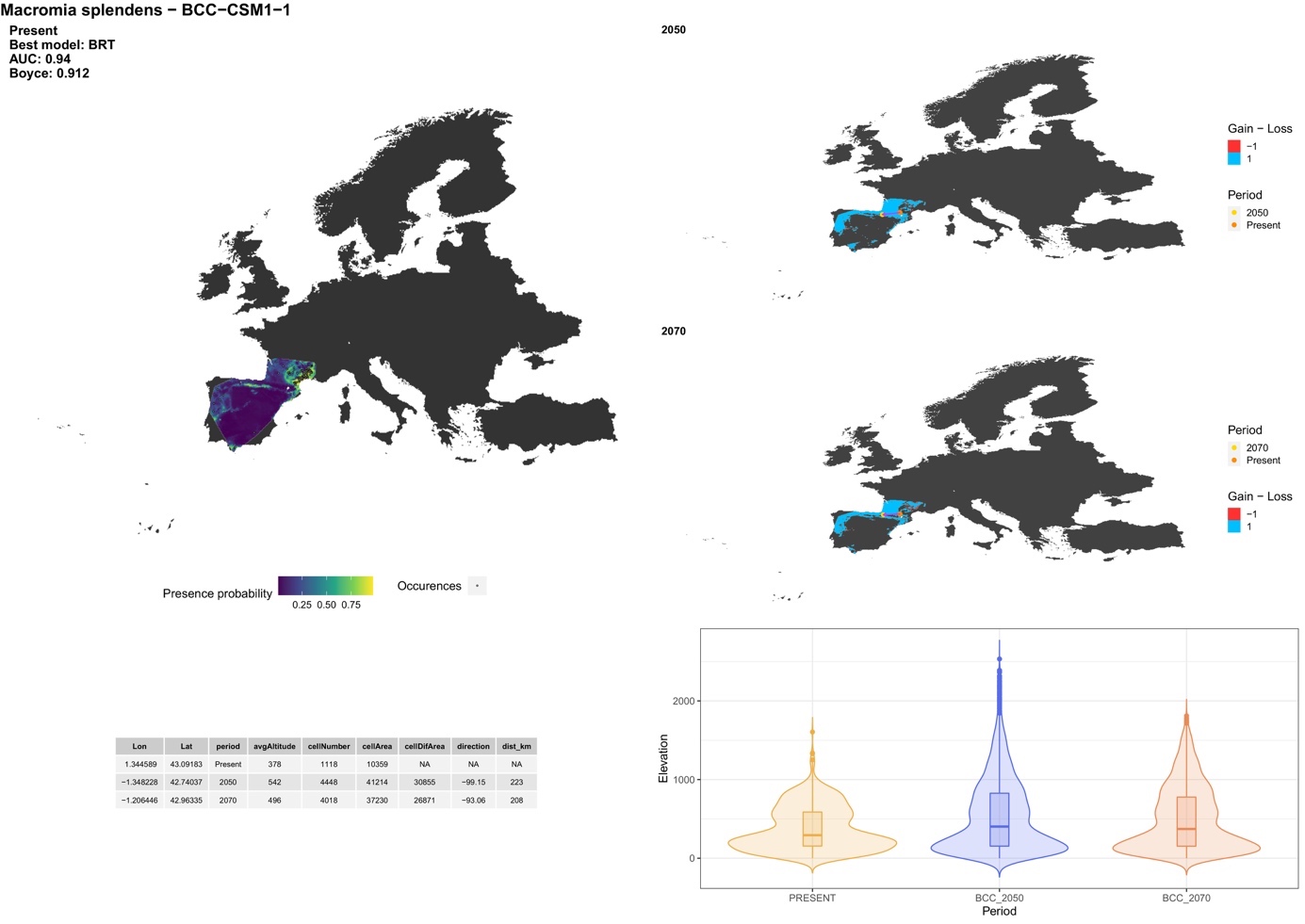


### **Family: Corduliidae**

### *Cordulia aenea* (Linnaeus, 1758)


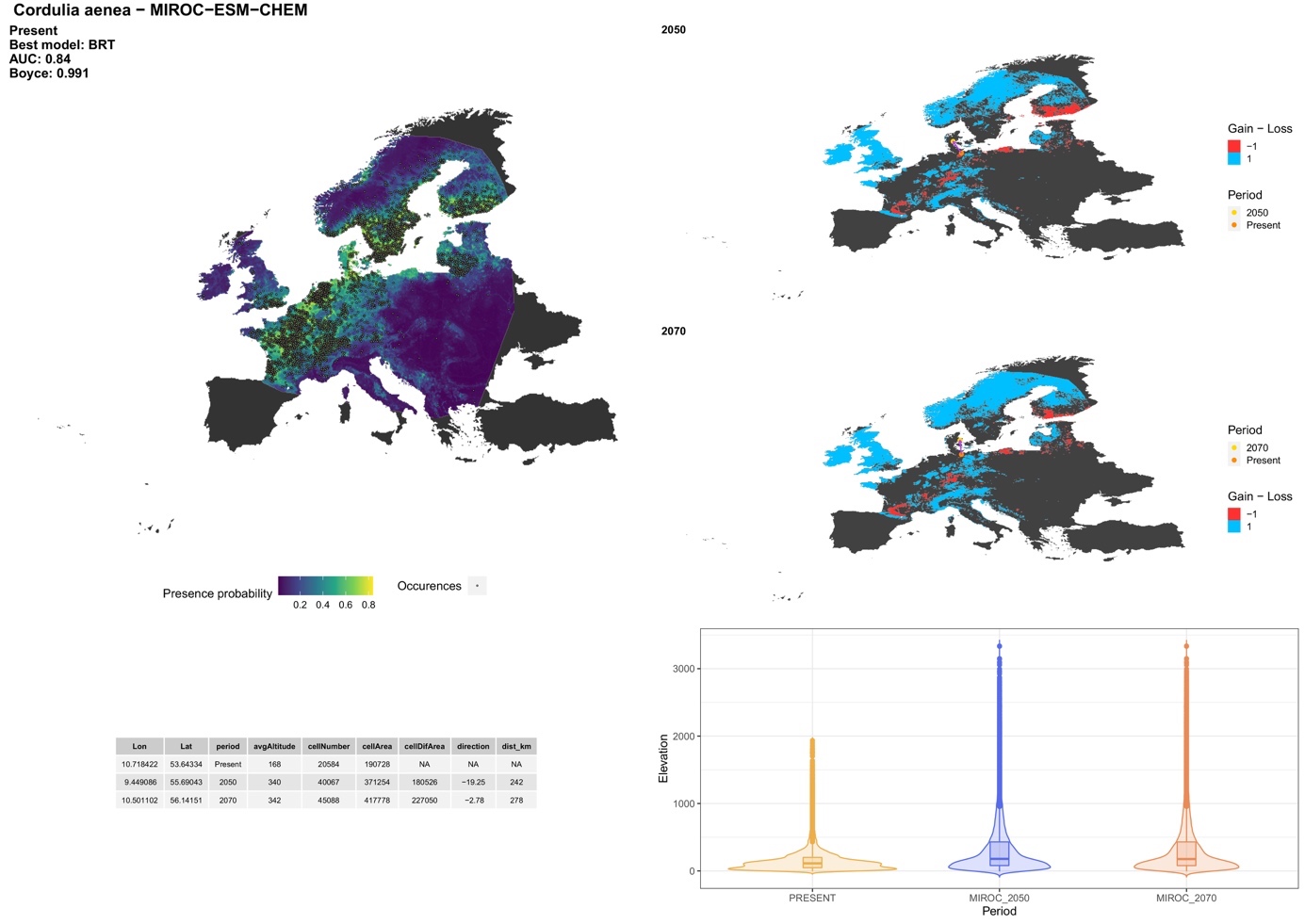


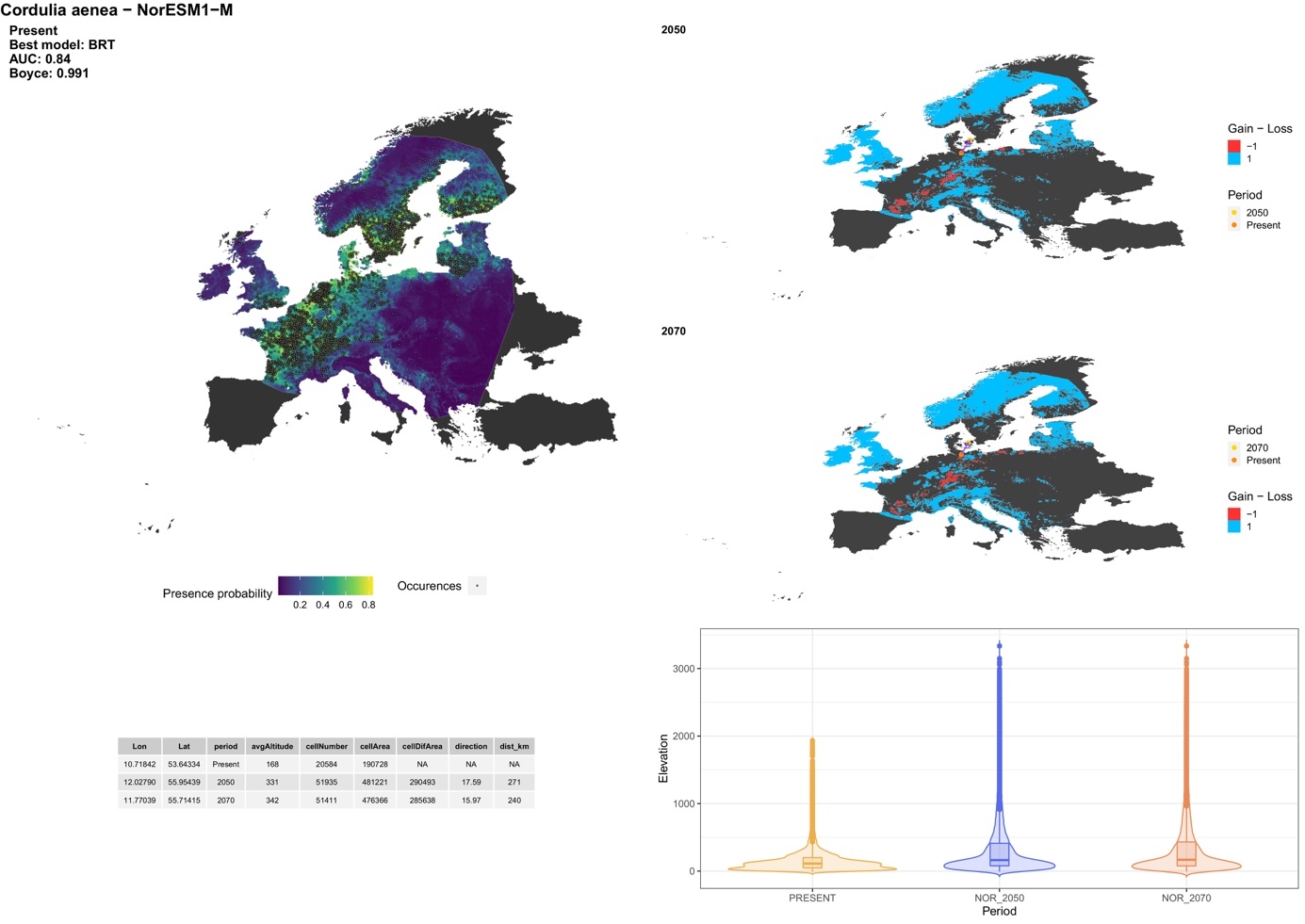


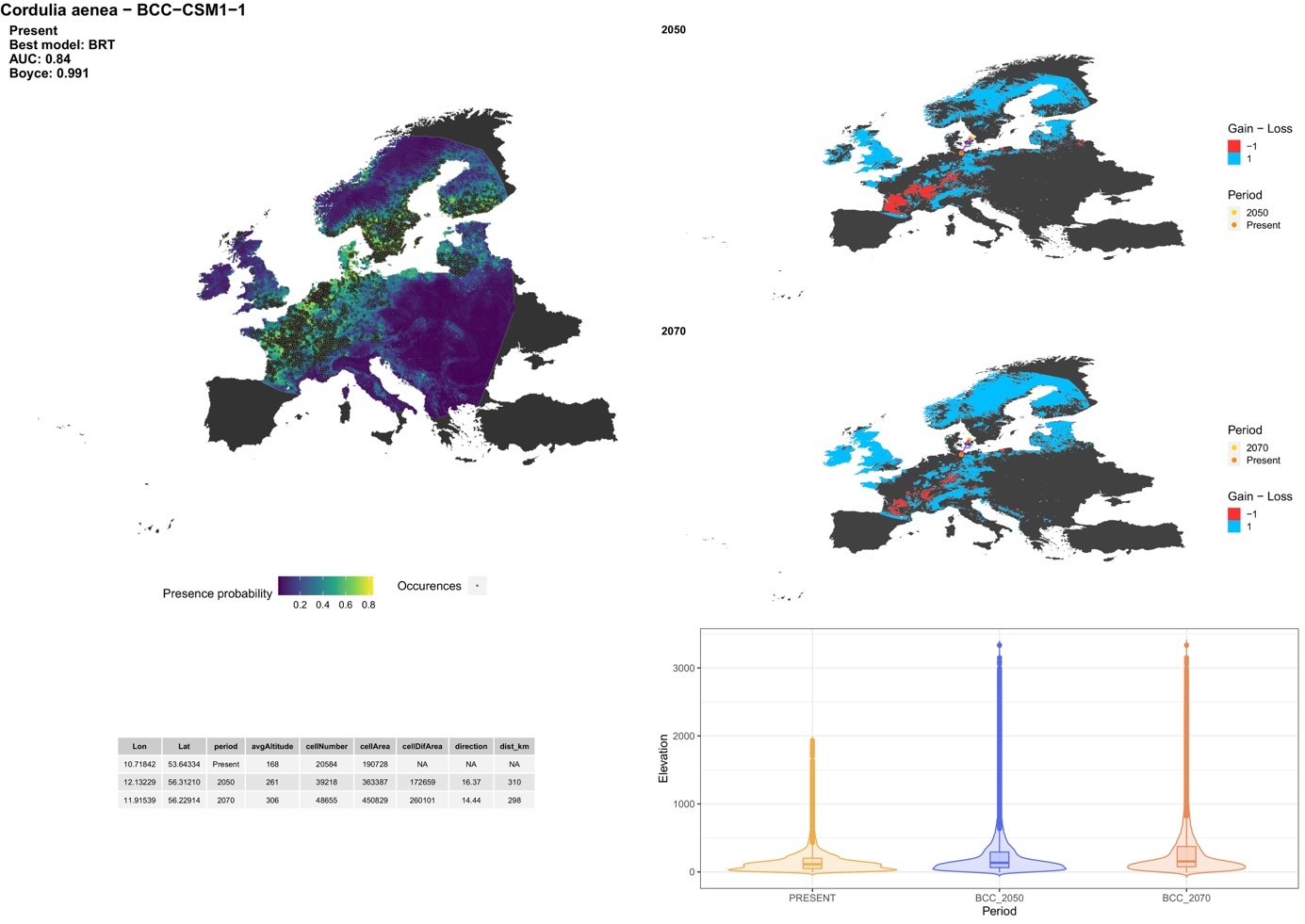


### *Epitheca bimaculate* (Charpentier, 1825)


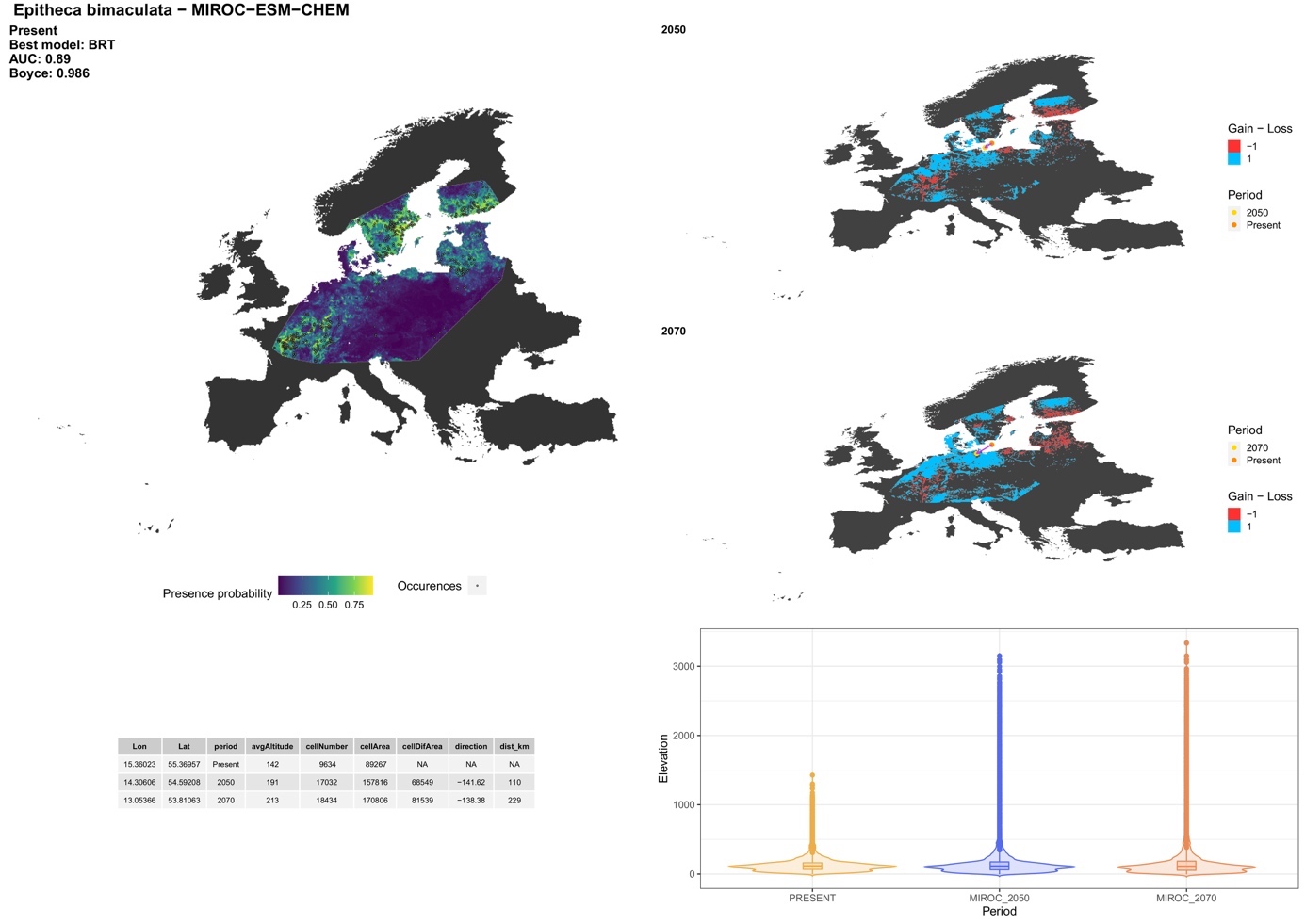


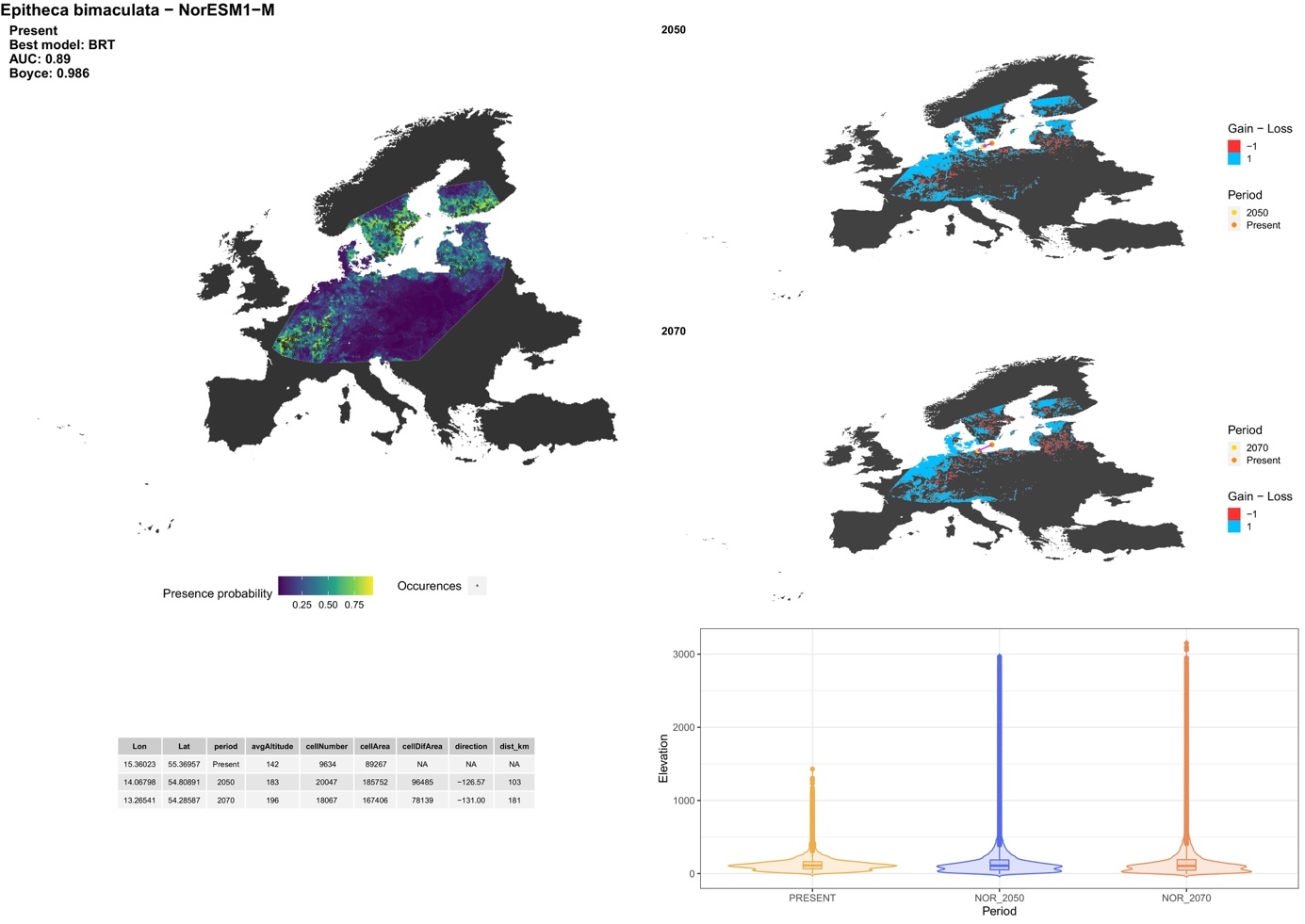


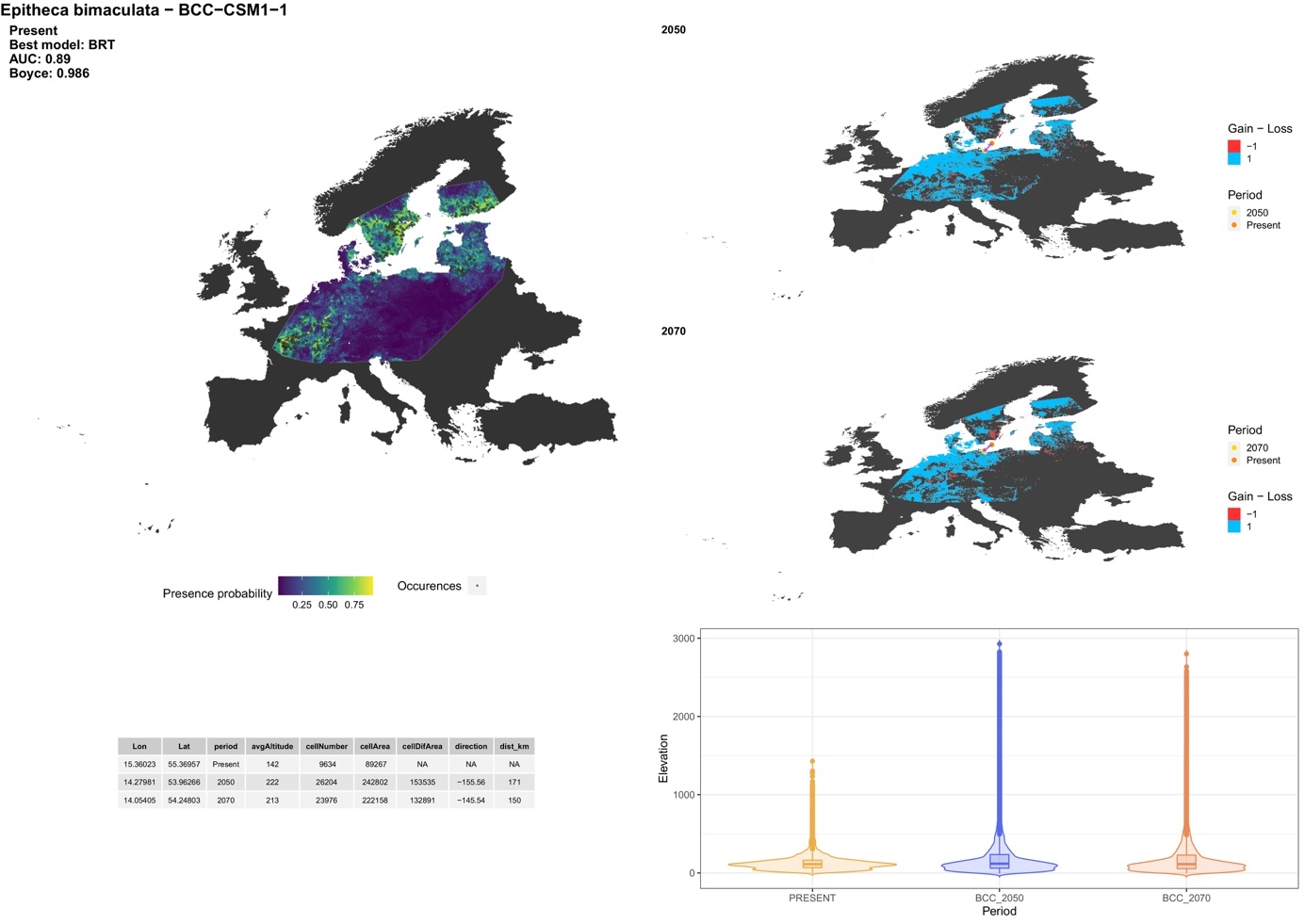


### *Somatochlora alpestris* (Selys, 1840)


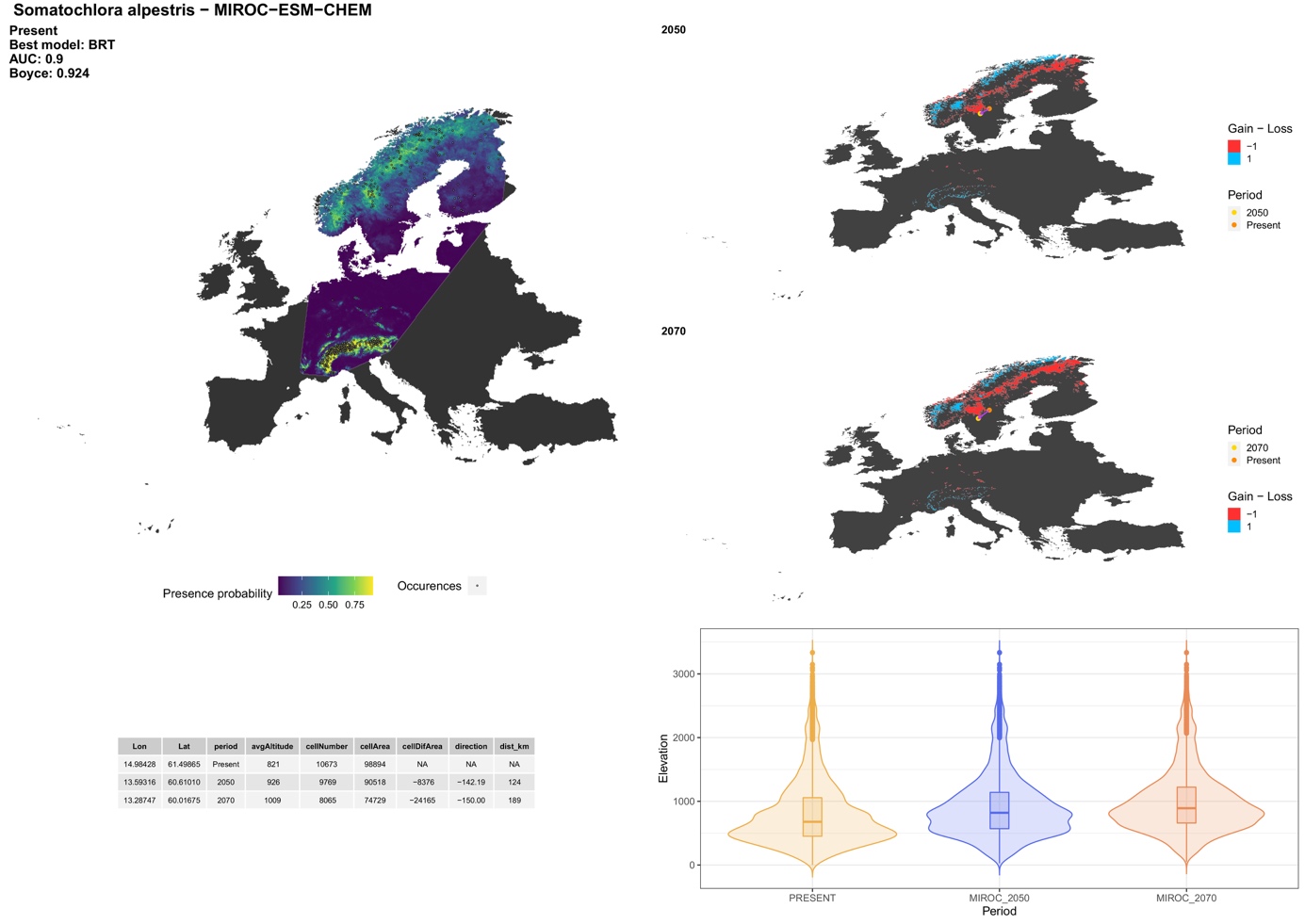


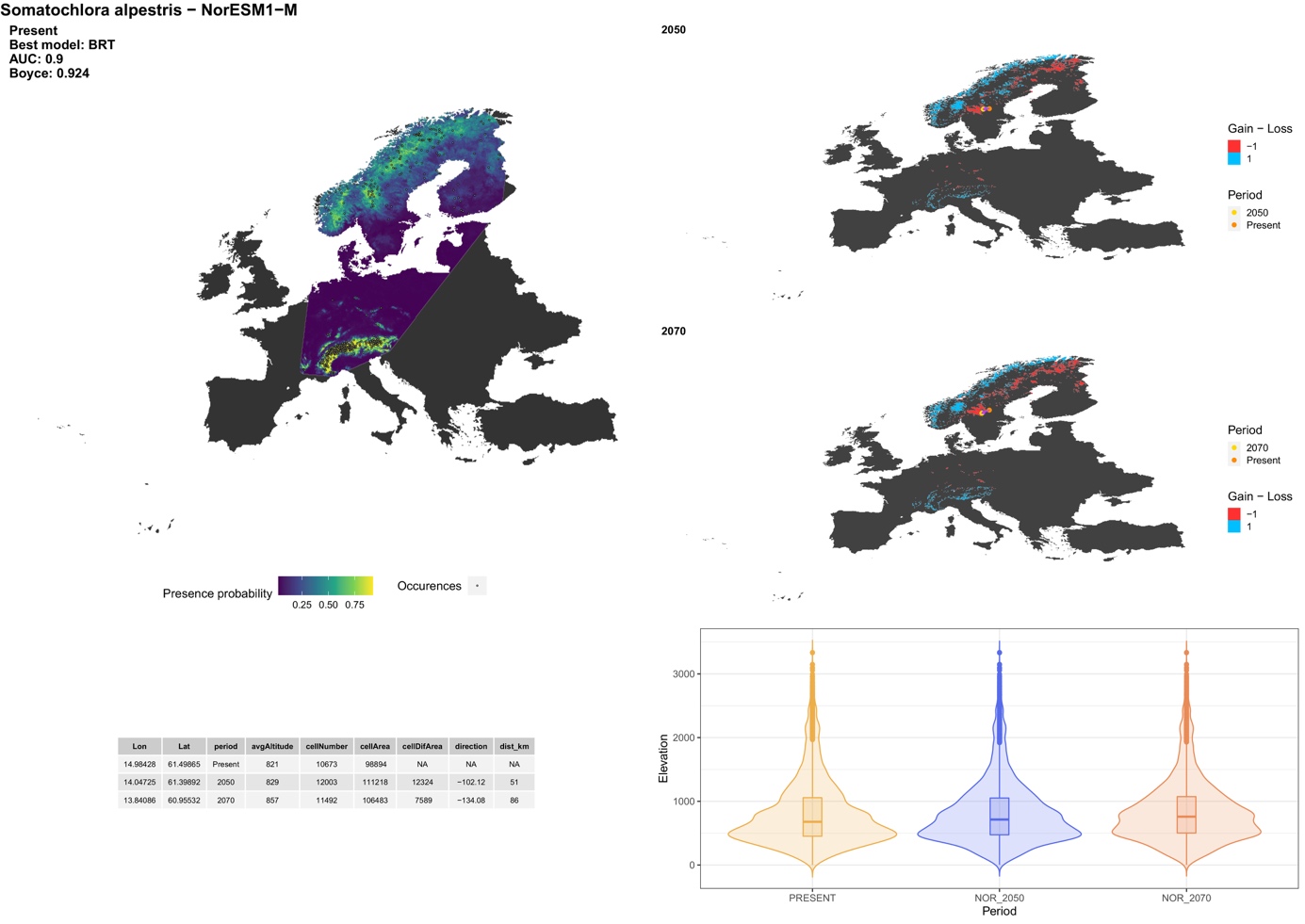


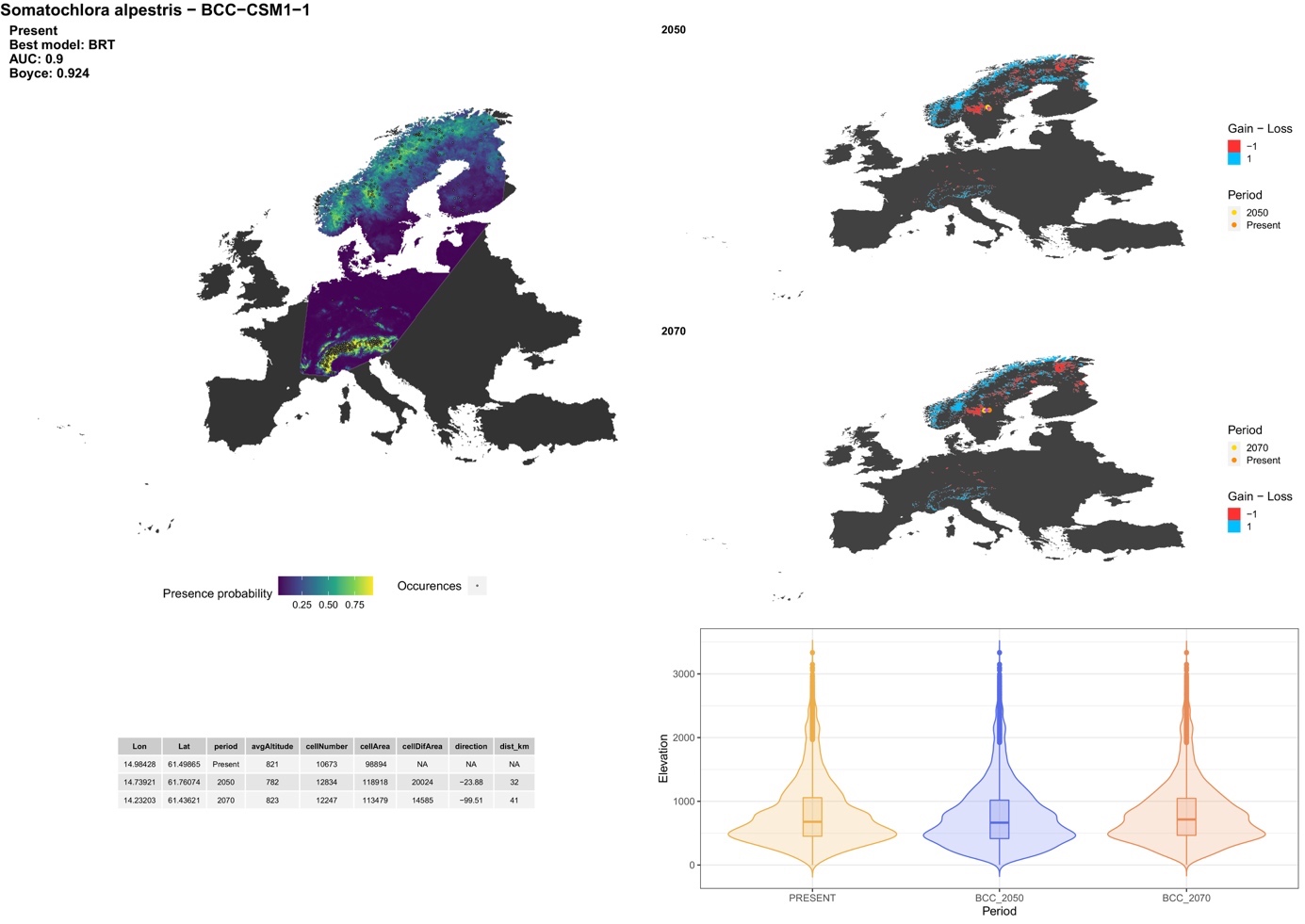


### *Somatochlora arctica* (Zetterstedt, 1840)


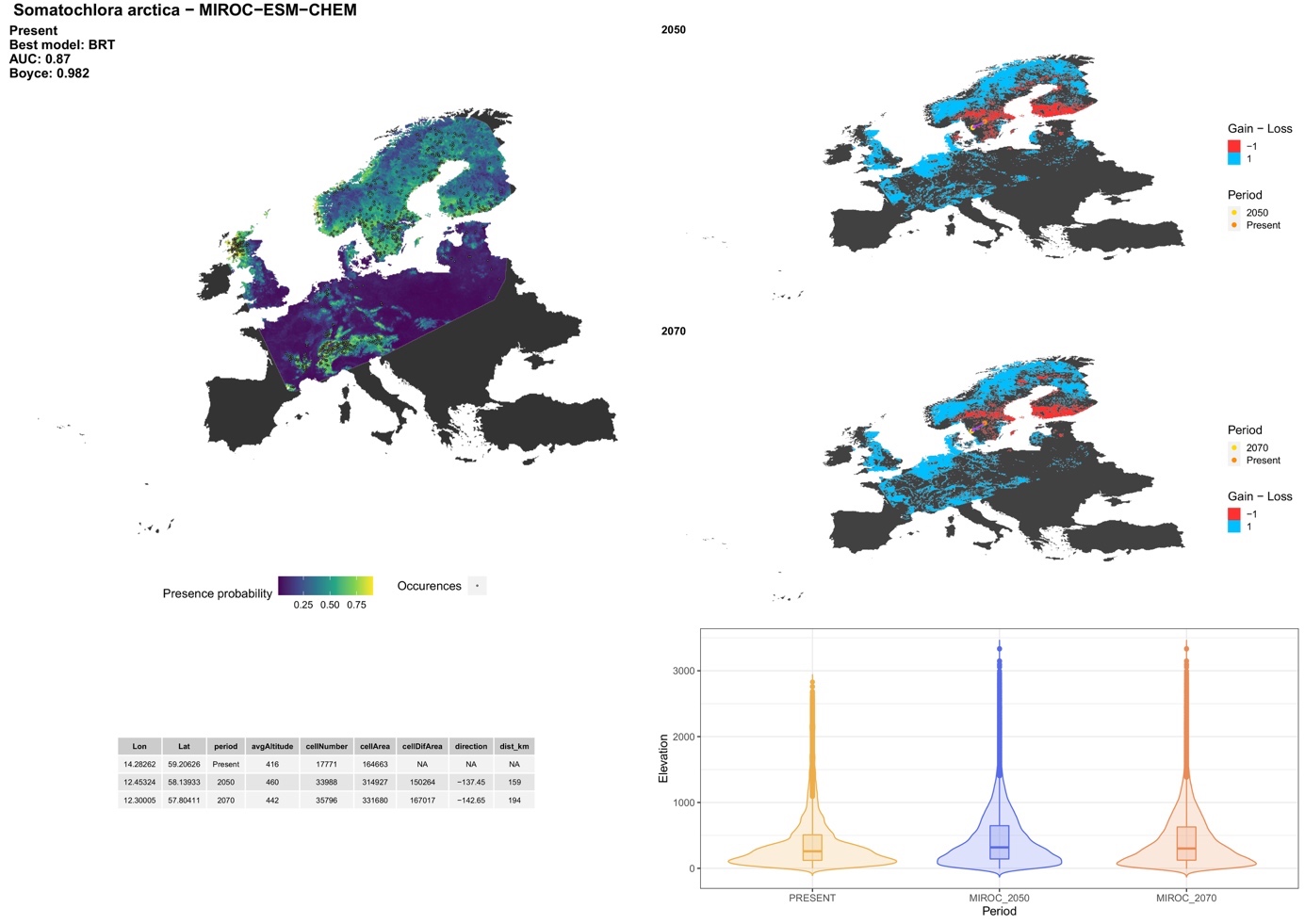


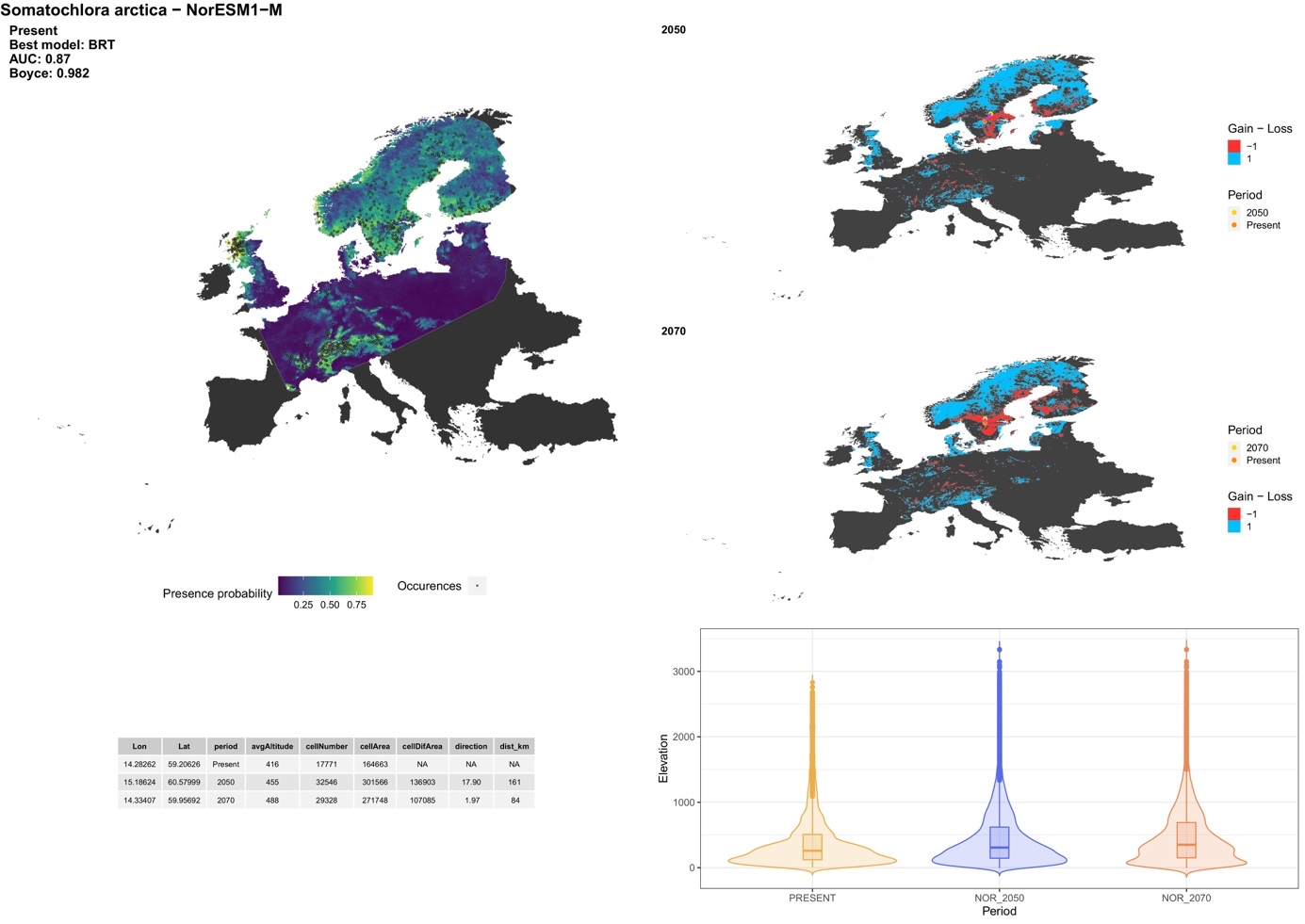


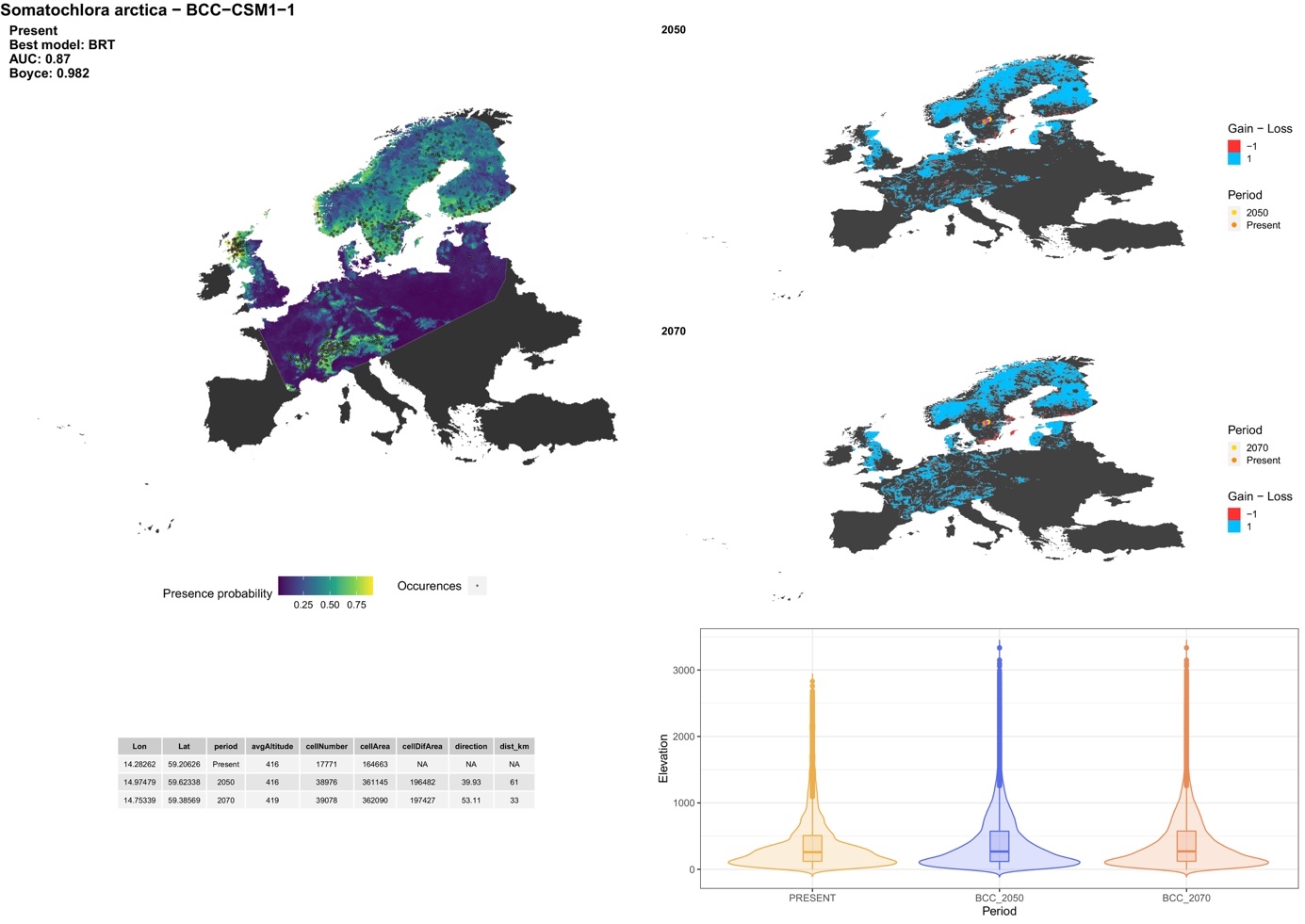


### *Somatochlora flavomaculata* (Vander Linden, 1825)

### *Somatochlora meridionalis* Nielsen, 1935

### *Somatochlora metallica* (Vander Linden, 1825)

### **Family: Libellulidae**

### *Brachythemis impartita* (Karsch, 1890)

### *Crocothemis erythraea* (Brullé, 1832)

### *Diplacodes lefebvrii* Rambur, 1842

### *Leucorrhinia albifrons* (Burmeister, 1839)

### *Leucorrhinia caudalis* (Charpentier, 1840)

### *Leucorrhinia dubia* (Vander Linden, 1825)

### *Leucorrhinia pectoralis* (Charpentier, 1825)

### *Leucorrhinia rubicunda* (Linnaeus, 1758)

### *Libellula depressa* Linnaeus, 1758

### *Libellula fulva* Müller, 1764

### *Libellula quadrimaculata* Linnaeus, 1758

### *Orthetrum albistylum* (Selys, 1848)

### *Orthetrum brunneum* (Fonscolombe, 1837)

### *Orthetrum cancellatum* (Linnaeus, 1758)

### *Orthetrum chrysostigma* (Burmeister, 1839)

### *Orthetrum coerulescens* (Fabricius, 1798)

### *Orthetrum taeniolatum* (Schneider, 1845)

### *Orthetrum trinacria* (Selys, 1841)

### *Selysiothemis nigra* (Vander Linden, 1825)

### *Sympetrum danae* (Sulzer, 1776)

### *Sympetrum depressiusculum* (Selys, 1841)

### *Sympetrum flaveolum* (Linnaeus, 1758)

### *Sympetrum fonscolombii* (Selys, 1840)

### *Sympetrum meridionale* (Selys, 1841)

### *Sympetrum pedemontanum* (Müller in Allioni, 1766)

### *Sympetrum sanguineum* (Müller, 1764)

### *Sympetrum sinaiticum* Dumont, 1977

### *Sympetrum striolatum* (Charpentier, 1840)

### *Sympetrum vulgatum* (Linnaeus, 1758)

### *Trithemis annulata* (Palisot de Beauvois, 1807)

### *Trithemis arteriosa* (Burmeister, 1839)

### *Trithemis kirbyi* Selys, 1891

# **Zygoptera**

### **Family: Calopterygidae**

### *Calopteryx haemorrhoidalis* (Vander Linden, 1825)

### *Calopteryx splendens* (Harris, 1780)

### *Calopteryx virgo* (Linnaeus, 1758)

### *Calopteryx xanthostoma* (Charpentier, 1825)

### **Family: Coenagrionidae**

### *Ceriagrion tenellum* (De Villers, 1789)

### *Coenagrion armatum* (Charpentier, 1840)

### *Coenagrion caerulescens* (Fonscolombe, 1838)

### *Coenagrion hastulatum* (Charpentier, 1825)

### *Coenagrion johanssoni* Wallengren, 1894

### *Coenagrion lunulatum* (Charpentier, 1840)

### *Coenagrion mercuriale* (Charpentier, 1840)

### *Coenagrion ornatum* (Selys, 1850)

### *Coenagrion puella* (Linnaeus, 1758)

### *Coenagrion pulchellum* (Vander Linden, 1825)

### *Coenagrion scitulum* (Rambur, 1842)

### *Enallagma cyathigerum* (Charpentier, 1840)

### *Erythromma lindenii* (Selys, 1840)

### *Erythromma najas* (Hansemann, 1823)

### *Erythromma viridulum* (Charpentier, 1840)

### *Ischnura elegans* (Vander Linden, 1820)

### *Ischnura genei* (Rambur, 1842)

### *Ischnura graellsii* (Rambur, 1842)

### *Ischnura pumilio* (Charpentier, 1825)

### *Nehalennia speciosa* (Charpentier, 1840)

### *Pyrrhosoma nymphula* (Sulzer, 1776)

### **Family: Euphaeidae**

### *Epallage fatime* (Charpentier, 1840)

### **Family: Lestidae**

### *Chalcolestes parvidens* Artobolevsky, 1929

### *Chalcolestes viridis* (Vander Linden, 1825)

### *Lestes barbarous* (Fabricius, 1798)

### *Lestes dryas* Kirby, 1890

### *Lestes macrostigma* (Eversmann, 1836)

### *Lestes sponsa* (Hansemann, 1823)

### *Lestes virens* (Charpentier, 1825)

### *Sympecma fusca* (Vander Linden, 1820)

### *Sympecma paedisca* (Brauer, 1877)

### **Family: Platycnemididae**

### *Platycnemis acutipennis* Selys, 1841

### *Platycnemis latipes* Rambur, 1842

### *Platycnemis pennipes* (Pallas, 1771)

### **Family: *Incertae sedis***

### *Oxygastra curtisii* (Dale, 1834)
