# Supplementary material for: Climate change will redefine taxonomic, functional, and phylogenetic diversity of Odonata in space and time
Source: NPJ Biodivers. 2022 Nov 17;1:1. doi: 10.1038/s44185-022-00001-3 (PMC11290607; doi:10.1038/s44185-022-00001-3)

**Supplementary Material 4**.

Quantification of *α* diversity per different climate scenarios (BCC-CSM1-1; MIROC-ESM-CHEM; NorESM1-M) and time periods (current; 2050; 2070). For future scenarios, the cold-colour palette indicates the species loss, whereas the warm-colour palette indicates the spacies gain.


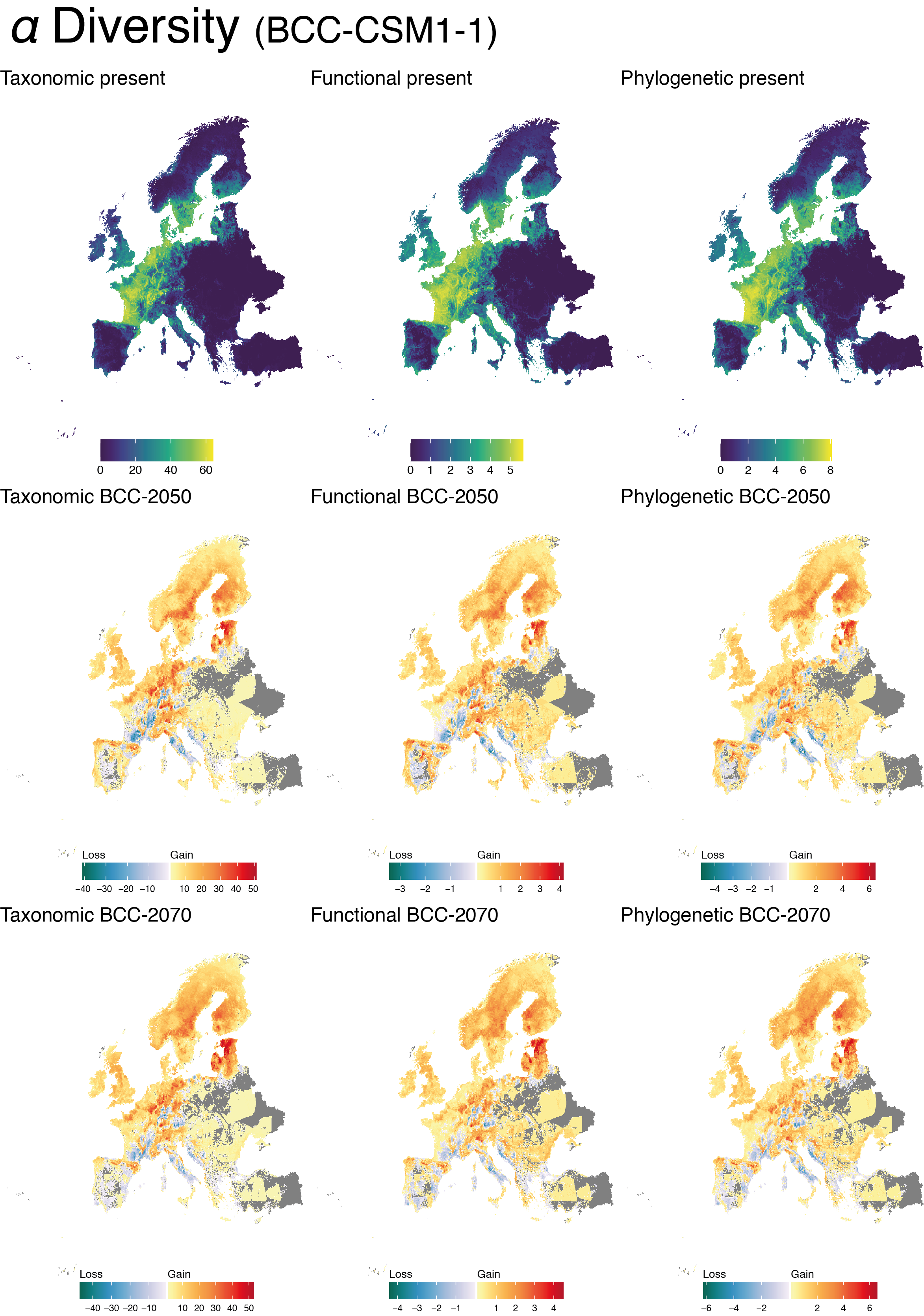


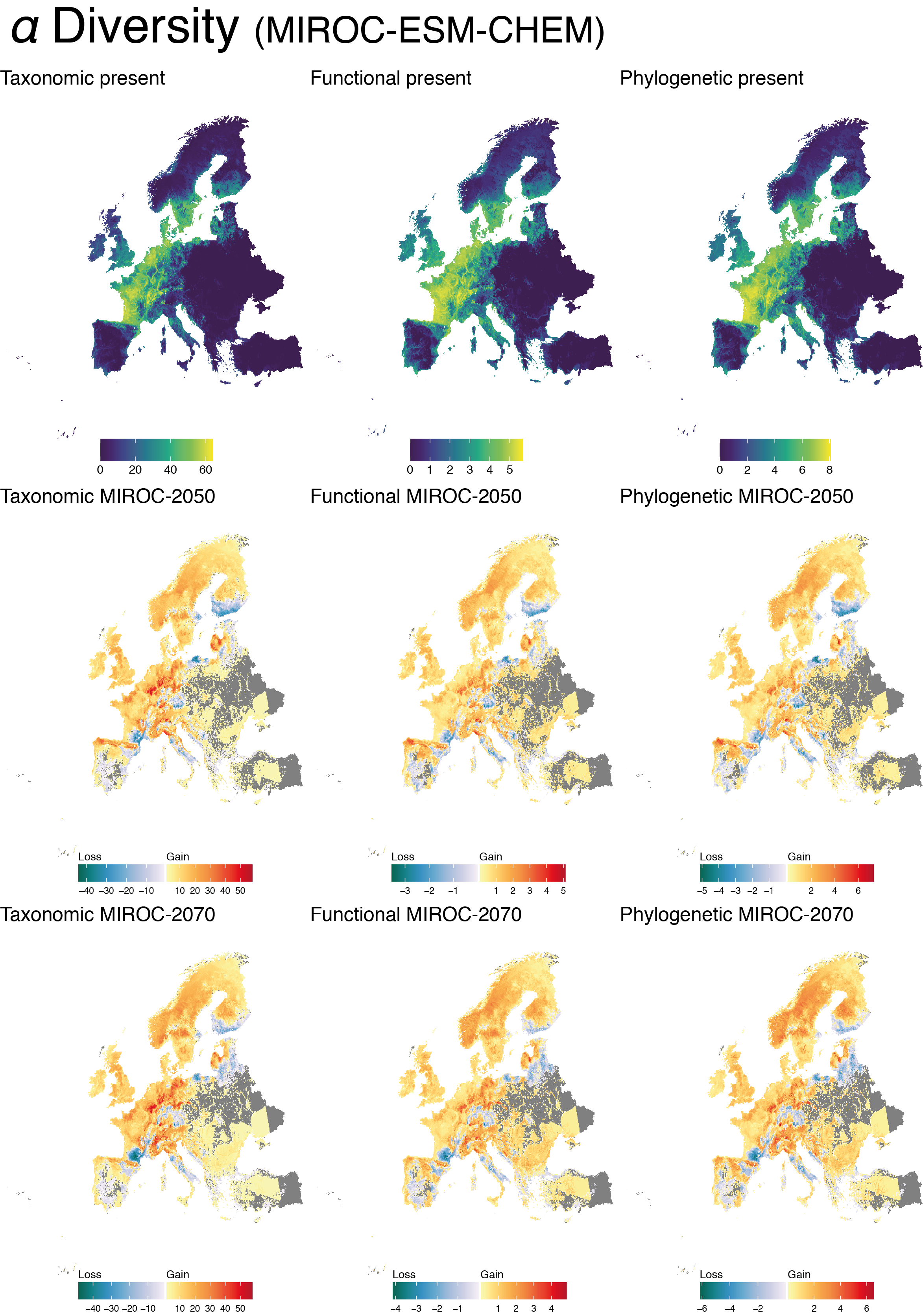


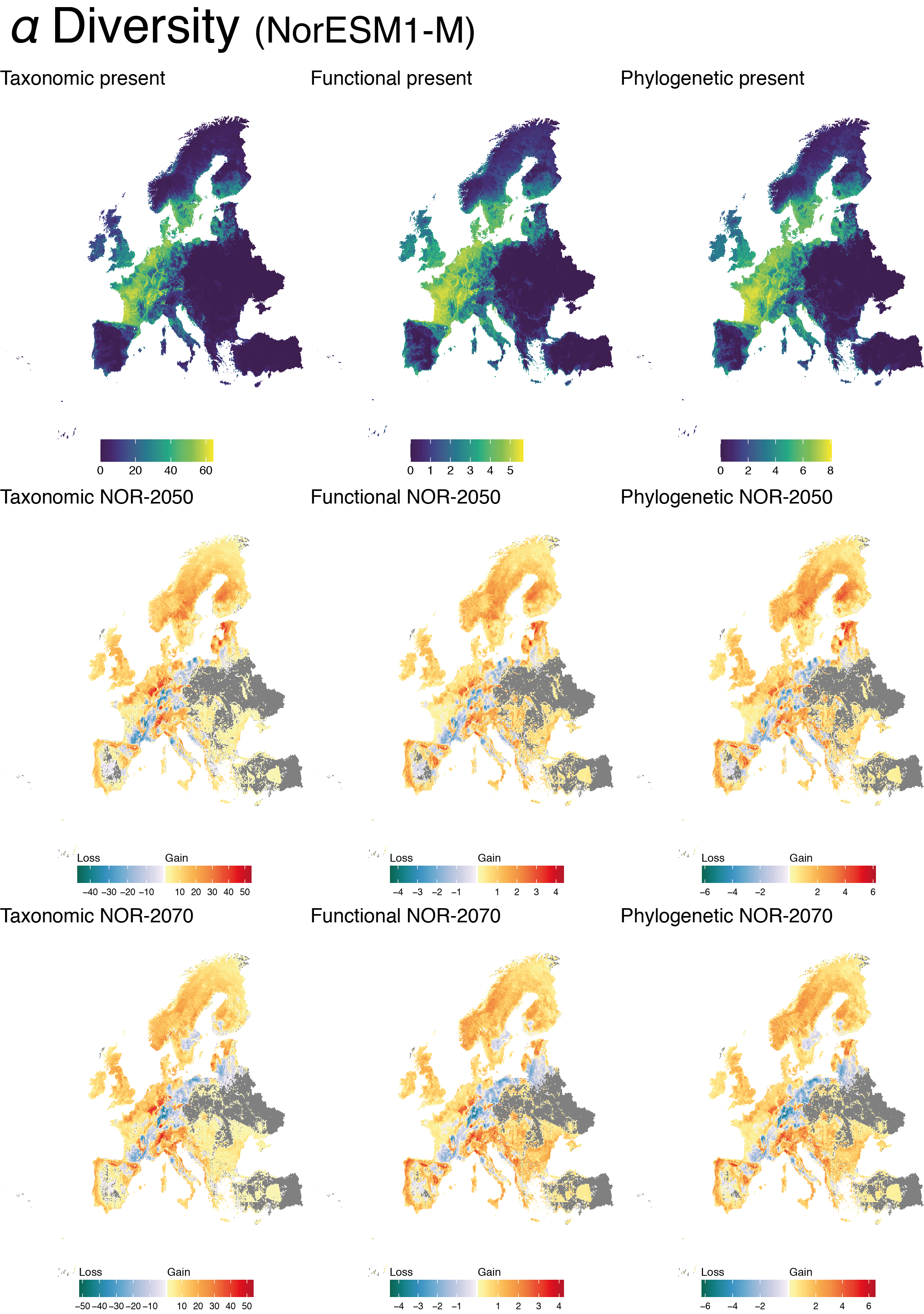


Quantification of *β* diversity (*β*-total; *β*-replacement; *β*-richness) per different climate scenarios (BCC-CSM1-1; MIROC-ESM-CHEM; NorESM1-M) and time periods (current; 2050; 2070).


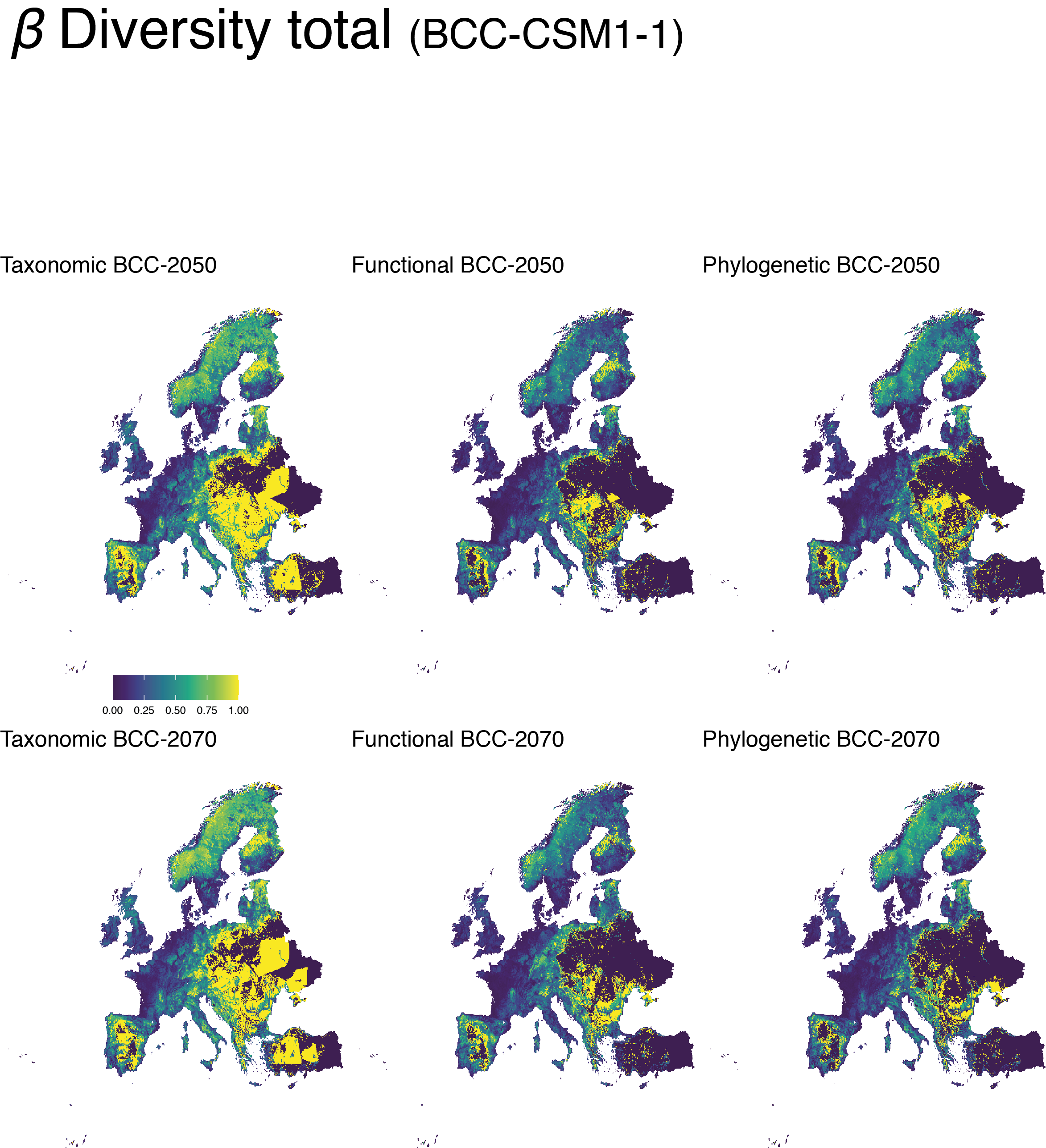


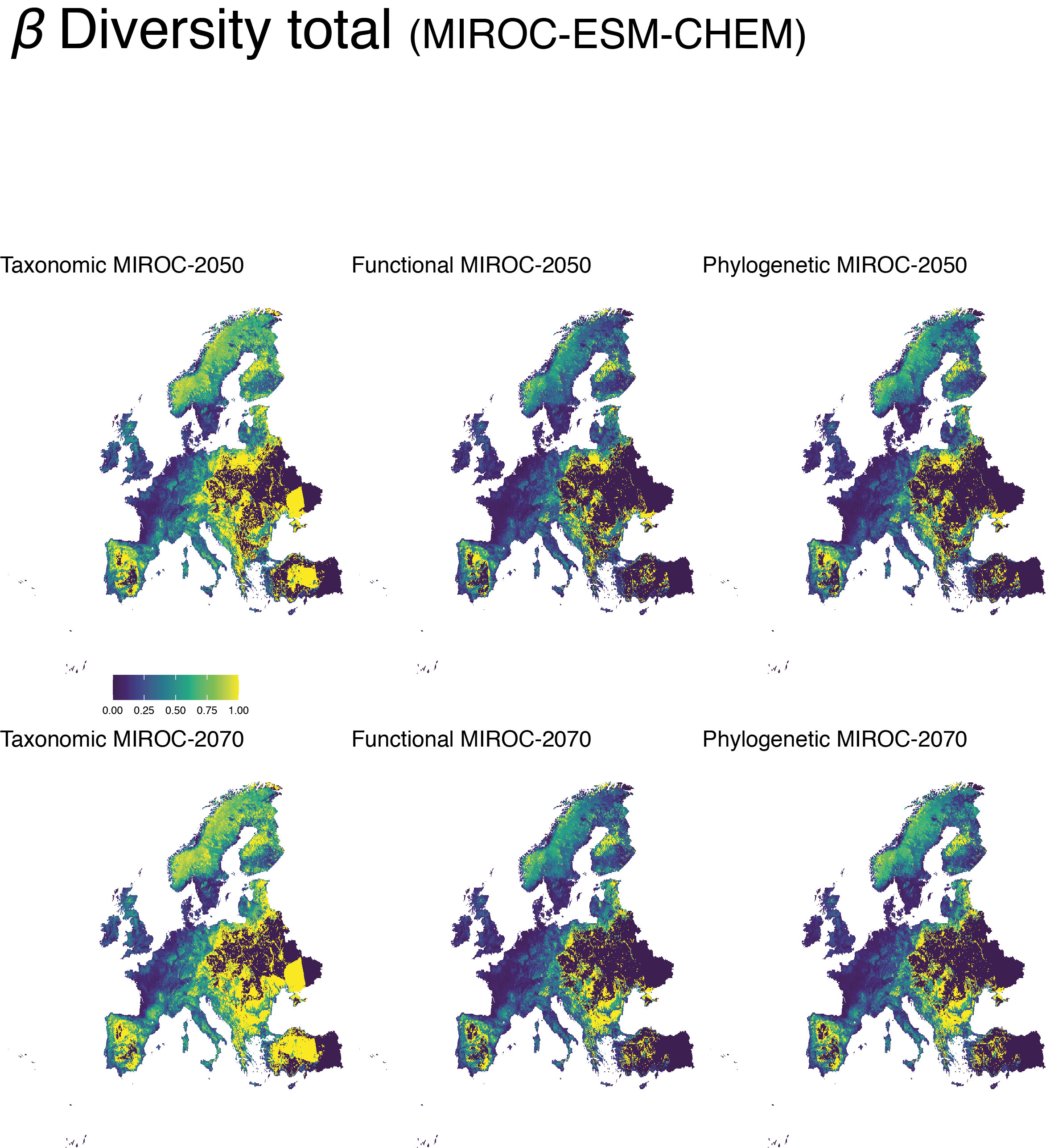


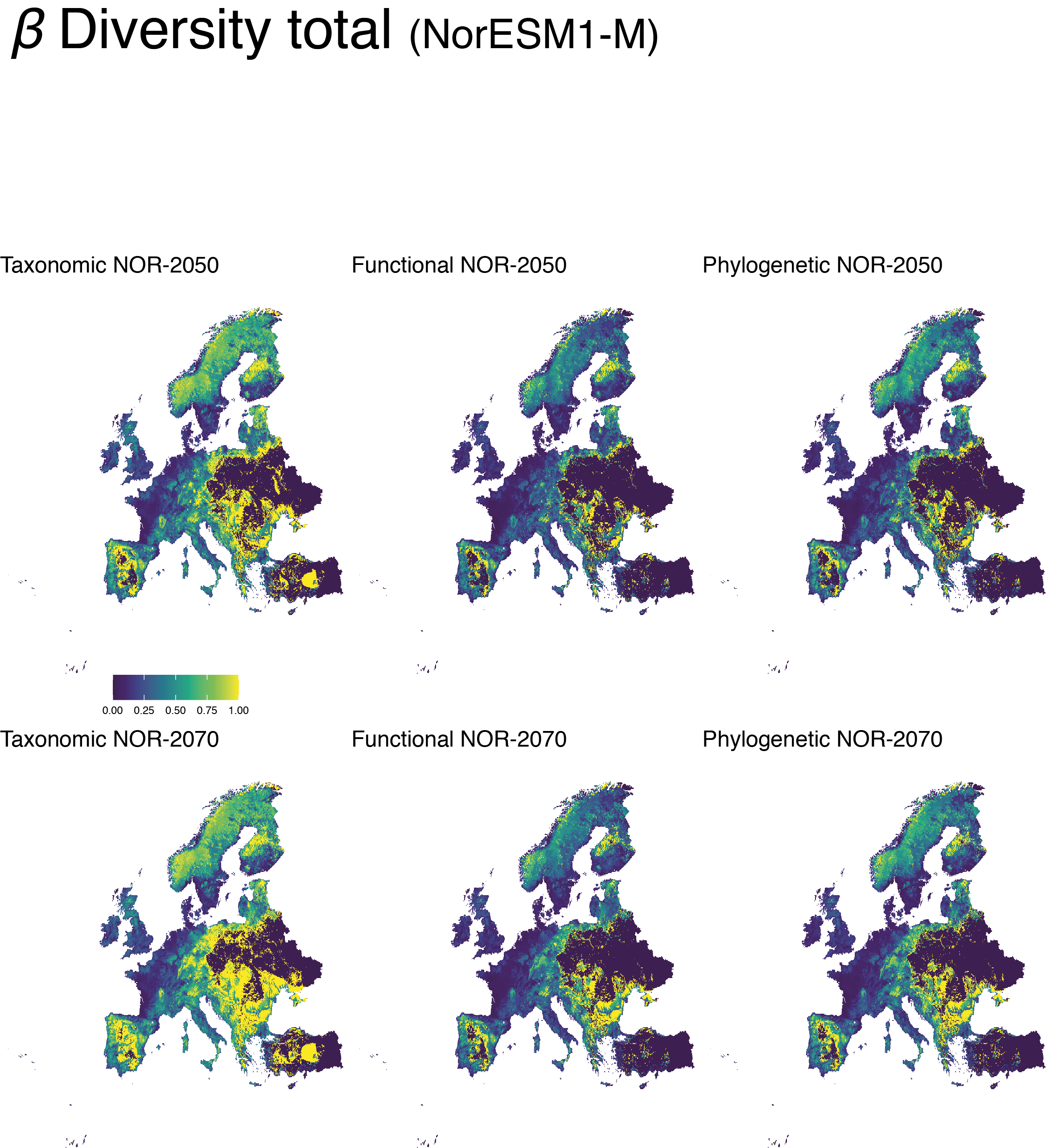


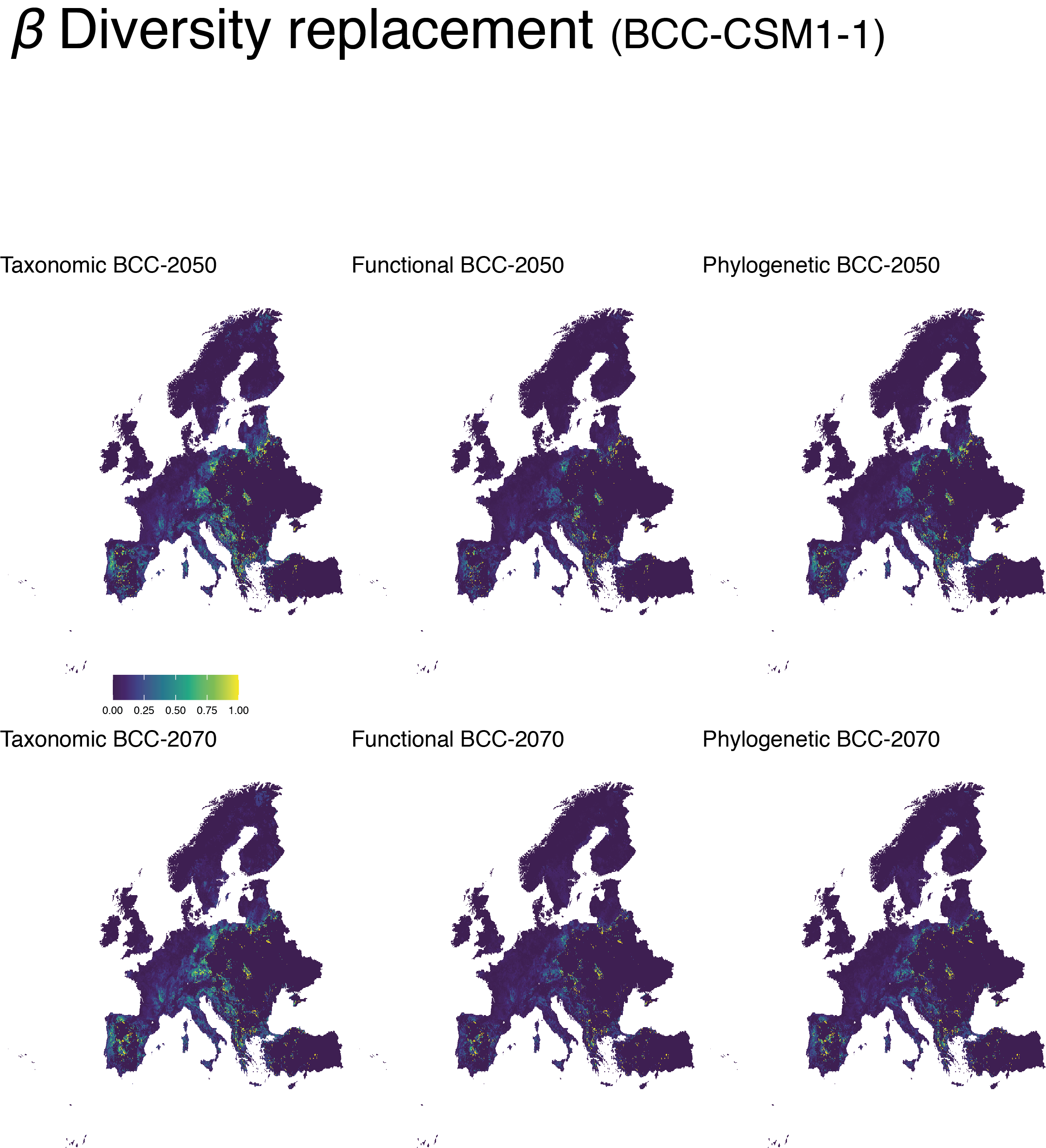


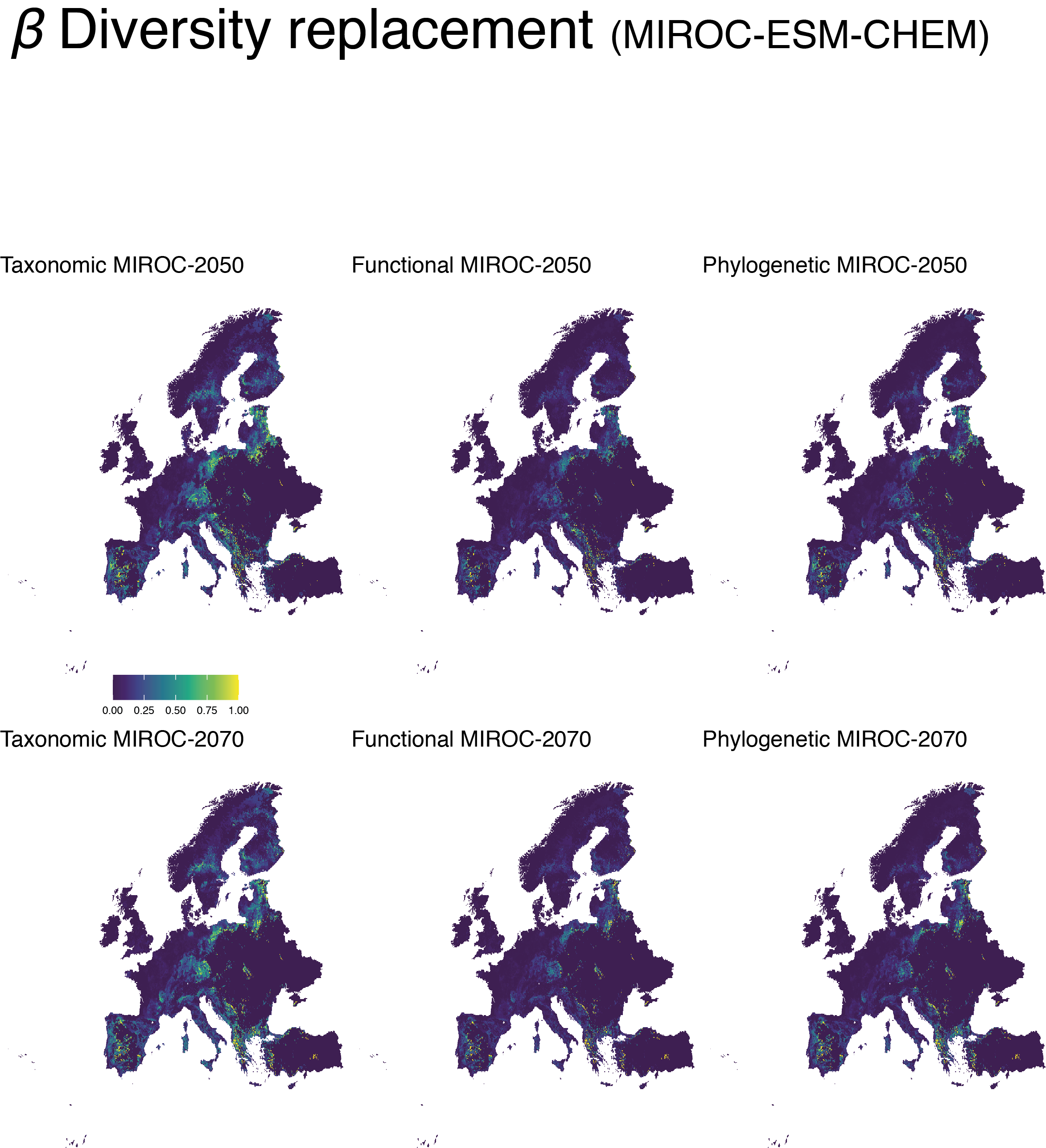


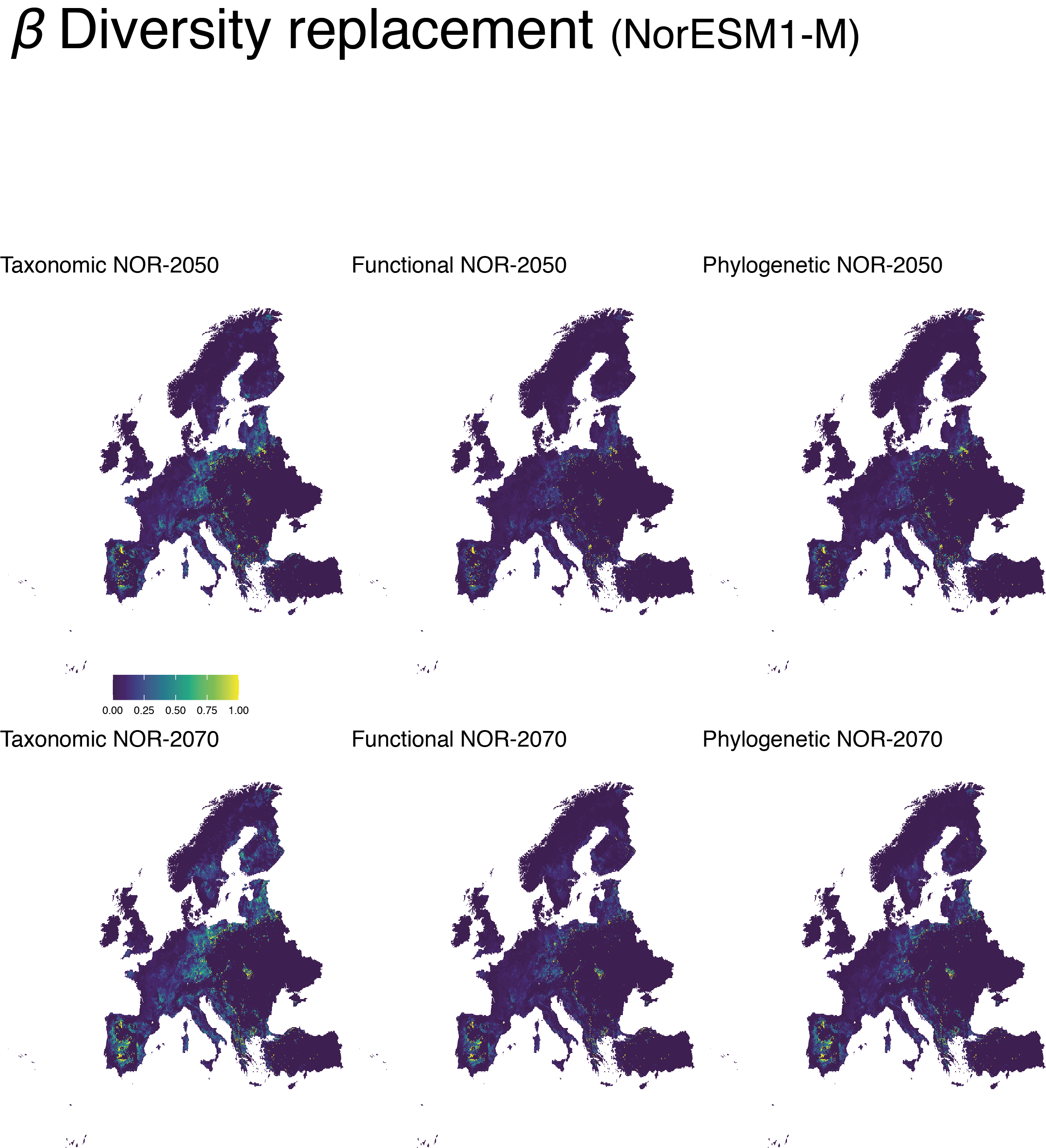


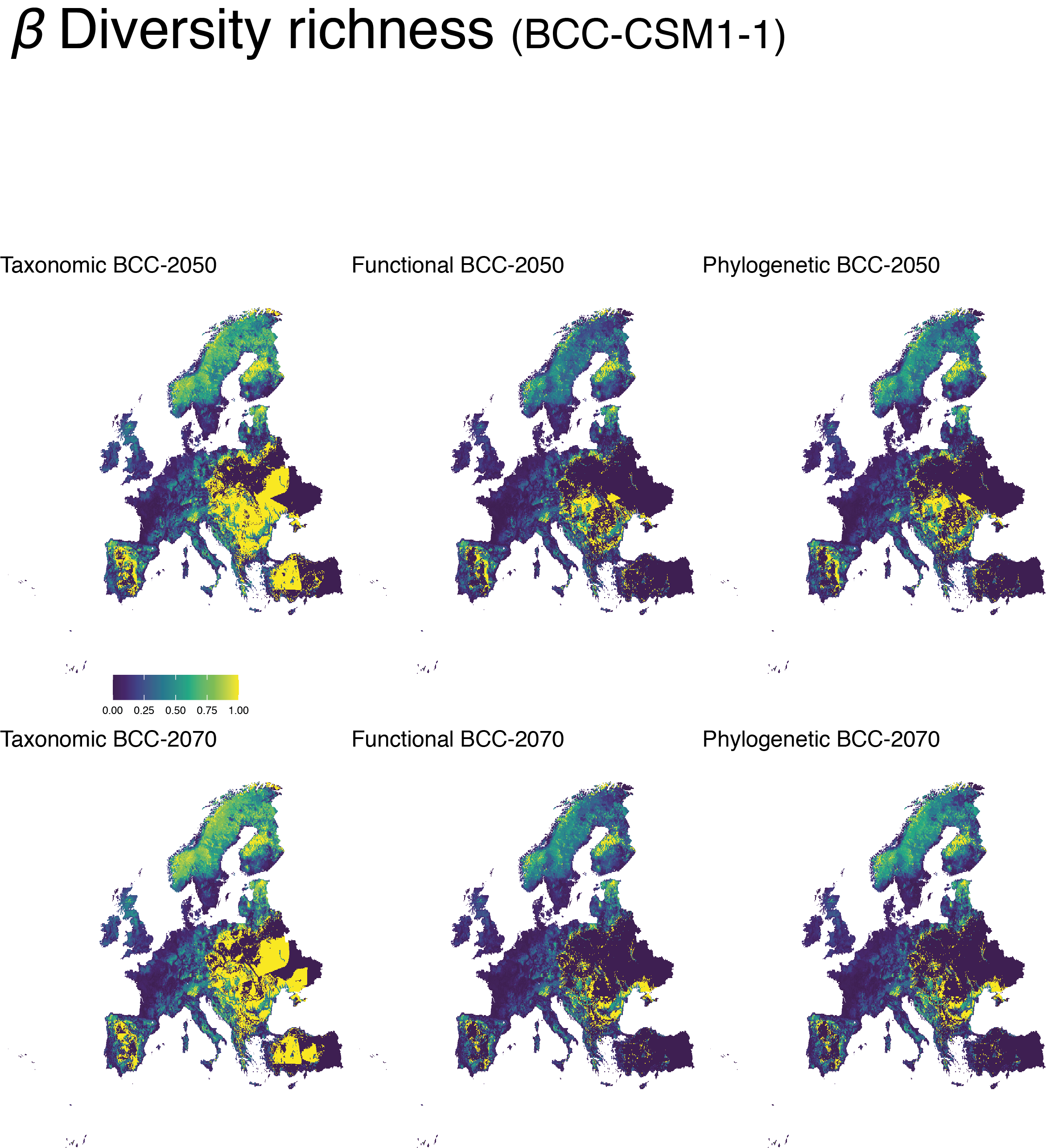


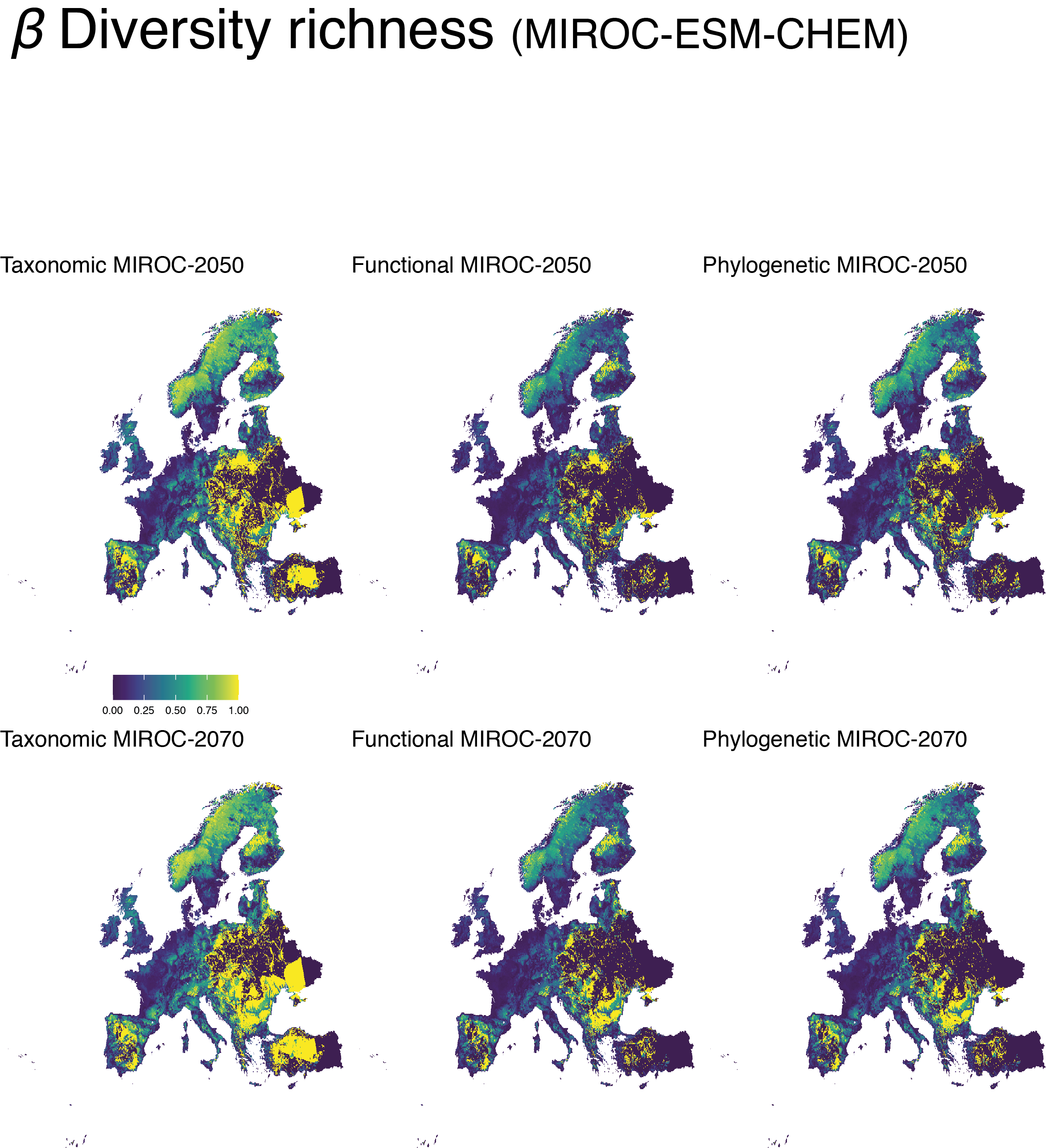


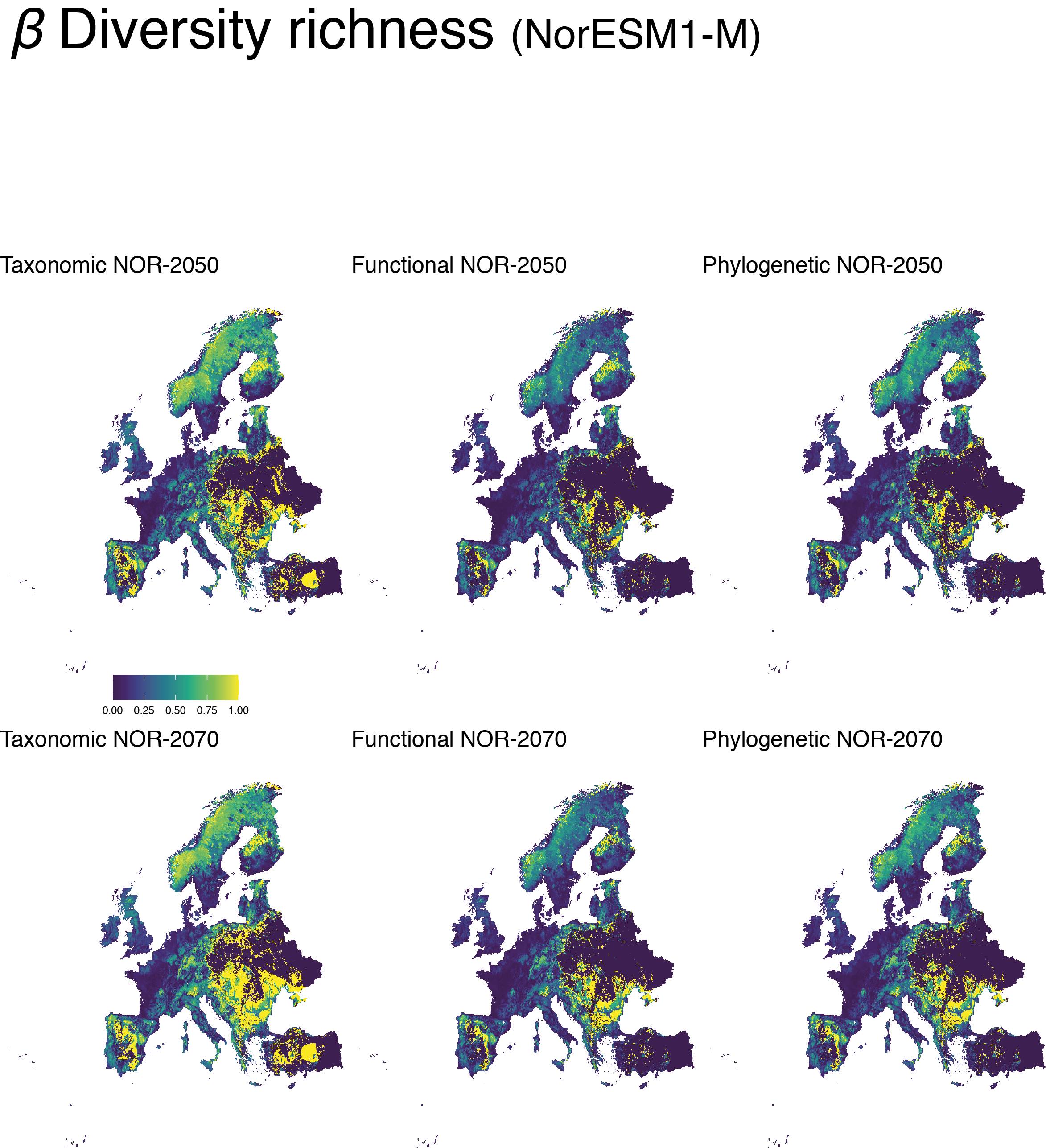

Supplement: Supplementary file 4 — Supplementary material 4 [file 44185_2022_1_MOESM4_ESM.docx]
