# Supplementary material for: Climate change will redefine taxonomic, functional, and phylogenetic diversity of Odonata in space and time
Source: NPJ Biodivers. 2022 Nov 17;1:1. doi: 10.1038/s44185-022-00001-3 (PMC11290607; doi:10.1038/s44185-022-00001-3)
Supplement: Supplementary file 6 — Supplementary material 6 [file 44185_2022_1_MOESM6_ESM.docx]

**Supplementary Material 6**

Reconstruction of ancestral character states for European Odonata per different climate scenarios (BCC-CSM1-1; MIROC-ESM-CHEM; NorESM1-M), time periods (current; 2050; 2070), and three selected characters (variation in habitat suitability; altitudinal shift; centroid shift).

**Odonata (Order)**

**
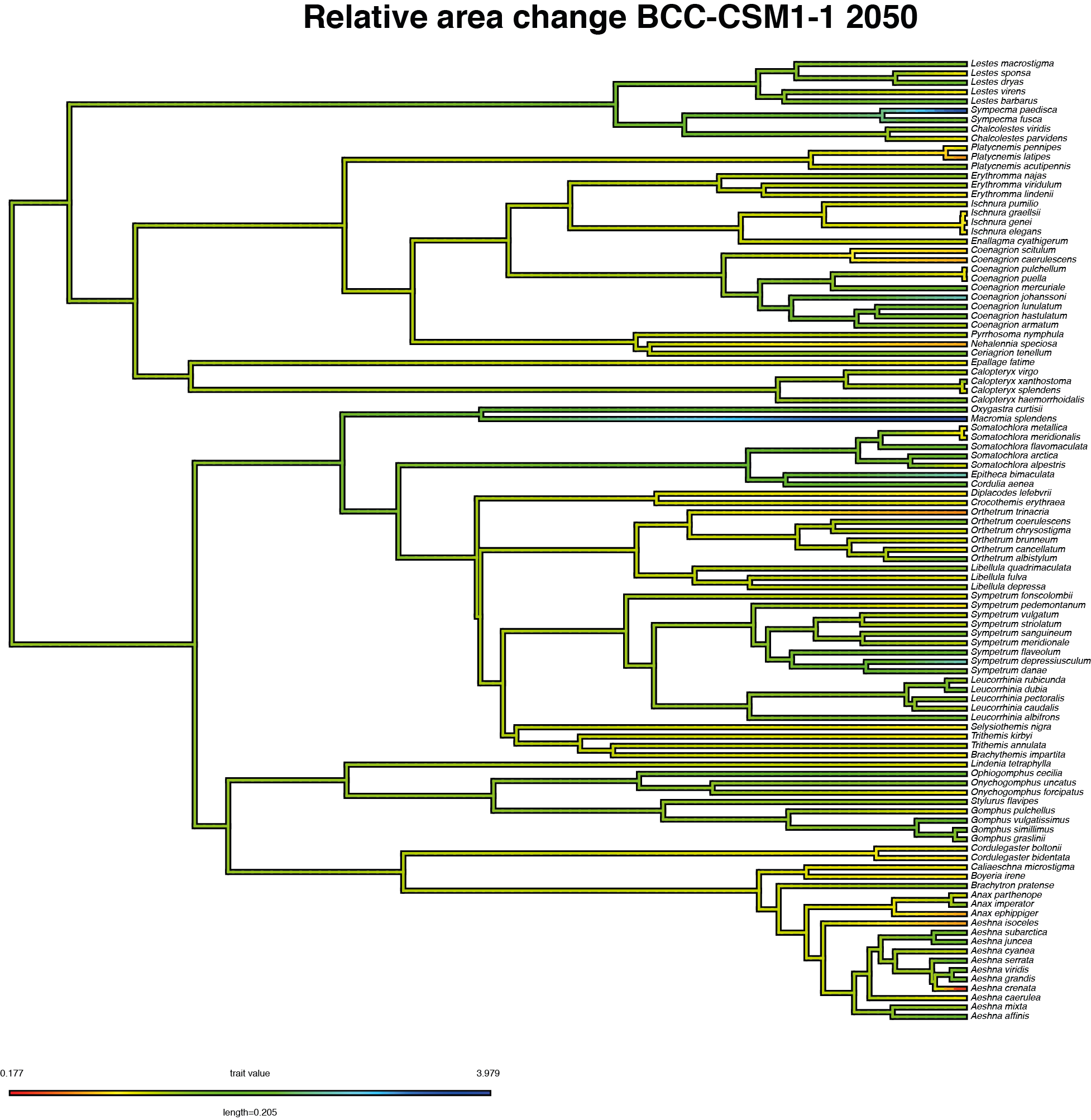
**


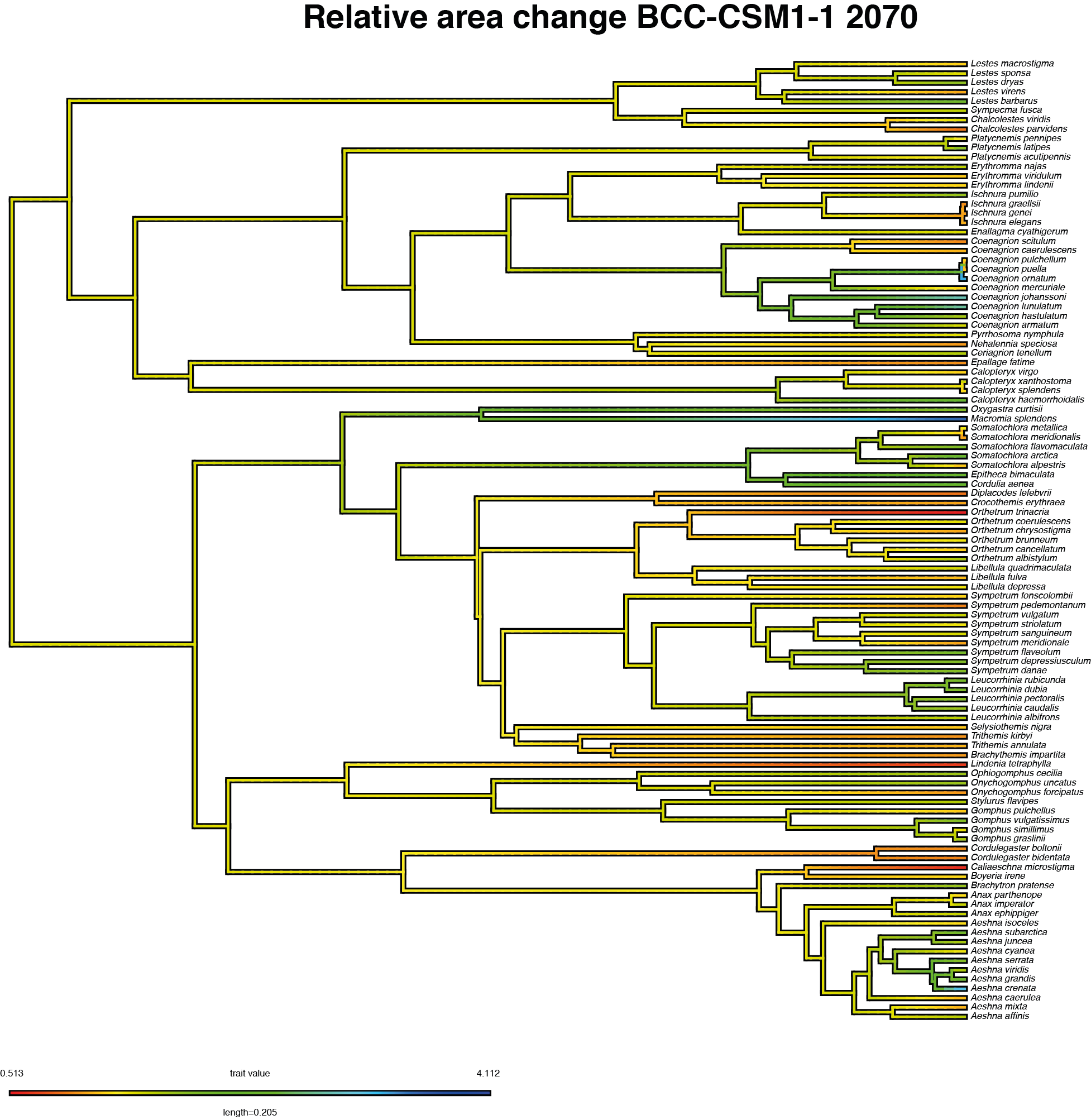


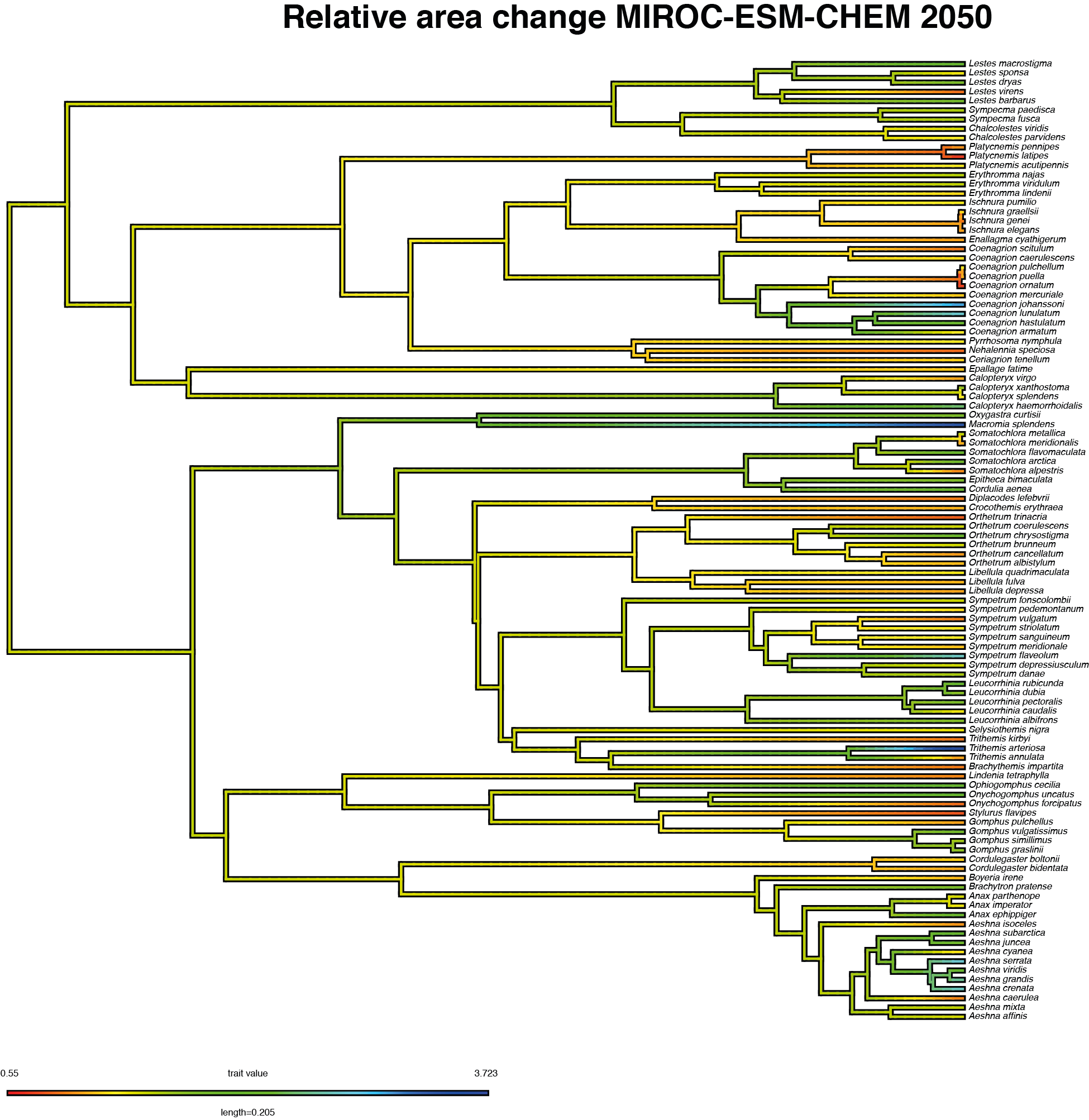


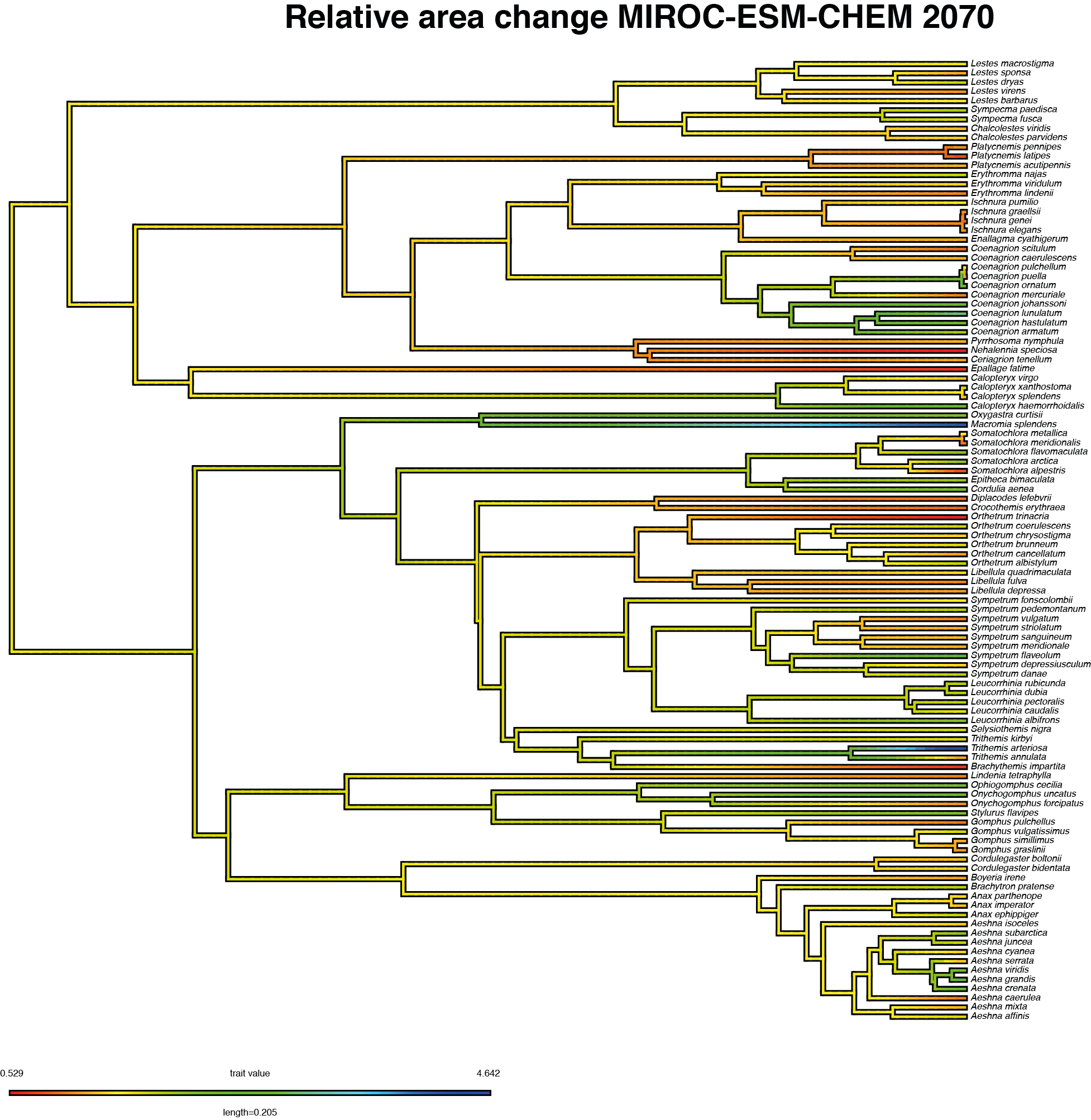


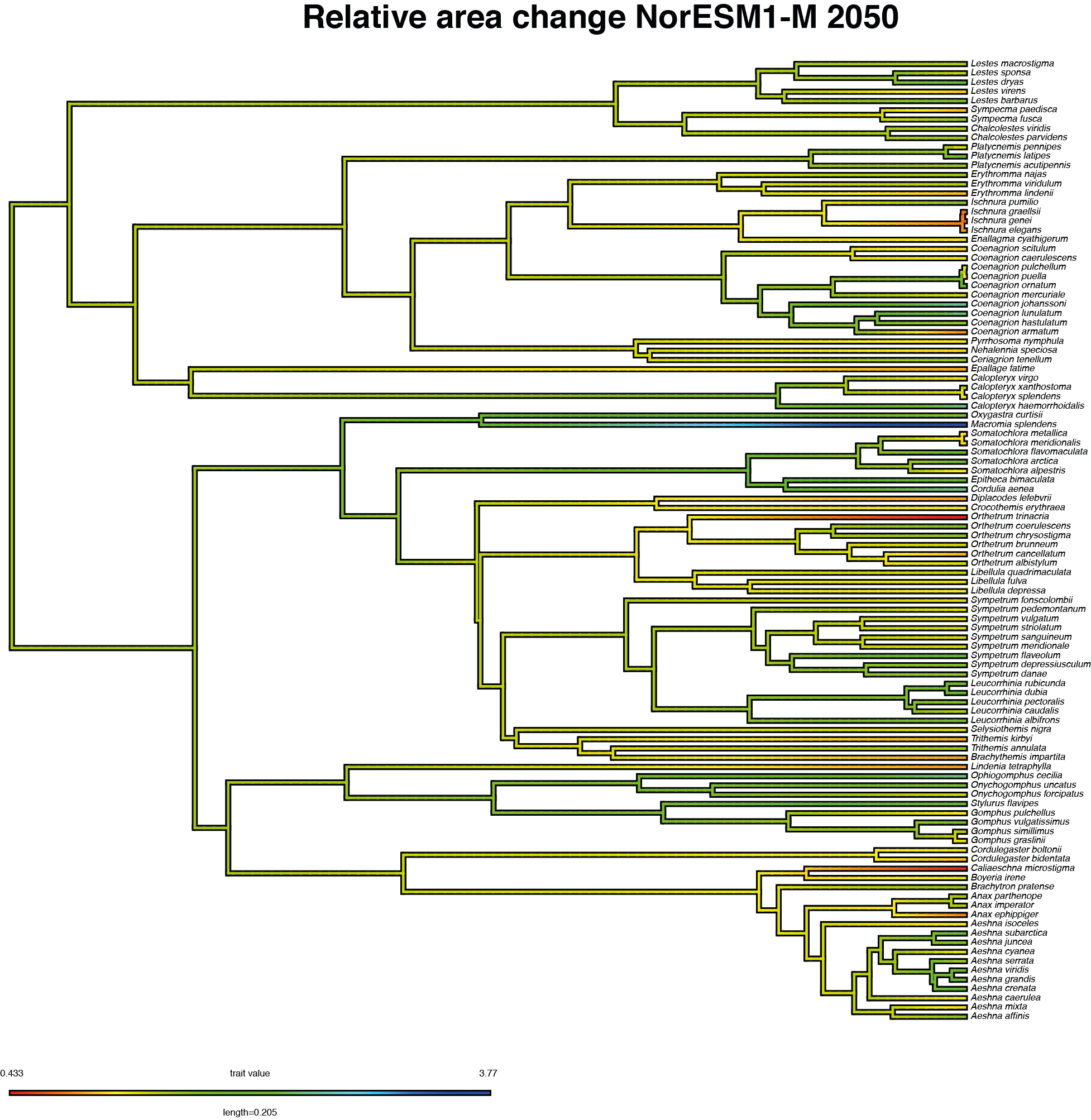


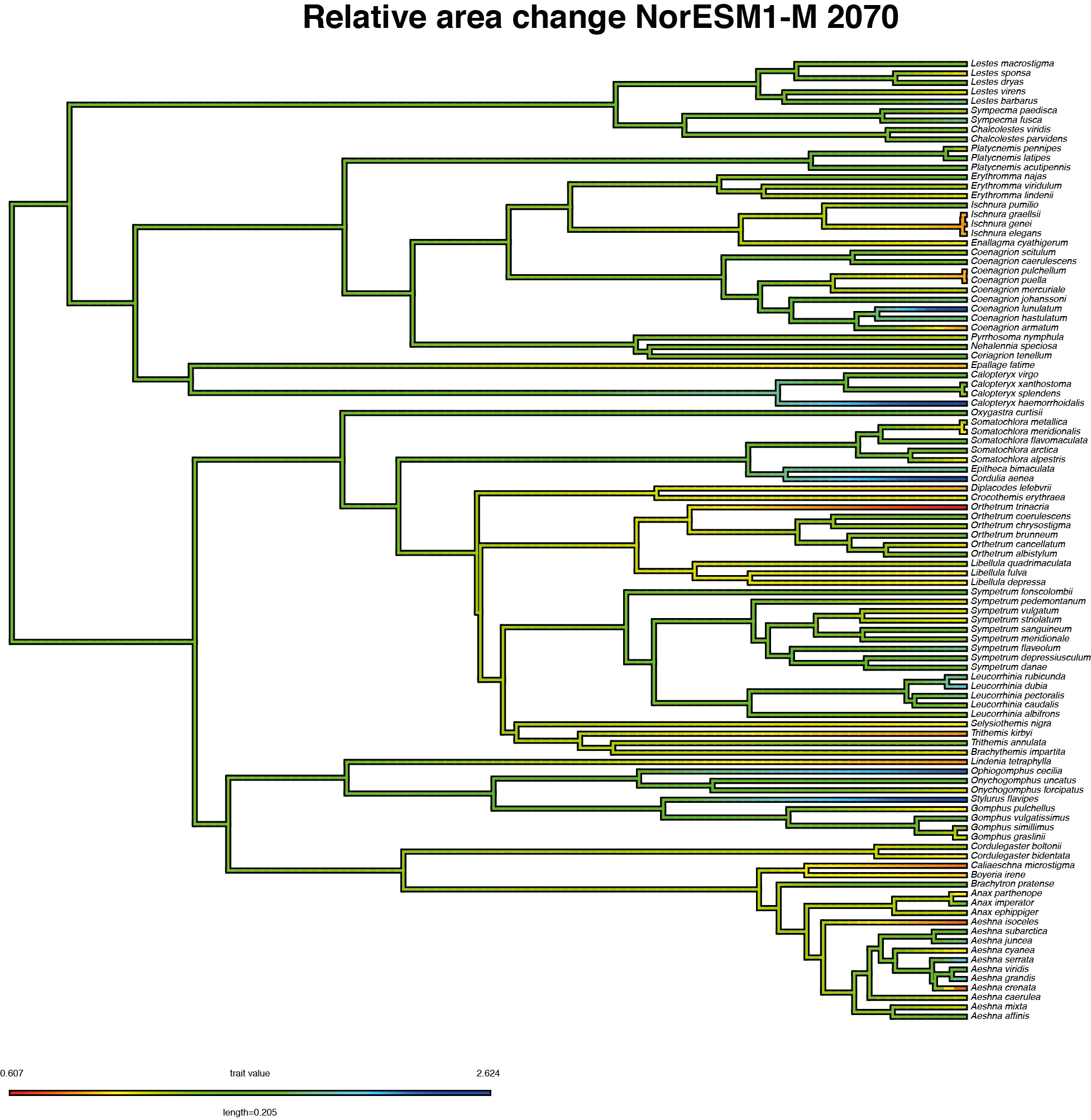


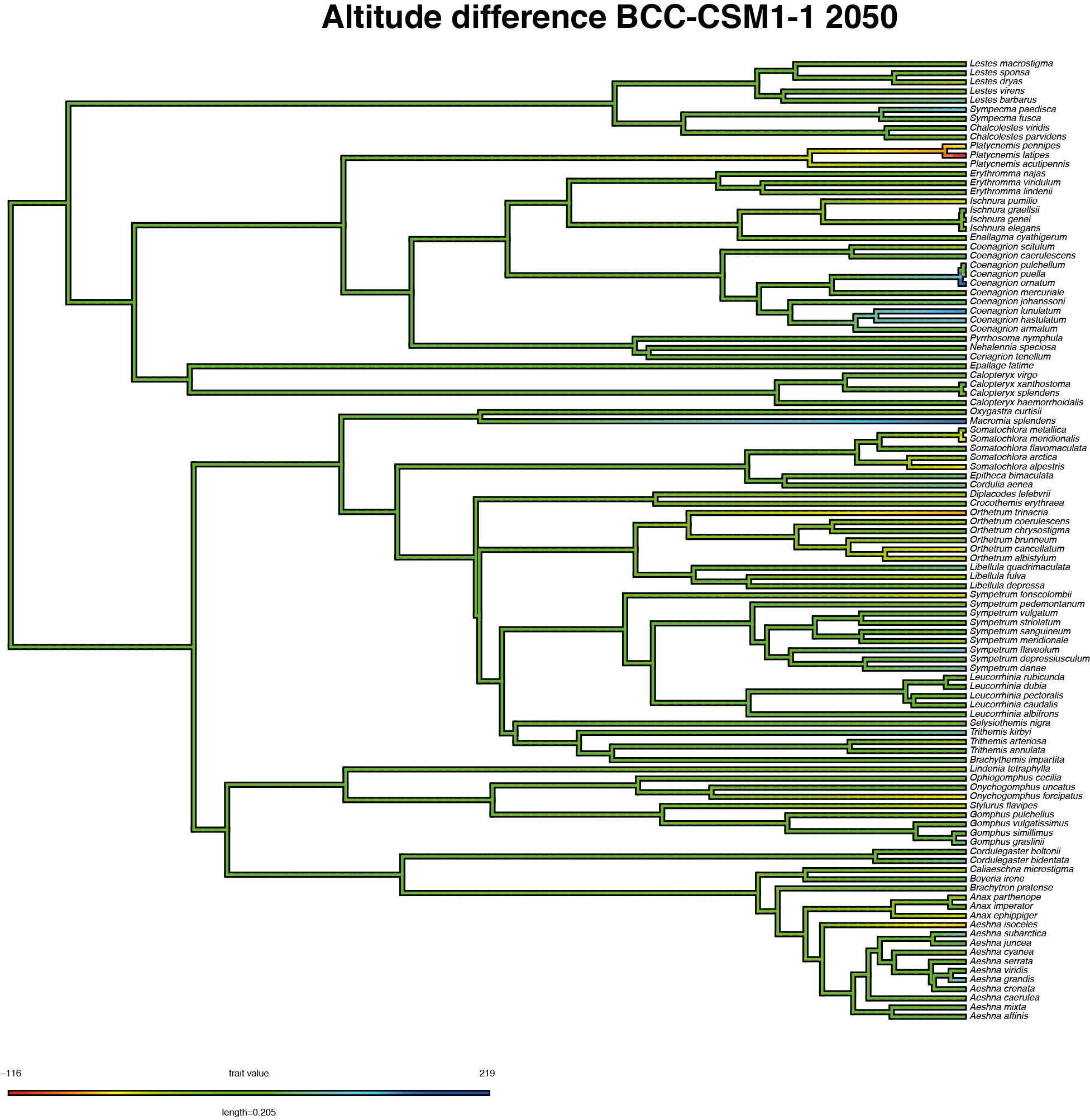


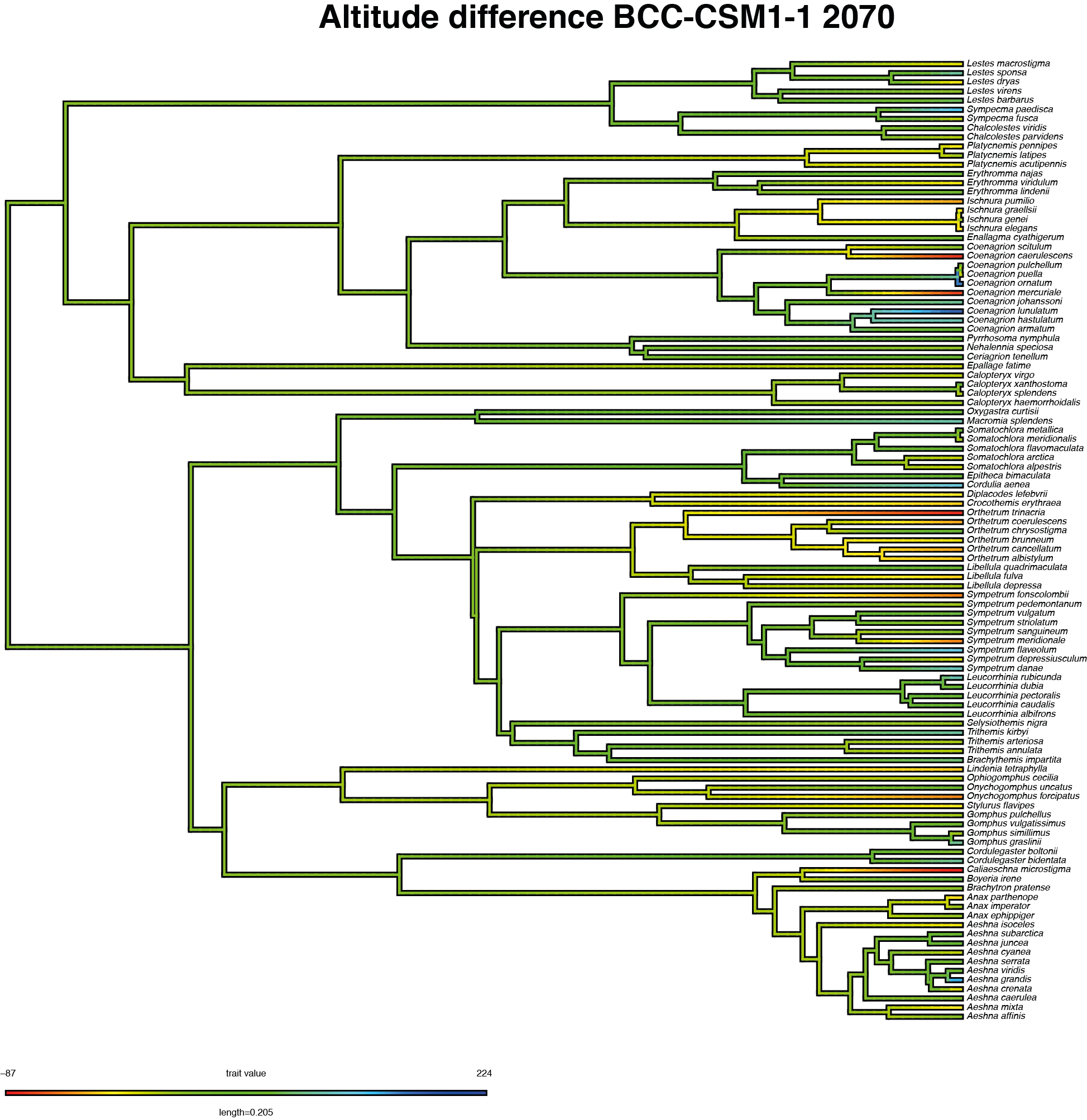


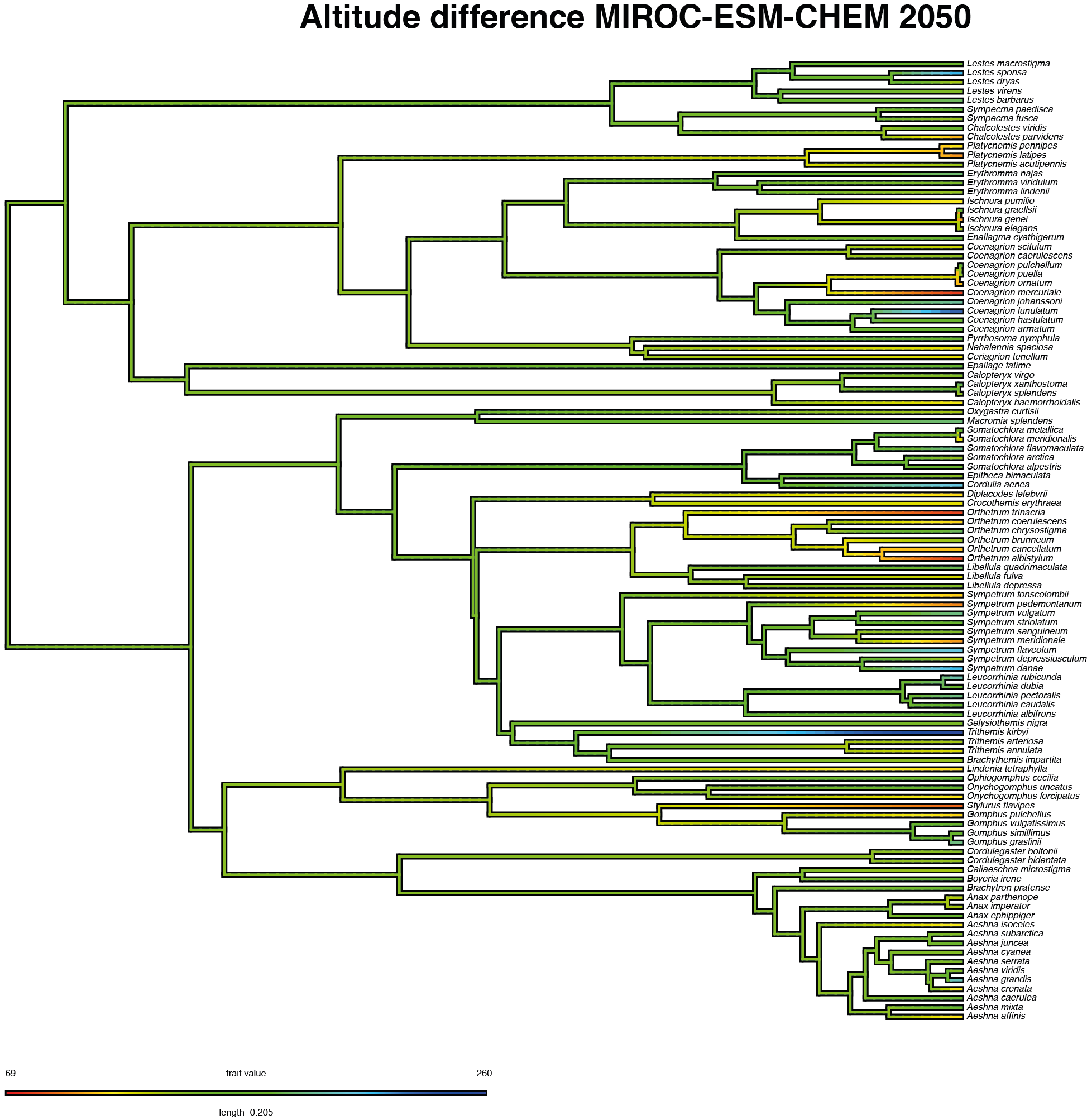


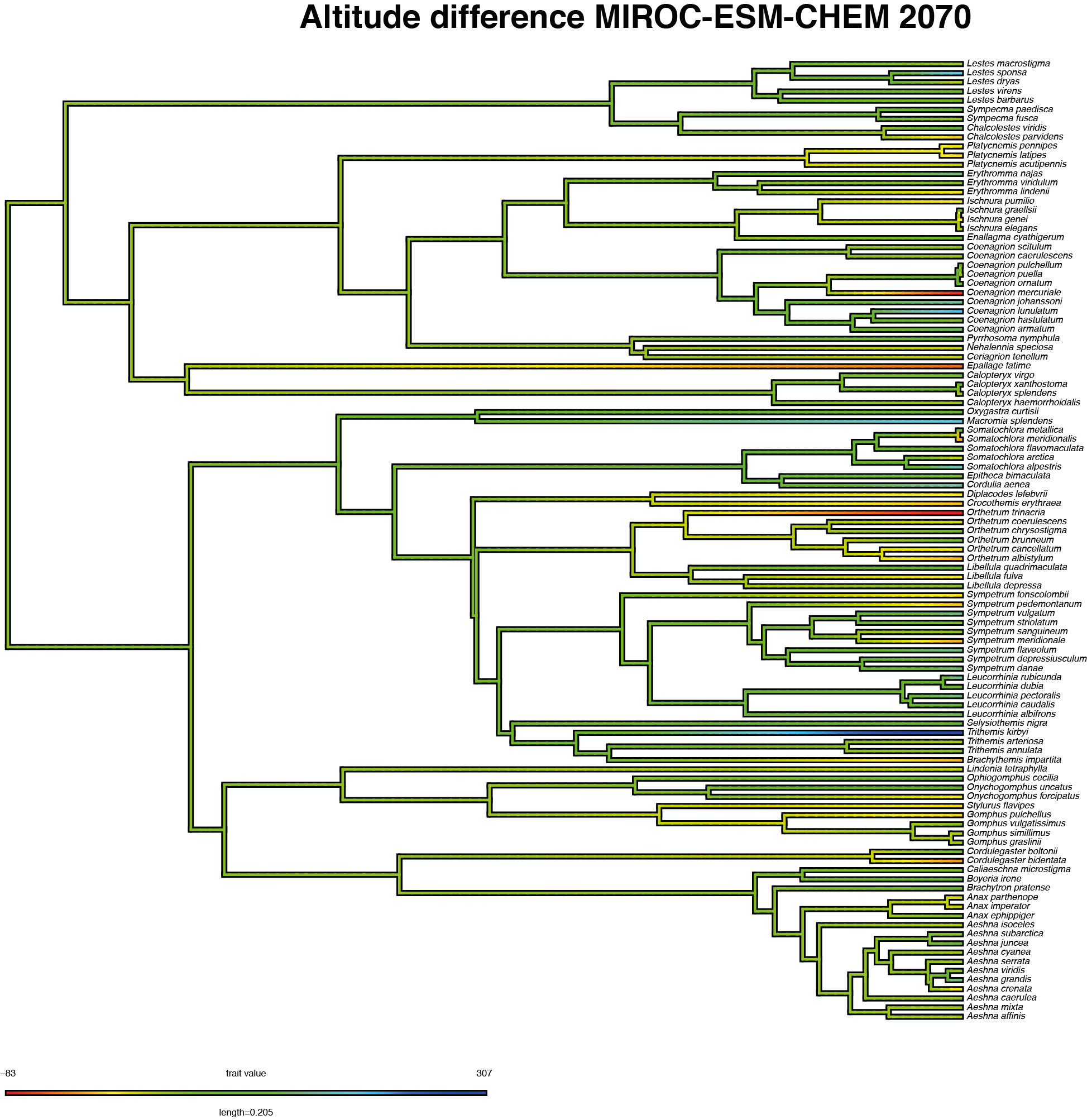


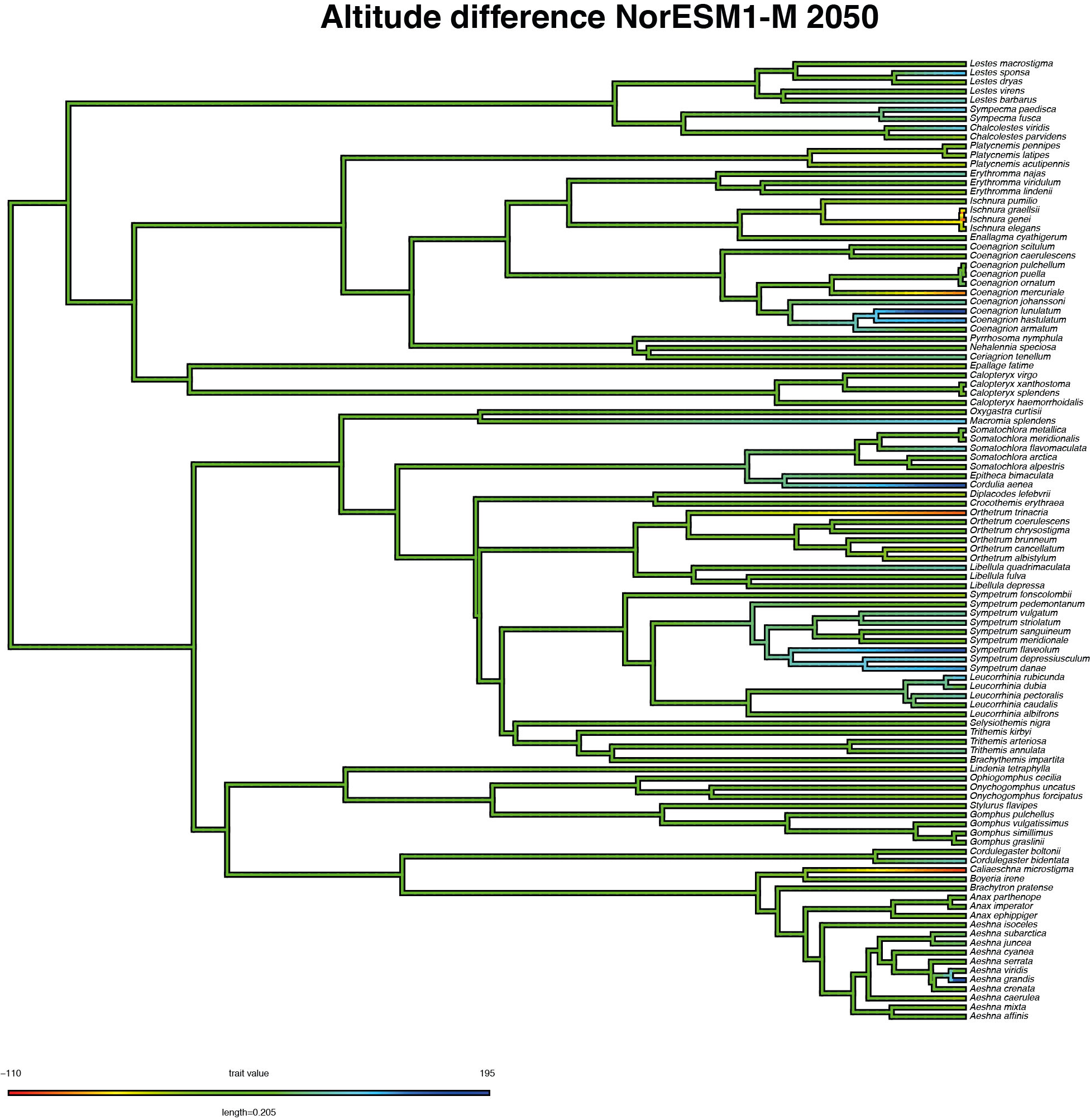


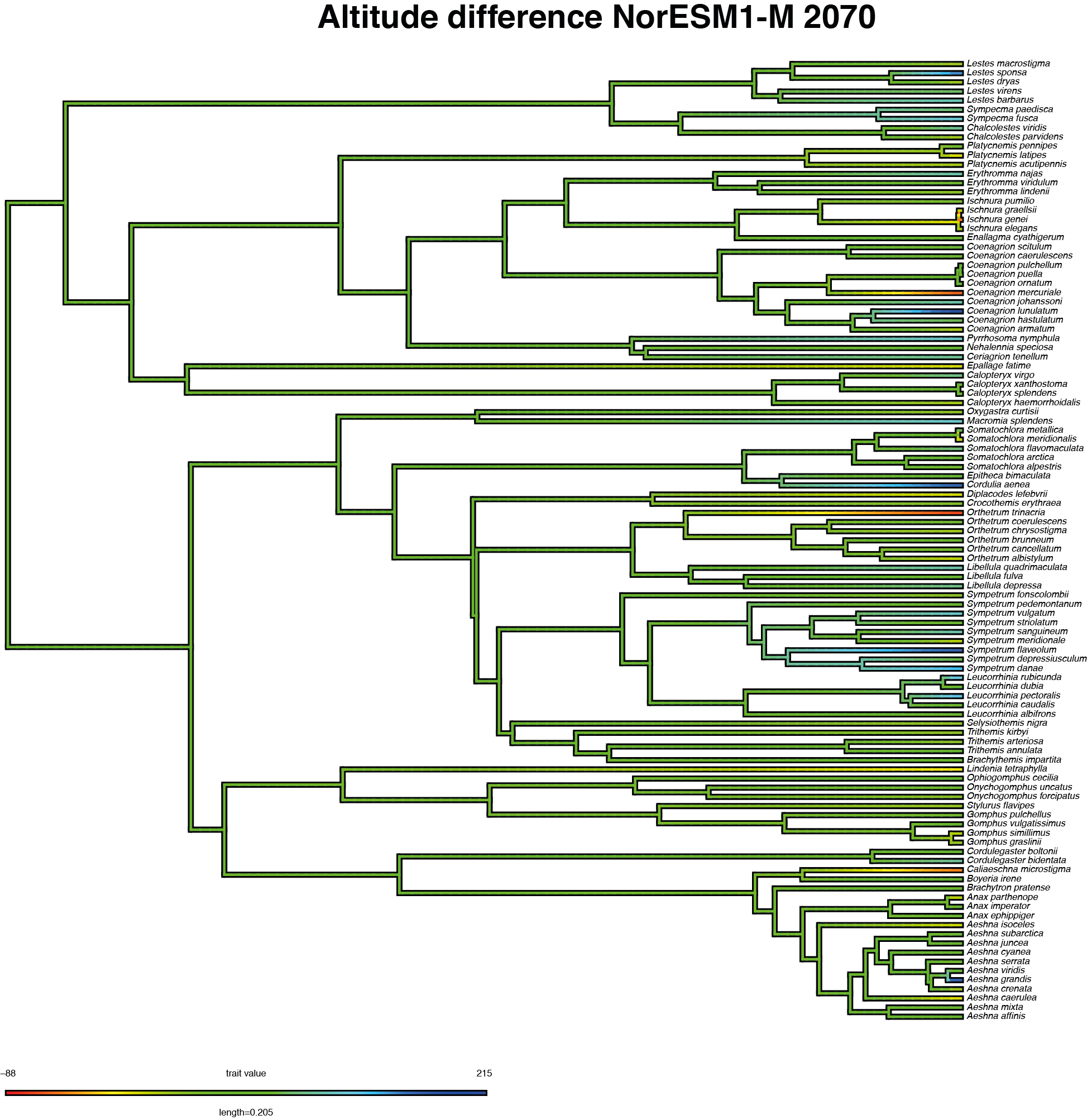


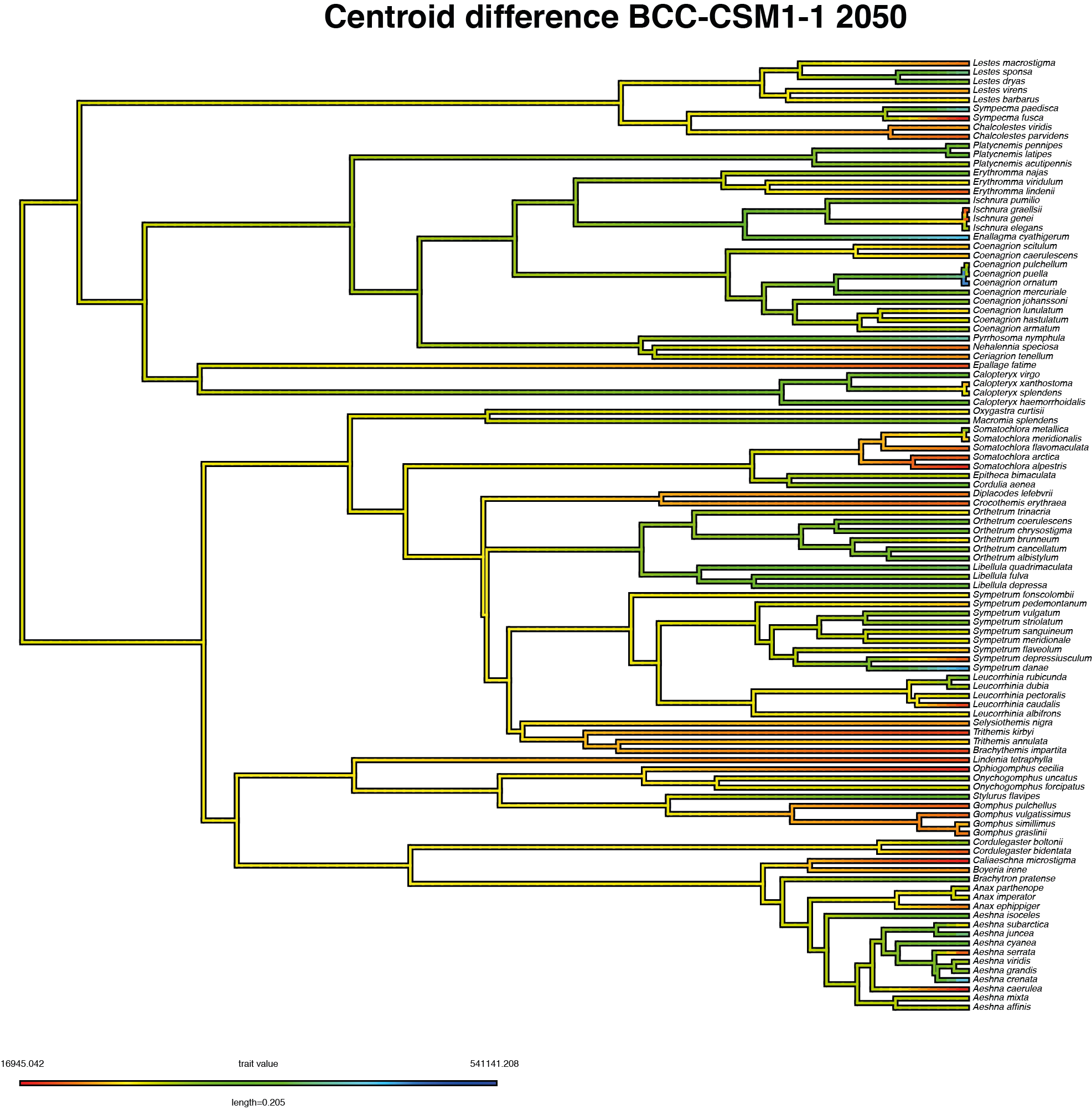


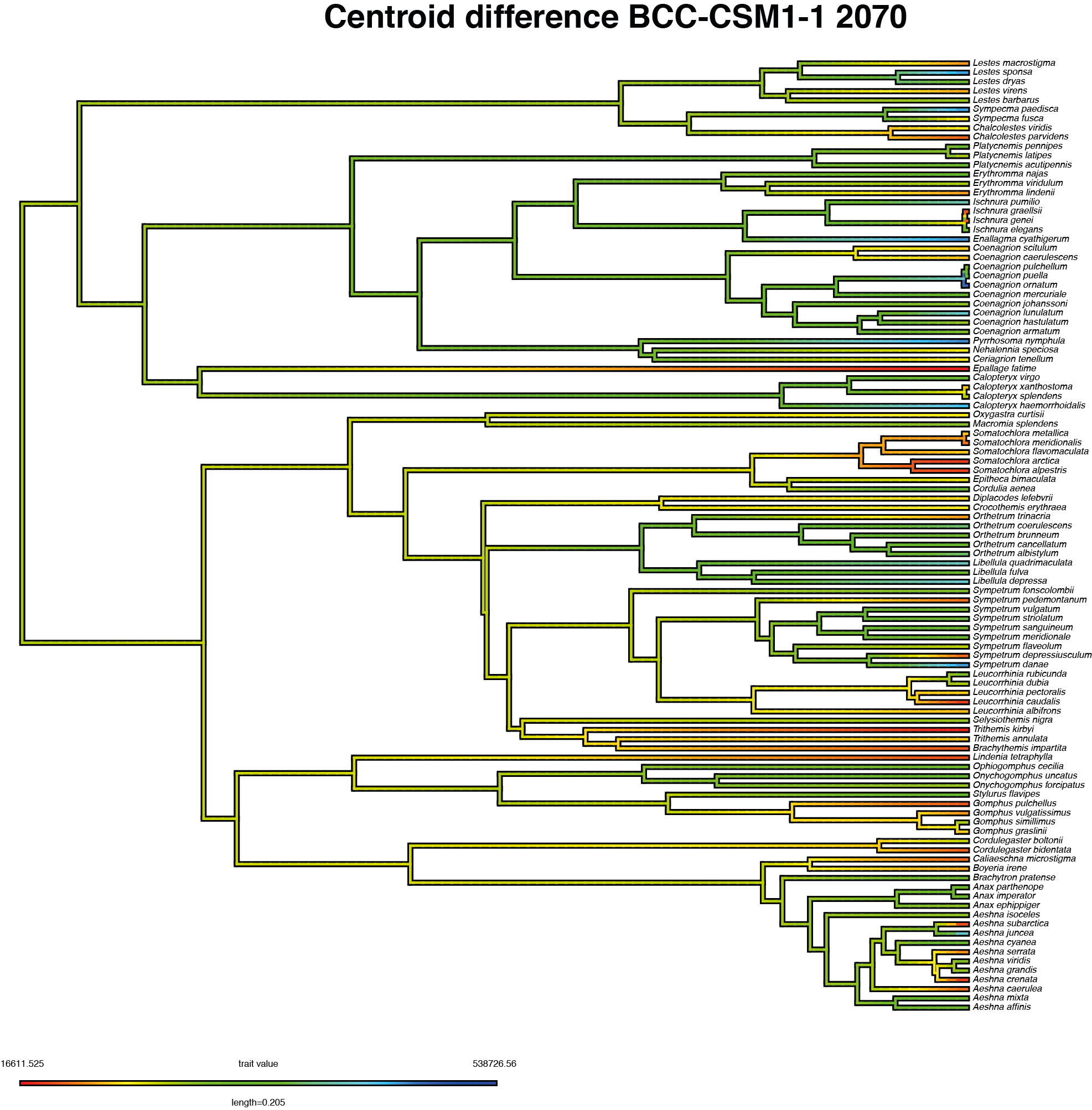


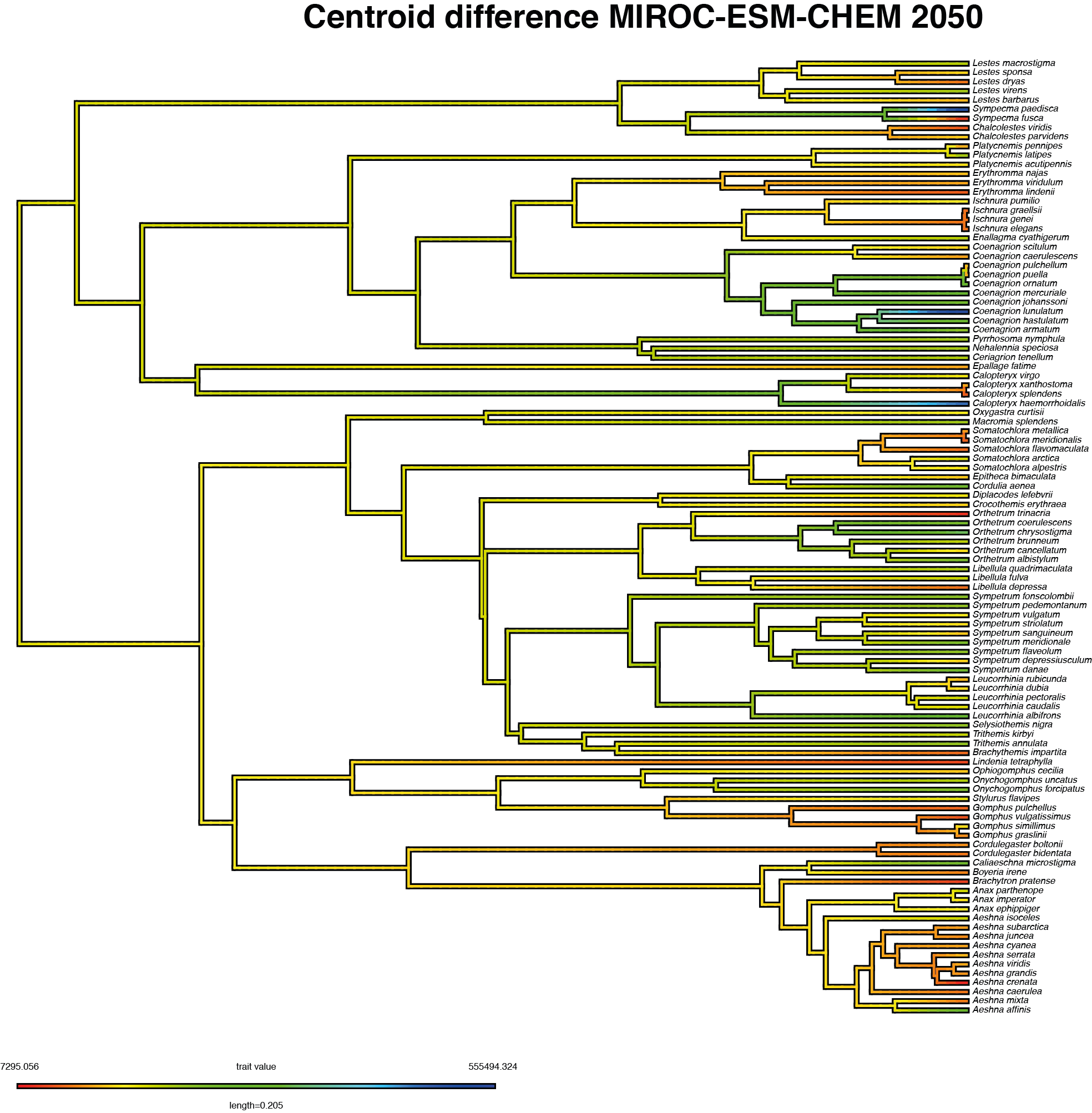


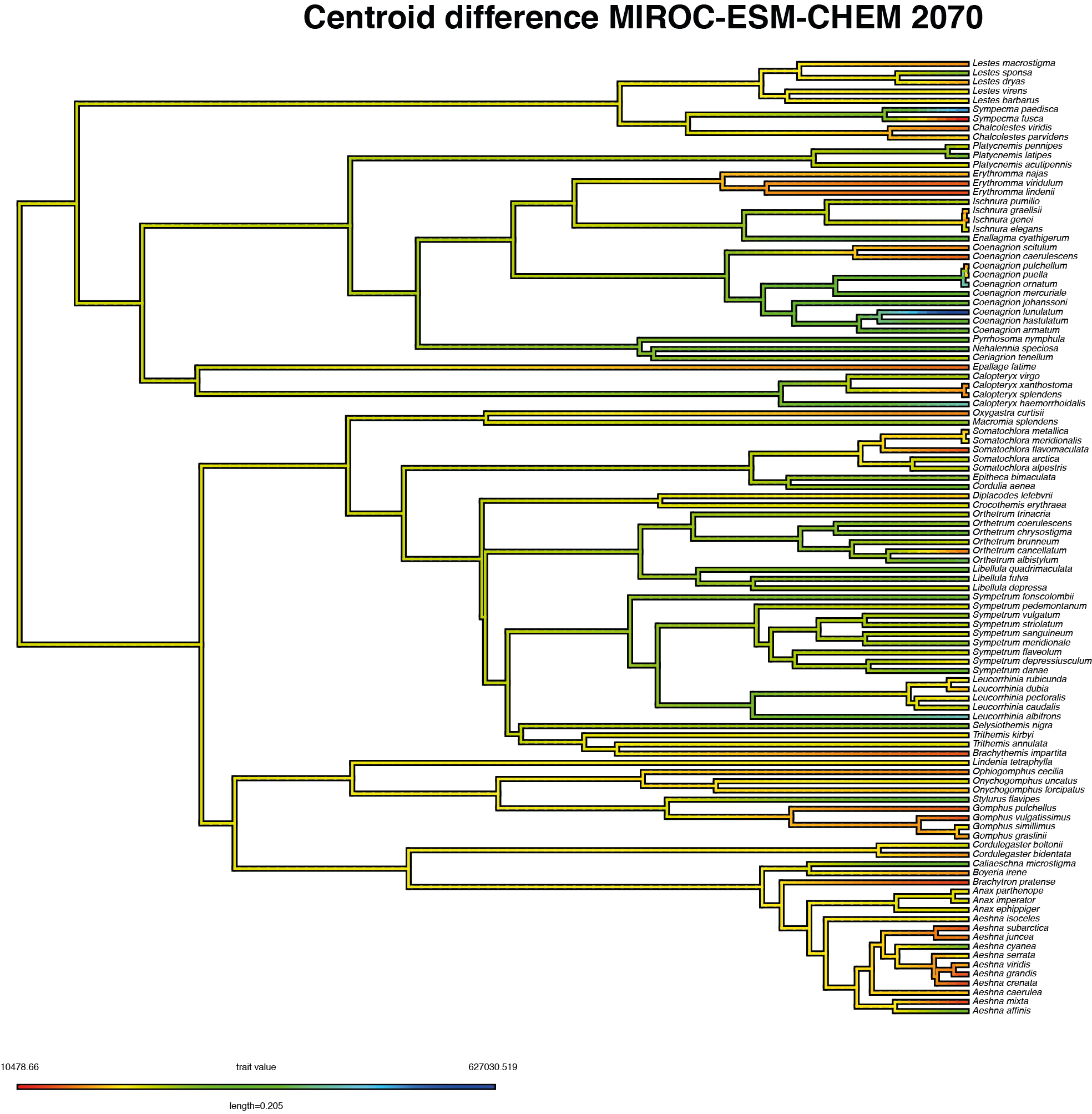


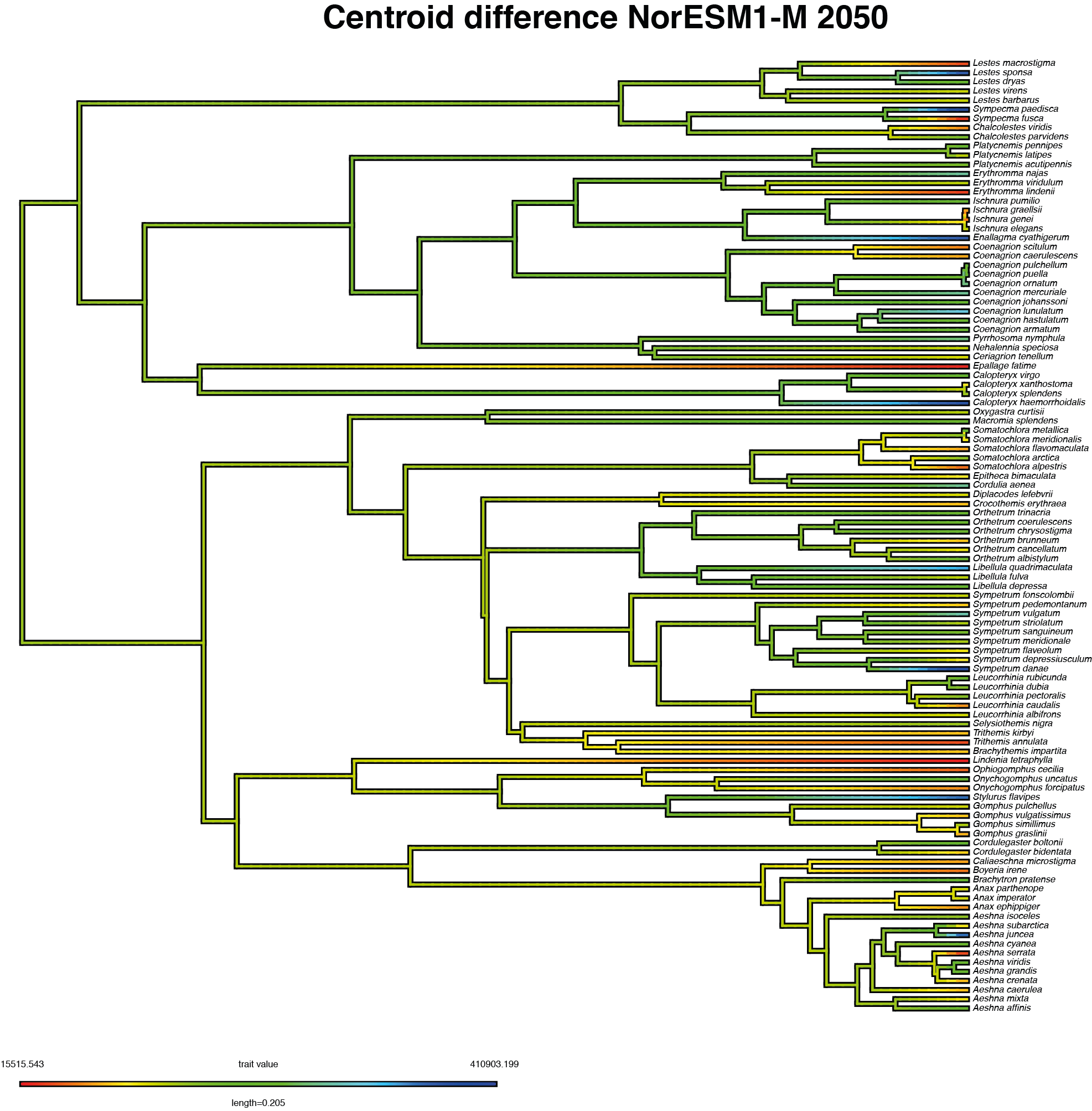


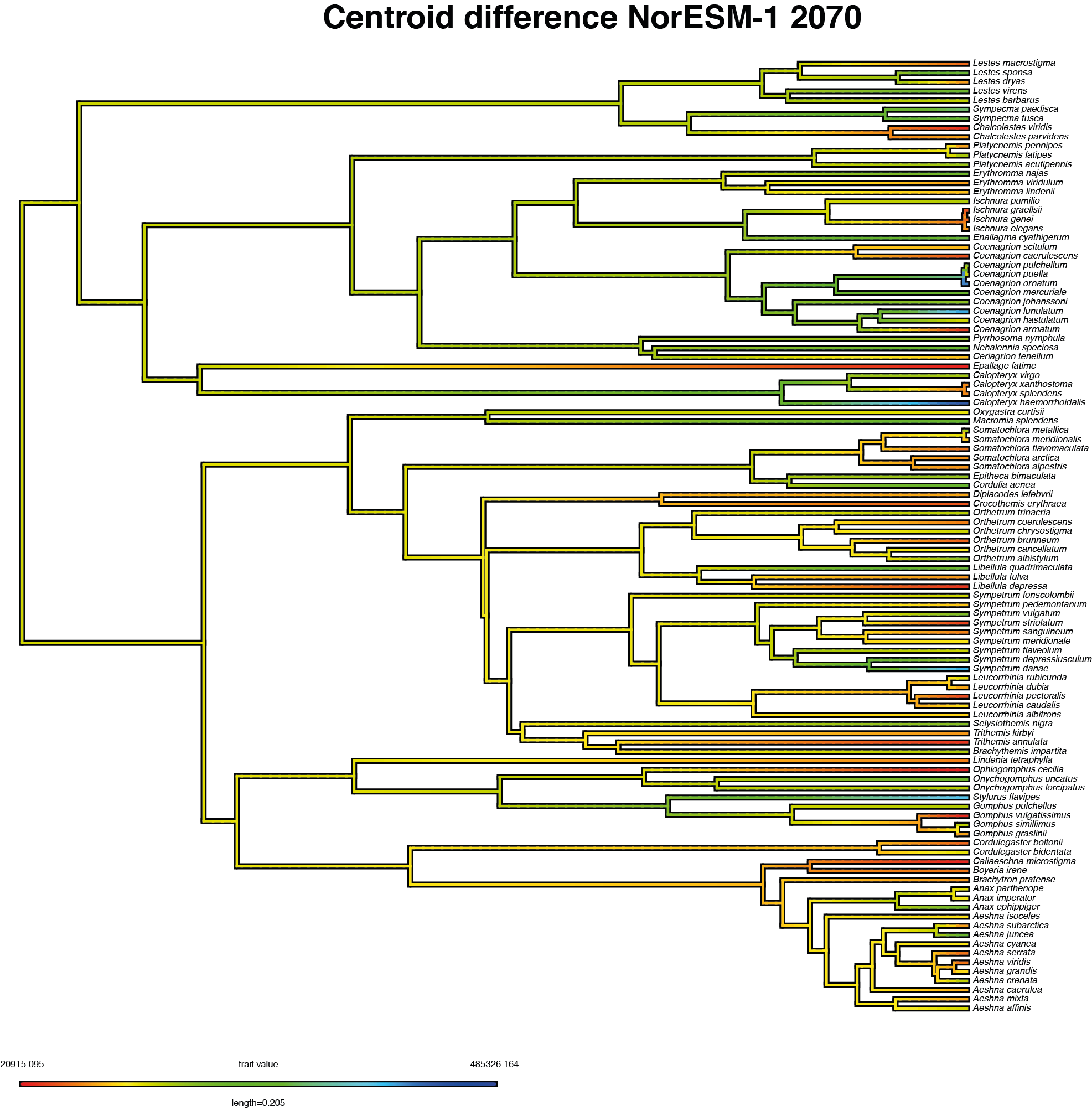


**Anisoptera (Suborder)**

**
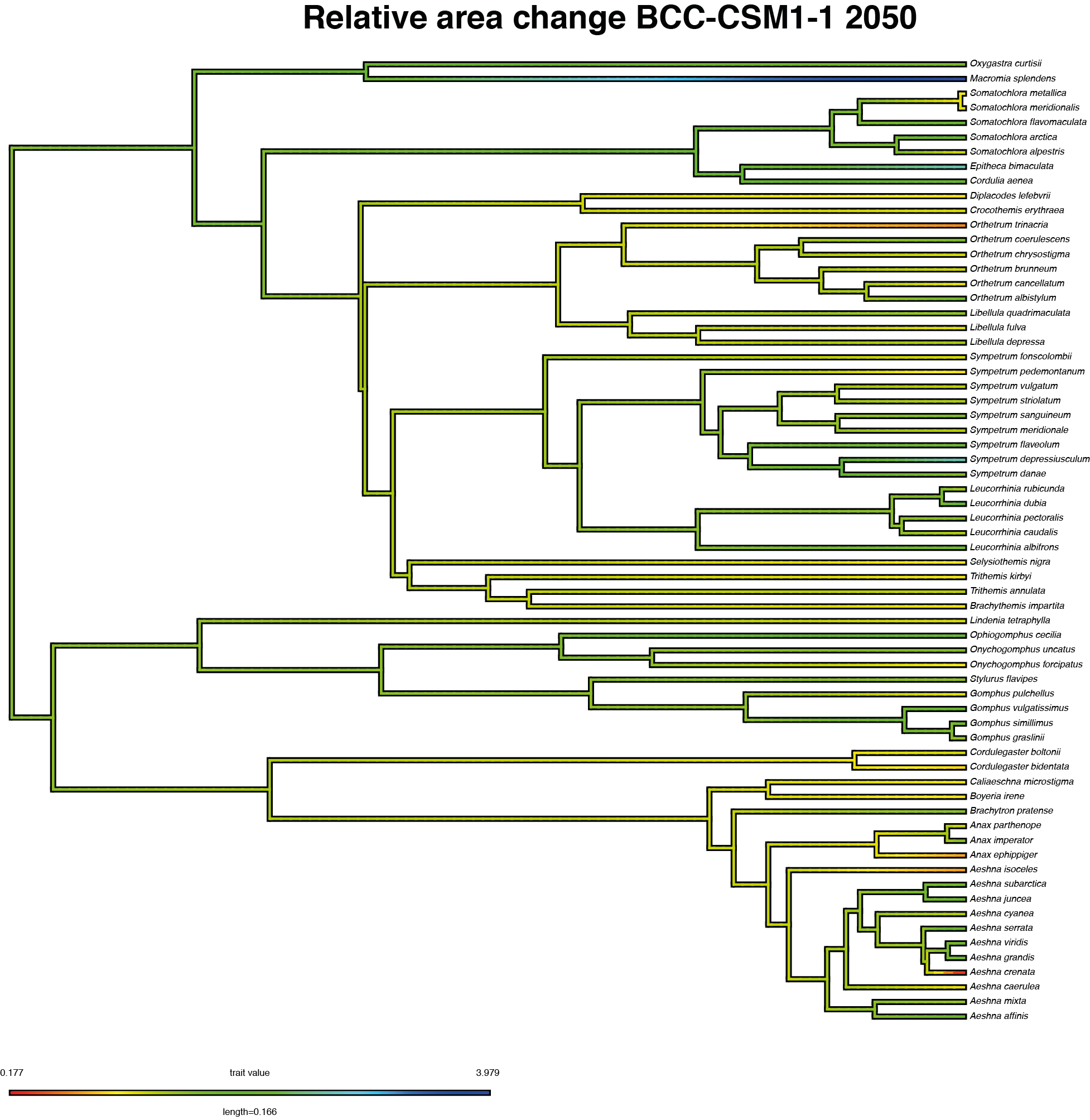
**

**
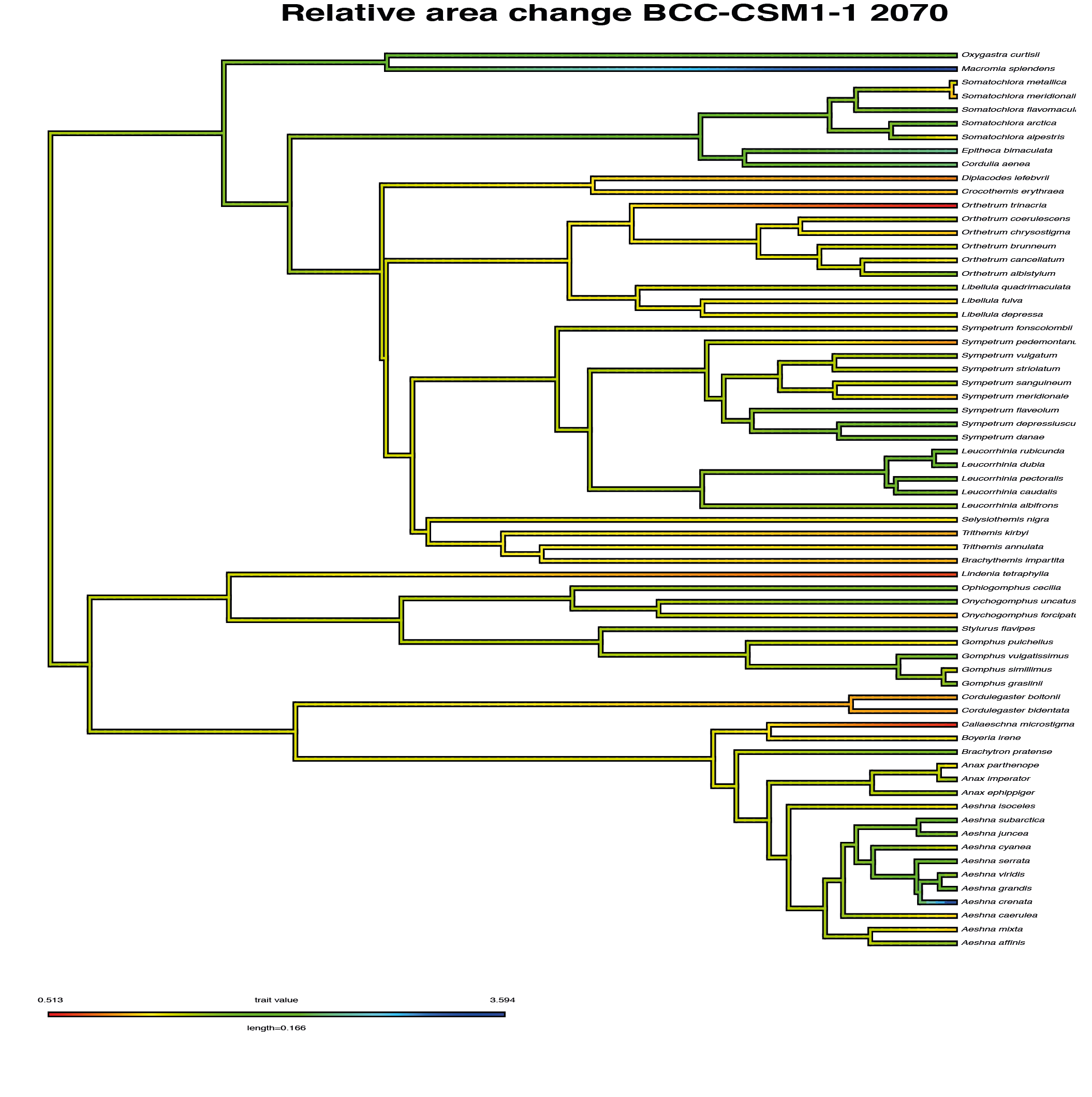
**

**
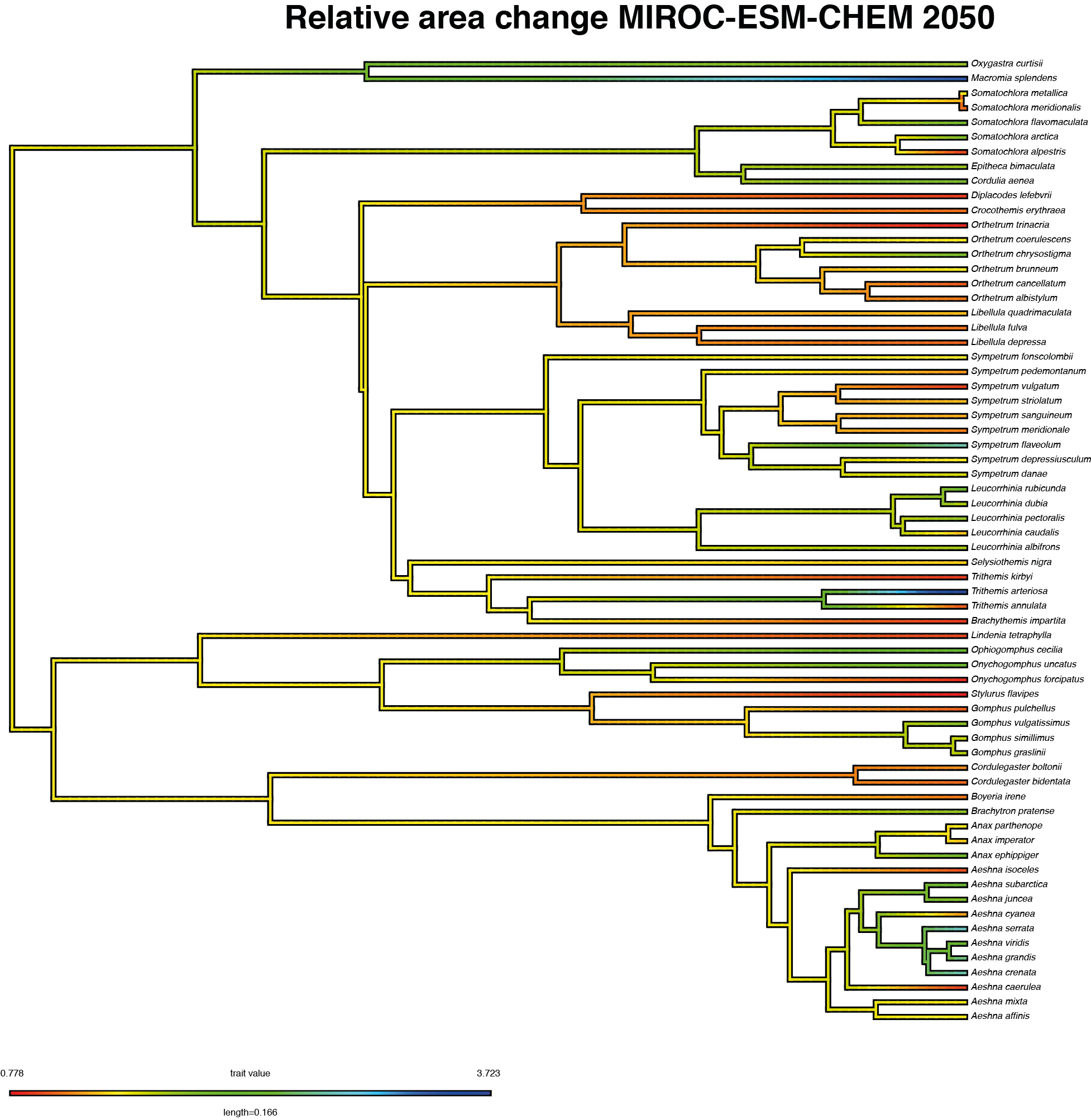
**

**
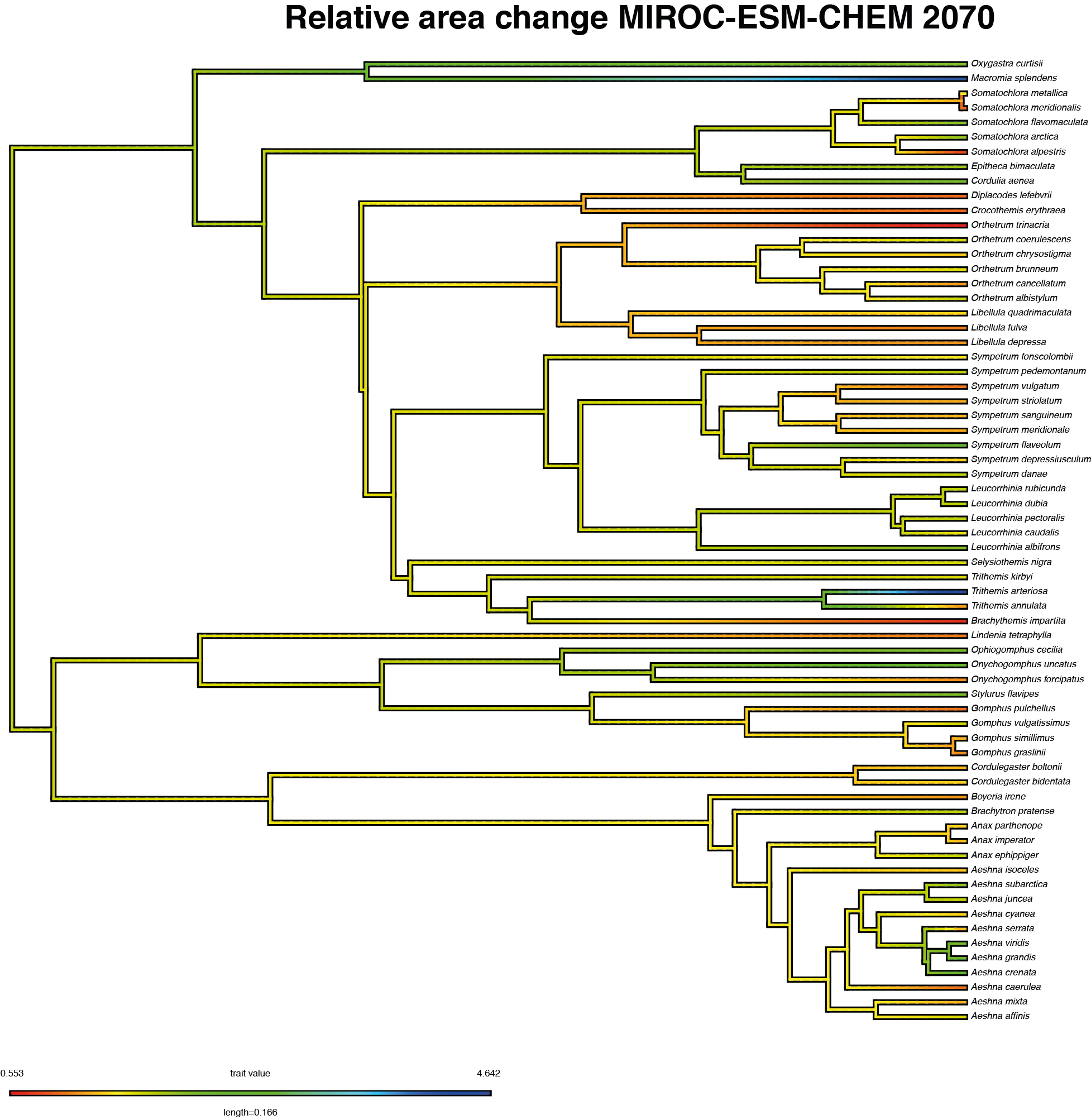
**

**
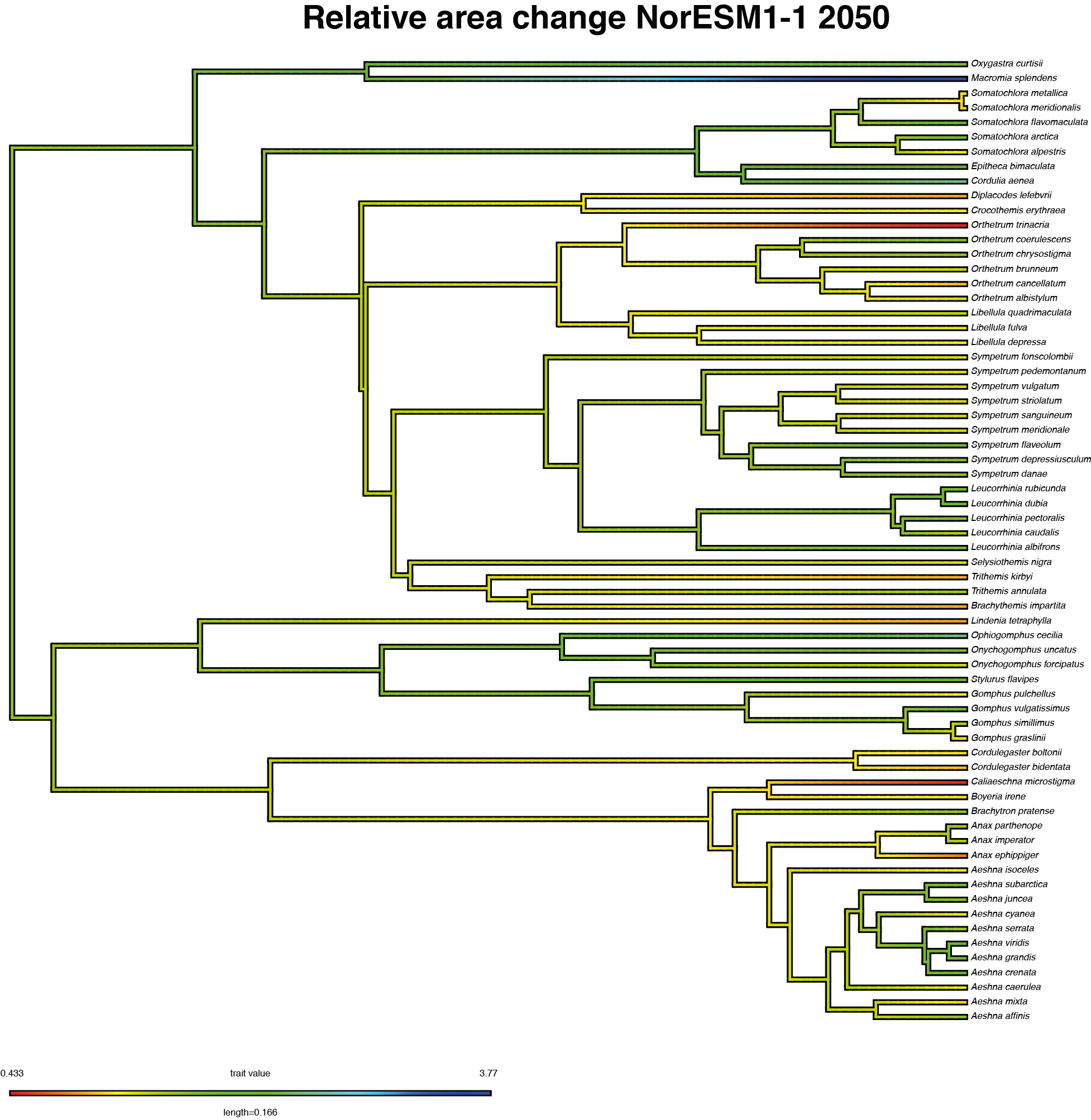
**

**
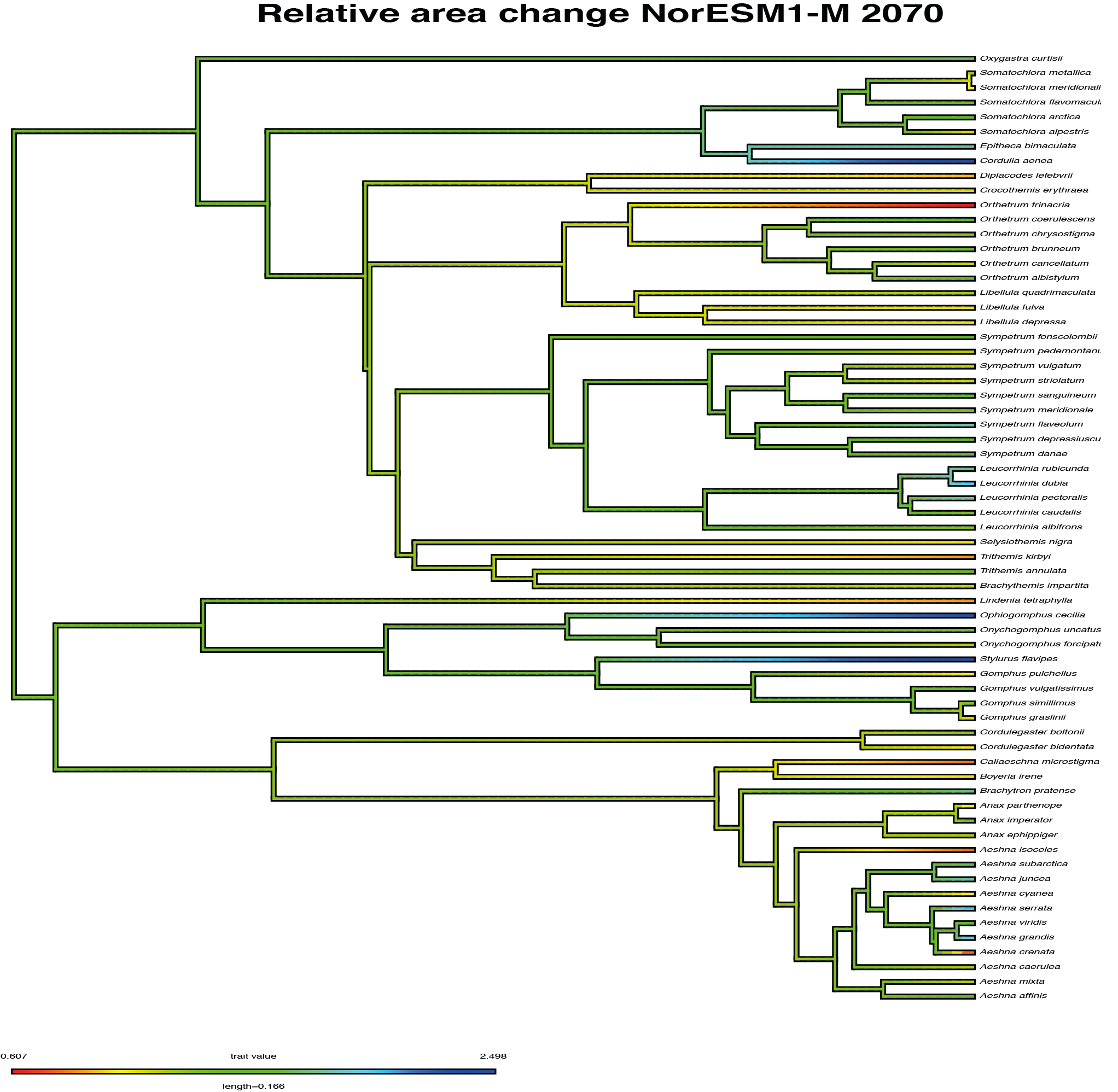
**

**
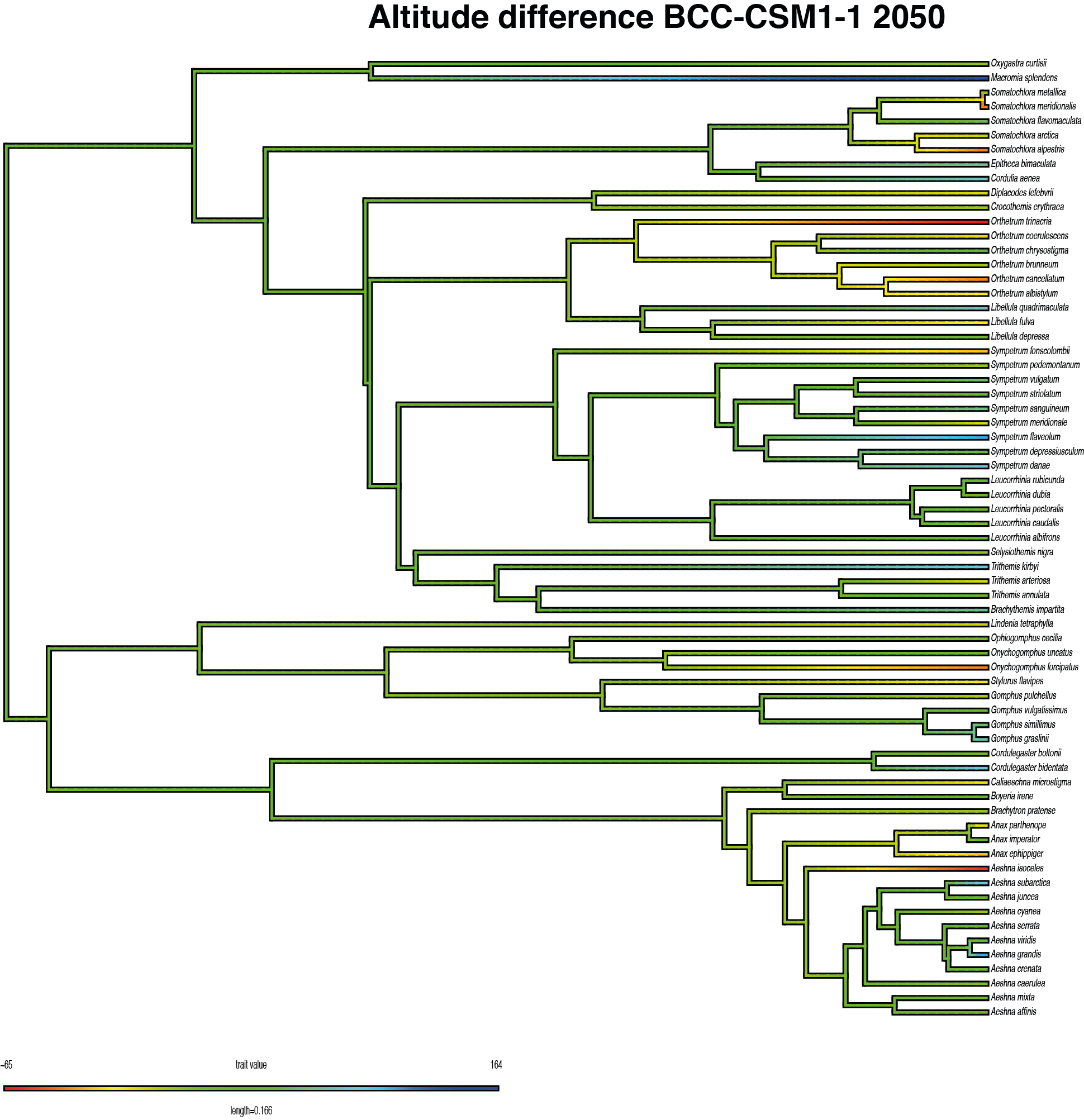
**

**
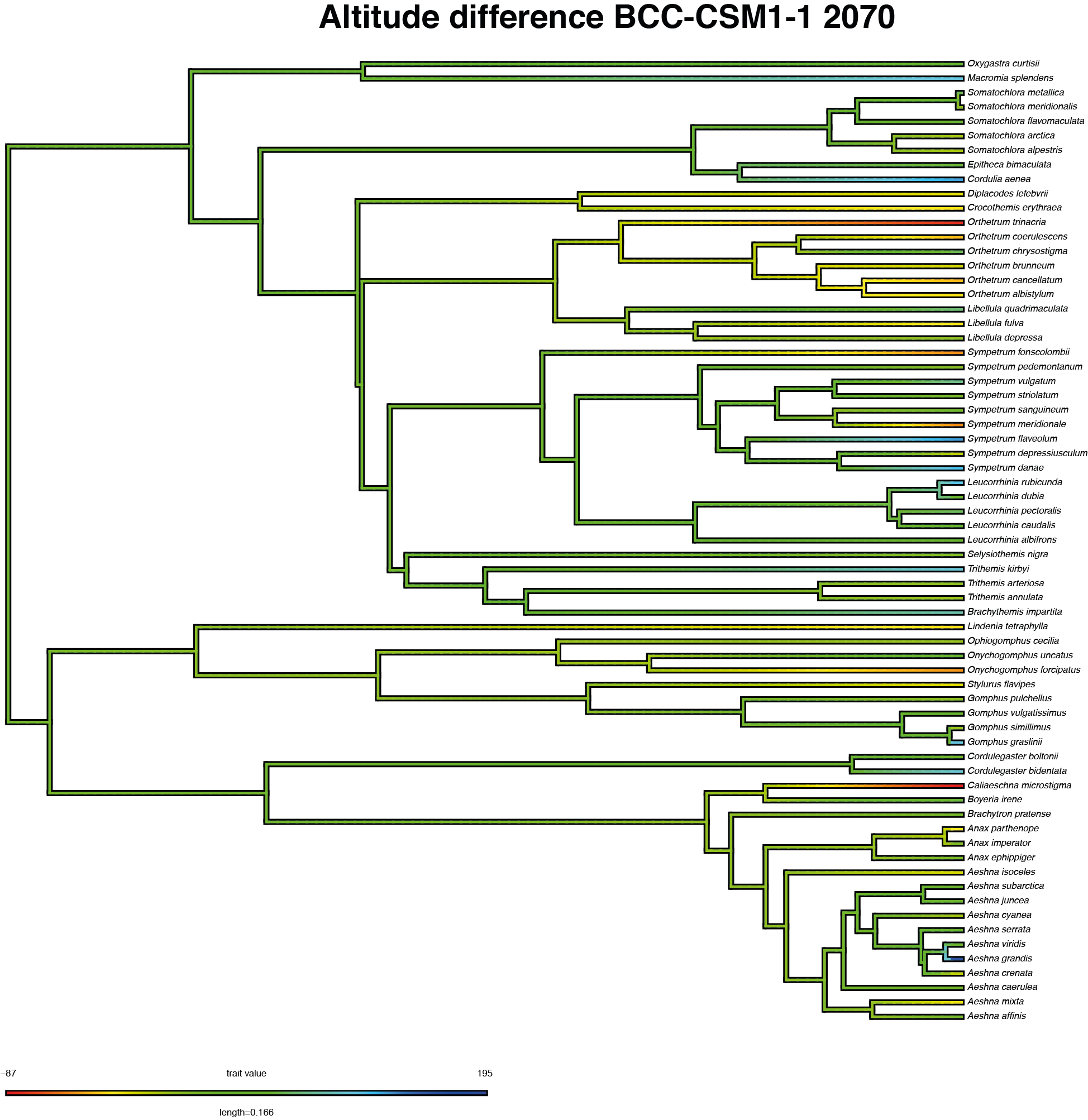
**

**
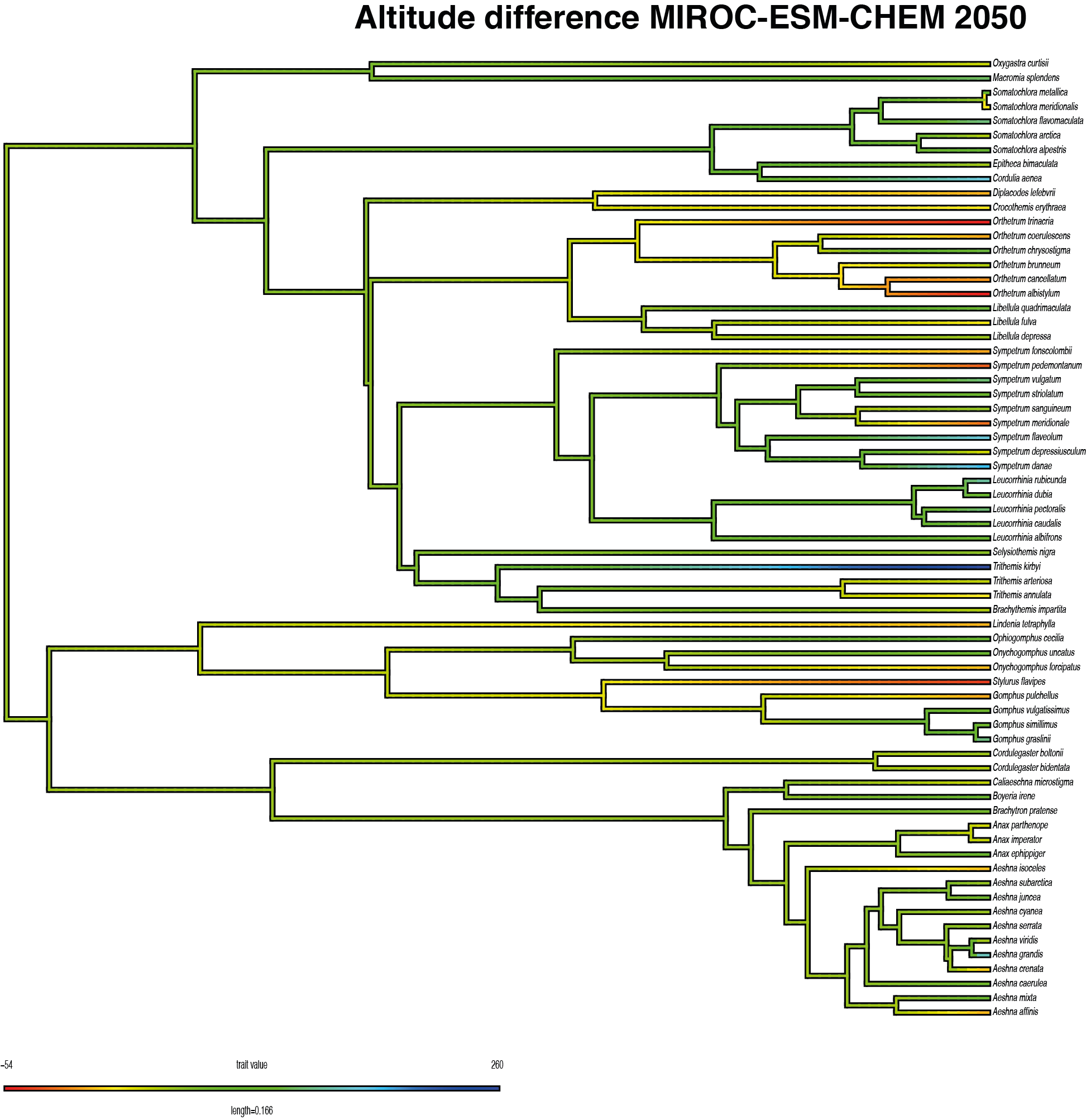
**

**
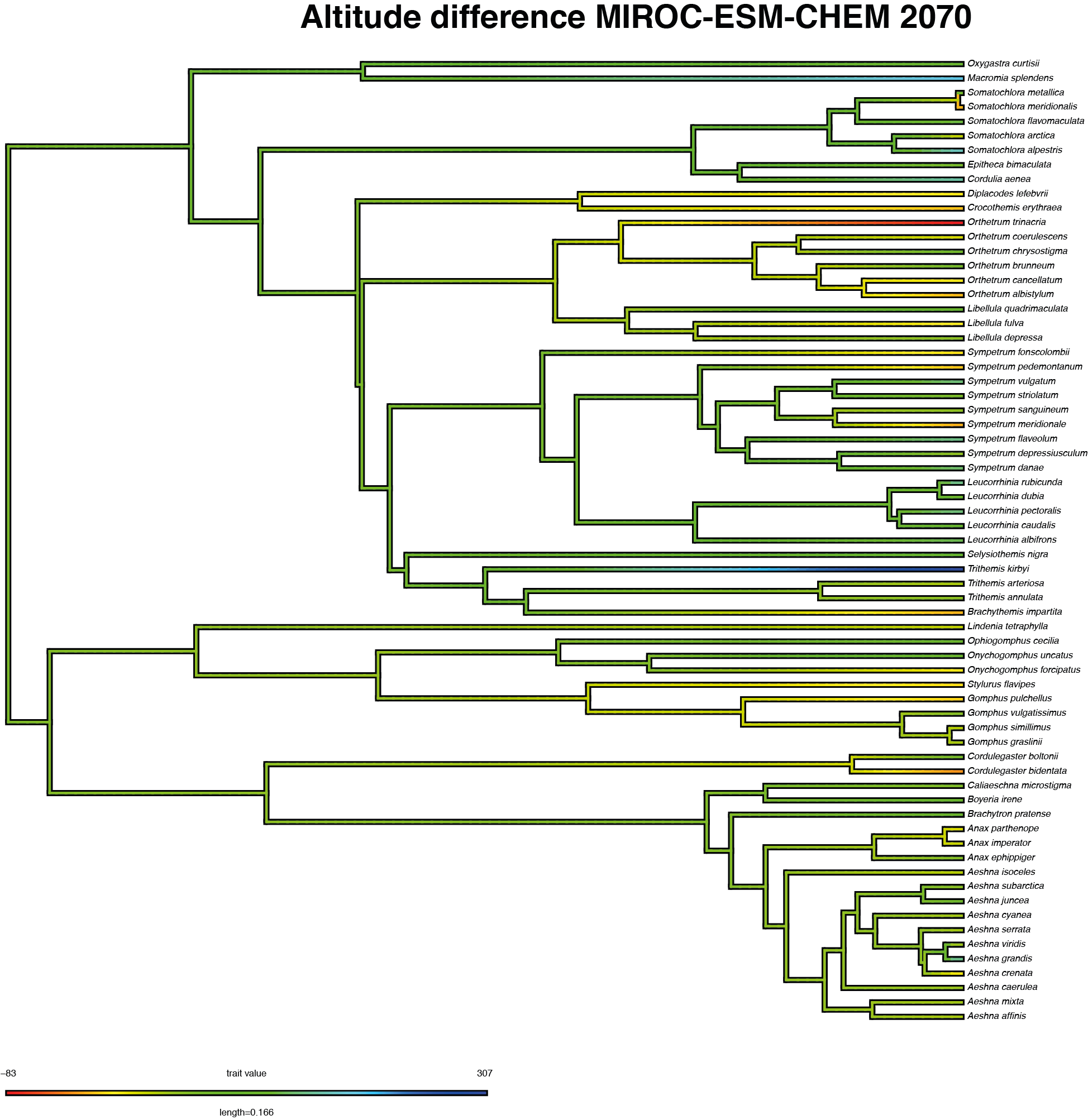
**

**
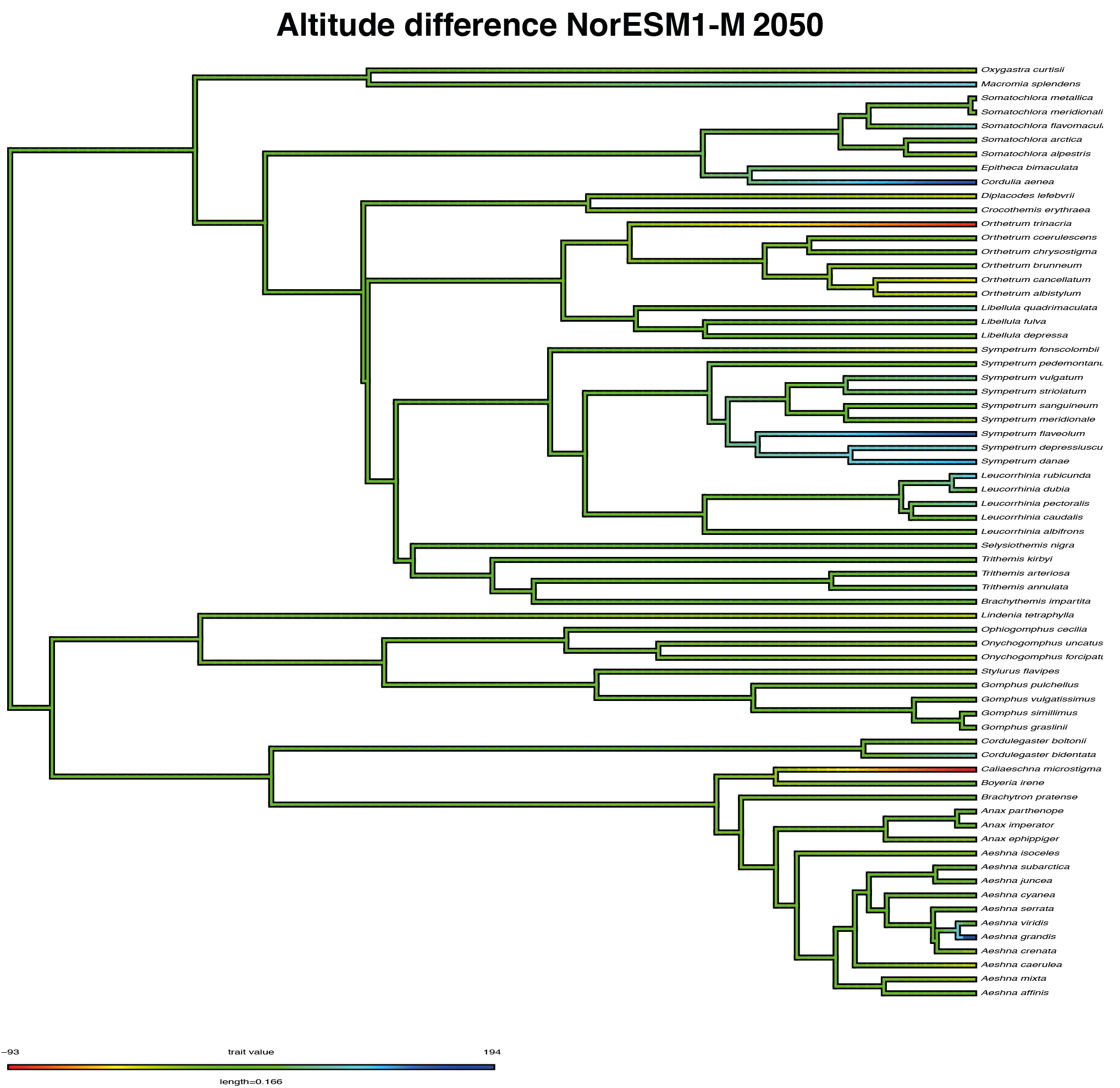
**

**
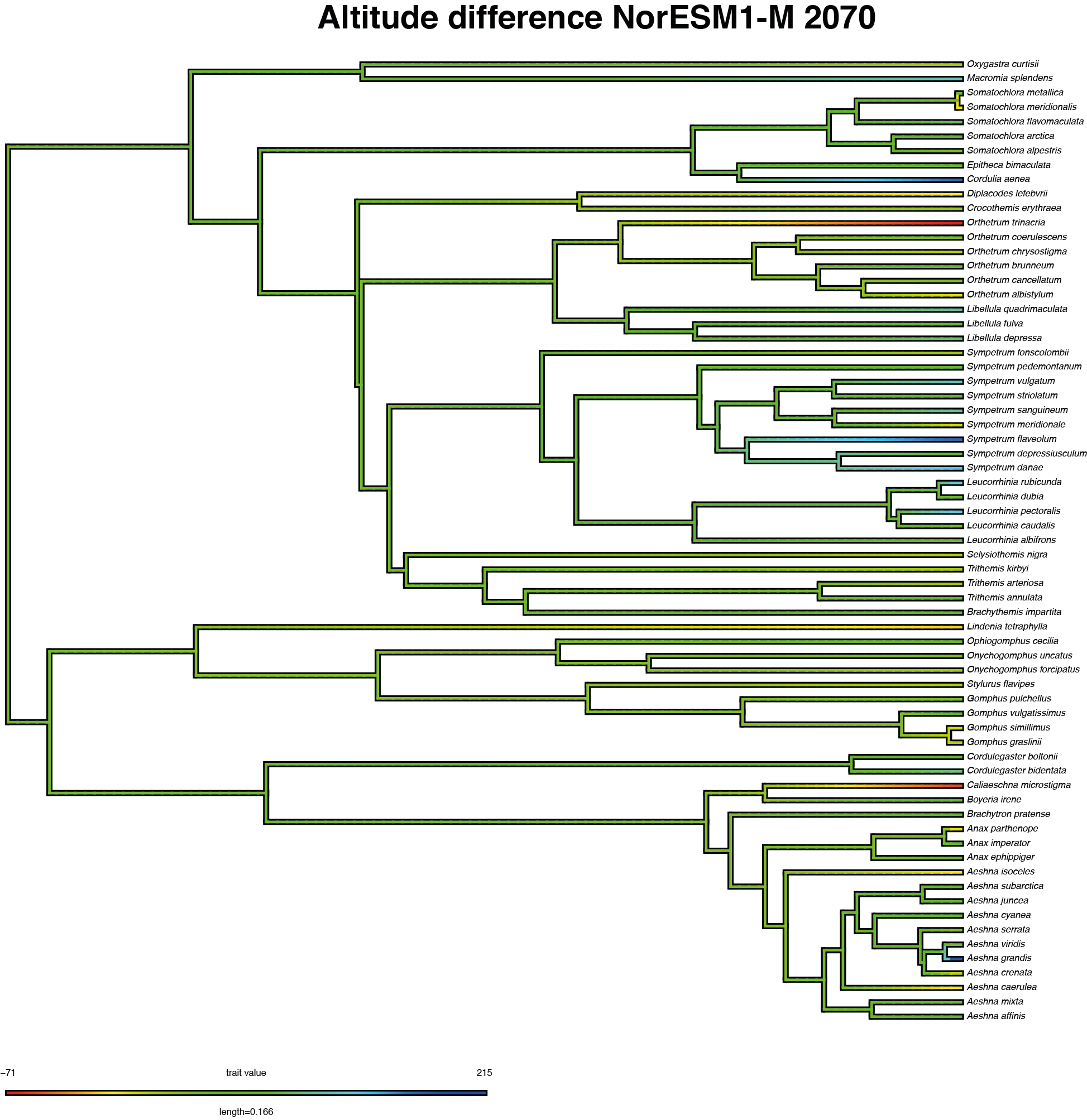
**

**
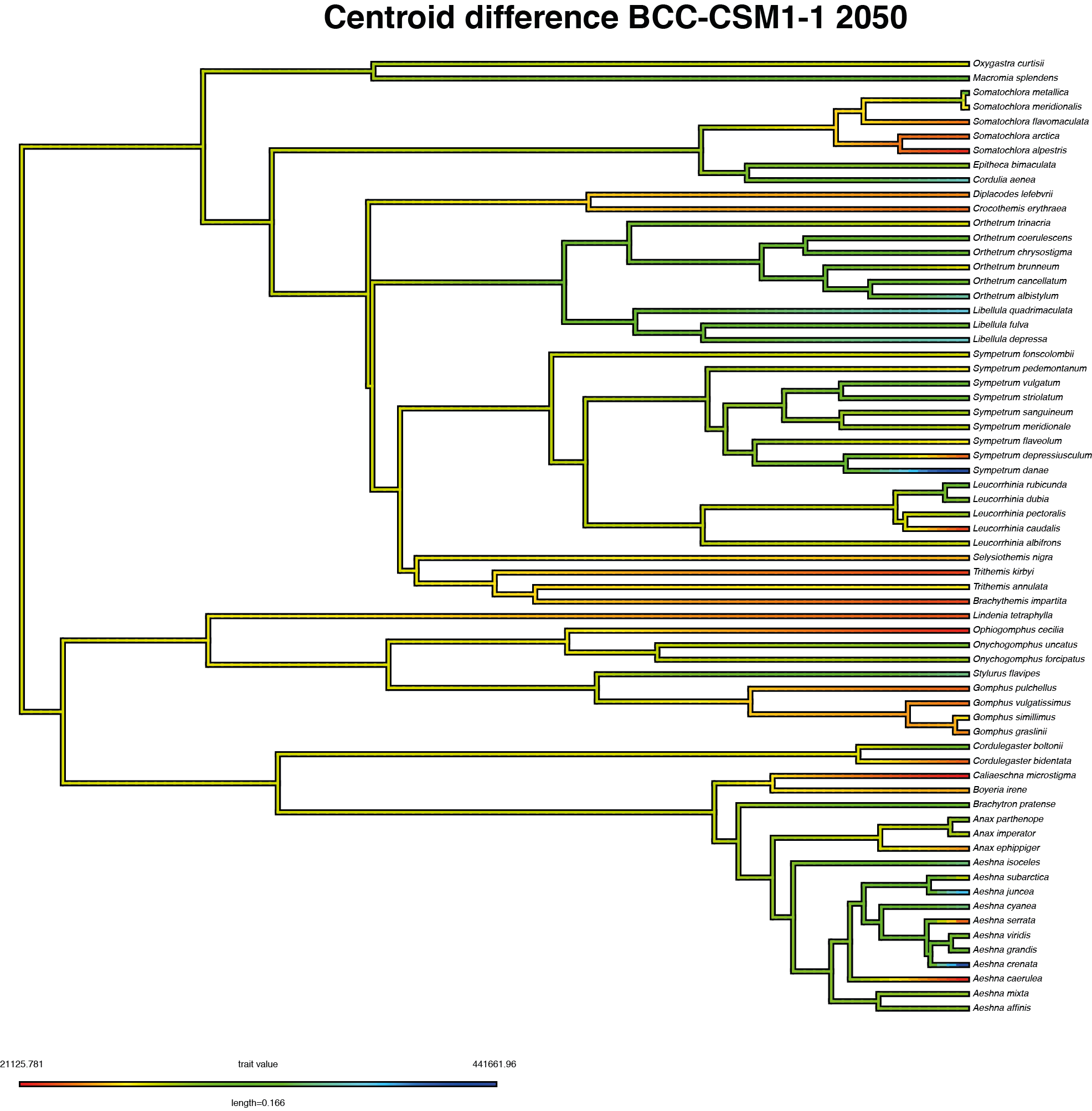
**

**
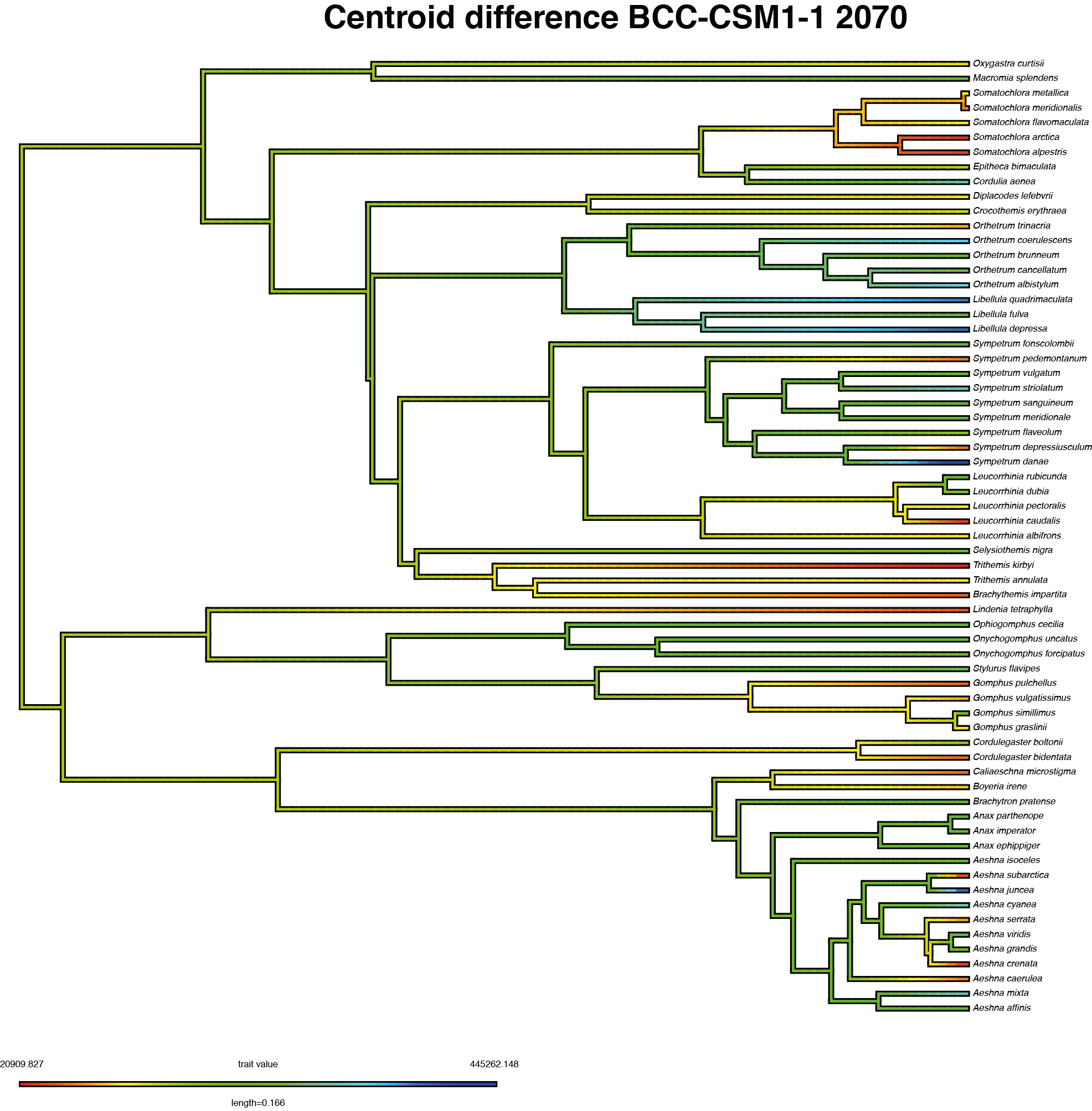
**

**
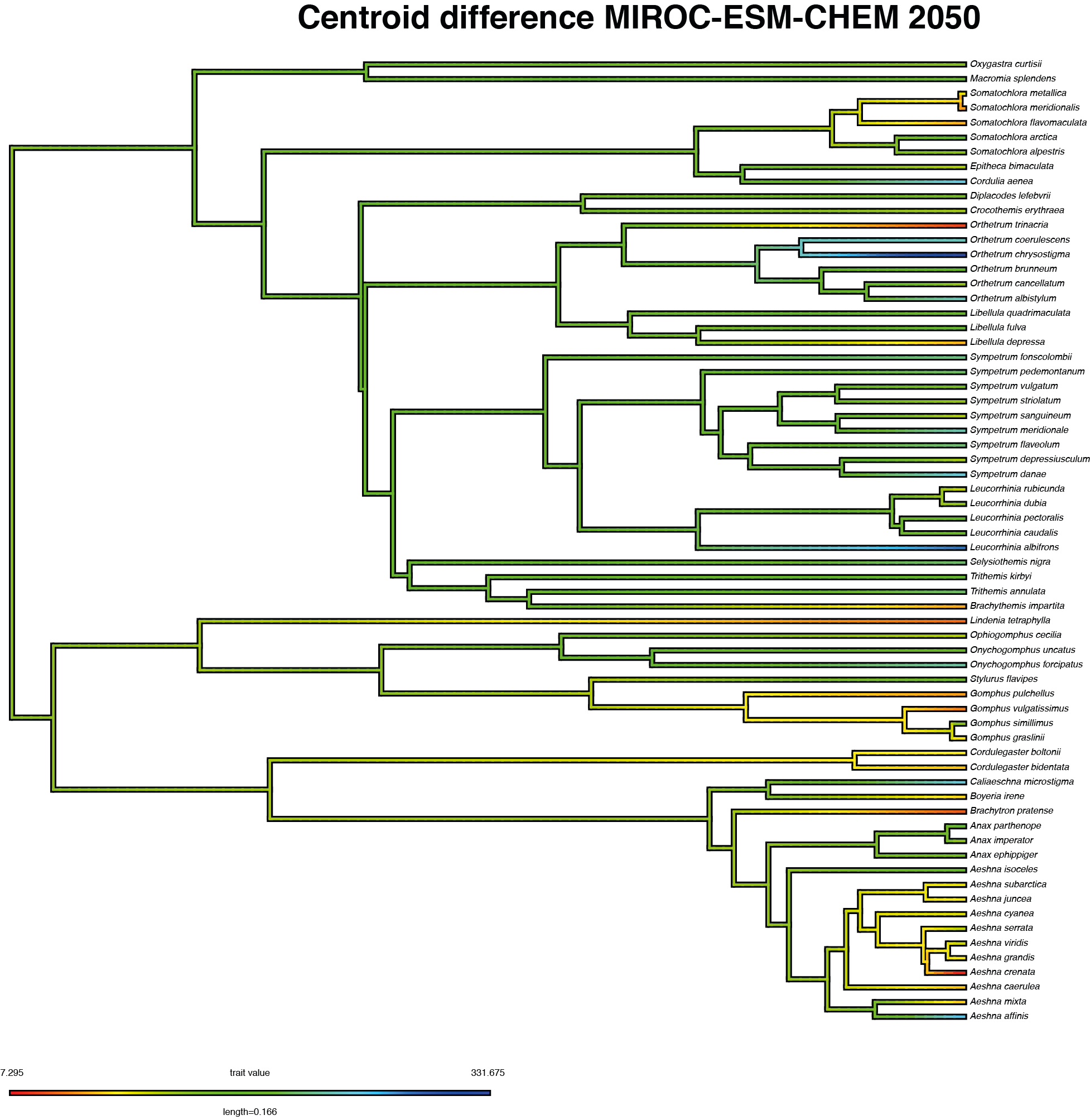
**

**
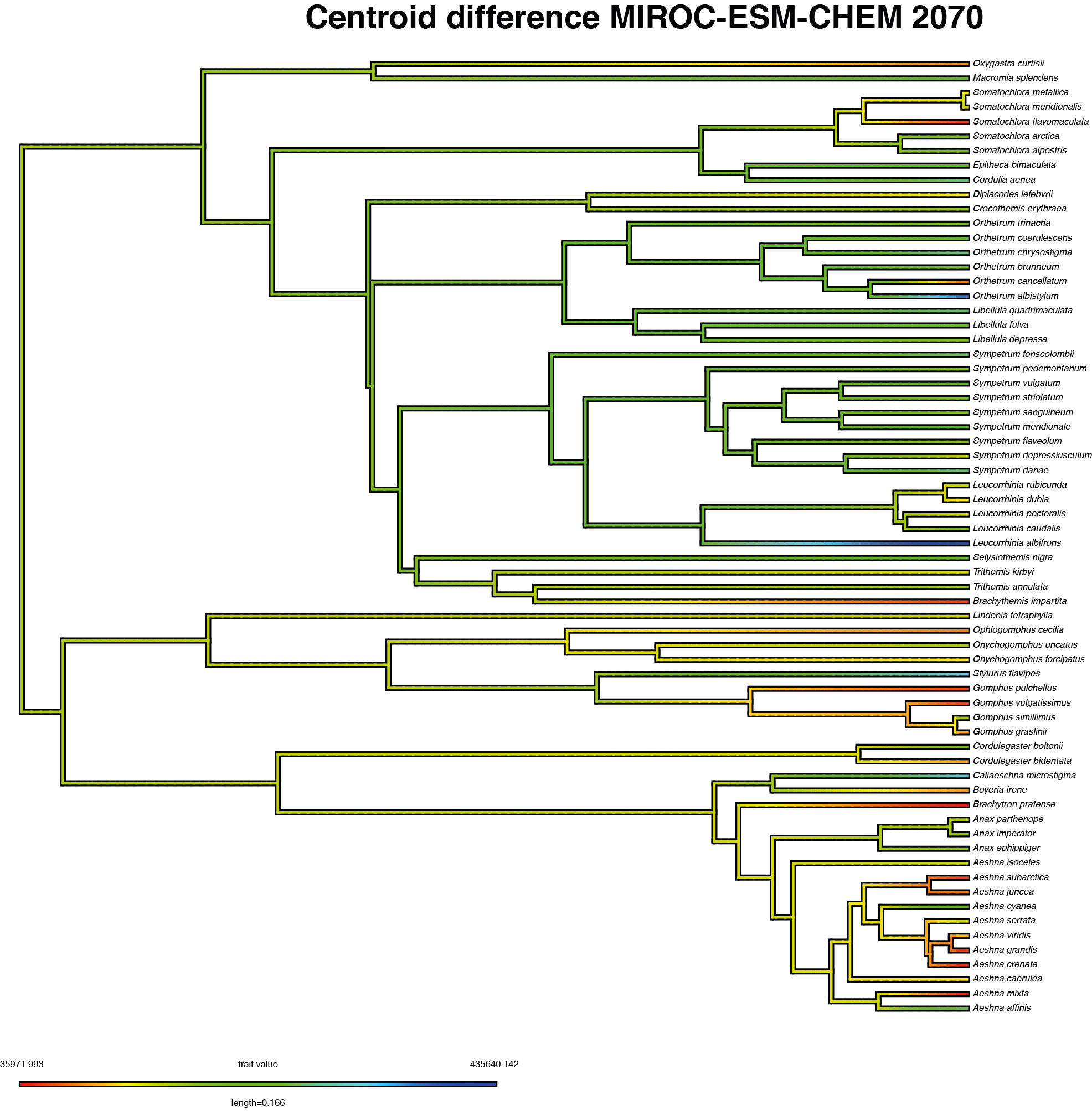
**

**
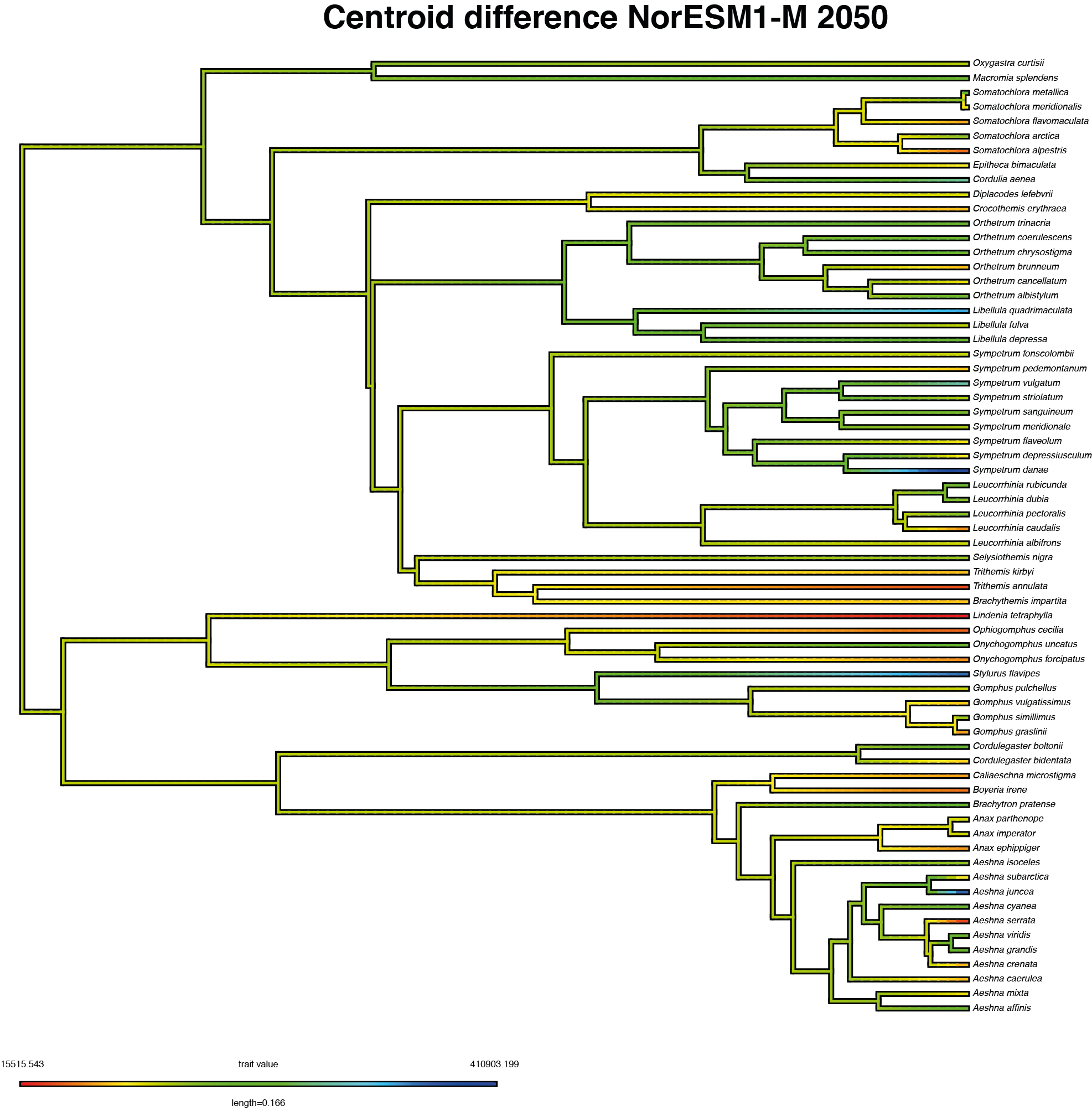
**

**
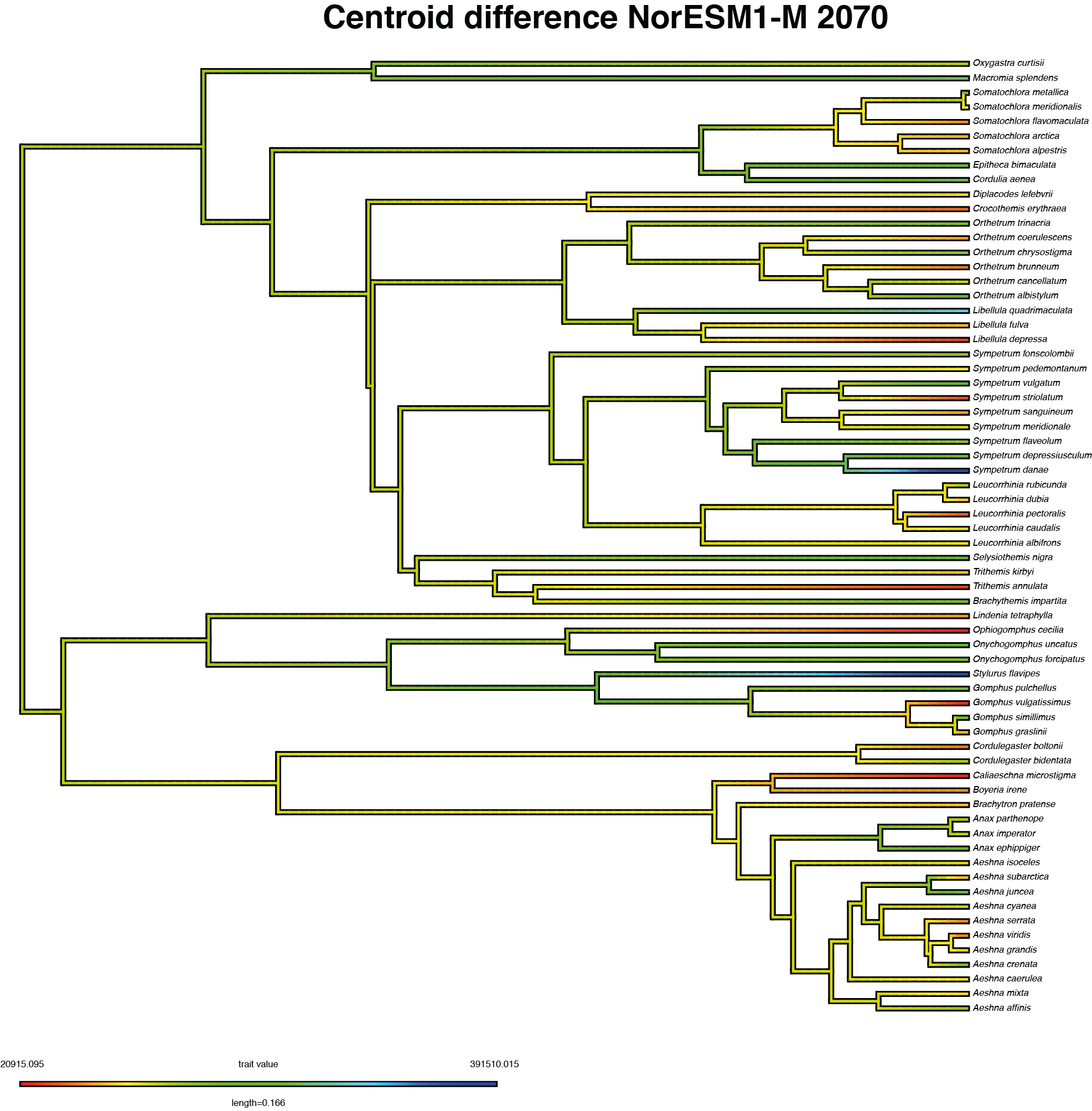
**

**Zygoptera (Suborder)**

**
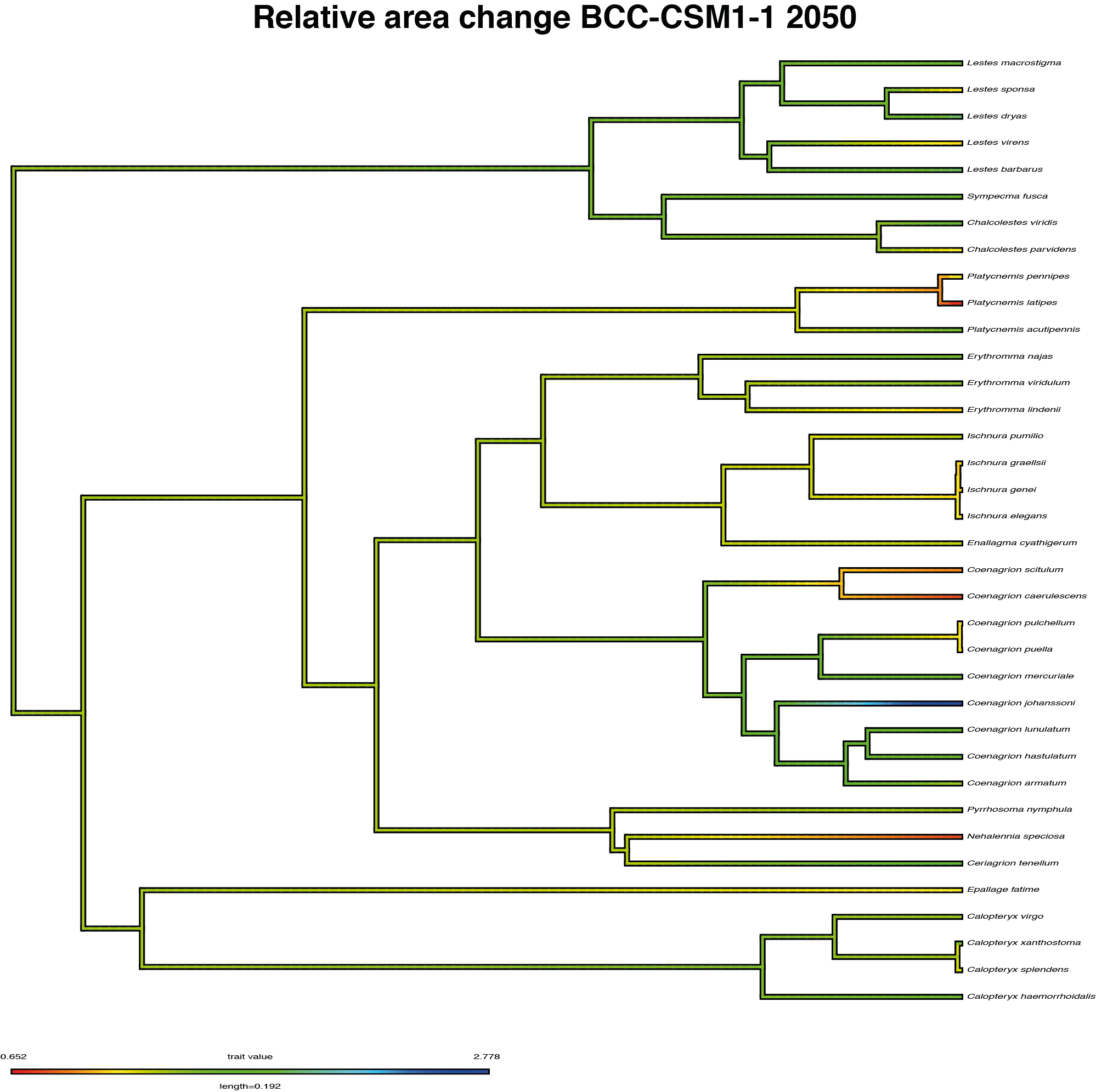
**

**
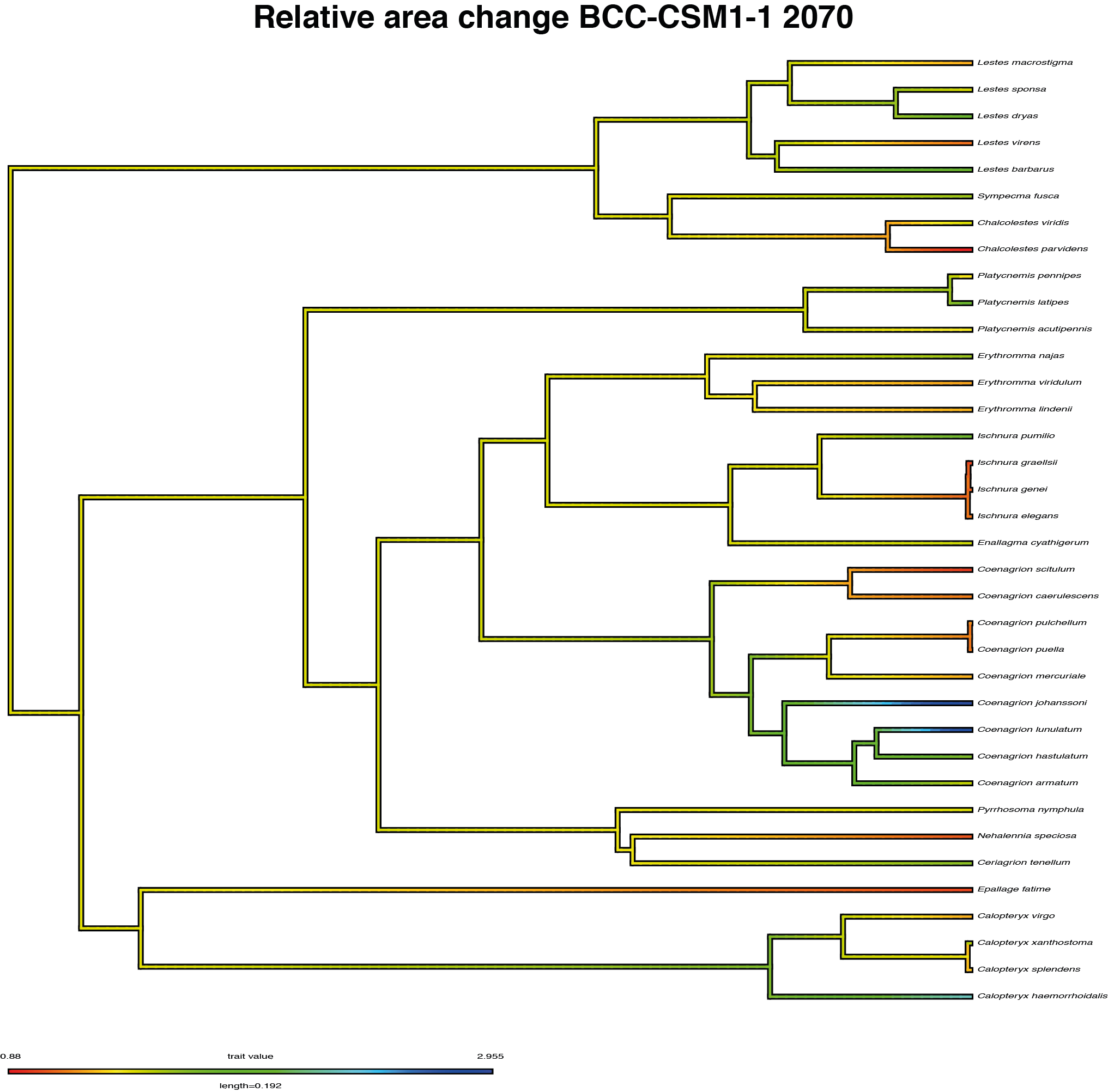
**

**
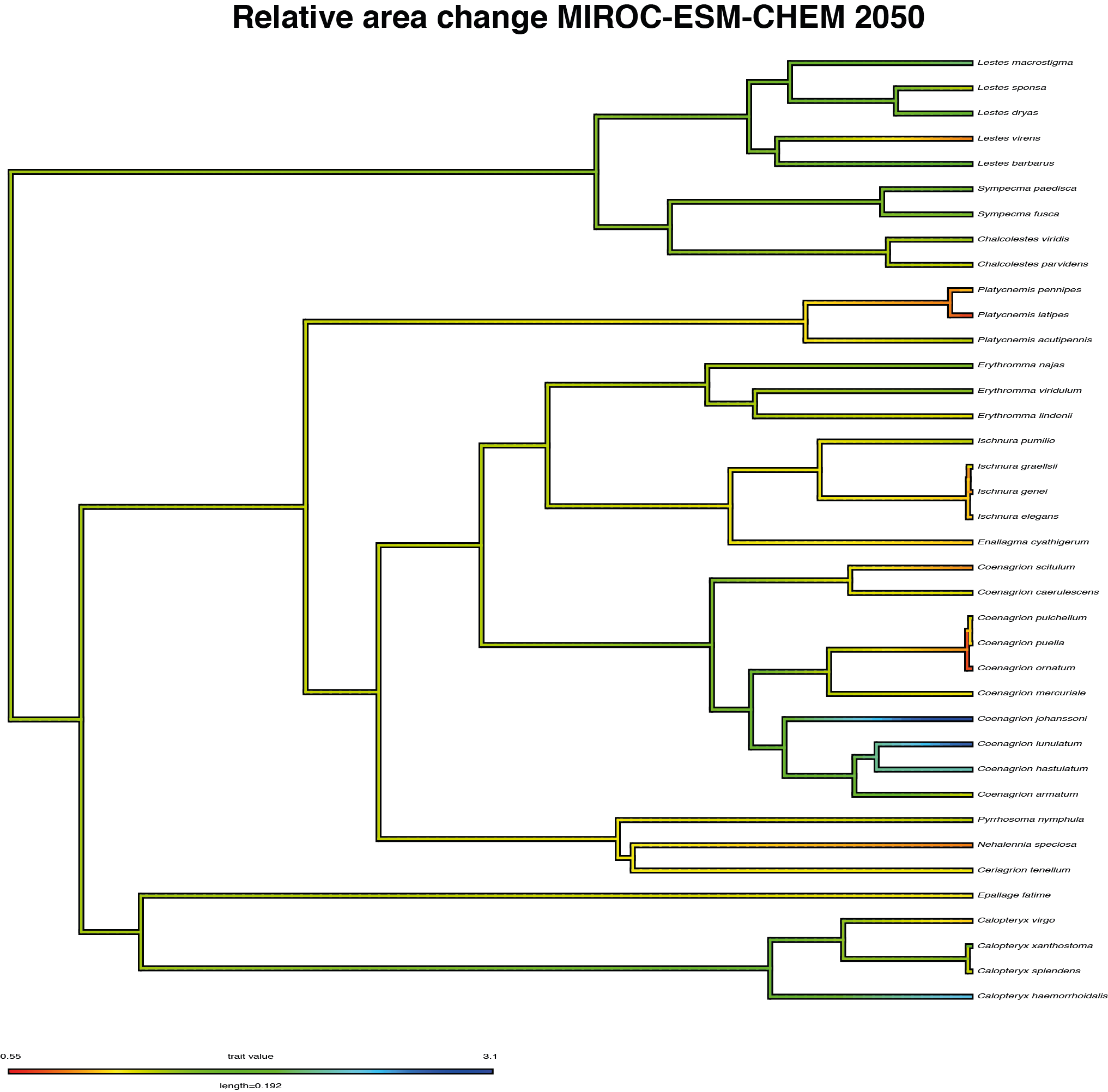
**

**
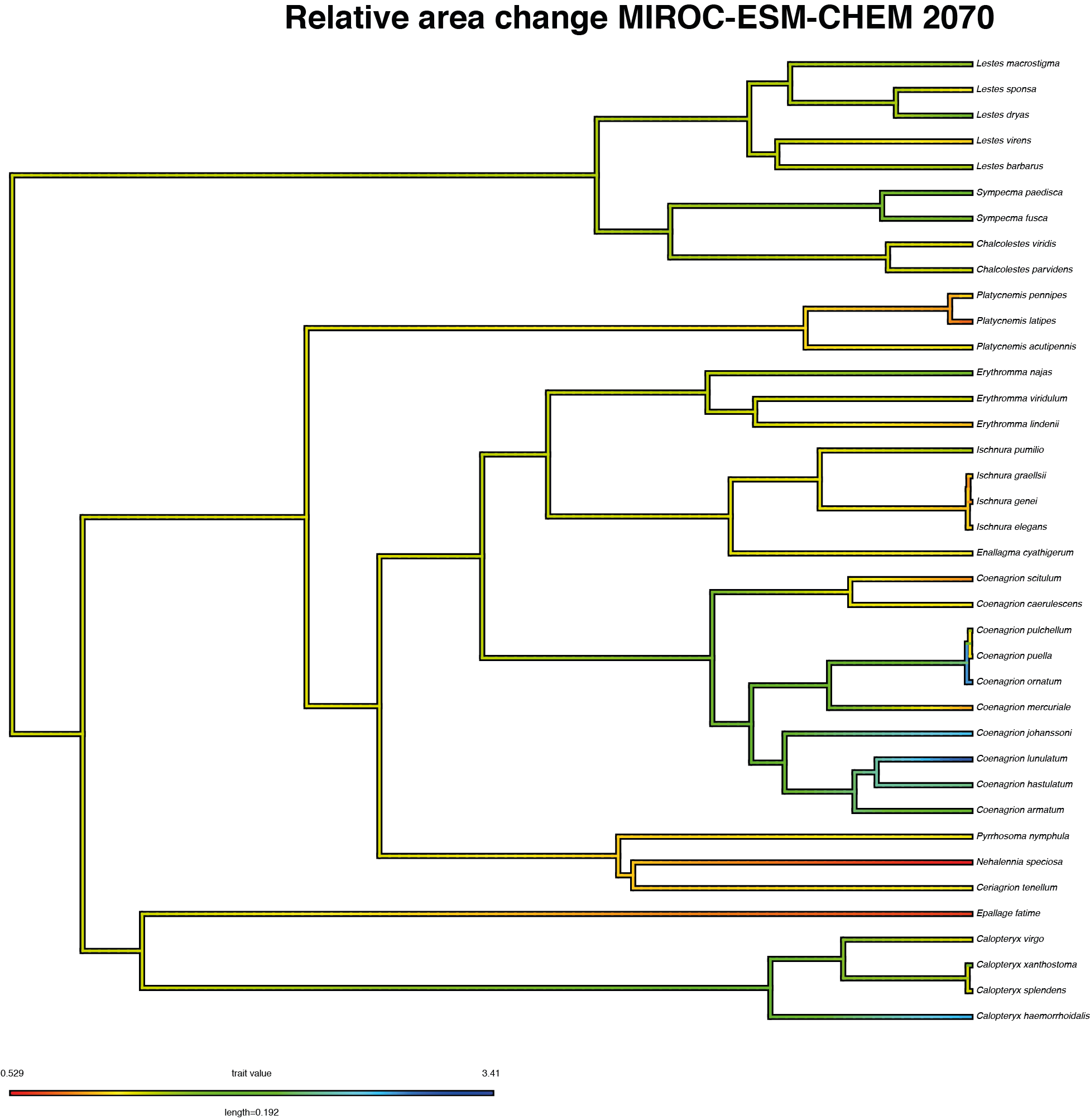
**

**
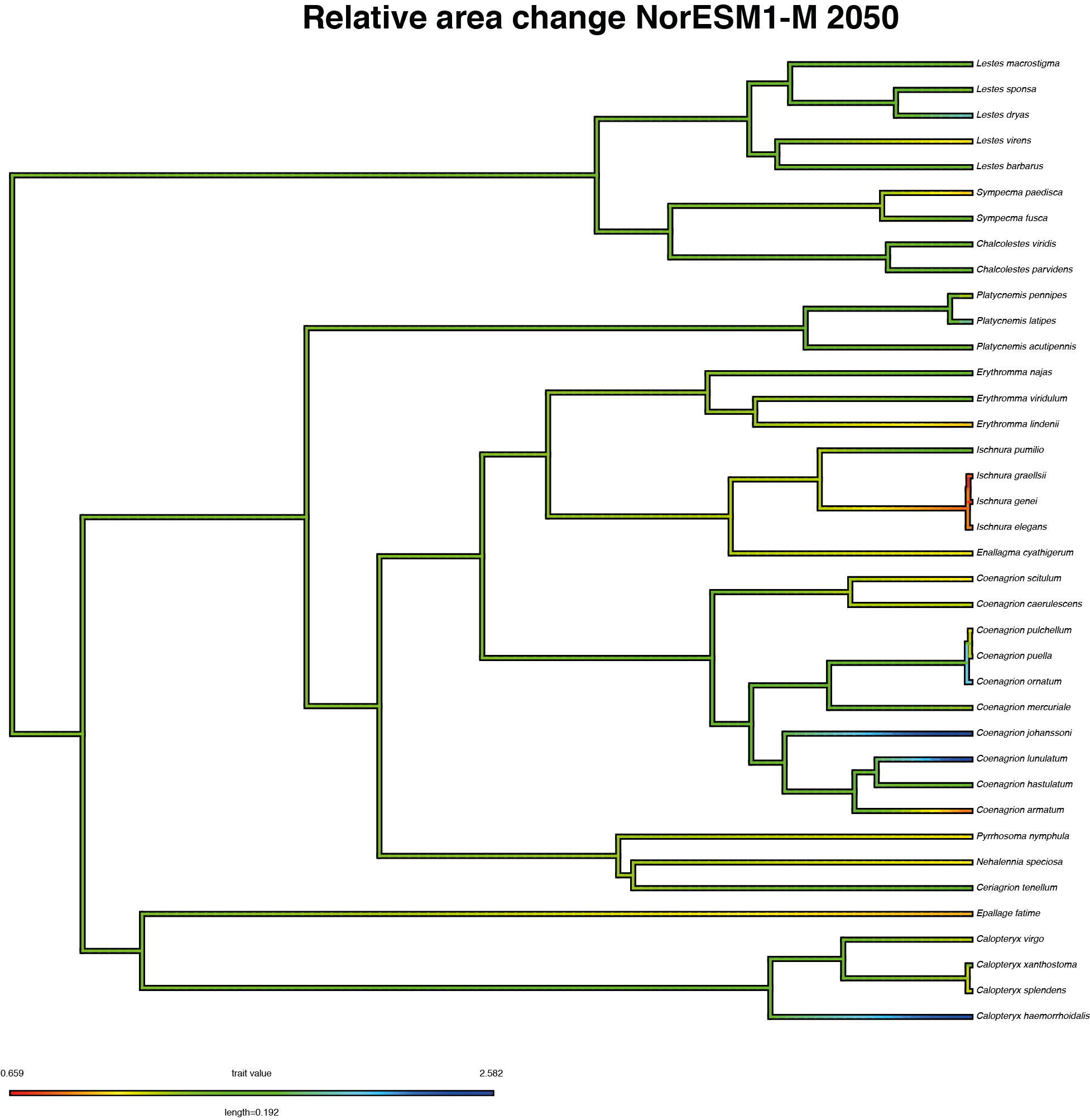
**

**
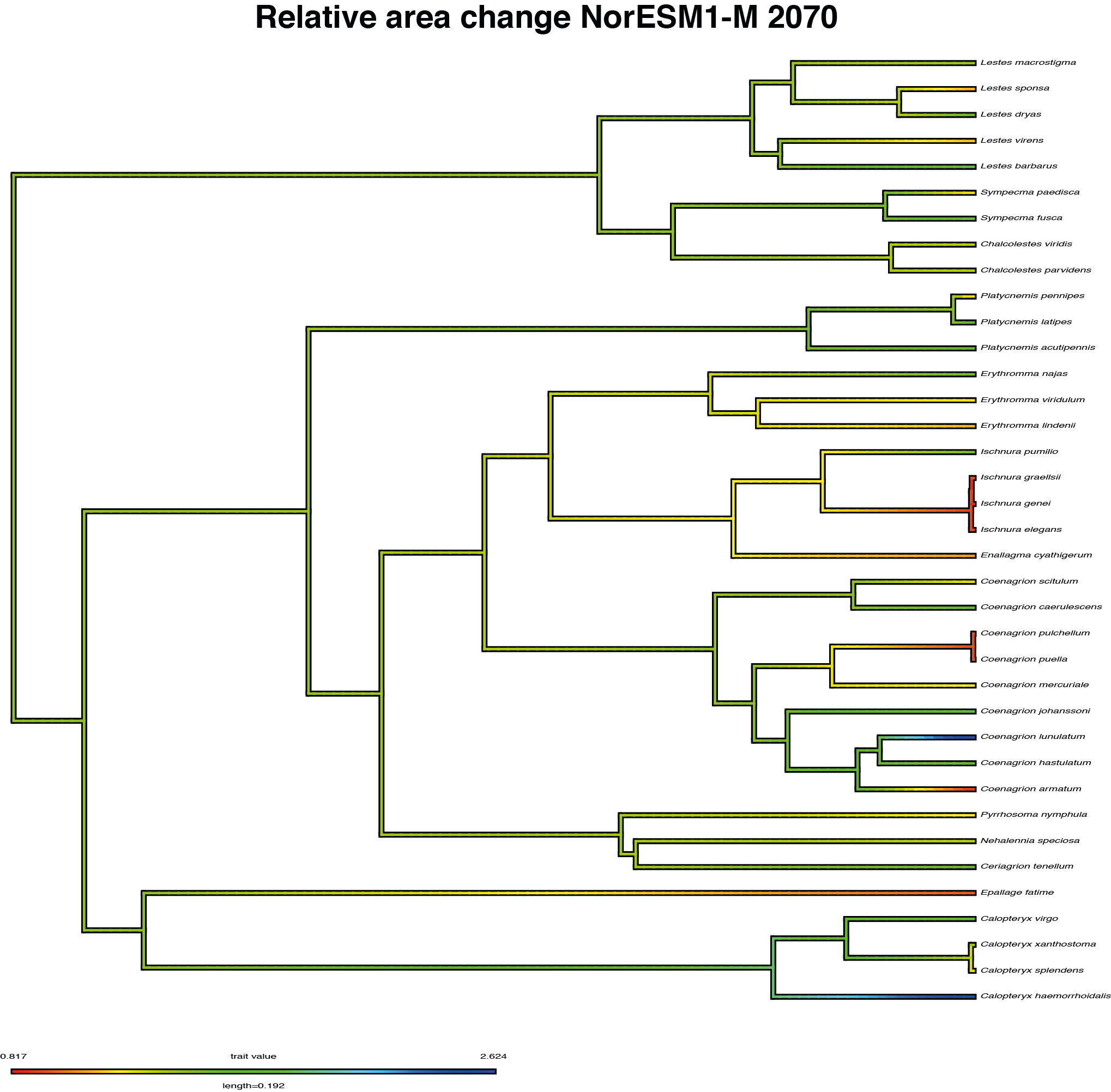
**

**
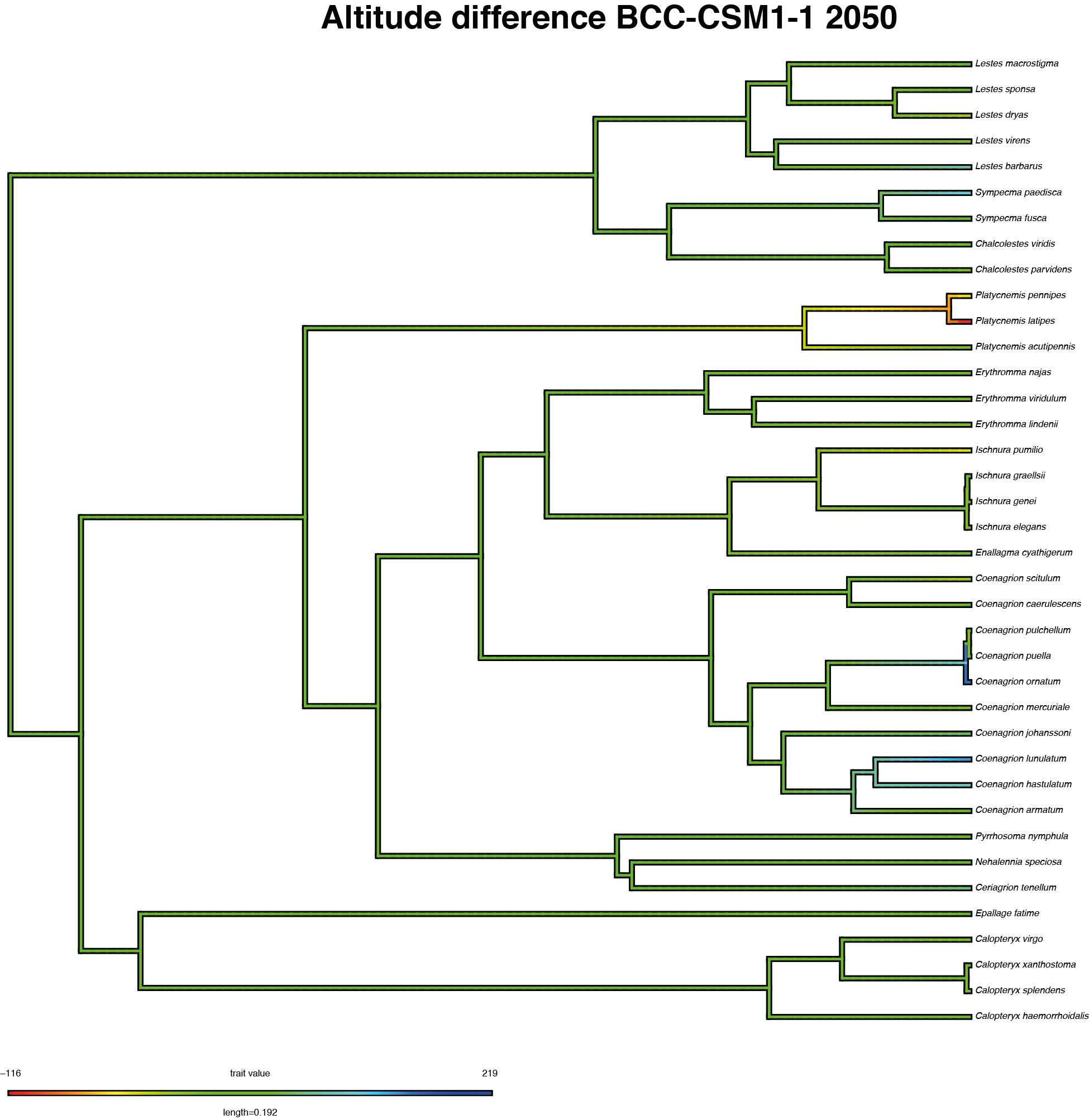
**

**
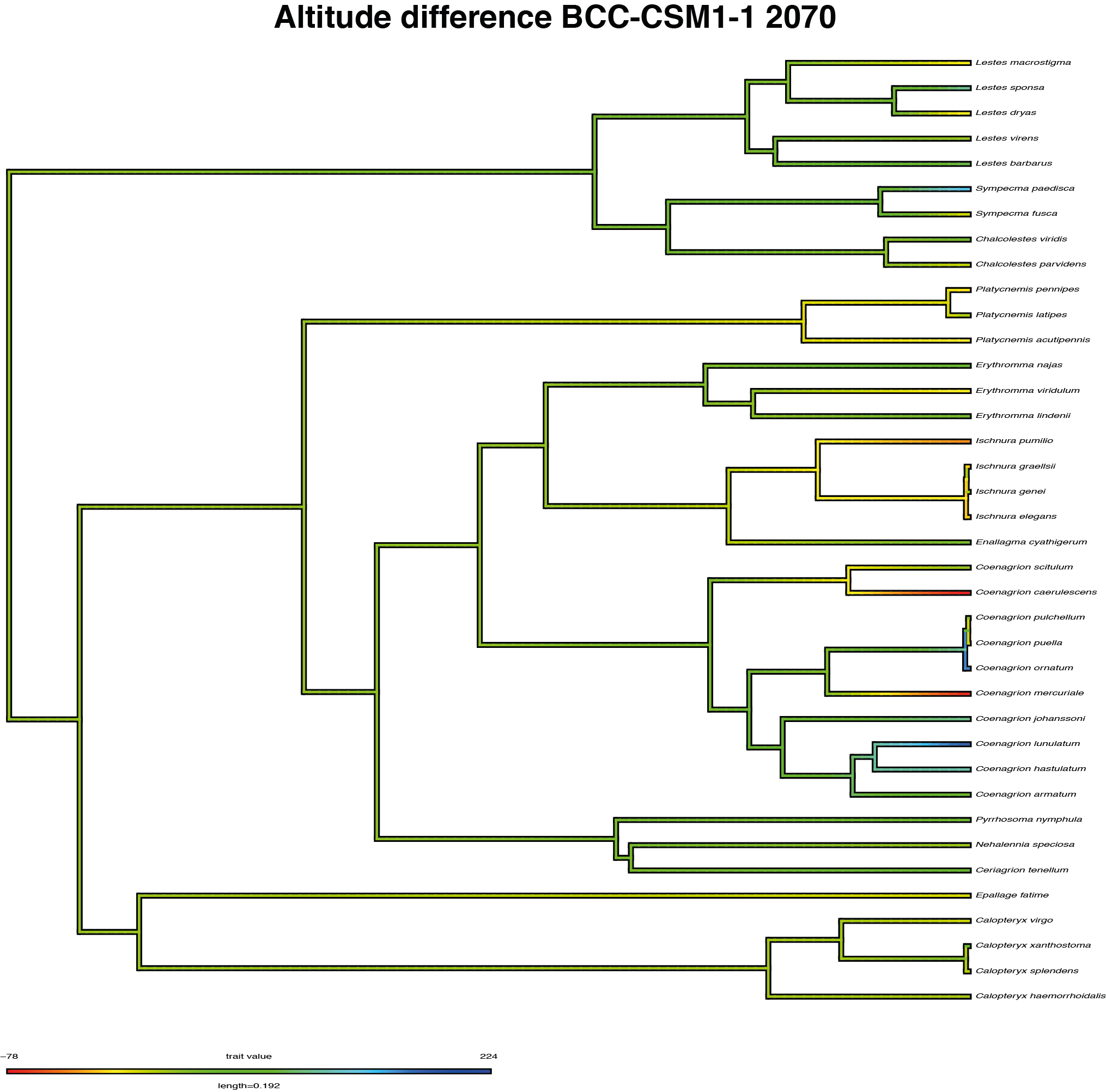
**

**
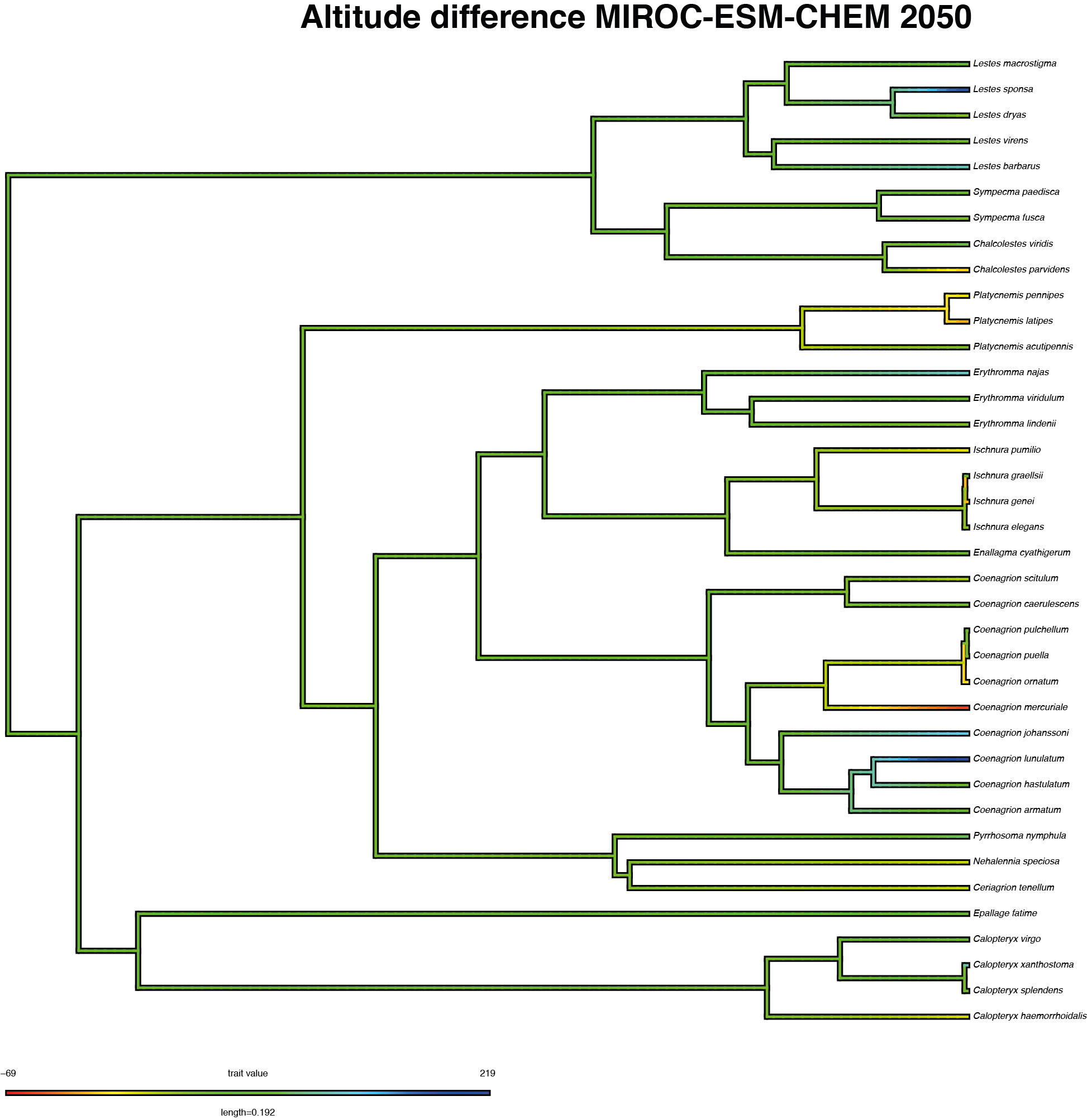
**

**
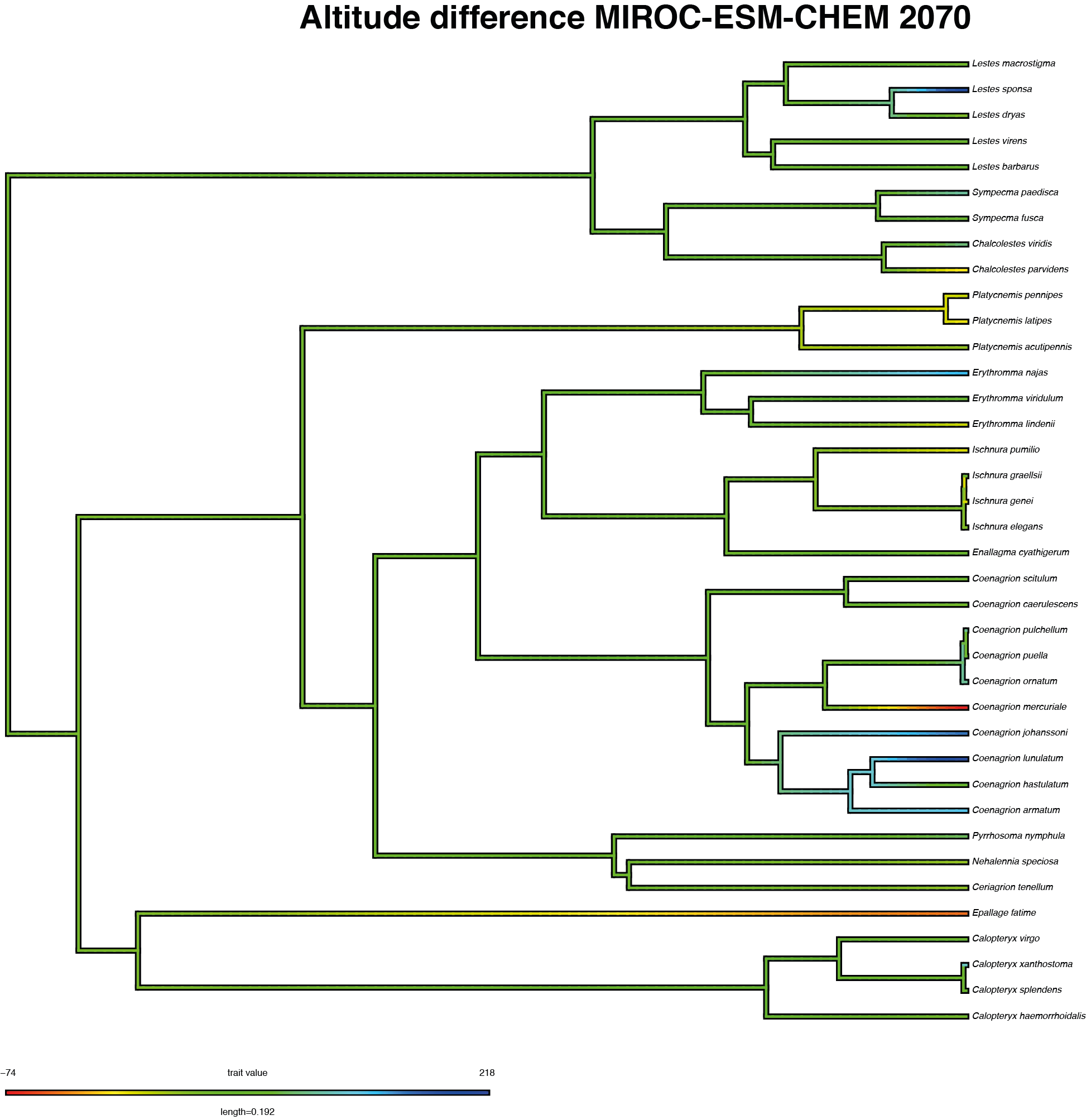
**

**
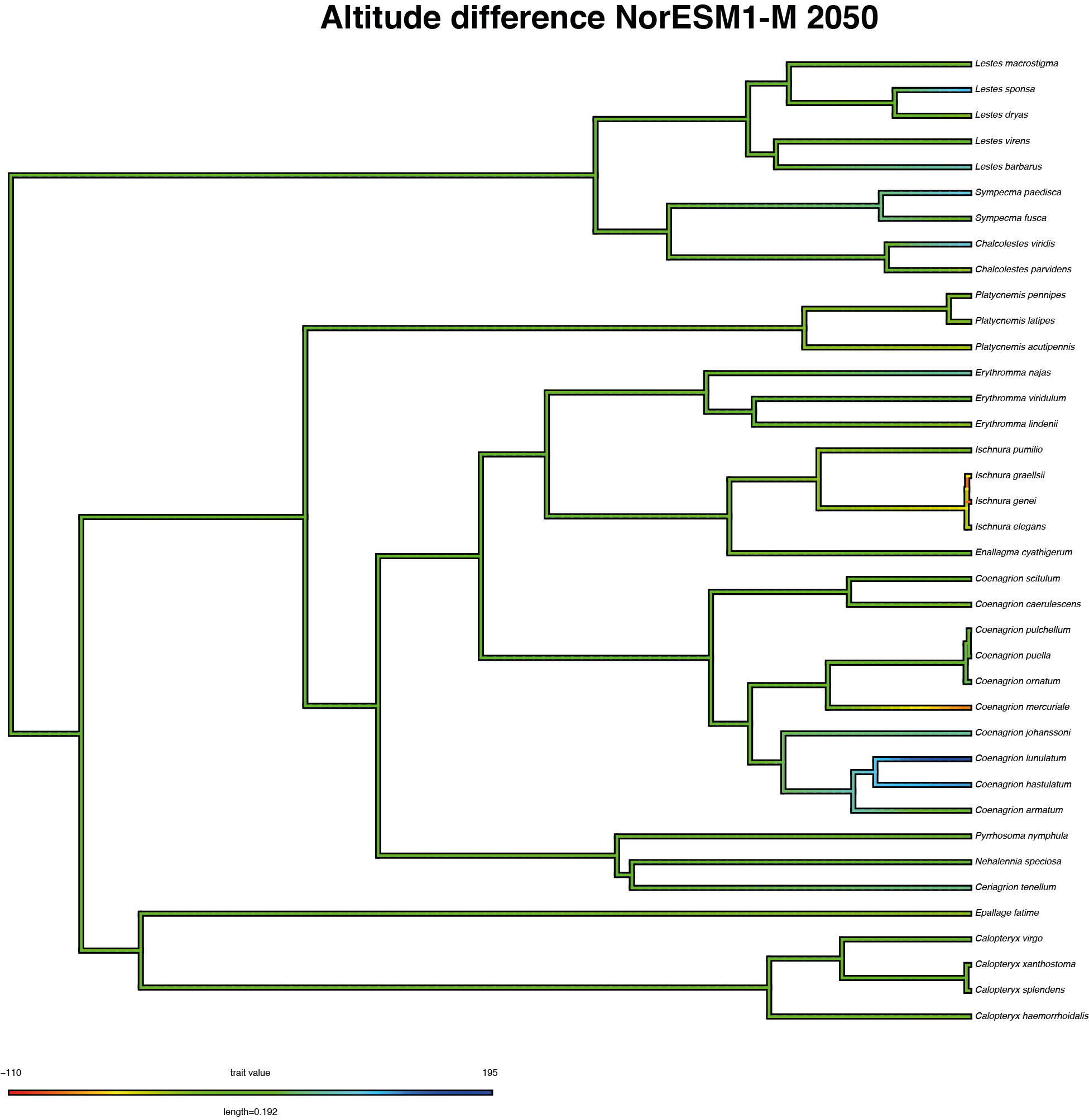
**

**
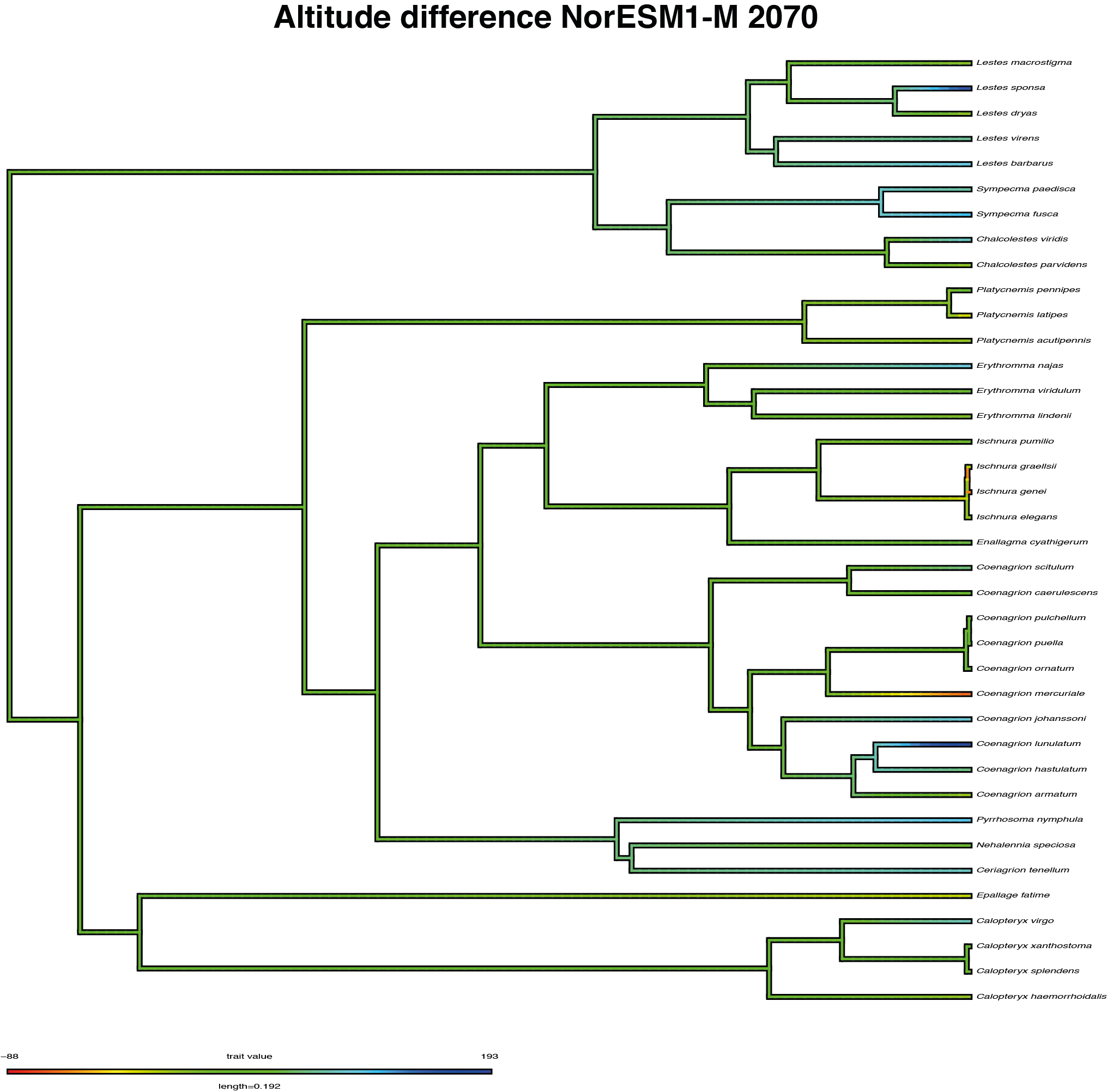
**

**
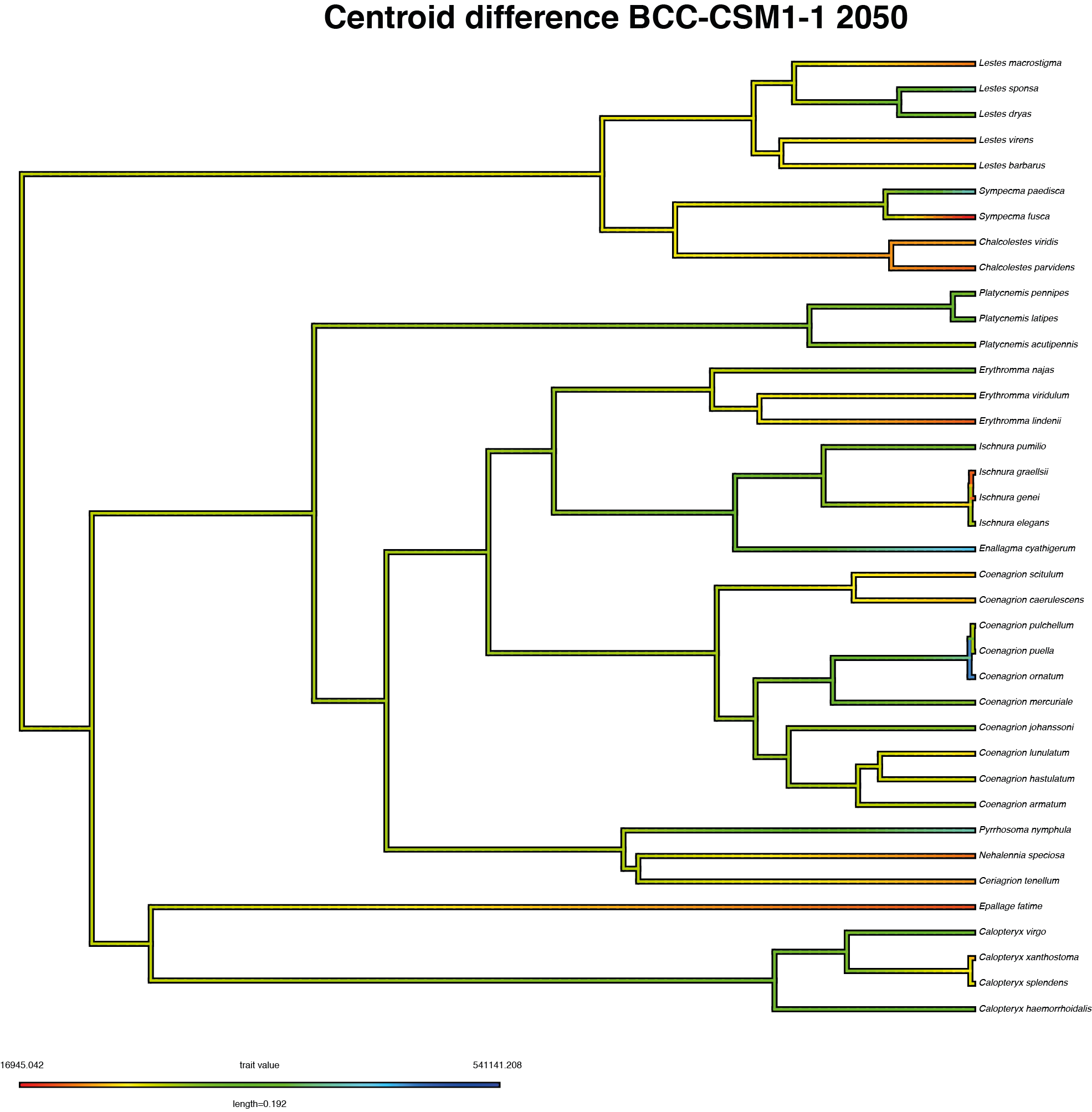
**

**
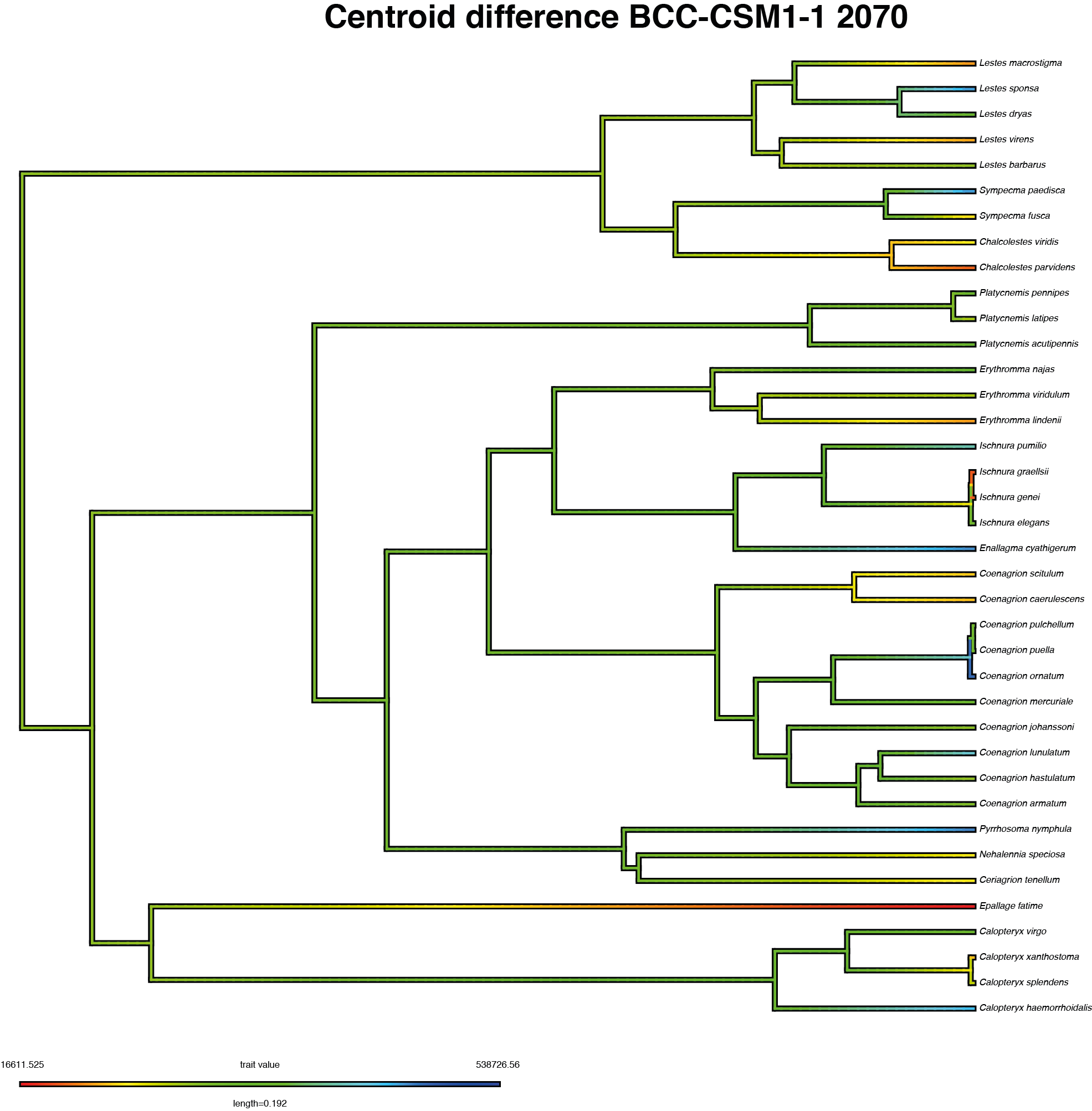
**

**
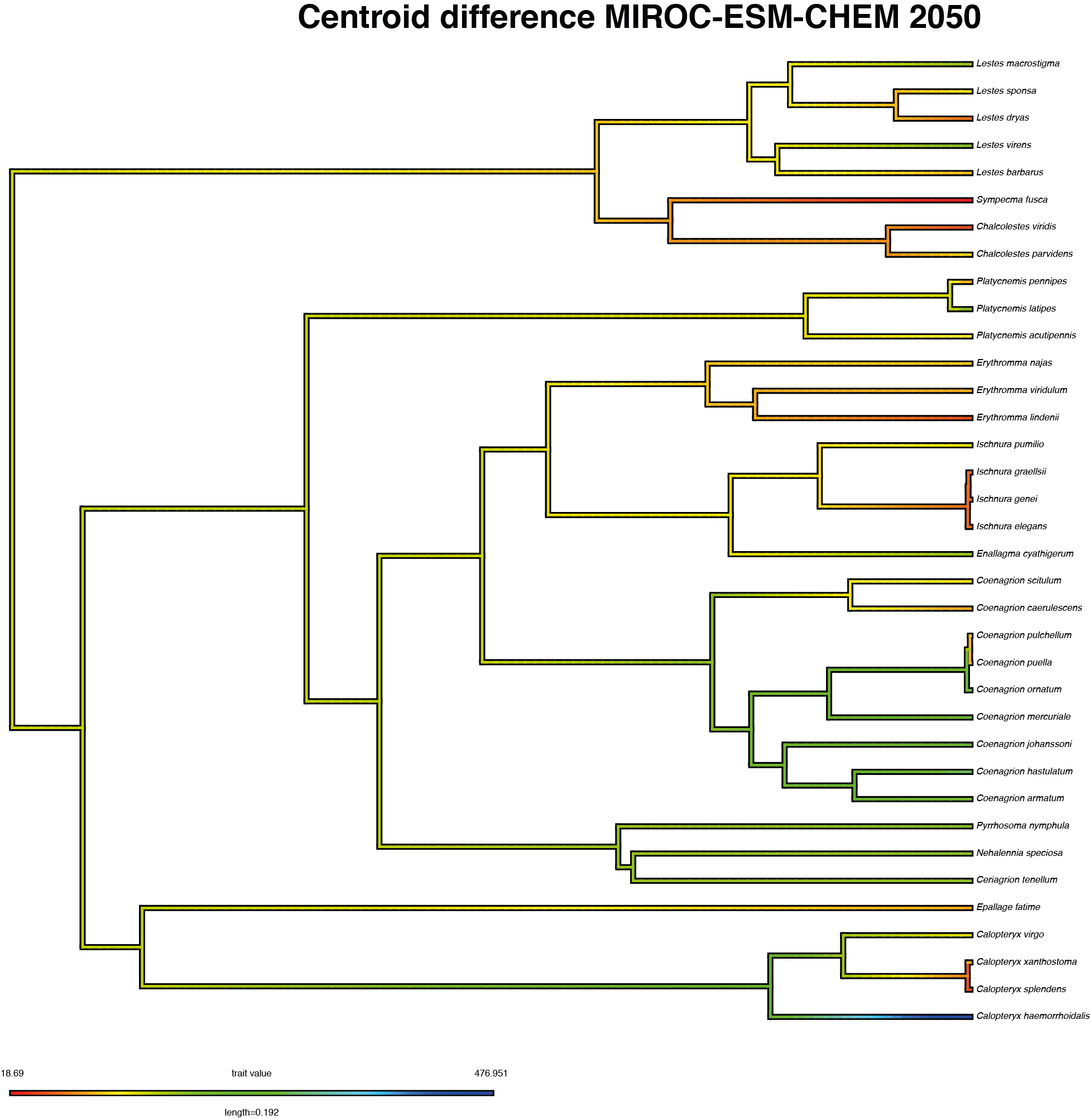
**

**
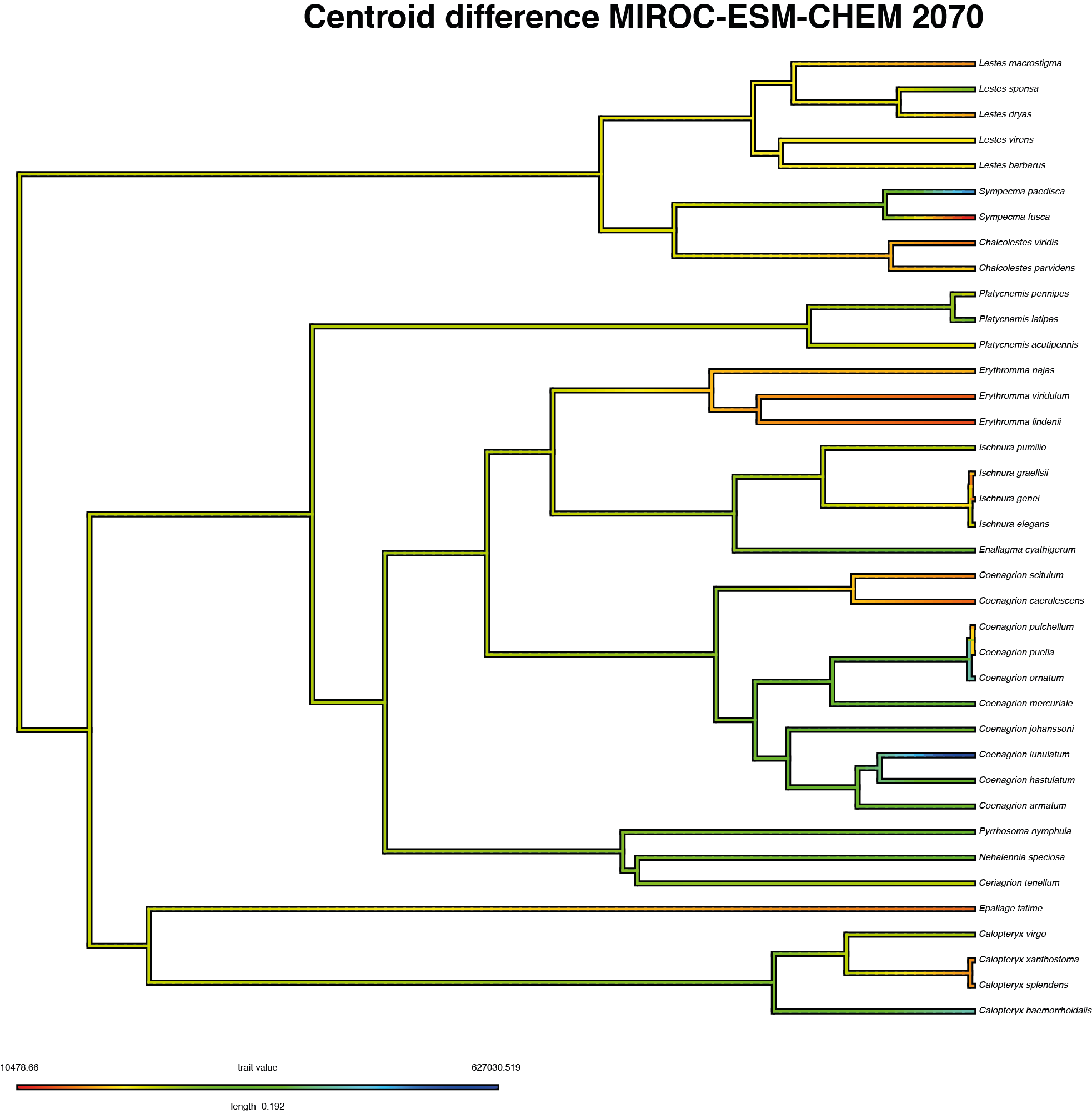
**

**
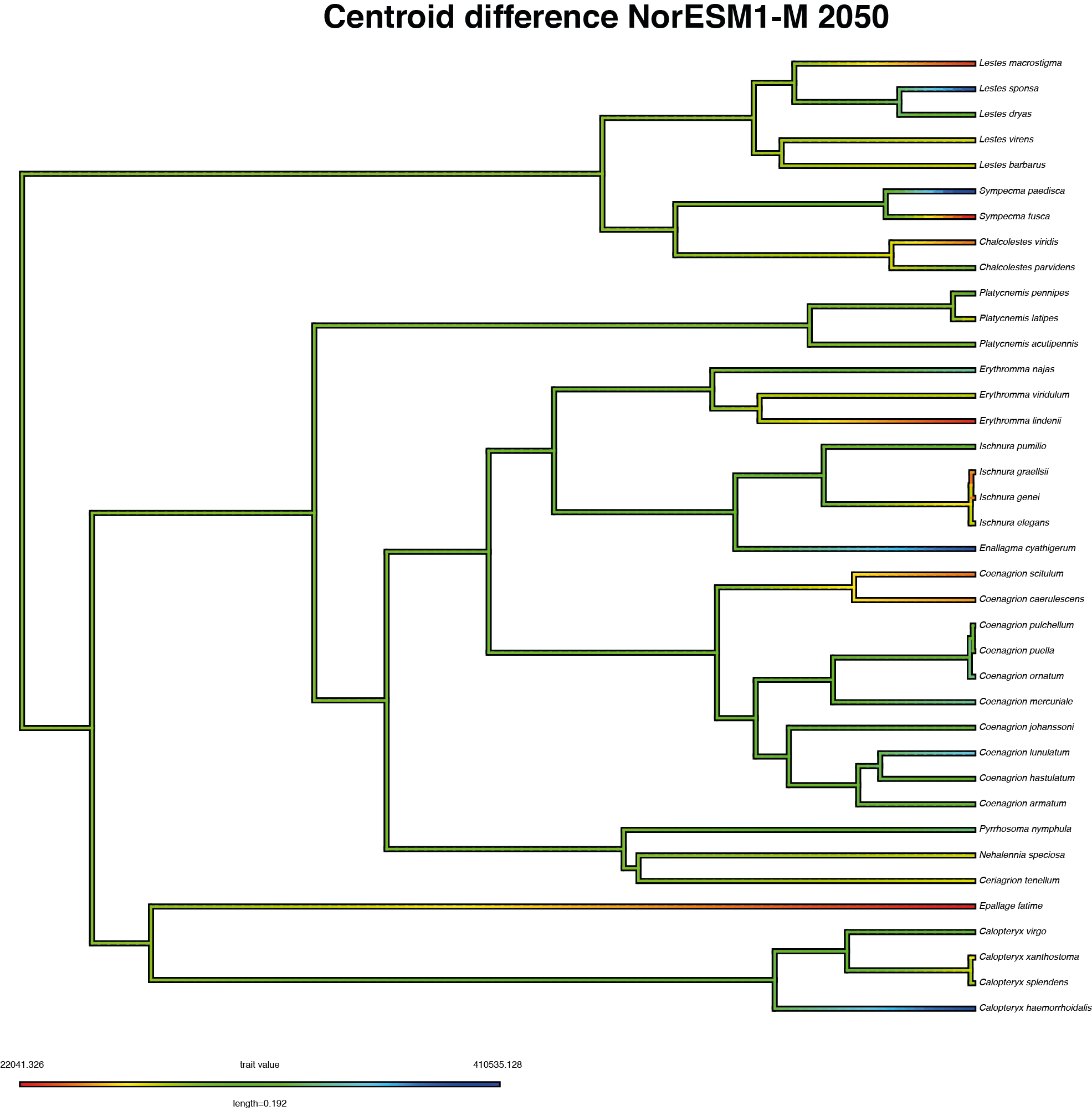
**

**
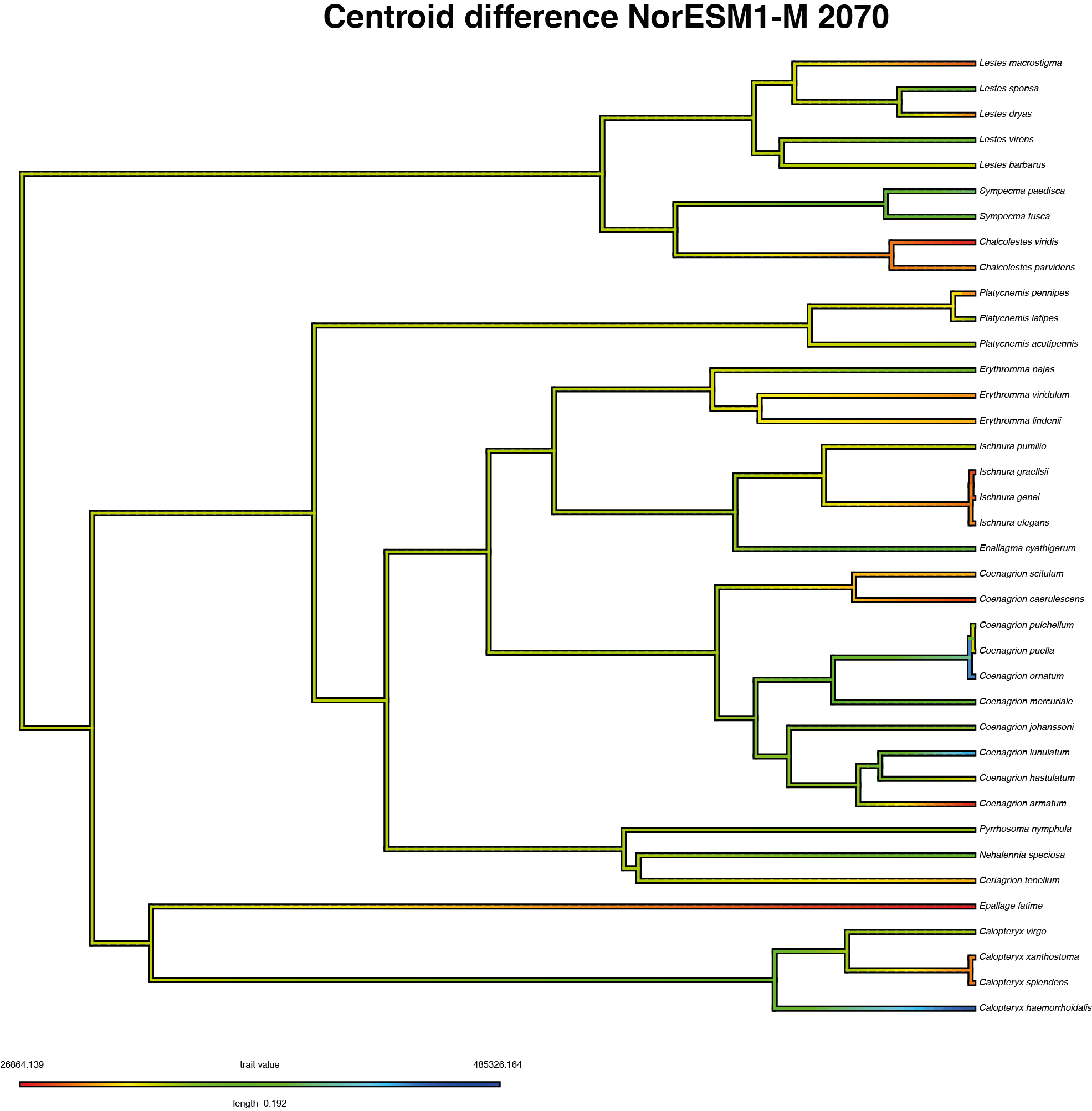
**
